# Oxidative generation of isobenzofurans from phthalans: application to the formal synthesis of (±)-morphine†‡

**DOI:** 10.1039/d4sc05890a

**Published:** 2024-10-19

**Authors:** Mirai Kage, Hiroyuki Yamakoshi, Manami Tabata, Eisaku Ohashi, Kimihiro Noguchi, Takeshi Watanabe, Manato Uchida, Minetatsu Takada, Kazutada Ikeuchi, Seiichi Nakamura

**Affiliations:** a Graduate School of Pharmaceutical Sciences, Nagoya City University 3-1 Tanabe-dori, Mizuho-ku Nagoya 467-8603 Japan nakamura@phar.nagoya-cu.ac.jp

## Abstract

Treatment of phthalan derivatives with *p*-chloranil in dodecane in the presence of molecular sieves at 160–200 °C allowed the generation of unstabilized isobenzofurans, which underwent intramolecular Diels–Alder reaction to give endo cycloadducts exclusively. The cycloaddition turned out to be reversible, providing an equilibrium mixture of endo adducts when heating a substrate with a stereocenter on the tether. We also demonstrated the regioselective allylation of an oxygen-bridged cycloadduct upon exposure to EtAlCl_2_ in the presence of allyltrimethylsilane, and the conversion to Rice's intermediate completed a formal synthesis of (±)-morphine.

## Abstract 2

An oxidative method for generation of isobenzofurans from phthalans, which enables the formal synthesis of morphine, has been developed.

## Graphical abstract

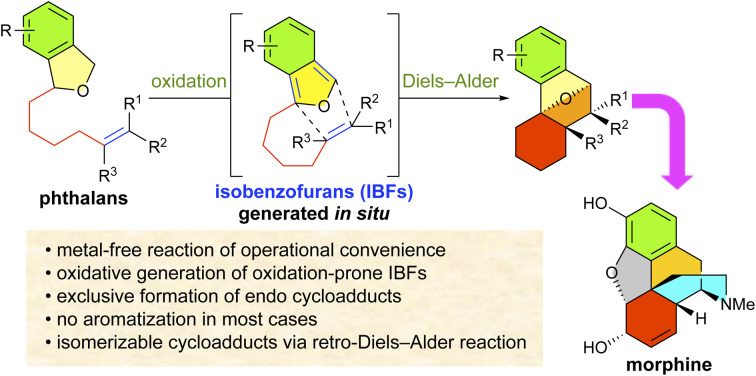

## Introduction

Due to the structural features and the ease of aromatization of Diels–Alder adducts under acidic conditions, isobenzofurans (IBFs) have been recognized as useful intermediates for the preparation of fused polycyclic aromatic compounds.^1^ Despite being aromatic compounds with 10π-electrons, IBFs are extremely reactive and prone to dimerization or polymerization in solution.^2^ While aryl and electron-withdrawing substituents on the furanoid ring stabilize the system, less stable IBFs should be generated *in situ* and used for the following reaction without isolation.

**Scheme 1:**
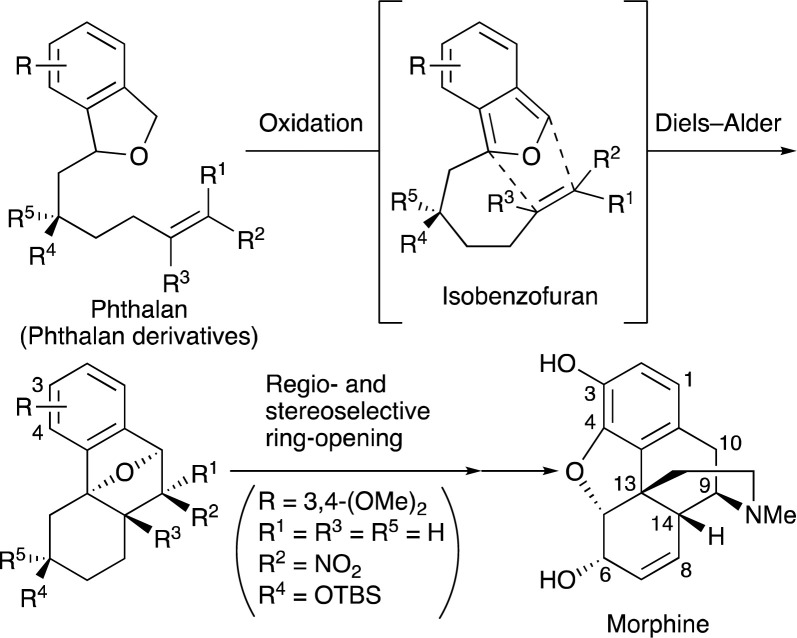
Tandem oxidation/intramolecular Diels–Alder approach to octahydrophenanthrene derivatives and the structure of morphine.

To date, a variety of methods involving retro Diels–Alder reaction,^2*a*,3^ 1,4-elimination of dihydroisobenzofuranols and their ethers,^4^ isomerization of benzalphthalan,^5^ enolization of phthalides,^6^ transannular cyclization of carbenes, carbenoids, or Pummerer cations with adjacent carbonyl groups,^7^ and electrophilic cyclization of *o*-carbonylated phenylacetylenes^8^ have been developed to generate IBFs.^9^ However, oxidation has never been utilized for this purpose except for a few examples,^10^ probably due to the electron-rich nature of IBFs. With the availability of phthalan derivatives in mind,^11^ we wondered whether IBFs could be generated from the corresponding phthalans by oxidation. Intramolecular trapping of IBFs with π-bonds at a suitable position can circumvent the aforementioned stability issue, leading to the formation of benzene-fused, oxygen-bridged polycyclic compounds that would be employed for the syntheses of bioactive natural products^12^ (Scheme 1). In this paper, we report a novel oxidation/intramolecular Diels–Alder (IMDA) strategy for the construction of an octahydrophe-nanthrene skeleton, the synthetic utility of which was demonstrated by the formal total synthesis of (±)-morphine.

## Results and discussion

At the outset of this study, we selected phthalan 1 (ref. 13) as a substrate and investigated the tandem oxidation/IMDA sequence. Although palladium catalysts can be used for dehydrogenative aromatization reactions,^14^ treatment of 1 with Pd/C in 2-methylnaphthalene afforded no reaction and heating at 240 °C led to partial decomposition (Table 1, entry 1). In contrast, the use of 2,3-dichloro-5,6-dicyano-1,4-benzoquinone (DDQ)^15,16^ as a stoichiometric oxidant in CH_2_Cl_2_ at room temperature resulted in a complex mixture of products (entry 2). We then speculated that the IBF generated from 1 could not adopt the folded conformation 2 at room temperature. This hypothesis was validated by the observation that cycloadduct 3 was obtained as a single isomer in 20% yield when the reaction was performed in dodecane at 100 °C (entry 3). However, competitive overoxidation under these conditions was suggested by the formation of ketoaldehyde 4 as a byproduct. This overoxidation was suppressed by switching the oxidant from DDQ to less reactive *p*-chloranil, although the reaction was quite slow and the product yield (23%) was comparable at this temperature due to the 40% recovery of 1 after a prolonged reaction time (10 h, entry 4). An examination of the temperature profile of the reaction revealed that the product yield was improved to 65% by raising the temperature to 180 °C, but the reaction at a higher temperature (200 °C) afforded no discernible benefits (entries 4–6). A significant solvent effect exists in this transformation; dodecane proved to be the solvent of choice for this transformation, whereas the reaction in *o*-dichlorobenzene shortened the reaction time (entries 5 *vs.* 7, 8). While the addition of 2,6-di-*tert*-butyl-4-methylphenol (BHT) as a radical scavenger resulted in a low yield, we were gratified to find a beneficial effect of molecular sieves (MS),^17^ providing cycloadduct 3 in 91% yield by the use of 3 Å MS (entries 9–12). It should be mentioned that naphthalene derivatives arising from aromatization were not detected under these conditions. The stereochemical assignment for cycloadduct 3 was determined by the diagnostic ^1^H NOE correlation between H_g_ and H_h_.^18^

**Table 1:** Oxidation/IMDA sequence using phthalan 1^a^

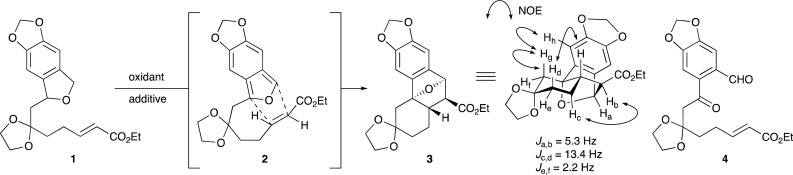						
Entry	Oxidant	Solvent	Additive	Temp. (°C)	Time (h)	Yield (%)
1	Pd/C	2-Methylnaphthalene		240	6	0^b^
2	DDQ	CH_2_Cl_2_		25	1	0^b^^,^^c^
3	DDQ	Dodecane		100	8	20^b^^,^^c^
4	*p*-Chloranil	Dodecane		100	10	23^b^
5	*p*-Chloranil	Dodecane		180	5	65
6	*p*-Chloranil	Dodecane		200	2	62
7	*p*-Chloranil	*o*-C_6_H_4_Cl_2_		180	0.5	31^b^
8	*p*-Chloranil	Triglyme		180	6	0^b^
9	*p*-Chloranil	Dodecane	BHT	180	7	38^b^
10	*p*-Chloranil	Dodecane	3 Å MS	180	5	91
11	*p*-Chloranil	Dodecane	4 Å MS	180	5	84
12	*p*-Chloranil	Dodecane	5 Å MS	180	5	77

a All reactions were carried out on a 0.13 mmol scale with 1.3 equivalents of the oxidant at a concentration of 0.01 M.

b TLC analysis indicated that unreacted starting material remained.

c The formation of overoxidation product 4 was observed.

**Scheme 2:**
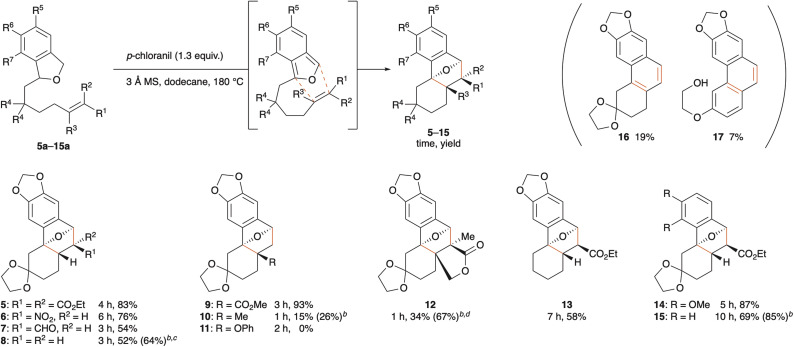
Substrate scope.^*a a*^ All reactions were carried out on a 0.13 mmol scale with 1.3 equivalents of the oxidant at a concentration of 0.01 M. ^*b*^ Yields in parentheses are based on recovered starting material. ^*c*^ Naphthalene and phenanthrene derivatives 16 and 17 were obtained as byproducts in 19% and 7% yields, respectively. ^*d*^ At a concentration of 1 mM.

Having optimized the reaction conditions, the scope of the tandem oxidation/IMDA sequence was explored (Scheme 2). As expected, reactions of phthalans having trisubstituted or nitro-substituted olefins gave the corresponding endo cycloadducts 5 and 6 in good yields. It is noteworthy that chemoselective oxidation could be attained under these conditions, leaving the formyl group intact, albeit in modest yield of aldehyde 7 (54%). The electron-withdrawing substituent on the olefin was found to be unnecessary, but the tandem reaction of phthalan 8a was accompanied by aromatization of the 11-oxatricyclo[6.2.1.0^1,6^] undecane moiety, giving naphthalene and phenanthrene derivatives 16 and 17 as byproducts in 19% and 7% yields, respectively. A quaternary stereocenter could be created by using 1,1-disubstituted alkenes 9a and 10a as substrates: even the alkene bearing an electron-donating methyl group gave cycloadduct 10, albeit in 15% yield, whereas the hetero atom-substituted alkene in phthalan 11a did not undergo cycloaddition in accord with the general trend, resulting in decomposition. The tetrasubstituted alkene in phthalan 12a could also serve as a dienophile for the sequential reaction, allowing for the simultaneous construction of three contiguous quaternary stereocenters.^19^ Although Kanematsu and co-workers reported that IMDA reaction of an IBF lacking substitution on the tether met with failure due to rapid decomposition of the IBF,^20^ unsubstituted product 13 was obtained according to this protocol. With regard to substituents on the benzene ring, an *ortho*-methoxy group in phthalan 14a did not reduce the product yield, and this transformation could be applicable even to the unsubstituted substrate 15a.

**Scheme 3:**
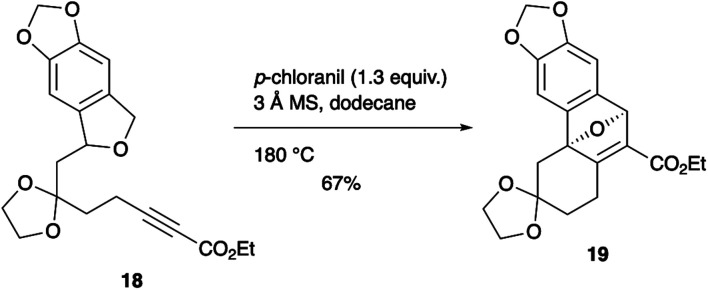
Tandem oxidation/intramolecular Diels–Alder reaction of ynoate 18.

The scope of the present method is not limited to the use of alkene dienophiles (Scheme 3). The reaction of alkyne 18 furnished conjugated ester 19 (67% yield), the alkene moiety of which can be used as a handle for further functionalization.

To demonstrate the synthetic utility of our method, we then addressed the formal synthesis of (±)-morphine. Due to their important pharmacological properties and strained pentacyclic structure, morphine and related natural products have been recognized as attractive targets of synthetic interest, and many groups have made impressive contributions to the literature on the syntheses of these molecules.^21–54^

**Scheme 4:**
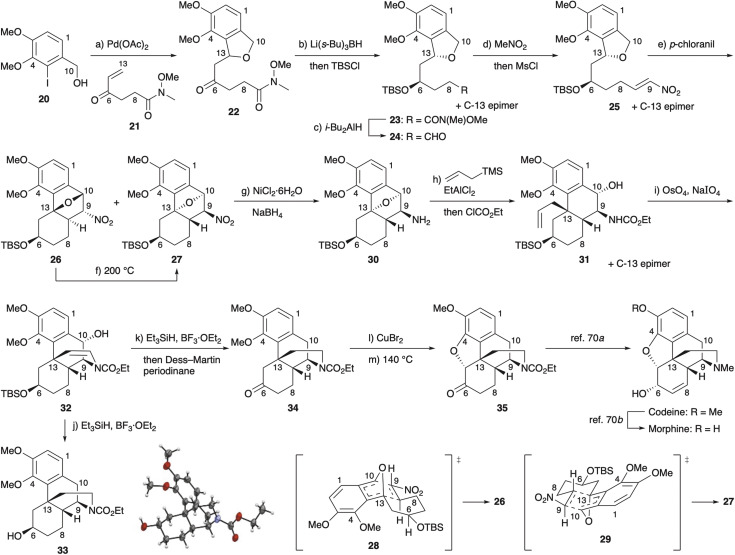
Formal synthesis of (±)-morphine. Reagents and conditions: (a) enone 21 (1.2 equiv.), Pd(OAc)_2_ (10 mol%), (*o*-MeC_6_H_4_)_3_P (40 mol%), Ag_3_PO_4_ (10 mol%), Et_3_N (3.1 equiv.), DMF, 110 °C, 25 h, 82%; (b) Li(*s*-Bu)_3_BH (2 equiv.), THF, −78 °C, 1 h, then TBSCl (7 equiv.), imidazole (11 equiv.), DMF, 9 h, 85%, dr = 15 : 1; (c) *i*-Bu_2_AlH (1.6 equiv.), CH_2_Cl_2_, −78 °C, 16 h, 80%; (d) CH_3_NO_2_ (102 equiv.), tetramethylguanidine (0.1 equiv.), toluene, 0 °C, 1 h, then MsCl (15 equiv.), Et_3_N (15 equiv.), 1 h, 86%; (e) *p*-chloranil (1.5 equiv.), 3 Å MS, dodecane, 200 °C, 3 h, 81% (26 : 27 = 1 : 2.7); (f) BHT (0.1 equiv.), dodecane, 200 °C, 25 h, 98% (26 : 27 = 1 : 3.6); (g) NiCl_2_·6H_2_O (0.5 equiv.), NaBH_4_ (13 equiv.), MeOH, 3 h, 82%; (h) H_2_C=CHCH_2_TMS (1.7 equiv.), EtAlCl_2_ (3 equiv.), CH_2_Cl_2_, −45 °C, 6 h, then ClCO_2_Et (5 equiv.), 1 M aq. NaOH, 14 h, 31 38%, 13-*epi*-31 19%; (i) OsO_4_ (2 mol%), NaIO_4_ (4 equiv.), 2,6-lutidine (2 equiv.), 3 : 1 1,4-dioxane/H_2_O, 36 h, 77%; (j) Et_3_SiH (3 equiv.), BF_3_·OEt_2_ (2.5 equiv.), CH_2_Cl_2_, −78 to 0 °C, 2.5 h, 70%; (k) Et_3_SiH (3 equiv.), BF_3_·OEt_2_ (3 equiv.), CH_2_Cl_2_, −78 to 0 °C, 2 h, then Dess–Martin periodinane (2 equiv.), pyridine (2.5 equiv.), 1 h, 73%; (l) CuBr_2_ (2.2 equiv.), 1 : 1 CHCl_3_/AcOEt, 70 °C, 2 h; (m) DMF, 140 °C, 25.5 h, 42% (59% after two cycles). Ms = methanesulfonyl; TMS = trimethylsilyl.

The synthesis was initiated with the sequential Heck/oxa-Michael reaction between iodide 20 (ref. 55) and enone 21 (ref. 56) in the presence of Ag_3_PO_4_ (ref. 59) in *N*,*N*-dimethylformamide (DMF) at 110 °C, affording phthalan 22 in 82% yield (Scheme 4). The carbonyl group was reduced with Li(*s*-Bu)_3_BH in THF at −78 °C,^60,61^ and the resultant alkoxide was protonated and silylated *in situ*^62^ to give *tert*-butyldimethylsilyl (TBS) ether 23 as an inseparable 15 : 1 mixture of diastereomers in 85% yield. After reduction of the Weinreb amide with *i*-Bu_2_AlH in CH_2_Cl_2_ at −78 °C, one-pot homologation of aldehyde 24 to nitroalkene 25 under Merck conditions^63^ set the stage for the key oxidation/IMDA sequence.

We expected that stereoinduction would be observed when using phthalans with a stereocenter in the tether as substrates. As anticipated, a 1 : 2.7 mixture of cycloadducts 26 and 27 was obtained in 81% yield upon heating at 200 °C for 3 h. Transition state 29, in which the cyclohexane ring adopts a chair conformation, is favoured over the diastereomeric transition state 28, thus leading to the preferential formation of 27.^64^ At this juncture, we noticed that independent submission of cycloadducts 26 and 27 to BHT in dodecane at 200 °C provided identical ratios of isomers (26 : 27 = 1 : 3.6).^65^ While the reactions of IBFs were reported not to be reversible under the conditions normally employed,^1*b*,66^ our results clearly revealed that the cycloaddition was reversible. Although both stereoisomers 26 and 27 could be carried forward, it was more expedient to work with a homogeneous material. We then proceeded forward in the synthesis with major isomer 27.

Given characteristic oxygen-bridged products obtained by the present oxidation/IMDA reactions, regio- and stereoselective ring-opening methods need to be devised for the application to total synthesis. After considerable experimentation with regard to the ring-opening of 27, reaction of amine 30, obtained by reduction of 27 using NiCl_2_/NaBH_4_ in MeOH in 82% yield,^67^ with allyltrimethylsilane with the aid of EtAlCl_2_ in CH_2_Cl_2_ at −45 °C was found to fulfill this requirement, providing a 2 : 1 mixture of allylation products 31 and 13-*epi*-31 in 57% yield after *N*-protection with ClCO_2_Et.^68^ Oxidative cleavage of the olefin in allylation product 31 with OsO_4_/NaIO_4_ (ref. 69) was accompanied by cyclization to furnish enecarbamate 32 in 77% yield. The benzylic hydroxy group and double bond were successfully reduced upon treatment of enecarbamate 32 with Et_3_SiH in the presence of BF_3_·OEt_2_ in CH_2_Cl_2_ at −78 °C, and raising the temperature to 0 °C effected desilylation, affording crystalline alcohol 33, the stereochemistry of which was unambiguously established by X-ray crystallography.^70^ Enecarbamate 32 could be converted to ketone 34 by a one-pot procedure involving oxidation of alcohol 33 with Dess–Martin periodinane^57^ buffered with pyridine. Ketone 34 underwent α-bromination with CuBr_2_ in refluxing CHCl_3_/AcOEt,^46,71^ and heating the crude product in DMF at 140 °C effected intramolecular etherification to provide *N*-carbethoxynorcodeinone (35)^72*a*^ in 59% yield after one recycle.^73^ While the conversion of 35 to codeine^72*a*^ and its *O*-demethylation to morphine^72*b*^ were reported by the Rice group, the synthesis of 35 constitutes a formal synthesis of (±)-morphine.

## Conclusions

We have developed a novel oxidation/IMDA reaction sequence, wherein unstabilized IBFs were generated as transient species from phthalans upon oxidation with *p*-chloranil. Exclusive formation of endo cycloadducts was observed and the reaction proved to be reversible under these conditions. This metal-free protocol represents the first general method for oxidative generation of IBFs and the first example that provides experimental evidence for the reversibility of Diels–Alder reactions of IBFs without using maleic anhydride as a dienophile. The method presented herein offers the advantage of obtaining cycloadducts without aromatization in most cases, and the bridging oxygen can serve as a handle for the installation of substituents at the benzylic position. The successful application to the formal synthesis of (±)-morphine attests to the power of the present method in natural product synthesis.

## Data availability

The data supporting this article have been included as part of the ESI. Crystallographic data for compound 33 have been deposited at the Cambridge Crystallographic Data Center (CCDC) under CCDC 2237321.‡

## Author contributions

S. N. conceived the project and prepared the manuscript. S. N. and H. Y. designed the project and directed the investigations. M. K., H. Y., Ma. T., E. O., K. N., T. W., M. U., Mi. T. and S. N. performed the experiments. H. Y., Ma. T., E. O., K. N., T. W., K. I. and S. N. prepared the ESI. All of the authors discussed the results.

## Conflicts of interest

There are no conflicts to declare.

## Supplementary Material

SC-OLF-D4SC05890A-s001

SC-OLF-D4SC05890A-s002
