# Supplementary material for: Oxidative generation of isobenzofurans from phthalans: application to the formal synthesis of (±)-morphine
Source: Chem Sci. 2024 Oct 19;15(45):19070–6. doi: 10.1039/d4sc05890a (PMC11523798; doi:10.1039/d4sc05890a)

Supplementary Information (SI) for Chemical Science.
This journal is © The Royal Society of Chemistry 2024

Supplementary Information

Oxidative generation of isobenzofurans from phthalans: application to the formal synthesis of (±)-morphine

Mirai Kage, Hiroyuki Yamakoshi, Manami Tabata, Eisaku Ohashi, Kimihiro Noguchi, Takeshi Watanabe, Manato Uchida, Minetatsu Takada, Kazutada Ikeuchi and Seiichi Nakamura*

Graduate School of Pharmaceutical Sciences, Nagoya City University
3-1 Tanabe-dori, Mizuho-ku, Nagoya 467-8603, Japan

nakamura@phar.nagoya-cu.ac.jp

Table of Contents

1. General Information 2
2. Experimental Procedures 3
   2-1. Substrate Preparation for the Tandem Reaction 3
   2-2. Tandem Oxidation/Intramolecular Diels−Alder Reaction 33
   2-3. Formal Synthesis of (±)-Morphine 44
3. X-Ray Chromatographic Information of Alcohol **33** 57
4. Computational Analysis 58
5. Additional References 71
6. Copies of ^{1}H and ^{13}C NMR Spectra 72
7. NOESY Correlation Diagram for Cycloadduct **3**, and Copy of NOESY Spectrum 140

1. General Information

Melting points were determined on a Büchi M-565 digital melting point apparatus and were uncorrected. Infrared (IR) spectra were recorded on a Shimazdu IR Affinity-1 FTIR spectrophotometer and absorbance bands are reported in wavenumber (cm^{-1}). Proton nuclear magnetic resonance (^1H NMR) spectra were recorded on JEOL JNM-ECZ500R (500 MHz) or Varian VNMRS 500 (500 MHz) spectrometers with tetramethylsilane (δ_H 0.00), $CHCl_3$ (δ_H 7.26), C_6H_6 (δ_H 7.16), or pyridine-d_5 (δ_H 7.22) as an internal standard. Coupling constants (J) are reported in hertz (Hz). Abbreviations of multiplicity are as follows: s, singlet; d, doublet; t, triplet; q, quartet; m, multiplet; br, broad. Data are presented as follows: chemical shift, multiplicity, coupling constants, integration and assignment. Morphine numbering is used for proton assignments of morphine intermediates. Carbon nuclear magnetic resonance (^{13}C NMR) spectra were recorded on JEOL JNM-ECZ500R (126 MHz), Varian VNMRS 500 (126 MHz), or Bruker AVANCE III HD600 (150 MHz) spectrometers with $CDCl_3$ (δ_C 77.0) or C_6D_6 (δ_C 128.0) as an internal standard. High-resolution mass spectra (HRMS) were recorded by electrospray ionization (ESI) using a JEOL JMS-T100LP AccuTOF LC-plus.

Column chromatography was carried out on silica gel 60 N (63−210 μm or 40−50 μm). Analytical thin layer chromatography (TLC) was carried out with 0.25 mm silica gel plates. Visualization was accomplished with ultraviolet light and anisaldehyde stain, followed by heating.

Reagents and solvents were purified by standard means or used as received unless otherwise noted. Dehydrated dichloromethane (CH_2Cl_2) and tetrahydrofuran (THF, stabilizer free) were purchased. Molecular sieves were finely ground in mortar and heated in vacuo at 180 °C for 4 h. Bromoalcohol **S7**,[74] iodoalcohol **S21**,[75] enone **S22**,[76] bromide **S28**,[77] dimethyl (trimethylsilyl)methylphosphonate,[78] aldehyde **S32**,[79] Ohira−Bestmann reagent [$(MeO)_2P(O)C(N_2)COMe$],[80] Dess−Martin periodinane,[57] allyl alcohol **S37**,[58] and iodoalcohol **20**[55] were prepared according to literature procedures. All reactions were conducted under an argon atmosphere unless otherwise noted.

2. Experimental Procedures

2-1. Substrate Preparation for the Tandem Reaction

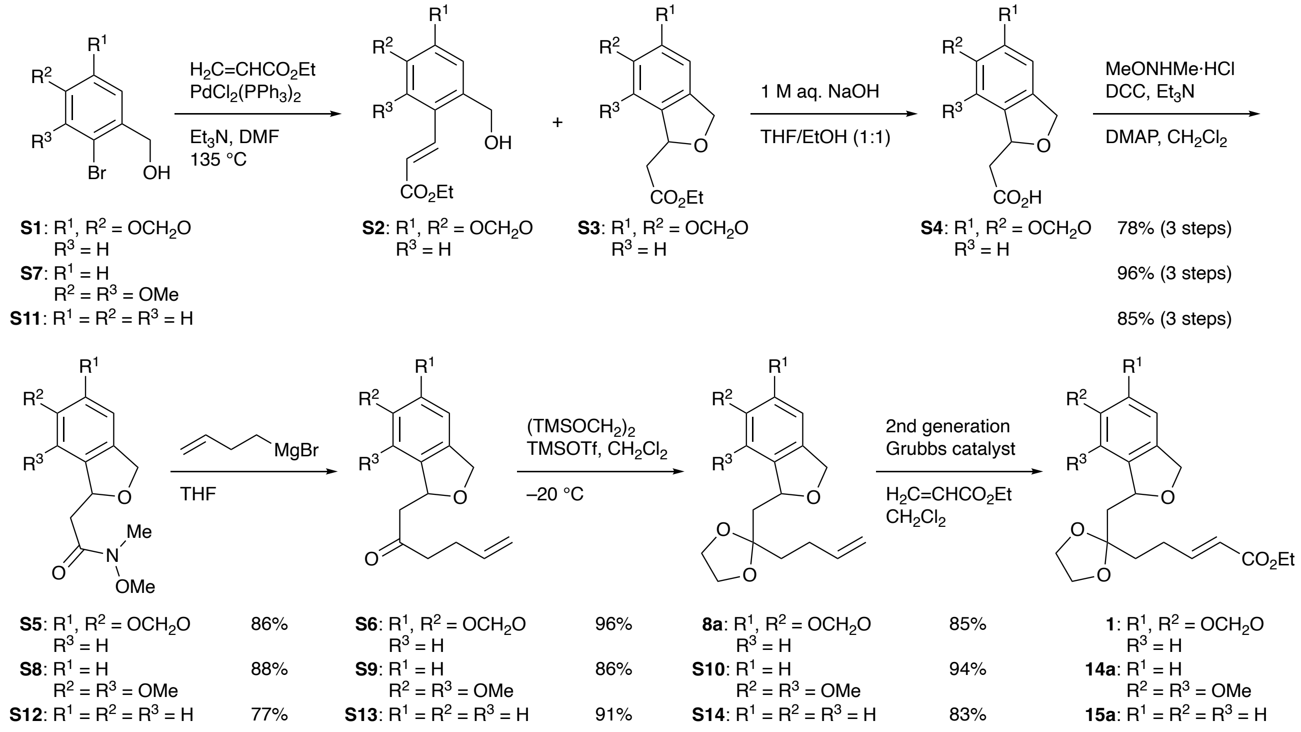

Typical Procedure for Preparation of Weinreb Amides from Bromoalcohols: *N*-Methoxy-*N*-methyl-(1,3-dihydro-5,6-methylenedioxyisobenzofuran-1-yl)ethanamide (S5). $PdCl_2(PPh_3)_2$ (91.9 mg, 0.131 mmol) was added to a mixture of bromoalcohol **S1** (3.03 g, 13.1 mmol), Et_3N (5.5 mL, 39.3 mmol) and ethyl acrylate (2.2 mL, 19.7 mmol) in DMF (26 mL), and the resulting solution was heated at 135 °C for 4 h. After cooling, the mixture was filtered through a Celite pad, and the filtrate was partitioned between *n*-hexane/AcOEt (1:4, 150 mL) and H_2O (50 mL). The aqueous layer was extracted with *n*-hexane/AcOEt (1:4, 2 × 150 mL), and the combined organic extracts were washed with brine (2 × 80 mL) and dried over anhydrous Na_2SO_4. Filtration and evaporation in vacuo furnished the crude mixture of esters **S2** and **S3** (4.64 g), which was used without further purification.

A 1 M aqueous NaOH solution (26 mL, 26.0 mmol) was added to an ice-cooled (0 °C) mixture of the crude esters **S2** and **S3** (4.64 g) in EtOH/THF (1:1, 26 mL). After 2 h of stirring at room temperature, the mixture was acidified to pH 1 with 2 M aqueous hydrochloric acid, and the resulting mixture was extracted with Et_2O (3 × 150 mL). The combined organic extracts were washed with brine (2 × 80 mL) and dried over anhydrous Na_2SO_4. Filtration and evaporation in vacuo furnished the crude carboxylic acid **S4** (2.75 g), which was used without further purification.

DCC (2.67 g, 13.1 mmol) was added to an ice-cooled (0 °C) mixture of the crude carboxylic acid **S4** (2.75 g), *N*,*O*-dimethylhydroxylamine hydrochloride (1.56 g, 16.0 mmol), Et_3N (2.2 mL, 15.7 mmol) and DMAP (162 mg, 1.31 mmol) in CH_2Cl_2 (65 mL). After 17 h of stirring at room temperature, the reaction was quenched with saturated aqueous NH_4Cl (80 mL), and the resulting mixture was filtered through a Celite pad. The filtrate was extracted with AcOEt (2 × 200 mL), and the combined organic extracts were washed with brine (2 × 80 mL) and dried over anhydrous Na_2SO_4. Filtration and evaporation in vacuo furnished the crude product (4.50 g), which was purified by column chromatography (silica gel 60 g, *n*-hexane/AcOEt 1:1) to give amide **S5** (2.72 g, 78% for three steps) as a white solid. R_f = 0.69 (AcOEt); mp 72.0−75.0 °C (colorless needles from *n*-hexane/ CH_2Cl_2 10:1); IR (KBr) ν_{max}/cm^{-1} 1659, 1494, 1475, 1391, 1368, 1339, 1275, 1055, 1040, 989, 939; δ_H (500 MHz; $CDCl_3$) 6.71 (1 H, s, Ar*H*), 6.65 (1 H, s, Ar*H*), 5.97 (2 H, s, OC*H*2O), 5.64 (1 H, dddd, *J* = 1.7, 2.9, 4.9, 7.6 Hz, ArC*H*CH2), 5.04 (1 H, dd, *J* = 2.9, 11.7 Hz, one of ArC*H*2O), 4.97 (1 H, dd, *J* = 1.7, 11.7 Hz, one of ArC*H*2O), 3.67 (3 H, s, OC*H*3), 3.23 (3 H, s, NC*H*3), 2.97 (1 H, dd, *J* = 7.6, 15.7 Hz, one of C*H*2CO), 2.73 (1 H, dd, *J* = 4.9, 15.7 Hz, one of C*H*2CO); δ_C (126 MHz; $CDCl_3$) 171.6 (C), 147.8 (C), 147.5 (C), 134.2 (C), 131.9 (C), 102.2 (CH), 101.5 (CH), 101.4 (CH_2), 80.3 (CH), 72.5 (CH_2), 61.2 (CH_3), 39.1 (CH_2), 32.0 (CH_3); HRMS (ESI) *m*/*z* $[M + Na]^+$ calcd for $C_{13}H_{15}NO_5Na$ 288.0842; found 288.0852; elemental analysis found: C, 58.64; H, 5.74; N, 5.40. $C_{13}H_{15}NO_5$ requires C, 58.86; H, 5.70; N, 5.28%.

***N*-Methoxy-*N*-methyl-(1,3-dihydro-4,5-dimethoxyisobenzofuran-3-yl)ethanamide (S8).** The conversion of bromoalcohol **S7**[74] (10.0 g, 40.5 mmol) to Weinreb amide **S8** was performed according to the typical procedure employing $PdCl_2(PPh_3)_2$ (433 mg, 0.617 mmol), Et_3N (17.0 mL, 122 mmol), ethyl acrylate (6.6 mL, 60.8 mmol), DMF (80 mL), 1 M aqueous NaOH (81 mL, 81.0 mmol), EtOH/THF (1:1, 80 mL), DCC (10.6 g, 51.4 mmol), *N*,*O*-dimethylhydroxylamine hydrochloride (5.96 g, 60.8 mmol), Et_3N (8.5 mL, 60.8 mmol), DMAP (745 mg, 6.08 mmol) and CH_2Cl_2 (200 mL). The crude product (16.2 g) was purified by column chromatography (silica gel 300 g, *n*-hexane/ AcOEt 1:1) to give amide **S8** (11.0 g, 96% for three steps) as a colorless oil. R_f = 0.63 (AcOEt); IR (neat) ν_{max}/cm^{-1} 2940, 2837, 1661, 1493, 1427, 1385, 1366, 1304, 1271, 1223, 1179, 1084, 1032, 995; δ_H (500 MHz; $CDCl_3$) 6.87 (2 H, s, Ar*H*), 5.87 (1 H, dddd, *J* = 1.1, 2.4, 2.5, 9.8 Hz, ArC*H*CH2),

5.09 (1 H, dd, *J* = 2.5, 11.6 Hz, one of ArCH_2O), 5.00 (1 H, dd, *J* = 1.1, 11.6 Hz, one of ArCH_2O), 3.87 (3 H, s, OCH_3), 3.86 (3 H, s, OCH_3), 3.68 (3 H, s, OCH_3), 3.23 (3 H, s, NCH_3), 3.09 (1 H, dd, *J* = 2.4, 15.7 Hz, one of CH_2CO), 2.89 (1 H, dd, *J* = 9.8, 15.7 Hz, one of CH_2CO); δ_C (126 MHz; $CDCl_3$) 171.8 (C), 151.7 (C), 143.5 (C), 134.4 (C), 132.9 (C), 116.0 (CH), 113.0 (CH), 79.3 (CH), 72.3 (CH_2), 61.2 (CH_3), 60.5 (CH_3), 56.3 (CH_3), 37.5 (CH_2), 32.1 (CH_3); HRMS (ESI) *m*/*z* $[M + Na]^+$ calcd for $C_{14}H_{19}NO_5Na$ 304.1150; found 304.1164.

***N*-Methoxy-*N*-methyl-(1,3-dihydroisobenzofuran-1-yl)ethanamide (S12).** The conversion of bromoalcohol **S11** (3.02 g, 16.0 mmol) to Weinreb amide **S12** was performed according to the typical procedure employing $PdCl_2(PPh_3)_2$ (168 mg, 0.239 mmol), Et_3N (6.8 mL, 48.0 mmol), ethyl acrylate (2.6 mL, 24.0 mmol), DMF (32 mL), 1 M aqueous NaOH (32 mL, 32.0 mmol), EtOH/THF (1:1, 32 mL), DCC (3.47 g, 16.0 mmol), *N*,*O*-dimethylhydroxylamine hydrochloride (1.60 g, 16.3 mmol), Et_3N (2.2 mL, 16.0 mmol), DMAP (2.06 g, 16.9 mmol) and CH_2Cl_2 (80 mL). The crude product (4.11 g) was purified by column chromatography (silica gel 25 g, *n*-hexane/AcOEt 3:2 → 1:1) to give amide **S12** (3.01 g, 85% for three steps) as a colorless oil. R_f = 0.42 (*n*-hexane/AcOEt 1:2); IR (neat) ν_{max}/cm^{-1} 2938, 2903, 2855, 1659, 1462, 1420, 1387, 1179, 1109, 1034, 955; δ_H (500 MHz; $CDCl_3$) 7.31−7.22 (4 H, m, Ar*H*), 5.76 (1 H, ddd, *J* = 2.5, 4.6, 8.3 Hz, Ar$CH$$CH_2$), 5.15 (1 H, dd, *J* = 2.5, 12.3 Hz, one of ArCH_2O), 5.08 (1 H, d, *J* = 12.3 Hz, one of ArCH_2O), 3.67 (3 H, s, OCH_3), 3.24 (3 H, s, NCH_3), 3.02 (1 H, dd, *J* = 8.3, 15.6 Hz, one of CH_2CO), 2.80 (1 H, dd, *J* = 4.6, 15.6 Hz, one of CH_2CO); δ_C (126 MHz; $CDCl_3$) 171.5 (C), 141.4 (C), 139.2 (C), 127.7 (CH), 127.4 (CH), 121.4 (CH), 121.0 (CH), 80.3 (CH), 72.5 (CH_2), 61.3 (CH_3), 39.0 (CH_2), 32.0 (CH_3); HRMS (ESI) *m*/*z* $[M + Na]^+$ calcd for $C_{12}H_{15}NO_3Na$ 244.0944; found 244.0954.

Typical Procedure for Preparation of Ketones from Weinreb Amides: 1-(1,3-Dihydro-5,6-methylenedioxyisobenzofuran-1-yl)hex-5-en-2-one (S6). 4-Bromo-1-butene (1.2 mL, 11.3 mmol) was added to a suspension of magnesium (420 mg, 17.3 mmol) in THF (8 mL). After 1 h of stirring, the reaction mixture was cooled to 0 °C, and a solution of amide **S5** (2.00 g, 7.54 mmol) in THF (10 mL plus 2 × 2.5 mL rinse) was added. After 1 h of stirring at room temperature, the reaction was quenched with 1 M aqueous hydrochloric acid (15 mL), and the resulting mixture was partitioned

between AcOEt (120 mL) and H_2O (15 mL). The aqueous layer was extracted with AcOEt (120 mL), and the combined organic extracts were washed with brine (2 × 60 mL) and dried over anhydrous Na_2SO_4. Filtration and evaporation in vacuo furnished the crude product (2.12 g), which was purified by column chromatography (silica gel 60 g, *n*-hexane/AcOEt 8:1) to give ketone **S6** (1.68 g, 86%) as a white solid. R_f = 0.49 (*n*-hexane/AcOEt 4:1); mp 63.0−64.0 °C (colorless needles from *n*-hexane/Et_2O 10:1); IR (KBr) ν_{max}/cm^{-1} 2899, 2857, 1705, 1501, 1479, 1381, 1342, 1267, 1103, 1076, 1038, 941, 914, 866; δ_H (500 MHz; $CDCl_3$) 6.64 (1 H, s, Ar*H*), 6.61 (1 H, s, Ar*H*), 5.97 (2 H, s, OC*H*2O), 5.82 (1 H, ddt, *J* = 10.3, 17.0, 6.8 Hz, CH_2=C*H*), 5.57 (1 H, dddd, *J* = 1.7, 3.0, 5.0, 7.6 Hz, ArC*H*CH_2CO), 5.04 (1 H, dd, *J* = 3.0, 11.7 Hz, one of ArC*H*2O), 5.01 (1 H, dq, *J* = 17.0, 1.6 Hz, one of CH=C*H*2), 4.98 (1 H, dq, *J* = 10.3, 1.6 Hz, one of CH=C*H*2), 4.95 (1 H, dd, *J* = 1.7, 11.7 Hz, one of ArC*H*2O), 2.87 (1 H, dd, *J* = 7.6, 16.0 Hz, one of ArCHC*H*2CO), 2.76 (1 H, dd, *J* = 5.0, 16.0 Hz, one of ArCHC*H*2CO), 2.62 (1 H, dt, *J* = 17.3, 7.4 Hz, one of CH_2=CHCH_2C*H*2), 2.56 (1 H, dt, *J* = 17.3, 7.4 Hz, one of CH_2=CHCH_2C*H*2), 2.35 (2 H, dtt, *J* = 6.8, 1.6, 7.4 Hz, CH_2=CHC*H*2); δ_C (126 MHz; $CDCl_3$) 207.9 (C), 147.9 (C), 147.6 (C), 137.0 (CH), 134.0 (C), 131.8 (C), 115.3 (CH_2), 101.9 (CH), 101.6 (CH), 101.5 (CH_2), 80.0 (CH), 72.6 (CH_2), 49.6 (CH_2), 42.9 (CH_2), 27.5 (CH_2); HRMS (ESI) *m*/*z* $[M + Na]^+$ calcd for $C_{15}H_{16}O_4Na$ 283.0941; found 283.0939; elemental analysis found: C, 68.93; H, 6.24. $C_{15}H_{16}O_4$ requires C, 69.22; H, 6.20%.

1-(1,3-Dihydro-4,5-dimethoxyisobenzofuran-3-yl)hex-5-en-2-one (S9). The conversion of amide **S8** (2.50 g, 8.88 mmol) to ketone **S9** was performed according to the typical procedure (32 mL THF, rt, 30 min) employing 4-bromo-1-butene (1.4 mL, 13.3 mmol) and magnesium (433 mg, 17.8 mmol). The crude product (2.65 g) was purified by column chromatography (silica gel 60 g, *n*-hexane/AcOEt 2:1) to give ketone **S9** (2.15 g, 88%) as a colorless oil. R_f = 0.61 (*n*-hexane/AcOEt 2:1); IR (neat) ν_{max}/cm^{-1} 3076, 2940, 2837, 1715, 1641, 1489, 1366, 1269, 1223, 1080, 1036, 916; δ_H (500 MHz; $CDCl_3$) 6.86 (2 H, s, Ar*H*), 5.82 (1 H, ddt, *J* = 10.3, 17.1, 6.5 Hz, C*H*=CH_2), 5.78 (1 H, dddd, *J* = 1.4, 2.5, 3.3, 8.8 Hz, ArC*H*CH_2), 5.06 (1 H, dd, *J* = 2.5, 11.7 Hz, one of ArC*H*2O), 5.04 (1 H, dq, *J* = 17.1, 1.7 Hz, one of CH=C*H*2), 4.98 (1 H, dq, *J* = 10.3, 1.7 Hz, one of CH=C*H*2), 4.97 (1 H, dd, *J* = 1.4, 11.7 Hz, one of ArC*H*2O), 3.86 (3 H, s, OC*H*3), 3.83 (3 H, s, OC*H*3), 3.08 (1 H, dd, *J* = 3.3, 16.0 Hz, one of ArCHC*H*2), 2.79 (1 H, dd, *J* = 8.8, 16.0 Hz, one of ArCHC*H*2), 2.62 (1 H, dt, *J*

= 17.5, 7.6 Hz, one of C*H*$_2$CH$_2$CH=CH$_2$), 2.58 (1 H, dt, J = 17.5, 7.1 Hz, one of C*H*$_2$CH$_2$CH=CH$_2$), 2.36 (2 H, dddt, *J* = 6.5, 7.1, 7.6, 1.7 Hz, CH$_2$C*H*$_2$CH=CH$_2$); δ_C (126 MHz; CDCl$_3$) 207.8 (C), 151.7 (C), 143.3 (C), 137.2 (CH), 134.1 (C), 132.7 (C), 116.0 (CH), 115.1 (CH$_2$), 113.0 (CH), 79.0 (CH), 72.4 (CH$_2$), 60.5 (CH$_3$), 56.3 (CH$_3$), 48.0 (CH$_2$), 42.7 (CH$_2$), 27.5 (CH$_2$); HRMS (ESI) *m/z* [M + Na]$^+$ calcd for $C_{16}H_{20}O_4Na$ 299.1254; found 299.1246.

(1,3-Dihydroisobenzofuran-1-yl)hex-5-en-2-one (S13). The conversion of amide **S12** (1.99 g, 9.04 mmol) to ketone **S13** was performed according to the typical procedure (24 mL THF, rt, 30 min) employing 4-bromo-1-butene (1.4 mL, 13.3 mmol) and magnesium (458 mg, 18.1 mmol). The crude product (1.30 g) was purified by column chromatography (silica gel 50 g, *n*-hexane/AcOEt 4:1) to give ketone **S13** (1.51 g, 77%) as a colorless oil. R_f = 0.43 (*n*-hexane/AcOEt 4:1); IR (neat) ν_{max}/cm^{-1} 3076, 2905, 1713, 1641, 1479, 1462, 1406, 1366, 1317, 1260, 1107, 1039, 1001, 914; δ_H (500 MHz; CDCl$_3$) 7.30−7.22 (3 H, m, Ar*H*), 7.17 (1 H, m, Ar*H*), 5.82 (1 H, ddt, *J* = 10.3, 16.9, 6.7 Hz, C*H*=CH$_2$), 5.68 (1 H, ddd, *J* = 2.4, 4.7, 8.0 Hz, ArC*H*CH$_2$), 5.13 (1 H, dd, *J* = 2.4, 12.2 Hz, one of ArC*H*$_2$O), 5.06 (1 H, d, *J* = 12.2 Hz, one of ArC*H*$_2$O), 5.04 (1 H, dq, *J* = 16.9, 1.7 Hz, one of CH=C*H*$_2$), 4.99 (1 H, dq, *J* = 10.3, 1.7 Hz, one of CH=C*H*$_2$), 2.91 (1 H, dd, *J* = 8.0, 16.1 Hz, one of ArCHOC*H*$_2$), 2.83 (1 H, dd, *J* = 4.7, 16.1 Hz, one of ArCHC*H*$_2$), 2.63 (1 H, dt, *J* = 17.3, 7.6 Hz, one of C*H*$_2$CH$_2$CH=CH$_2$), 2.58 (1 H, dt, *J* = 17.3, 7.6 Hz, one of C*H*$_2$CH$_2$CH=CH$_2$), 2.37 (2 H, dtt, *J* = 6.7, 1.7, 7.6 Hz, CH$_2$C*H*$_2$CH=CH$_2$); δ_C (126 MHz; CDCl$_3$) 207.9 (C), 141.1 (C), 139.1 (C), 137.0 (CH), 127.7 (CH), 127.4 (CH), 121.2 (CH), 121.0 (CH), 115.3 (CH$_2$), 80.0 (CH), 72.6 (CH$_2$), 49.5 (CH$_2$), 42.8 (CH$_2$), 27.5 (CH$_2$); HRMS (ESI) *m/z* [M + Na]$^+$ calcd for $C_{14}H_{16}O_2Na$ 239.1043; found 239.1044.

Typical Procedure for Acetalization: 1-[2-(But-3-enyl)-1,3-dioxolan-2-yl]methyl-1,3-dihydro-5, 6-methylenedioxyisobenzofuran (8a). A solution of ketone **S6** (1.20 g, 4.61 mmol) in CH$_2$Cl$_2$ (5 mL) was added to a cooled (−78 °C) mixture of TMSOTf (30 μL, 0.163 mmol) and 1,2-bis(trimethylsiloxy)ethane (1.4 mL, 5.53 mmol) in CH$_2$Cl$_2$ (4 mL). After 9 h of stirring at −20 °C, the reaction was quenched with pyridine (0.1 mL), and the resulting mixture was partitioned between AcOEt (80 mL) and saturated aqueous NaHCO$_3$ (20 mL). The organic layer was washed with brine (20 mL)

and dried over anhydrous Na_2SO_4. Filtration and evaporation in vacuo furnished the crude product (1.93 g), which was purified by column chromatography (silica gel 50 g, *n*-hexane/AcOEt 5:1) to give acetal **8a** (1.35 g, 96%) as a pale yellow oil. R_f = 0.63 (toluene/AcOEt 6:1); IR (neat) ν_{max}/cm^{-1} 2974, 2886, 1477, 1267, 1152, 1130, 1038, 935; δ_H (500 MHz; $CDCl_3$) 6.65 (1 H, s, Ar*H*), 6.63 (1 H, s, Ar*H*), 5.96 (2 H, s, OC*H*$_2$O), 5.85 (1 H, ddt, *J* = 10.3, 17.0, 6.6 Hz, C*H*=CH_2), 5.28 (1 H, dddd, *J* = 1.8, 2.8, 3.3, 8.0 Hz, ArC*H*CH_2), 5.04 (1 H, dq, *J* = 17.0, 1.7 Hz, one of CH=C*H*$_2$), 5.02 (1 H, dd, *J* = 2.8, 11.2 Hz, one of ArC*H*$_2$O), 4.94 (1 H, dq, *J* = 10.3, 1.7 Hz, one of CH=C*H*$_2$), 4.92 (1 H, dd, *J* = 1.8, 11.2 Hz, one of ArC*H*$_2$O), 4.09−3.98 (4 H, m, OC*H*$_2$C*H*$_2$O), 2.26−2.12 (2 H, m, CH_2=CHC*H*$_2$), 2.08 (1 H, dd, *J* = 8.0, 15.0 Hz, one of ArCHC*H*$_2$), 2.03 (1 H, dd, *J* = 3.3, 15.0 Hz, one of ArCHC*H*$_2$), 1.87 (2 H, t, *J* = 8.0 Hz, CH_2=CHCH_2C*H*$_2$); δ_C (126 MHz; $CDCl_3$) 147.54 (C), 147.46 (C), 138.6 (CH), 135.5 (C), 131.9 (C), 114.3 (C), 110.4 (CH_2), 102.0 (CH_2), 101.44 (CH), 101.38 (CH), 80.0 (CH), 72.5 (CH_2), 65.02 (CH_2), 64.97 (CH_2), 44.1 (CH_2), 37.1 (CH_2), 28.1 (CH_2); HRMS (ESI) *m*/*z* $[M + Na]^+$ calcd for $C_{17}H_{20}O_5Na$ 327.1203; found 327.1199.

3-[2-(But-3-enyl)-1,3-dioxolan-2-yl]methyl-1,3-dihydro-4,5-dimethoxyisobenzofuran (S10). The acetalization was performed according to the typical procedure (3 mL CH_2Cl_2, −20 °C, 24 h) employing ketone **S9** (371 mg, 1.34 mmol), TMSOTf (10 μL, 54.3 μmol), and 1,2-bis(trimethylsiloxy)ethane (0.4 mL, 1.61 mmol). The crude product (512 mg) was purified by column chromatography (silica gel 15 g, *n*-hexane/AcOEt 6:1) to give acetal **S10** (371 mg, 86%) as a colorless oil. R_f = 0.40 (2:1 *n*-hexane/AcOEt); IR (neat) ν_{max}/cm^{-1} 2938, 2884, 1639, 1491, 1427, 1304, 1269, 1150, 1069, 1032, 910; δ_H (500 MHz; $CDCl_3$) 6.84 (1 H, d, *J* = 8.4 Hz, Ar*H*), 6.82 (1 H, d, *J* = 8.4 Hz, Ar*H*), 5.87 (1 H, ddt, *J* = 10.2, 17.0, 6.6 Hz, C*H*=CH_2), 5.52 (1 H, dddd, *J* = 1.0, 1.3, 2.5, 9.5 Hz, ArC*H*CH_2), 5.07 (1 H, dd, *J* = 2.5, 11.8 Hz, one of ArC*H*$_2$O), 5.03 (1 H, dq, *J* = 17.0, 1.8 Hz, one of CH=C*H*$_2$), 4.96 (1 H, dd, *J* = 1.0, 11.8 Hz, one of ArC*H*$_2$O), 4.93 (1 H, dq, *J* = 10.2, 1.8 Hz, one of CH=C*H*$_2$), 4.08−3.96 (4 H, m, OC*H*$_2$C*H*$_2$O), 3.86 (3 H, s, OC*H*$_3$), 3.85 (3 H, s, OC*H*$_3$), 2.36 (1 H, dd, *J* = 1.3, 14.9 Hz, one of ArCHC*H*$_2$), 2.27−2.15 (2 H, m, CH_2C*H*$_2$CH=CH_2), 1.98−1.88 (3 H, m, C*H*$_2$$CH_2$CH=$CH_2$, one of ArCHC*H*$_2$); δ_C (126 MHz; $CDCl_3$) 151.6 (C), 143.4 (C), 138.8 (CH), 135.4 (C), 132.7 (C), 115.7 (CH), 114.1 (CH_2), 112.7 (CH), 110.6 (C), 79.4 (CH), 72.1 (CH_2), 64.9 (CH_2), 64.7 (CH_2), 60.3 (CH_3), 56.3 (CH_3), 41.4 (CH_2), 36.8 (CH_2), 28.0 (CH_2); HRMS (ESI) *m*/*z* [M +

Na]$^+$ calcd for $C_{18}H_{24}O_5Na$ 343.1516; found 343.1503.

1-[2-(But-3-enyl)-1,3-dioxolan-2-yl]methyl-1,3-dihydroisobenzofuran (S14). The acetalization was performed according to the typical procedure (7 mL CH_2Cl_2, −20 °C, 20 h) employing ketone **S13** (1.01 g, 4.62 mmol), TMSOTf (25 μL, 136 μmol), and 1,2-bis(trimethylsiloxy)ethane (1.5 mL, 6.01 mmol). The crude product (1.45 g) was purified by column chromatography (silica gel 25 g, *n*-hexane/AcOEt 4:1) to give acetal **S14** (1.10 g, 91%) as a white solid. R_f = 0.43 (*n*-hexane/AcOEt 4:1); mp 38.0−39.0 °C (colorless needles from *n*-hexane/Et_2O 10:1); IR (KBr) ν_{max}/cm^{-1} 2889, 1643, 1477, 1306, 1150, 1051, 1022, 1009, 951; δ_H (500 MHz; $CDCl_3$) 7.28−7.24 (2 H, m, Ar*H*), 7.22−7.18 (2 H, m, Ar*H*), 5.86 (1 H, ddt, *J* = 10.3, 17.0, 6.6 Hz, C*H*=CH$_2$), 5.38 (1 H, dd, *J* = 2.2, 2.3 Hz, ArC*H*CH$_2$), 5.14 (1 H, dd, *J* = 2.3, 12.2 Hz, one of ArC*H*$_2$O), 5.04 (1 H, dq, *J* = 17.0, 1.3 Hz, one of CH=C*H*$_2$), 5.03 (1 H, d, *J* = 12.2 Hz, one of ArC*H*$_2$O), 4.95 (1 H, dq, *J* = 10.3, 1.3 Hz, one of CH=C*H*$_2$), 4.10−4.00 (4 H, m, OC*H*$_2$C*H*$_2$O), 2.27−2.14 (2 H, m, CH$_2$C*H*$_2$CH=CH$_2$), 2.13 (1 H, d, *J* = 16.0 Hz, one of ArCHC*H*$_2$), 2.09 (1 H, dd, *J* = 2.2, 16.0 Hz, one of ArCHC*H*$_2$), 1.91 (2 H, t, *J* = 8.4 Hz, C*H*$_2$CH$_2$CH=CH$_2$); δ_C (126 MHz; $CDCl_3$) 142.6 (C), 139.3 (C), 138.6 (CH), 127.4 (CH), 127.2 (CH), 121.2 (CH), 120.8 (CH), 114.2 (CH_2), 110.4 (C), 80.0 (CH), 72.5 (CH_2), 65.04 (CH_2), 64.98 (CH_2), 44.0 (CH_2), 37.1 (CH_2), 28.1 (CH_2); HRMS (ESI) *m/z* [M + Na]$^+$ calcd for $C_{16}H_{20}O_3Na$ 283.1305; found 283.1314.

Typical Procedure for Cross Metathesis: Ethyl (2*E*)-5-[2-(1,3-Dihydro-5,6-methylenedioxyiso-benzofuran-1-yl)methyl-1,3-dioxoran-2-yl]pent-2-enoate (1). The second-generation Grubbs catalyst (24.2 mg, 28.5 μmol) was added to a mixture of alkene **8a** (173 mg, 0.570 mmol) and ethyl acrylate (0.62 mL, 5.70 mmol) in CH_2Cl_2 (3 mL). After 43 h of stirring, the volatile elements were removed in vacuo, and the residue (269 mg) was purified by column chromatography (silica gel 15 g, *n*-hexane/AcOEt 4:1) to give enoate **1** (182 mg, 85%) as a white solid. R_f = 0.44 (*n*-hexane/AcOEt 2:1); mp 90.0−91.0 °C (colorless needles from *n*-hexane/CH_2Cl_2 10:1); IR (KBr) ν_{max}/cm^{-1} 2982, 2914, 2893, 1717, 1653, 1504, 1477, 1319, 1288, 1263, 1211, 1163, 1109, 1038, 931; δ_H (500 MHz; $CDCl_3$) 7.00 (1 H, dt, *J* = 15.6, 6.9 Hz, C*H*=CHCO$_2$Et), 6.63 (1 H, s, Ar*H*), 6.62 (1 H, s, Ar*H*), 5.96 (2 H, s, OC*H*$_2$O), 5.83 (1 H, dt, *J* = 15.6, 1.5 Hz, CH=C*H*CO$_2$Et), 5.26 (1 H, dddd, *J* = 1.7, 2.8, 3.4,

7.8 Hz, ArC*H*CH$_2$), 5.01 (1 H, dd, *J* = 2.8, 11.8 Hz, one of ArC*H*$_2$O), 4.92 (1 H, dd, *J* = 1.7, 11.8 Hz, one of ArC*H*$_2$O), 4.18 (2 H, q, *J* = 7.1 Hz, OC*H*$_2$CH$_3$), 4.08−3.98 (4 H, m, OC*H*$_2$C*H*$_2$O), 2.40−2.27 (2 H, m, CH=CHC*H*$_2$), 2.04 (1 H, dd, *J* = 7.8, 14.8 Hz, one of ArCHC*H*$_2$), 1.99 (1 H, dd, *J* = 3.4, 14.8 Hz, one of ArCHC*H*$_2$), 1.95 (2 H, t, *J* = 8.0 Hz, CH$_2$=CHCH$_2$C*H*$_2$), 1.28 (3 H, t, *J* = 7.1 Hz, OCH$_2$C*H*$_3$); δ_C (126 MHz; CDCl$_3$) 166.7 (C), 149.0 (CH), 147.6 (C), 147.5 (C), 135.2 (C), 131.9 (C), 121.1 (CH), 110.1 (C), 101.9 (CH), 101.5 (CH), 101.4 (CH$_2$), 80.0 (CH), 72.5 (CH$_2$), 65.03 (CH$_2$), 65.01 (CH$_2$), 60.1 (CH$_2$), 44.1 (CH$_2$), 36.0 (CH$_2$), 26.6 (CH$_2$), 14.3 (CH$_3$); HRMS (ESI) *m/z* [M + Na]$^+$ calcd for $C_{20}H_{24}O_7Na$ 399.1414; found 399.1425; elemental analysis found: C, 63.78; H, 6.55. $C_{20}H_{24}O_7$ requires C, 63.82; H, 6.43%.

Ethyl (2*E*)-5-[2-(1,3-Dihydro-4,5-dimethoxyisobenzofuran-3-yl)methyl-1,3-dioxolan-2-yl]pent-2-enoate (14a). The cross metathesis was performed according to the typical procedure (9.5 mL CH$_2$Cl$_2$, rt, 22 h) employing alkene **S10** (304 mg, 0.950 mmol), ethyl acrylate (1.1 mL, 9.50 mmol), and the second-generation Grubbs catalyst (40.8 mg, 47.5 μmol). The crude product (718 mg) was purified by column chromatography (silica gel 20 g, *n*-hexane/AcOEt 3:1) to give cross metathesis product **14a** (349 mg, 94%) as a colorless oil. R_f = 0.32 (*n*-hexane/AcOEt 2:1); IR (neat) ν_{max}/cm^{-1} 2955, 2934, 1717, 1653, 1489, 1456, 1368, 1267, 1159, 1041; δ_H (500 MHz; CDCl$_3$) 7.02 (1 H, dt, *J* = 15.7, 6.9 Hz, C*H*=CHCO$_2$Et), 6.86 (1 H, d, *J* = 8.4 Hz, Ar*H*), 6.84 (1 H, d, *J* = 8.4 Hz, Ar*H*), 5.84 (1 H, dt, *J* = 15.7, 1.6 Hz, CH=C*H*CO$_2$Et), 5.50 (1 H, ddd, *J* = 0.8, 2.2, 9.5 Hz, ArC*H*CH$_2$), 5.07 (1 H, dd, *J* = 2.2, 11.7 Hz, one of ArC*H*$_2$O), 4.96 (1 H, d, *J* = 11.7 Hz, one of ArC*H*$_2$O), 4.18 (2 H, q, *J* = 7.2 Hz, OC*H*$_2$CH$_3$), 4.07−3.97 (4 H, m, OC*H*$_2$C*H*$_2$O), 3.86 (6 H, s, 2 × OC*H*$_3$), 2.36 (1 H, dd, *J* = 0.8, 14.9 Hz, one of ArCHC*H*$_2$), 2.43−2.30 (2 H, m, CH$_2$C*H*$_2$CH=CH), 2.05 (1 H, ddd, *J* = 5.9, 10.1, 14.1 Hz, one of C*H*$_2$CH$_2$CH=CH), 1.98 (1 H, ddd, *J* = 6.1, 9.8, 14.1 Hz, one of C*H*$_2$CH$_2$CH=CH), 1.90 (1 H, dd, *J* = 9.5, 14.9 Hz, one of ArCHC*H*$_2$), 1.28 (3 H, t, *J* = 7.2 Hz, OCH$_2$C*H*$_3$); δ_C (126 MHz; CDCl$_3$) 166.8 (C), 151.7 (C), 149.3 (CH), 143.4 (C), 135.3 (C), 132.7 (C), 121.0 (CH), 115.8 (CH), 112.8 (CH), 110.3 (C), 79.4 (CH), 72.2 (CH$_2$), 64.9 (CH$_2$), 64.8 (CH$_2$), 60.4 (CH$_3$), 60.1 (CH$_2$), 56.3 (CH$_3$), 41.5 (CH$_2$), 35.8 (CH$_2$), 26.5 (CH$_2$), 14.3 (CH$_3$); HRMS (ESI) *m/z* [M + Na]$^+$ calcd for $C_{21}H_{28}O_7Na$ 415.1727; found 415.1744.

Ethyl (2*E*)-5-[2-(1,3-Dihydroisobenzofuran-1-yl)methyl-1,3-dioxolan-2-yl]pent-2-enoate (15a). The cross metathesis was performed according to the typical procedure (10 mL CH_2Cl_2, rt, 16 h) employing alkene **S14** (400 mg, 1.54 mmol), ethyl acrylate (1.7 mL, 15.4 mmol), and the second-generation Grubbs catalyst (66.6 mg, 78.4 μmol). The crude product (1.77 g) was purified by column chromatography (silica gel 20 g, *n*-hexane/AcOEt 6:1) to give cross metathesis product **15a** (455 mg, 83%) as a colorless oil. R_f = 0.39 (*n*-hexane/AcOEt 2:1); IR (neat) ν_{max}/cm^{-1} 2884, 1717, 1653, 1368, 1265, 1179, 1150, 1042, 949; δ_H (500 MHz; $CDCl_3$) 7.28−7.25 (2 H, m, Ar*H*), 7.22 (1 H, m, Ar*H*), 7.17 (1 H, m, Ar*H*), 7.01 (1 H, dt, *J* = 15.8, 6.8 Hz, C*H*=$CHCO_2Et$), 5.84 (1 H, dt, *J* = 15.8, 1.6 Hz, CH=C*H*CO_2Et), 5.37 (1 H, dddd, *J* = 1.2, 2.4, 3.6, 7.7 Hz, ArC*H*CH_2), 5.13 (1 H, dd, *J* = 2.4, 12.3 Hz, one of ArCH_2O), 5.03 (1 H, dd, *J* = 1.2, 12.3 Hz, one of ArCH_2O), 4.18 (2 H, q, *J* = 7.2 Hz, OC$H_2$$CH_3$), 4.09−3.98 (4 H, m, OC$H_2CH_2$O), 2.43−2.28 (2 H, m, CH_2CH_2CH=CH), 2.10 (1 H, dd, *J* = 3.6, 14.8 Hz, one of ArCHCH_2), 2.07 (1 H, dd, *J* = 7.7, 14.8 Hz, one of ArCHCH_2), 1.99 (2 H, t, *J* = 8.1 Hz, C$H_2$$CH_2$CH=CH), 1.28 (3 H, t, *J* = 7.2 Hz, OCH_2CH_3); δ_C (126 MHz; $CDCl_3$) 166.7 (C), 149.0 (CH), 142.4 (C), 139.2 (C), 127.4 (CH), 127.3 (CH), 121.13 (CH), 121.12 (CH), 120.9 (CH), 110.1 (C), 80.0 (CH), 72.5 (CH_2), 65.0 (2 × CH_2), 60.1 (CH_2), 44.0 (CH_2), 36.1 (CH_2), 26.6 (CH_2), 14.3 (CH_3); HRMS (ESI) *m*/*z* $[M + Na]^+$ calcd for $C_{19}H_{24}O_5Na$ 355.1516; found 355.1531.

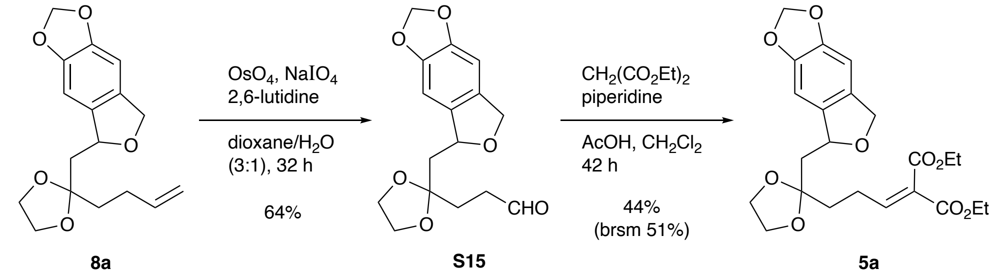

3-[2-(1,3-Dihydro-5,6-methylenedioxyisobenzofuran-1-yl)methyl-1,3-dioxolan-2-yl]propanal (S15). A 0.157 M solution of OsO_4 in *t*-BuOH (0.23 mL, 35.0 μmol) was added to a mixture of alkene **8a** (532 mg, 1.75 mmol), 2,6-lutidine (0.41 mL, 3.50 mmol) and $NaIO_4$ (1.50 g, 7.01 mmol) in dioxane/H_2O (3:1, 20 mL). After 32 h of stirring, the reaction was quenched with 1 M aqueous $Na_2S_2O_3$ (20 mL), and the resulting mixture was extracted with AcOEt (2 × 70 mL). The combined organic extracts were washed with brine (2 × 30 mL) and dried over anhydrous Na_2SO_4. Filtration and evaporation in vacuo furnished the crude product (772 mg), which was purified by column chromatography (silica gel 20 g, *n*-hexane/AcOEt 2:1) to give aldehyde **S15** (344 mg, 64%) as a

white solid. R_f = 0.54 (*n*-hexane/AcOEt 1:1); mp 70.0−71.0 °C (colorless needles from *n*-hexane/Et_2O 5:1); IR (KBr) ν_{max}/cm^{-1} 2897, 1715, 1499, 1477, 1340, 1287, 1273, 1152, 1034, 961, 935; δ_H (500 MHz; $CDCl_3$) 9.73 (1 H, t, *J* = 2.1 Hz, C*H*O), 6.64 (1 H, s, Ar*H*), 6.61 (1 H, s, Ar*H*), 5.97 (2 H, s, OCH_2O), 5.26 (1 H, ddt, *J* = 1.9, 2.8, 5.4 Hz, ArC*H*CH_2), 5.02 (1 H, dd, *J* = 2.8, 11.7 Hz, one of ArCH_2O), 4.93 (1 H, dd, *J* = 1.9, 11.7 Hz, one of ArCH_2O), 4.05−3.93 (4 H, m, OCH_2CH_2O), 2.52 (1 H, ddt, *J* = 2.1, 17.0, 6.9 Hz, one of CH_2CH_2CHO), 2.48 (1 H, ddt, *J* = 2.1, 17.0, 6.9 Hz, one of CH_2CH_2CHO), 2.30 (1 H, dt, *J* = 14.8, 6.9 Hz, one of C$H_2$$CH_2$CHO), 2.18 (1 H, dt, *J* = 14.8, 6.9 Hz, one of C$H_2$$CH_2$CHO), 2.01 (2 H, d, *J* = 5.4 Hz, ArCHCH_2); δ_C (126 MHz; $CDCl_3$) 202.3 (CH), 147.7 (C), 147.5 (C), 135.0 (C), 131.8 (C), 109.9 (C), 101.8 (CH), 101.5 (CH), 101.4 (CH_2), 80.0 (CH), 72.5 (CH_2), 65.03 (CH_2), 64.97 (CH_2), 44.3 (CH_2), 38.4 (CH_2), 30.7 (CH_2); HRMS (ESI) *m/z* $[M + Na]^+$ calcd for $C_{16}H_{18}O_6Na$ 329.0996; found 329.0981; elemental analysis found: C, 62.63; H, 6.12. $C_{16}H_{18}O_6$ requires C, 62.74; H, 5.92%.

Diethyl 3-[2-(1,3-Dihydro-5,6-methylenedioxyisobenzofuran-1-yl)methyl-1,3-dioxolan-2-yl]-propylidenemalonate (5a). AcOH (0.01 mL, 0.174 mmol) was added to an ice-cooled (0 °C) mixture of aldehyde **S15** (200 mg, 0.653 mmol), diethyl malonate (0.12 mL, 0.784 mmol) and piperidine (0.02 mL, 0.174 mmol) in CH_2Cl_2 (3.3 mL). After 42 h of stirring at room temperature, the reaction was quenched with H_2O (10 mL), and the resulting mixture was extracted with AcOEt (2 × 40 mL). The combined organic extracts were washed with brine (2 × 20 mL) and dried over anhydrous Na_2SO_4. Filtration and evaporation in vacuo furnished the crude product (490 mg), which was purified by column chromatography (silica gel 20 g, *n*-hexane/AcOEt 2:1) to give alkene **5a** (129 mg, 44%) as a colorless oil. R_f = 0.55 (*n*-hexane/AcOEt 1:1); IR (neat) ν_{max}/cm^{-1} 2961, 2891, 1726, 1476, 1377, 1346, 1273, 1254, 1223, 1153, 1038, 941; δ_H (500 MHz; $CDCl_3$) 7.05 (1 H, t, *J* = 7.8 Hz, C*H*=C(CO_2Et)$_2$), 6.63 (1 H, s, Ar*H*), 6.61 (1 H, s, Ar*H*), 5.97 (2 H, s, OCH_2O), 5.25 (1 H, dddd, *J* = 1.8, 2.9, 6.0, 7.0 Hz, ArC*H*CH_2), 5.02 (1 H, dd, *J* = 2.9, 11.8 Hz, one of ArCH_2O), 4.92 (1 H, dd, *J* = 1.8, 11.8 Hz, one of ArCH_2O), 4.29 (2 H, q, *J* = 7.0 Hz, OC$H_2$$CH_3$), 4.23 (2 H, q, *J* = 7.3 Hz, OC$H_2$$CH_3$), 4.06−3.96 (4 H, m, OC$H_2CH_2$O), 2.47 (1 H, ddt, *J* = 7.8, 18.8, 7.0 Hz, one of CH_2CH_2CH=C), 2.44 (1 H, ddt, *J* = 7.8, 18.8, 7.0 Hz, one of CH_2CH_2CH=C), 2.02 (1 H, dd, *J* = 6.0, 14.2 Hz, one of ArCHCH_2), 2.00 (2 H, t, *J* = 7.0 Hz, C$H_2$$CH_2$CH=C), 1.97 (1 H, dd, *J* = 7.0, 14.2 Hz,

one of ArCHCH_2), 1.32 (3 H, t, J = 7.0 Hz, OCH_2CH_3), 1.29 (3 H, t, J = 7.3 Hz, OCH_2CH_3); δ_C (126 MHz; $CDCl_3$) 165.5 (C), 164.0 (C), 149.5 (CH), 147.6 (C), 147.5 (C), 135.1 (C), 131.8 (C), 128.2 (C), 109.9 (C), 101.9 (CH), 101.44 (CH), 101.37 (CH_2), 80.0 (CH), 72.4 (CH_2), 64.96 (CH_2), 64.95 (CH_2), 61.2 (CH_2), 61.1 (CH_2), 44.0 (CH_2), 36.0 (CH_2), 24.3 (CH_2), 14.09 (CH_3), 14.07 (CH_3); HRMS (ESI) m/z $[M + Na]^+$ calcd for $C_{23}H_{28}O_9Na$ 471.1626; found 471.1611.

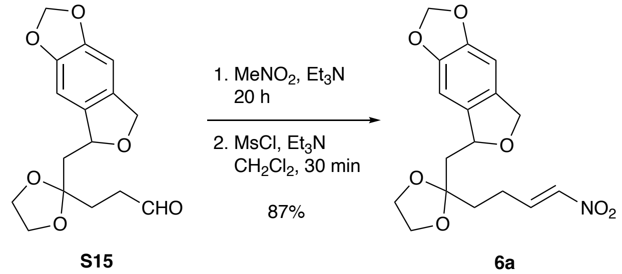

1,3-Dihydro-1-[2-((3*E*)-4-nitrobut-3-enyl)-1,3-dioxolan-2-yl]methyl-5,6-methylenedioxyiso-benzofuran (6a). Et_3N (0.11 mL, 0.784 mmol) was added to a solution of aldehyde **S15** (200 mg, 0.653 mmol) in nitromethane (4.4 mL). After 20 h of stirring, the volatile elements were removed in vacuo, and the residual yellow oil (257 mg) was used without further purification.

MsCl (80 μL, 0.979 mmol) was added to an ice-cooled (0 °C) mixture of the crude nitromethane adduct (257 mg) and Et_3N (0.18 mL, 1.31 mmol) in CH_2Cl_2 (6.5 mL). After 30 min of stirring at room temperature, the reaction was quenched with saturated aqueous $NaHCO_3$ (10 mL), and the resulting mixture was extracted with AcOEt (2 × 40 mL). The combined organic extracts were washed with brine (2 × 20 mL), and dried over anhydrous Na_2SO_4. Filtration and evaporation in vacuo furnished the crude product (318 mg), which was purified by column chromatography (silica gel 15 g, *n*-hexane/AcOEt 2:1) to give nitroalkene **6a** (199 mg, 87%) as a pale yellow solid. R_f = 0.62 (*n*-hexane/AcOEt 1:1); mp 118.0−119.0 °C (pale yellow needles from *n*-hexane/CH_2Cl_2 10:1); IR (KBr) ν_{max}/cm^{-1} 2899, 1655, 1526, 1503, 1476, 1352, 1319, 1267, 1152, 1036, 1020, 932; δ_H (500 MHz; $CDCl_3$) 7.34 (1 H, dt, J = 13.4, 7.4 Hz, CH=$CHNO_2$), 7.00 (1 H, dt, J = 13.4, 1.6 Hz, CH=C$H$$NO_2$), 6.65 (1 H, s, Ar$H$), 6.60 (1 H, s, Ar$H$), 5.97 (2 H, s, OC$H_2$O), 5.25 (1 H, ddt, J = 1.9, 2.8, 5.7 Hz, ArC$H$$CH_2$), 5.01 (1 H, dd, J = 2.8, 11.8 Hz, one of ArCH_2O), 4.93 (1 H, dd, J = 1.9, 11.8 Hz, one of ArCH_2O), 4.09−3.97 (4 H, m, OCH_2CH_2O), 2.49−2.35 (2 H, m, CH_2CH_2CH=CH), 2.09−2.01 (2 H, m, C$H_2$$CH_2$CH=CH), 2.00 (2 H, d, J = 5.7 Hz, ArCHCH_2); δ_C (126 MHz; $CDCl_3$) 147.7 (C), 147.6 (C), 143.0 (CH), 139.3 (CH), 134.9 (C), 131.8 (C), 109.8 (C), 101.8 (CH), 101.54 (CH), 101.46

(CH_2), 80.0 (CH), 72.5 (CH_2), 65.1 (CH_2), 65.0 (CH_2), 44.0 (CH_2), 35.6 (CH_2), 23.0 (CH_2); HRMS (ESI) *m*/*z* $[M + Na]^+$ calcd for $C_{17}H_{19}NO_7Na$ 372.1054; found 372.1023.

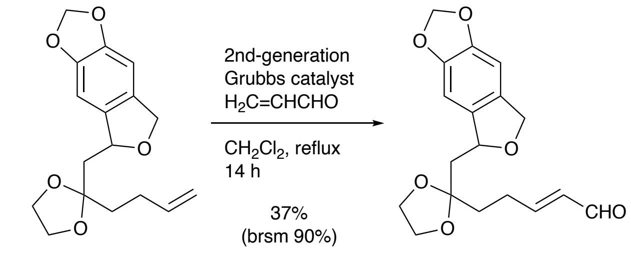

8a **7a**

(2*E*)-5-[2-(1,3-Dihydro-5,6-methylenedioxyisobenzofuran-1-yl)methyl-1,3-dioxolan-2-yl]pent-2-enal (7a). The second-generation Grubbs catalyst (61.1 mg, 70.5 μmol) was added to a mixture of alkene **8a** (430 mg, 1.41 mmol) and acrolein (2.8 mL, 42.4 mmol) in CH_2Cl_2 (9 mL). After 14 h of heating under reflux, the volatile elements were removed in vacuo, and the residue (2.24 g) was purified by column chromatography (silica gel 50 g, *n*-hexane/AcOEt 2:1) to give aldehyde **7a** (174 mg, 37%) as a pale yellow solid, along with recovered alkene **8a** (253 mg, 59%). R_f = 0.22 (*n*-hexane/AcOEt 2:1); mp 88.0−89.0 °C (colorless needles from *n*-hexane/Et_2O 2:1); IR (KBr) ν_{max}/cm^{-1} 2914, 2895, 1692, 1476, 1352, 1288, 1265, 1150, 1035, 978, 928; δ_H (500 MHz; $CDCl_3$) 9.51 (1 H, d, *J* = 7.9 Hz, C*H*O), 6.91 (1 H, dt, *J* = 15.6, 6.7 Hz, C*H*=CHCHO), 6.64 (1 H, s, Ar*H*), 6.61 (1 H, s, Ar*H*), 6.13 (1 H, ddt, *J* = 7.9, 15.6, 1.6 Hz, CH=C*H*CHO), 5.97 (2 H, s, OC*H*2O), 5.27 (1 H, m, ArC*H*CH2), 5.01 (1 H, dd, *J* = 2.8, 11.8 Hz, one of ArC*H*2O), 4.93 (1 H, dd, *J* = 1.9, 11.8 Hz, one of ArC*H*2O), 4.08−3.97 (4 H, m, OC*H*2C*H*2O), 2.56−2.43 (2 H, m, CH2C*H*2CH=CH), 2.08−1.98 (4 H, m, ArCHC*H*2, C*H*2CH2CH=CH); δ_C (126 MHz; $CDCl_3$) 194.2 (CH), 158.9 (CH), 147.7 (C), 147.5 (C), 135.1 (C), 132.7 (CH), 131.8 (C), 110.0 (C), 101.8 (CH), 101.5 (CH), 101.4 (CH_2), 80.0 (CH), 72.5 (CH_2), 65.1 (CH_2), 65.0 (CH_2), 44.1 (CH_2), 35.9 (CH_2), 27.2 (CH_2); HRMS (ESI) *m*/*z* $[M + Na]^+$ calcd for $C_{18}H_{20}O_6Na$ 355.1152; found 355.1144; elemental analysis found: C, 64.79; H, 6.20. $C_{18}H_{20}O_6$ requires C, 65.05; H, 6.07%.

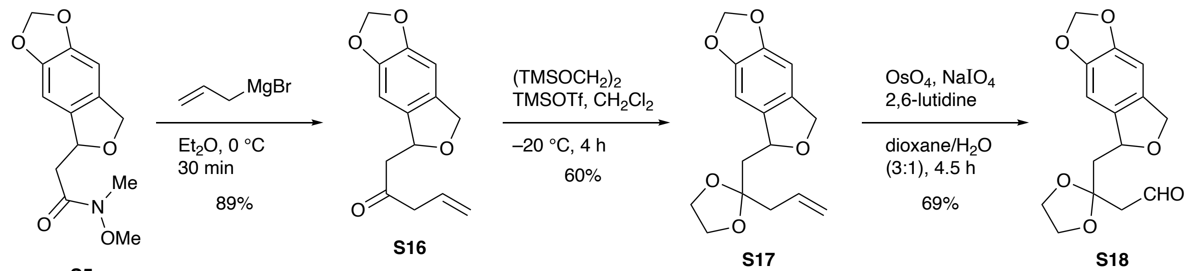

S5 **S16** **S17** **S18**

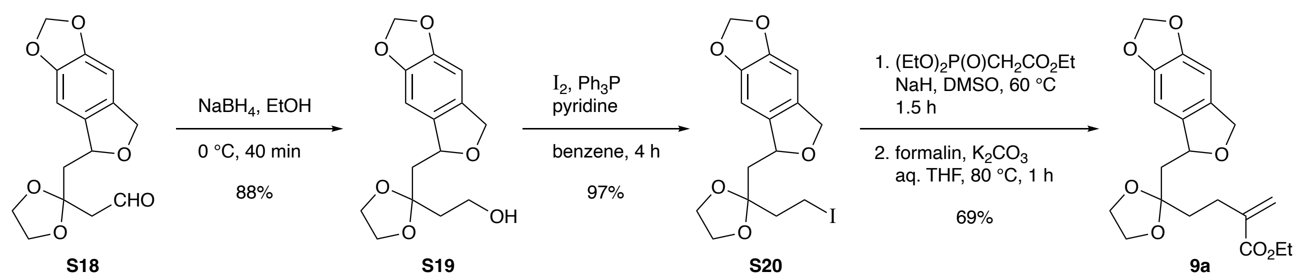

1-(1,3-Dihydro-5,6-methylenedioxyisobenzofuran-1-yl)pent-4-en-2-one (S16). Allyl bromide (0.66 mL, 7.54 mmol) was added to a suspension of magnesium (367 mg, 15.1 mmol) in Et_2O (8 mL). After 1 h of stirring, the reaction mixture was cooled to 0 °C, and a solution of amide **S5** (1.01 g, 3.77 mmol) in Et_2O (5 mL plus 2 × 1.5 mL rinse) was added. After 30 min of stirring, the reaction was quenched with 1 M aqueous hydrochloric acid (15 mL), and the resulting mixture was partitioned between AcOEt (100 mL) and H_2O (20 mL). The aqueous layer was extracted with AcOEt (100 mL), and the combined organic extracts were successively washed with H_2O (40 mL), saturated aqueous $NaHCO_3$ (40 mL), and brine (40 mL), and dried over anhydrous Na_2SO_4. Filtration and evaporation in vacuo furnished the crude product (1.11 g), which was purified by column chromatography (silica gel 20 g, *n*-hexane/AcOEt 3:1) to give ketone **S16** (829 mg, 89%) as a white solid. R_f = 0.49 (*n*-hexane/AcOEt 3:1); mp 60.0−62.0 °C (colorless needles from *n*-hexane/Et_2O 10:1); IR (KBr) ν_{max}/cm^{-1} 2905, 2862, 1701, 1499, 1474, 1368, 1348, 1275, 1260, 1159, 1098, 1040, 927; δ_H (500 MHz; $CDCl_3$) 6.64 (1 H, s, Ar*H*), 6.61 (1 H, s, Ar*H*), 5.97 (2 H, s, OC*H*$_2$O), 5.94 (1 H, ddt, *J* = 10.2, 17.2, 7.0 Hz, CH_2=C*H*), 5.57 (1 H, dddd, *J* = 1.5, 2.9, 4.9, 7.6 Hz, ArC*H*CH_2CO), 5.19 (1 H, dq, *J* = 10.2, 1.4 Hz, one of CH=C*H*$_2$), 5.15 (1 H, dq, *J* = 17.2, 1.4 Hz, one of CH=C*H*$_2$), 5.01 (1 H, dd, *J* = 2.9, 11.7 Hz, one of ArC*H*$_2$O), 4.95 (1 H, dd, *J* = 1.5, 11.7 Hz, one of ArC*H*$_2$O), 3.27 (1 H, ddt, *J* = 7.0, 16.8, 1.4 Hz, one of CH_2=CHC*H*$_2$), 3.23 (1 H, ddt, *J* = 7.0, 16.8, 1.4 Hz, one of CH_2=CHC*H*$_2$), 2.90 (1 H, dd, *J* = 7.6, 16.2 Hz, one of ArCHC*H*$_2$CO), 2.79 (1 H, dd, *J* = 4.9, 16.2 Hz, one of ArCHC*H*$_2$CO); δ_C (126 MHz; $CDCl_3$) 206.5 (C), 147.9 (C), 147.6 (C), 133.8 (C), 131.8 (C), 130.2 (CH), 119.1 (CH_2), 101.9 (CH), 101.6 (CH), 101.5 (CH_2), 79.9 (CH), 72.6 (CH_2), 49.1 (CH_2), 48.6 (CH_2); HRMS (ESI) *m*/*z* $[M + Na]^+$ calcd for $C_{14}H_{14}O_4Na$ 269.0784; found 269.0776.

1-[2-(Prop-2-enyl)-1,3-dioxolan-2-yl]methyl-1,3-dihydro-5,6-methylenedioxyisobenzofuran (S17). A solution of ketone **S16** (900 mg, 3.65 mmol) in CH_2Cl_2 (2 mL plus 2 × 0.5 mL rinse) was

added to a cooled (−78 °C) mixture of TMSOTf (20 μL, 0.111 mmol) and 1,2-bis(trimethylsiloxy)-ethane (1.1 mL, 4.48 mmol) in CH_2Cl_2 (1 mL). After 4 h of stirring at −20 °C, the reaction was quenched with pyridine (0.08 mL), and the resulting mixture was partitioned between AcOEt (100 mL) and saturated aqueous $NaHCO_3$ (20 mL). The aqueous layer was extracted with AcOEt (100 mL), and the combined organic extracts were washed with brine (40 mL) and dried over anhydrous Na_2SO_4. Filtration and evaporation in vacuo furnished the crude product (1.49 g), which was purified by column chromatography (silica gel 30 g, *n*-hexane/AcOEt 4:1) to give acetal **S17** (640 mg, 60%) as a white solid. R_f = 0.50 (*n*-hexane/AcOEt 2:1); mp 41.0−44.0 °C (colorless needles from *n*-hexane/Et_2O 10:1); IR (KBr) ν_{max}/cm^{-1} 2947, 2895, 1477, 1342, 1271, 1155, 1134, 1072, 1038, 951, 922; δ_H (500 MHz; $CDCl_3$) 6.64 (1 H, s, Ar*H*), 6.63 (1 H, s, Ar*H*), 5.96 (2 H, s, OC*H*$_2$O), 5.87 (1 H, ddt, *J* = 10.2, 17.3, 7.2 Hz, C*H*=CH$_2$), 5.30 (1 H, dddd, *J* = 1.8, 2.8, 5.5, 8.0 Hz, ArC*H*CH$_2$), 5.13 (1 H, dq, *J* = 17.3, 2.1 Hz, one of CH=C*H*$_2$), 5.11 (1 H, dq, *J* = 10.2, 2.1 Hz, one of CH=C*H*$_2$), 5.02 (1 H, dd, *J* = 2.8, 11.8 Hz, one of ArC*H*$_2$O), 4.93 (1 H, dd, *J* = 1.8, 11.8 Hz, one of ArC*H*$_2$O), 4.08−3.98 (4 H, m, OC*H*$_2$C*H*$_2$O), 2.55 (1 H, ddt, *J* = 7.2, 14.2, 2.1 Hz, one of CH$_2$=CHC*H*$_2$), 2.53 (1 H, ddt, *J* = 7.2, 14.2, 2.1 Hz, one of CH$_2$=CHC*H*$_2$), 2.08 (1 H, dd, *J* = 8.0, 15.0 Hz, one of ArCHC*H*$_2$), 2.03 (1 H, dd, *J* = 5.5, 15.0 Hz, one of ArCHC*H*$_2$); δ_C (126 MHz; $CDCl_3$) 147.5 (C), 147.4 (C), 135.4 (C), 133.3 (CH), 131.9 (C), 118.3 (CH_2), 109.9 (C), 102.0 (CH), 101.40 (CH), 101.36 (CH_2), 79.9 (CH), 72.4 (CH_2), 65.04 (CH_2), 64.98 (CH_2), 43.9 (CH_2), 42.6 (CH_2); HRMS (ESI) *m*/*z* $[M + Na]^+$ calcd for $C_{16}H_{18}O_5Na$ 313.1046; found 313.1031; elemental analysis found: C, 65.96; H, 6.41. $C_{16}H_{18}O_5$ requires C, 66.20; H, 6.25%.

2-[2-(1,3-Dihydro-5,6-methylenedioxyisobenzofuran-1-yl)methyl-1,3-dioxolan-2-yl]ethanal (S18). A 0.157 M solution of OsO_4 in *t*-BuOH (45 μL, 6.7 μmol) was added to a mixture of alkene **S17** (97.1 mg, 0.335 mmol), 2,6-lutidine (80 μL, 0.67 mmol) and $NaIO_4$ (293 mg, 1.37 mmol) in dioxane/H_2O (3:1, 1.6 mL). After 4.5 h of stirring, the reaction was quenched with 1 M aqueous $Na_2S_2O_3$ (10 mL), and the resulting mixture was filtered through a Celite pad. The filtrate was extracted with AcOEt (10 mL), and the organic extract was successively washed with 1 M aqueous hydrochloric acid (10 mL), saturated aqueous $NaHCO_3$ (10 mL), and brine (10 mL), and dried over anhydrous Na_2SO_4. Filtration and evaporation in vacuo furnished the crude product (94.5 mg),

which was purified by column chromatography (silica gel 5 g, *n*-hexane/AcOEt 5:1) to give aldehyde **S18** (67.4 mg, 69%) as a white solid. R_f = 0.43 (toluene/AcOEt 6:1); mp 100.0−102.0 °C (colorless plates from Et_2O); IR (KBr) ν_{max}/cm^{-1} 2889, 1715, 1506, 1476, 1371, 1352, 1323, 1269, 1254, 1148, 1125, 1088, 1042, 1015; δ_H (500 MHz; $CDCl_3$) 9.79 (1 H, t, *J* = 2.6 Hz, C*H*O), 6.64 (1 H, s, Ar*H*), 6.60 (1 H, s, Ar*H*), 5.97 (2 H, s, OCH_2O), 5.30 (1 H, dddd, *J* = 1.5, 2.8, 3.7, 7.9 Hz, ArC*H*CH_2), 5.00 (1 H, dd, *J* = 2.8, 11.8 Hz, one of ArCH_2O), 4.93 (1 H, dd, *J* = 1.5, 11.8 Hz, one of ArCH_2O), 4.14−4.01 (4 H, m, OCH_2CH_2O), 2.96 (1 H, dd, *J* = 2.6, 15.7 Hz, one of CH_2CHO), 2.88 (1 H, dd, *J* = 2.6, 15.7 Hz, one of CH_2CHO), 2.12 (1 H, dd, *J* = 7.9, 14.9 Hz, one of ArCHCH_2), 2.08 (1 H, dd, *J* = 3.7, 14.9 Hz, one of ArCHCH_2); δ_C (126 MHz; $CDCl_3$) 200.5 (CH), 147.8 (C), 147.6 (C), 134.6 (C), 131.8 (C), 108.3 (C), 101.8 (CH), 101.6 (CH), 101.5 (CH_2), 79.8 (CH), 72.5 (CH_2), 65.0 (2 × CH_2), 51.1 (CH_2), 44.7 (CH_2); HRMS (ESI) *m*/*z* $[M + Na]^+$ calcd for $C_{15}H_{16}O_6Na$ 315.0839; found 315.0850.

2-[2-(1,3-Dihydro-5,6-methylenedioxyisobenzofuran-1-yl)methyl-1,3-dioxolan-2-yl]ethanol (S19). Aldehyde **S18** (52.8 mg, 0.209 mmol) was added to an ice-cooled (0 °C) solution of $NaBH_4$ (3.9 mg, 0.10 mmol) in EtOH (0.9 mL). After 40 min of stirring, brine (5 mL) and H_2O (1 mL) were added, and the mixture was extracted with AcOEt (5 mL). The organic extract was washed with brine (5 mL) and dried over anhydrous Na_2SO_4. Filtration and evaporation in vacuo furnished the crude product (67.7 mg), which was purified by column chromatography (silica gel 5 g, *n*-hexane/ AcOEt 3:1 → AcOEt) to give alcohol **S19** (44.2 mg, 88%) as a colorless oil. R_f = 0.39 (*n*-hexane/ AcOEt 2:1); IR (neat) ν_{max}/cm^{-1} 3441, 2955, 2887, 1503, 1476, 1379, 1346, 1271, 1153, 1128, 1038, 939; δ_H (500 MHz; $CDCl_3$) 6.64 (1 H, s, Ar*H*), 6.61 (1 H, s, Ar*H*), 5.97 (2 H, s, OCH_2O), 5.28 (1 H, dddd, *J* = 1.5, 2.3, 2.8, 8.6 Hz, ArC*H*CH_2), 5.02 (1 H, dd, *J* = 2.8, 11.7 Hz, one of ArCH_2O), 4.93 (1 H, dd, *J* = 1.5, 11.7 Hz, one of ArCH_2O), 4.09−4.02 (4 H, m, OCH_2CH_2O), 3.80 (2 H, br dt, *J* = 4.5, 6.3 Hz, CH_2OH), 2.84 (1 H, br t, *J* = 4.5 Hz, CH_2O*H*), 2.13 (1 H, dd, *J* = 8.6, 14.9 Hz, one of ArCHCH_2), 2.12 (2 H, t, *J* = 6.3 Hz, C$H_2$$CH_2$OH), 2.04 (1 H, dd, *J* = 2.3, 14.9 Hz, one of ArCHCH_2); δ_C (126 MHz; $CDCl_3$) 147.7 (C), 147.5 (C), 134.9 (C), 131.7 (C), 111.1 (C), 101.8 (CH), 101.5 (CH), 101.4 (CH_2), 80.0 (CH), 72.5 (CH_2), 64.83 (CH_2), 64.80 (CH_2), 58.9 (CH_2), 43.9 (CH_2), 39.1 (CH_2); HRMS (ESI) *m*/*z* $[M + Na]^+$ calcd for $C_{15}H_{18}O_6Na$ 317.0996; found 317.0997.

1-[2-(2-Iodoethyl)-1,3-dioxolan-2-yl]methyl-1,3-dihydro-5,6-methylenedioxyisobenzofuran (S20). Iodine (266 mg, 1.05 mmol) was added to a mixture of alcohol **S19** (201 mg, 0.683 mmol), triphenylphosphine (271 mg, 1.03 mmol), and pyridine (0.16 mL, 1.99 mmol) in benzene (3.5 mL). After 4 h of stirring, the reaction was quenched with 1 M aqueous $Na_2S_2O_3$ (20 mL), and the resulting mixture was extracted with *n*-hexane/AcOEt (4:1, 15 mL). The organic extract was washed with brine (2 × 15 mL) and dried over anhydrous Na_2SO_4. Filtration and evaporation in vacuo furnished the crude product (713 mg), which was purified by column chromatography (silica gel 15 g, 7:1 *n*-hexane/AcOEt) to give iodide **S20** (267 mg, 97%) as a white solid. R_f = 0.74 (*n*-hexane/AcOEt 1:1); mp 96.0−97.0 °C (colorless needles from *n*-hexane); IR (KBr) ν_{max}/cm^{-1} 2891, 2847, 1508, 1474, 1335, 1283, 1198, 1144, 1125, 1034, 1022; δ_H (500 MHz; $CDCl_3$) 6.64 (1 H, s, Ar*H*), 6.61 (1 H, s, Ar*H*), 5.97 (2 H, s, OCH_2O), 5.25 (1 H, dddd, *J* = 1.5, 2.7, 3.3, 8.0 Hz, ArC*H*CH_2), 5.02 (1 H, dd, *J* = 2.7, 11.8 Hz, one of ArCH_2O), 4.93 (1 H, dd, *J* = 1.5, 11.8 Hz, one of ArCH_2O), 4.08−3.97 (4 H, m, OCH_2CH_2O), 3.24 (1 H, dt, *J* = 9.4, 8.5 Hz, one of CH_2I), 3.18 (1 H, dt, *J* = 9.4, 8.5 Hz, one of CH_2I), 2.45 (2 H, t, *J* = 8.5 Hz, C$H_2$$CH_2$I), 2.01 (1 H, dd, *J* = 8.0, 14.9 Hz, one of ArCHCH_2), 1.97 (1 H, dd, *J* = 3.3, 14.9 Hz, one of ArCHCH_2); δ_C (126 MHz; $CDCl_3$) 147.7 (C), 147.5 (C), 135.0 (C), 131.9 (C), 110.5 (C), 101.9 (CH), 101.5 (CH), 101.4 (CH_2), 79.8 (CH), 72.6 (CH_2), 65.14 (CH_2), 65.09 (CH_2), 43.8 (CH_2), 43.1 (CH_2), −2.2 (CH_2); HRMS (ESI) *m*/*z* $[M + Na]^+$ calcd for $C_{15}H_{17}IO_5Na$ 427.0013; found 427.0024.

Ethyl 4-[2-(1,3-Dihydro-5,6-methylenedioxyisobenzofuran-1-yl)methyl-1,3-dioxoran-2-yl]-2-methylidenebutanoate (9a). Triethyl phosphonoacetate (0.12 mL, 0.60 mmol) was added to a suspension of NaH (60% in oil, 30.2 mg, 0.755 mmol) in DMSO (0.40 mL). After 30 min of stirring, a solution of iodide **S20** (201 mg, 0.496 mmol) in DMSO (0.4 mL plus 2 × 0.1 mL rinse) was added, and the mixture was heated at 60 °C for 1.5 h. After cooling to 20 °C, the reaction was quenched with H_2O (20 mL), and the resulting mixture was extracted with AcOEt (2 × 20 mL). The combined organic extracts were washed with brine (2 × 20 mL) and dried over anhydrous Na_2SO_4. Filtration and evaporation in vacuo furnished the crude product (242 mg), which was used without further purification.

A solution of K_2CO_3 in H_2O (20%, 0.25 mL, 0.36 mmol) was added to an ice-cooled (0 °C) mix-

ture of the crude phosphonate (242 mg) and formalin (37%, 0.50 mL, 5.1 mmol) in THF (0.50 mL), and the resulting mixture was heated at 80 °C for 1 h. After cooling to 0 °C, the reaction mixture was partitioned between *n*-hexane/AcOEt (4:1, 15 mL) and H_2O (15 mL). The organic layer was washed with brine (2 × 15 mL) and dried over anhydrous Na_2SO_4. Filtration and evaporation in vacuo furnished the crude product (321 mg), which was purified by column chromatography (silica gel 15 g, *n*-hexane/AcOEt 6:1) to give enoate **9a** (129 mg, 69% for two steps) as a colorless oil. R_f = 0.65 (*n*-hexane/AcOEt 1:1); IR (neat) ν_{max}/cm^{-1} 2980, 2957, 2887, 1715, 1630, 1503, 1476, 1369, 1346, 1269, 1179, 1152, 1040, 943; δ_H (500 MHz; $CDCl_3$) 6.65 (1 H, s, Ar*H*), 6.63 (1 H, s, Ar*H*), 6.14 (1 H, d, *J* = 1.3 Hz, one of C=C*H*$_2$), 5.96 (2 H, s, OC*H*$_2$O), 5.55 (1 H, d, *J* = 1.3 Hz, one of C=C*H*$_2$), 5.28 (1 H, dddd, *J* = 1.6, 2.7, 4.6, 7.9 Hz, ArC*H*CH$_2$), 5.02 (1 H, dd, *J* = 2.7, 11.7 Hz, one of ArC*H*$_2$O), 4.92 (1 H, dd, *J* = 1.6, 11.7 Hz, one of ArC*H*$_2$O), 4.20 (2 H, q, *J* = 7.1 Hz, OC*H*$_2$CH$_3$), 4.07–3.98 (4 H, m, OC*H*$_2$C*H*$_2$O), 2.44 (2 H, t, *J* = 6.7 Hz, C*H*$_2$C(=CH$_2$)CO$_2$Et), 2.10 (1 H, dd, *J* = 7.9, 14.9 Hz, one of ArCHC*H*$_2$), 2.05 (1 H, dd, *J* = 4.6, 14.9 Hz, one of ArCHC*H*$_2$), 1.97 (1 H, dt, *J* = 17.0, 6.7 Hz, one of C*H*$_2$CH$_2$C(=CH$_2$)CO$_2$Et), 1.94 (1 H, dt, *J* = 17.0, 6.7 Hz, one of C*H*$_2$CH$_2$C(=CH$_2$)CO$_2$Et), 1.29 (3 H, t, *J* = 7.1 Hz, OCH$_2$C*H*$_3$); δ_C (126 MHz; $CDCl_3$) 167.1 (C), 147.54 (C), 147.45 (C), 140.7 (C), 135.4 (C), 131.9 (C), 124.3 (CH_2), 110.2 (C), 102.0 (CH), 101.42 (CH), 101.38 (CH_2), 80.0 (CH), 72.5 (CH_2), 65.0 (CH_2), 64.9 (CH_2), 60.6 (CH_2), 43.9 (CH_2), 36.4 (CH_2), 26.4 (CH_2), 14.2 (CH_3); HRMS (ESI) *m*/*z* $[M + Na]^+$ calcd for $C_{20}H_{24}O_7Na$ 399.1414; found 399.1416.

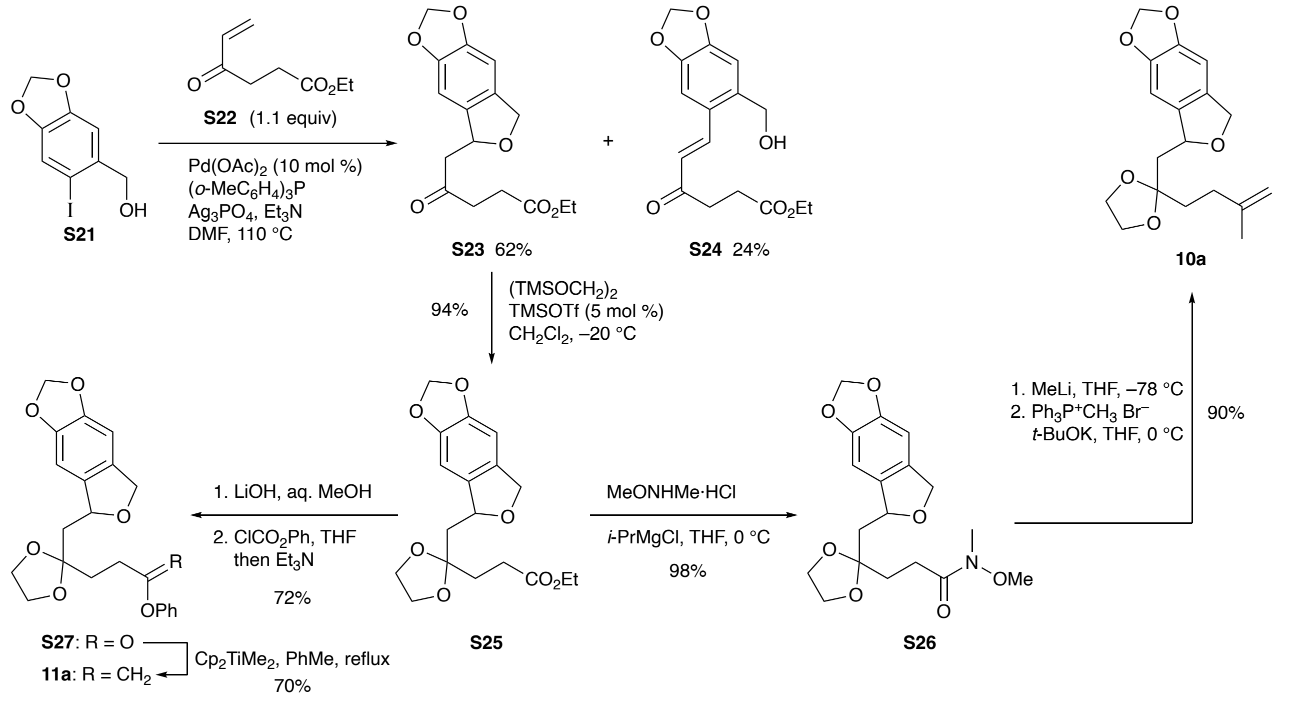

Ethyl 5-(1,3-Dihydro-5,6-methylenedioxyisobenzofuran-1-yl)-4-oxopentanoate (S23). Palladium(II) acetate (173 mg, 0.771 mmol) was added to a mixture of iodide **S21**[75] (2.15 g, 7.73 mmol), enone **S22**[76] (1.33 g, 8.52 mmol), tri(*o*-tolyl)phosphine (938 mg, 3.08 mmol), Et_3N (3.3 mL, 23.2 mmol) and Ag_3PO_4 (330 mg, 0.788 mmol) in DMF (16 mL). After 36 h of stirring at 110 °C, the reaction was quenched with 1 M aqueous hydrochloric acid (30 mL). The resulting mixture was diluted with *n*-hexane/AcOEt (1:9, 100 mL) and filtered through a Celite pad. The layers were separated, and the aqueous layer was extracted with *n*-hexane/AcOEt (1:9, 50 mL). The combined organic extracts were successively washed with water (40 mL), saturated aqueous $NaHCO_3$ (50 mL) and brine (2 × 50 mL) and dried over anhydrous Na_2SO_4. Filtration and evaporation in vacuo furnished the crude product (4.17 g, brown solid), which was purified by column chromatography (silica gel 210 g, *n*-hexane/AcOEt 3:1 → 5:3 → 4:3→ 1:1) to give phthalan **S23** (1.48 g, 62%) as a yellow solid, along with enone **S24** (580 mg, 24%) as a yellow solid. R_f = 0.60 (*n*-hexane/AcOEt 1:1); mp 51.8−53.0 °C (colorless needles from *n*-hexane/AcOEt 5:3); IR (KBr) ν_{max}/cm^{-1} 2978, 2911, 1724, 1711, 1501, 1476, 1377, 1342, 1275, 1263, 1198, 1161, 1098, 1038, 926, 860; δ_H (500 MHz; $CDCl_3$) 6.64 (1 H, s, Ar*H*), 6.61 (1 H, s, Ar*H*), 5.97 (2 H, s, OCH_2O), 5.58 (1 H, dddd, *J* = 1.4, 3.2, 5.1, 7.5 Hz, ArC*H*CH_2CO), 5.02 (1 H, dd, *J* = 3.2, 11.7 Hz, one of $ArCH_2O$), 4.95 (1 H, dd, *J* = 1.4, 11.7 Hz, one of $ArCH_2O$), 4.15 (1 H, dq, *J* = 10.9, 7.2 Hz, one of OCH_2CH_3), 4.13 (1 H, dq, *J* = 10.9, 7.2 Hz, one of OCH_2CH_3), 2.90 (1 H, dd, *J* = 7.5, 16.0 Hz, one of $ArCHCH_2CO$), 2.83 (1 H, dt, *J* = 18.4, 6.6 Hz, one of $CH_2CH_2CO_2Et$), 2.82 (1 H, dd, *J* = 5.1, 16.0 Hz, one of $ArCHCH_2CO$), 2.79 (1 H, dt, *J* = 18.4, 6.6 Hz, one of $CH_2CH_2CO_2Et$), 2.64 (1 H, dt, *J* = 17.2, 6.6 Hz, one of $CH_2CH_2CO_2Et$), 2.58 (1 H, dt, *J* = 17.2, 6.6 Hz, one of $CH_2CH_2CO_2Et$), 1.26 (3 H, t, *J* = 7.2 Hz, OCH_2CH_3); δ_C (126 MHz; $CDCl_3$) 206.7 (C), 172.7 (C), 147.9 (C), 147.6 (C), 133.8 (C), 131.8 (C), 101.9 (CH), 101.52 (CH), 101.49 (CH_2), 79.9 (CH), 72.6 (CH_2), 60.1 (CH_2), 49.6 (CH_2), 38.2 (CH_2), 27.8 (CH_2), 14.2 (CH_3); HRMS (ESI) *m*/*z* $[M + Na]^+$ calcd for $C_{16}H_{18}O_6Na$ 329.1001; found 329.1023; elemental analysis found: C, 62.45; H, 5.91. $C_{16}H_{18}O_6$ requires C, 62.74; H, 5.92%.

Data for ethyl (5*E*)-6-[2-(hydroxymethyl)-4,5-(methylenedioxy)phenyl]-4-oxohex-5-enoate (**S24**). R_f = 0.28 (*n*-hexane/AcOEt 1:1); mp 113.6−114.8 °C (colorless needles from AcOEt); IR (KBr) ν_{max}/cm^{-1} 3393, 2972, 2914, 1717, 1661, 1503, 1479, 1360, 1281, 1223, 1165, 1038, 1005; δ_H (500 MHz; $CDCl_3$) 7.90 (1 H, d, *J* = 16.0 Hz, ArC*H*=CHCO), 7.11 (1 H, s, Ar*H*), 6.93 (1 H, s, Ar*H*), 6.60

(1 H, d, J = 16.0 Hz, ArCH=C*H*CO), 6.01 (2 H, s, OCH_2O), 4.76 (2 H, s, ArCH_2OH), 4.15 (2 H, q, J = 7.2 Hz, OC$H_2$$CH_3$), 2.99 (2 H, t, J = 6.6 Hz, C$H_2$$CH_2$$CO_2$Et), 2.68 (2 H, t, J = 6.6 Hz, CH_2C$H_2$$CO_2$Et), 1.27 (3 H, t, J = 7.2 Hz, OCH_2CH_3); δ_C (126 MHz; $CDCl_3$) 198.0 (C), 173.0 (C), 149.7 (C), 147.8 (C), 138.9 (CH), 135.7 (C), 126.9 (C), 125.5 (CH), 109.3 (CH), 106.0 (CH), 101.7 (CH_2), 62.6 (CH_2), 60.7 (CH_2), 35.4 (CH_2), 28.3 (CH_2), 14.2 (CH_3); HRMS (ESI) *m*/*z* $[M + Na]^+$ calcd for $C_{16}H_{18}O_6Na$ 329.1001; found 329.1006.

Ethyl 3-[2-(1,3-Dihydro-5,6-methylenedioxyisobenzofuran-1-yl)methyl-1,3-dioxoran-2-yl]propionate (S25). The acetalization was performed according to the typical procedure (25 mL CH_2Cl_2, −20 °C, 32 h) employing ketone **S23** (1.14 g, 3.73 mmol), TMSOTf (35 μL, 187 μmol), and 1,2-bis-(trimethylsiloxy)ethane (1.19 mL, 4.85 mmol). The crude product (1.48 g, yellow oil) was purified by column chromatography (silica gel 40 g, *n*-hexane/AcOEt 3:1) to give acetal **S25** (1.23 g, 94%) as a colorless oil. R_f = 0.63 (*n*-hexane/AcOEt 1:1); IR (neat) ν_{max}/cm^{-1} 2978, 2889, 1732, 1501, 1476, 1373, 1344, 1271, 1180, 1153, 1126, 1038, 941; δ_H (500 MHz; $CDCl_3$) 6.64 (1 H, s, Ar*H*), 6.63 (1 H, s, Ar*H*), 5.97 (2 H, s, OCH_2O), 5.26 (1 H, dddd, J = 1.7, 2.9, 3.8, 7.5 Hz, ArC*H*CH_2), 5.02 (1 H, dd, J = 2.9, 11.8 Hz, one of ArCH_2O), 4.93 (1 H, dd, J = 1.7, 11.8 Hz, one of ArCH_2O), 4.13 (2 H, q, J = 7.2 Hz, OC$H_2$$CH_3$), 4.07−3.98 (4 H, m, OC$H_2CH_2$O), 2.45 (1 H, ddd, J = 6.8, 8.7, 15.7 Hz, one of C$H_2$$CO_2$Et), 2.40 (1 H, ddd, J = 6.4, 8.4, 15.7 Hz, one of C$H_2$$CO_2$Et), 2.20 (1 H, ddd, J = 6.4, 8.7, 14.4 Hz, one of C$H_2$$CH_2$$CO_2$Et), 2.14 (1 H, ddd, J = 6.8, 8.4, 14.4 Hz, one of C$H_2$$CH_2$$CO_2$Et), 2.04 (1 H, dd, J = 7.5, 14.9 Hz, one of ArCHCH_2), 2.01 (1 H, dd, J = 3.8, 14.9 Hz, one of ArCHCH_2), 1.26 (3 H, t, J = 7.2 Hz, OCH_2CH_3); δ_C (126 MHz; $CDCl_3$) 173.6 (C), 147.6 (C), 147.5 (C), 135.2 (C), 131.9 (C), 109.9 (C), 101.9 (CH), 101.5 (CH), 101.4 (CH_2), 80.0 (CH), 72.5 (CH_2), 65.0 (2 × CH_2), 60.3 (CH_2), 44.1 (CH_2), 32.8 (CH_2), 29.0 (CH_2), 14.2 (CH_3); HRMS (ESI) *m*/*z* $[M + Na]^+$ calcd for $C_{18}H_{22}O_7Na$ 373.1263; found 373.1269.

3-[2-(1,3-Dihydro-5,6-methylenedioxyisobenzofuran-1-yl)methyl-1,3-dioxoran-2-yl]-*N*-methoxy-*N*-methylpropanamide (S26). *i*-PrMgCl in THF (2.0 M, 1.60 mL, 3.20 mmol) was added to an ice-cooled (0 °C) mixture of ester **S25** (278 mg, 0.793 mmol) and MeONHMe·HCl (156 mg, 1.60 mmol) in THF (7 mL). After 3 h of stirring, the reaction was quenched with saturated aqueous

NH_4Cl (16 mL), and the resulting mixture was extracted with AcOEt (2 × 40 mL). The combined organic extracts were washed with brine (20 mL) and dried over anhydrous Na_2SO_4. Filtration and evaporation in vacuo furnished the crude product (331 mg, yellow solid), which was purified by column chromatography (silica gel 10 g, *n*-hexane/AcOEt 1:1) to give amide **S26** (284 mg, 98%) as a white solid. R_f = 0.10 (*n*-hexane/AcOEt 1:1); IR (KBr) ν_{max}/cm^{-1} 3441, 2941, 2889, 1651, 1476, 1346, 1283, 1271, 1250, 1155, 1045, 1036, 941; δ_H (500 MHz; $CDCl_3$) 6.65 (1 H, s, Ar*H*), 6.63 (1 H, s, Ar*H*), 5.97 (2 H, s, OCH_2O), 5.29 (1 H, ddt, *J* = 2.2, 8.0, 2.9 Hz, $ArCHCH_2$), 5.02 (1 H, dd, *J* = 2.9, 11.8 Hz, one of $ArCH_2O$), 4.93 (1 H, dd, *J* = 2.2, 11.8 Hz, one of $ArCH_2O$), 4.09−4.00 (4 H, m, OCH_2CH_2O), 3.69 (3 H, s, $NOCH_3$), 3.18 (3 H, s, NCH_3), 2.56 (1 H, dt, *J* = 15.3, 7.9 Hz, one of CH_2CO), 2.56 (1 H, dt, *J* = 15.3, 7.9 Hz, one of CH_2CO), 2.14 (2 H, t, *J* = 7.9 Hz, CH_2CH_2CO), 2.10 (1 H, dd, *J* = 8.0, 14.8 Hz, one of $ArCHCH_2$), 2.04 (1 H, dd, *J* = 2.9, 14.8 Hz, one of $ArCHCH_2$); δ_C (126 MHz; $CDCl_3$) 174.4 (C), 147.6 (C), 147.5 (C), 135.3 (C), 131.9 (C), 110.1 (C), 102.0 (CH), 101.42 (CH), 101.39 (CH_2), 79.9 (CH), 72.5 (CH_2), 65.0 (CH_2), 64.9 (CH_2), 61.2 (CH_3), 43.9 (CH_2), 32.3 (CH_3), 32.2 (CH_2), 26.6 (CH_2); HRMS (ESI) *m/z* $[M + Na]^+$ calcd for $C_{18}H_{23}NO_7Na$ 388.1372; found 388.1402.

1-[2-(3-Methylbut-3-enyl)-1,3-dioxolan-2-yl]methyl-1,3-dihydro-5,6-methylenedioxyisobenzofuran (10a). MeLi in diethoxymethane (3.1 M, 0.30 mL, 0.93 mmol) was added to a cooled (−78 °C) solution of amide **S26** (201 mg, 0.550 mmol) in THF (3.5 mL). After 1 h of stirring, the reaction mixture was poured into ice-cooled (0 °C) saturated aqueous NH_4Cl (10 mL), and the resulting mixture was extracted with AcOEt (2 × 20 mL). The combined organic extracts were washed with brine (20 mL) and dried over anhydrous Na_2SO_4. Filtration and evaporation in vacuo furnished the crude product (189 mg, yellow solid), which was used without further purification.

Methyltriphenylphosphonium bromide (997 mg, 2.79 mmol) was added to an ice-cooled (0 °C) solution of *t*-BuOK (302 mg, 2.69 mmol) in THF (11 mL). After 35 min of stirring at room temperature, a solution of the crude ketone (189 mg) in THF (3.5 mL plus a 2 mL rinse) was added at 0 °C, and the mixture was stirred for 1 h. The reaction was quenched with saturated aqueous NH_4Cl (20 mL), and the resulting mixture was extracted with AcOEt (2 × 30 mL). The combined organic extracts were washed with brine (30 mL) and dried over Na_2SO_4. Filtration and evaporation in vacuo

furnished the crude product (899 mg, white solid), which was purified by column chromatography (silica gel 27 g, *n*-hexane/AcOEt 10:1 → 6:1) to give alkene **10a** (157 mg, 90%) as a colorless oil. R_f = 0.60 (*n*-hexane/AcOEt 2:1); IR (neat) ν_{max}/cm^{-1} 2955, 2885, 1647, 1501, 1474, 1375, 1346, 1271, 1152, 1038, 943; δ_H (500 MHz; C_6D_6) 6.61 (1 H, s, Ar*H*), 6.28 (1 H, s, Ar*H*), 5.47 (1 H, dddd, *J* = 1.9, 2.8, 3.2, 8.0 Hz, ArC*H*CH$_2$), 5.34 (1 H, d, *J* = 1.1 Hz, one of OC*H*$_2$O), 5.33 (1 H, d, *J* = 1.1 Hz, one of OC*H*$_2$O), 4.88 (1 H, s, one of CMe=C*H*$_2$), 4.81 (1 H, s, one of CMe=C*H*$_2$), 4.78 (1 H, dd, *J* = 2.8, 11.8 Hz, one of ArC*H*$_2$O), 4.70 (1 H, dd, *J* = 1.9, 11.8 Hz, one of ArC*H*$_2$O), 3.62−3.50 (4 H, m, OC*H*$_2$C*H*$_2$O), 2.39−2.27 (2 H, m, CH$_2$=CMeC*H*$_2$), 2.24 (1 H, dd, *J* = 8.0, 14.8 Hz, one of ArCHC*H*$_2$), 2.19 (1 H, ddd, *J* = 5.6, 11.1, 13.6 Hz, one of CH$_2$=CMeCH$_2$C*H*$_2$), 2.11 (1 H, ddd, *J* = 5.1, 11.5, 13.6 Hz, one of CH$_2$=CMeCH$_2$C*H*$_2$), 2.02 (1 H, dd, *J* = 3.2, 14.8 Hz, one of ArCHC*H*$_2$), 1.71 (3 H, s, CC*H*$_3$=CH$_2$); δ_C (126 MHz; C_6D_6) 148.0 (C), 147.9 (C), 146.1 (C), 136.3 (C), 132.5 (C), 110.9 (C), 110.0 (CH$_2$), 102.5 (CH$_2$), 101.7 (CH), 101.3 (CH), 80.6 (CH), 72.4 (CH$_2$), 64.9 (2 × CH$_2$), 44.6 (CH$_2$), 36.8 (CH$_2$), 32.4 (CH$_2$), 22.8 (CH$_3$); HRMS (ESI) *m*/*z* [M + Na]$^+$ calcd for $C_{18}H_{22}O_5Na$ 341.1365; found 341.1367.

Phenyl 3-[2-(1,3-Dihydro-5,6-methylenedioxyisobenzofuran-1-yl)methyl-1,3-dioxoran-2-yl]propionate (S27). Lithium hydroxide monohydrate (3.61 mg, 85.6 μmol) was added to a solution of ester **S25** (19.8 mg, 56.5 μmol) in MeOH/H_2O (1:1, 2 mL). After 16 h of stirring, the volatile elements were removed in vacuo and the residue was azeotropically dried with THF and toluene to furnish the crude lithium carboxylate (21.3 mg), which was used without further purification.

Phenyl chloroformate (21.6 μL, 0.171 mmol) was added to a solution of the crude lithium carboxylate (21.3 mg) in THF (1 mL). After 21 h of stirring, Et_3N (23.7 μL, 0.171 mmol) was added and the resulting mixture was stirred for 1 h. After dilution with AcOEt (8 mL), the reaction was quenched with H_2O (4 mL), and the layers were separated. The organic layer was washed with brine (2 × 2 mL) and dried over anhydrous Na_2SO_4. Filtration and evaporation in vacuo furnished the crude product (28.3 mg), which was purified by column chromatography (silica gel 200 mg, CH_2Cl_2 → *n*-hexane/AcOEt 9:1 → 4:1) to give phenyl ester **S27** (16.4 mg, 72%) as an orange oil. R_f = 0.61 (*n*-hexane/AcOEt 1:1); IR (neat) ν_{max}/cm^{-1} 2955, 2891, 2851, 1751, 1593, 1491, 1474, 1342, 1271, 1198, 1161, 1038, 939; δ_H (500 MHz; $CDCl_3$) 7.37 (2 H, m, Ph*H*), 7.21 (1 H, m, Ph*H*), 7.09 (2 H, m,

Ph*H*), 6.64 (2 H, s, Ar*H*), 5.96 (2 H, s, OCH_2O), 5.30 (1 H, dddd, *J* = 1.9, 2.9, 4.0, 7.3 Hz, ArC*H*CH_2), 5.03 (1 H, dd, *J* = 2.9, 11.8 Hz, one of ArCH_2O), 4.94 (1 H, dd, *J* = 1.9, 11.8 Hz, one of ArCH_2O), 4.09−4.02 (4 H, m, OCH_2CH_2O), 2.69 (1 H, dt, *J* = 15.7, 7.3 Hz, one of C$H_2$$CO_2$Ph), 2.65 (1 H, dt, *J* = 15.7, 7.3 Hz, one of C$H_2$$CO_2$Ph), 2.37 (1 H, dt, *J* = 14.5, 7.3 Hz, one of C$H_2$$CH_2$$CO_2$Ph), 2.29 (1 H, dt, *J* = 14.5, 7.3 Hz, one of C$H_2$$CH_2$$CO_2$Ph), 2.09 (1 H, dd, *J* = 7.3, 14.9 Hz, one of ArCHCH_2), 2.06 (1 H, dd, *J* = 4.0, 14.9 Hz, one of ArCHCH_2); δ_C (126 MHz; $CDCl_3$) 172.2 (C), 150.9 (C), 147.6 (C), 147.5 (C), 135.1 (C), 131.9 (C), 129.3 (2 × CH), 125.6 (CH), 121.6 (2 × CH), 109.9 (C), 101.9 (CH), 101.5 (CH), 101.4 (CH_2), 80.0 (CH), 72.5 (CH_2), 65.14 (CH_2), 65.09 (CH_2), 44.2 (CH_2), 33.0 (CH_2), 29.2 (CH_2); HRMS (ESI) *m*/*z* $[M + Na]^+$ calcd for $C_{22}H_{22}O_7Na$ 421.1263; found 421.1262.

1-[2-(3-Phenoxybut-3-enyl)-1,3-dioxolan-2-yl]methyl-1,3-dihydro-5,6-methylenedioxyisobenzofuran (11a). Dimethyltitanocene in toluene/THF (5%, 986 μL, 0.211 mmol) was added to a solution of phenyl ester **S27** (28.0 mg, 70.3 μmol) in toluene (1.4 mL), and the mixture was refluxed for 2 h. After cooling to room temperature, the reaction mixture was filtered through a Celite pad, and the filtrate was evaporated in vacuo. Purification of the residue (42.1 mg) by column chromatography (silica gel 1 g, *n*-hexane/AcOEt 9:1 → 4:1) afforded enol ether **11a** (19.4 mg, 70%) as a colorless oil. R_f = 0.47 (*n*-hexane/AcOEt 2:1); IR (neat) ν_{max}/cm^{-1} 2955, 2922, 2889, 2853, 1638, 1593, 1476, 1346, 1269, 1221, 1140, 959, 943; δ_H (500 MHz; $CDCl_3$) 7.32 (2 H, m, Ph*H*), 7.10 (1 H, m, Ph*H*), 7.03 (2 H, m, Ph*H*), 6.66 (1 H, s, Ar*H*), 6.64 (1 H, s, Ar*H*), 5.96 (2 H, s, OCH_2O), 5.30 (1 H, dddd, *J* = 1.7, 2.8, 3.4, 7.9 Hz, ArC*H*CH_2), 5.02 (1 H, dd, *J* = 2.8, 11.7 Hz, one of ArCH_2O), 4.92 (1 H, dd, *J* = 1.7, 11.7 Hz, one of ArCH_2O), 4.19 (1 H, d, *J* = 1.8 Hz, one of C=CH_2), 4.10−4.00 (4 H, m, OCH_2CH_2O), 3.93 (1 H, d, *J* = 1.8 Hz, one of C=CH_2), 2.44 (1 H, ddd, *J* = 7.3, 9.1, 14.7 Hz, one of CH_2=C(OPh)CH_2), 2.39 (1 H, ddd, *J* = 6.8, 9.3, 14.7 Hz, one of CH_2=C(OPh)CH_2), 2.16−2.09 (2 H, m, CH_2=C(OPh)CH_2CH_2), 2.11 (1 H, dd, *J* = 7.9, 14.8 Hz, one of ArCHCH_2), 2.06 (1 H, dd, *J* = 3.4, 14.8 Hz, one of ArCHCH_2); δ_C (126 MHz; $CDCl_3$) 163.3 (C), 155.5 (C), 147.6 (C), 147.5 (C), 135.4 (C), 131.9 (C), 129.5 (2 × CH), 123.8 (CH), 120.8 (2 × CH), 110.2 (C), 102.0 (CH), 101.45 (CH), 101.39 (CH_2), 88.6 (CH_2), 80.0 (CH), 72.5 (CH_2), 65.0 (2 × CH_2), 44.0 (CH_2), 35.4 (CH_2), 28.6 (CH_2); HRMS (ESI) *m*/*z* $[M + Na]^+$ calcd for $C_{23}H_{24}O_6Na$ 419.1471; found 419.1492.

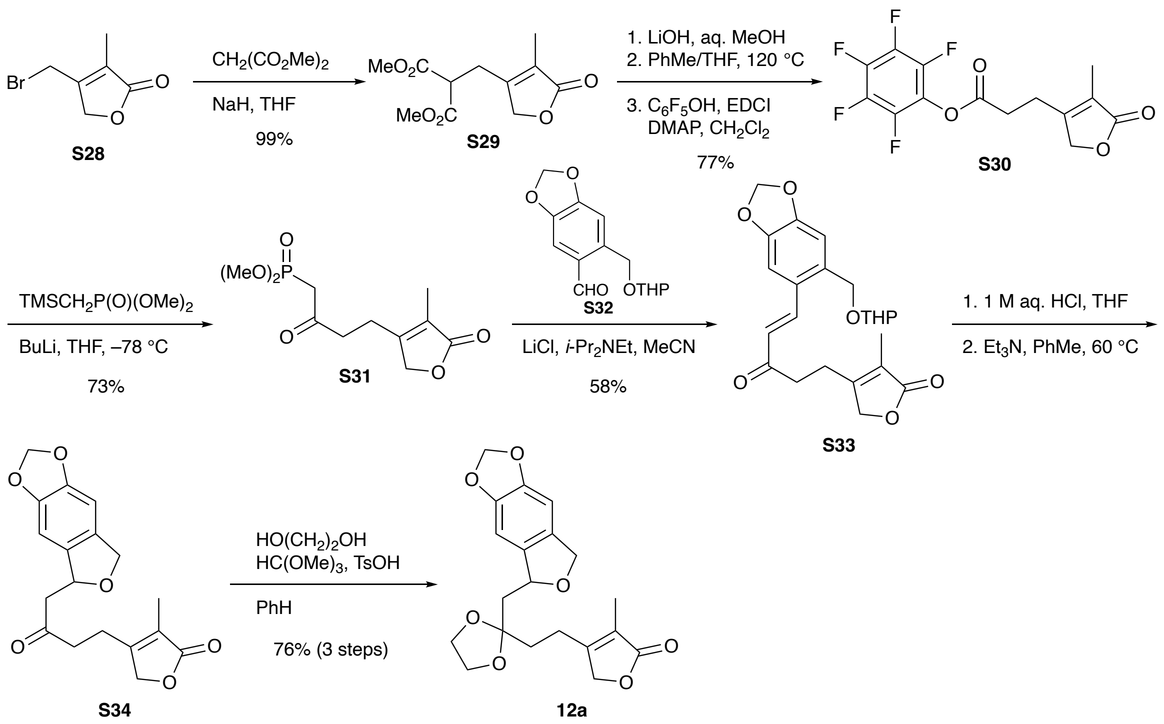

Dimethyl 2-[(4-Methyl-5-oxo-2,5-dihydrofuran-3-yl)methyl]malonate (S29). Dimethyl malonate (0.41 mL, 3.55 mmol) was added to an ice-cooled (0 °C) suspension of NaH (143 mg of a 60% dispersion in mineral oil, 3.58 mmol, washed with *n*-hexane) in THF (10 mL). After 40 min of stirring at room temperature, a solution of bromide **S28**[77] (270 mg, 1.42 mmol) in THF (2 mL plus a 0.5 mL rinse) was added, and the resulting mixture was stirred for 2 h. After cooling to 0 °C, the reaction was quenched with 1 M aqueous hydrochloric acid (4 mL), and the mixture was extracted with AcOEt (2 × 8 mL). The combined organic extracts were successively washed with water (5 mL) and brine (2 × 20 mL) and dried over anhydrous Na_2SO_4. Filtration and evaporation in vacuo furnished the crude product (550 mg, colorless oil), which was purified by column chromatography (silica gel 15 g, *n*-hexane/AcOEt 4:1 → 2:1) to give dimethyl ester **S29** (340 mg, 99%) as a colorless oil. R_f = 0.18 (*n*-hexane/AcOEt 2:1); IR (neat) ν_{max}/cm^{-1} 2957, 2868, 1748, 1680, 1437, 1337, 1283, 1248, 1211, 1157, 1084, 1034, 991; δ_H (500 MHz; $CDCl_3$) 4.66 (2 H, q, J = 1.7 Hz, OCH_2C=), 3.77 (6 H, s, 2 × CO_2CH_3), 3.58 (1 H, t, J = 7.5 Hz, $CH(CO_2CH_3)_2$), 3.00 (2 H, d, J = 7.5 Hz, $CH_2CH(CO_2CH_3)_2$), 1.86 (3 H, t, J = 1.7 Hz, CH_3C=); δ_C (126 MHz; $CDCl_3$) 174.7 (C), 168.4 (2 × C), 155.3 (C), 125.7 (C), 71.3 (CH_2), 53.0 (2 × CH_3), 49.4 (CH), 26.0 (CH_2), 8.6 (CH_3); HRMS (ESI) *m*/*z* $[M + Na]^+$ calcd for $C_{11}H_{14}O_6Na$ 265.0688; found 265.0710.

2,3,4,5,6-Pentafluorophenyl 3-(4-Methyl-5-oxo-2,5-dihydrofuran-3-yl)propionate (S30). Lith-

ium hydroxide monohydrate (241 mg, 5.73 mmol) was added to an ice-cooled (0 °C) solution of dimethyl ester **S29** (555 mg, 2.29 mmol) in MeOH/H_2O (3.1:1, 26.5 mL). After 20 h of stirring at room temperature, the reaction mixture was acidified with 1 M aqueous hydrochloric acid (30 mL) and extracted with AcOEt (4 × 100 mL), and the combined organic extracts were dried over anhydrous Na_2SO_4. Filtration and evaporation in vacuo furnished the crude dicarboxylic acid (481 mg), which was used without further purification.

The crude dicarboxylic acid (481 mg) in toluene/THF (6:1, 35 mL) was heated at 120 °C for 3.5 h. After cooling to room temperature, the solvents were evaporated in vacuo to furnish the crude product (389 mg), which was used without further purification.

To an ice-cooled (0 °C) solution of the crude carboxylic acid (389 mg) and pentafluorophenol (632 mg, 3.44 mmol) in CH_2Cl_2 (11 mL) was added 1-(3-dimethylaminopropyl)-3-ethylcarbodiimide hydrochloride (EDCI, 878 mg, 4.58 mmol), followed by DMAP (56.0 mg, 0.458 mmol). After 2 h of stirring at room temperature, the reaction was quenched with saturated aqueous NH_4Cl (30 mL), and the mixture was extracted with AcOEt (3 × 30 mL). The combined organic extracts were washed with brine (2 × 5 mL) and dried over anhydrous Na_2SO_4. Filtration and evaporation in vacuo furnished the crude product (1.02 g), which was purified by column chromatography (silica gel 30 g, *n*-hexane/AcOEt 9:1 → 4:1) to give ester **S30** (593 mg, 77%) as a white solid. R_f = 0.39 (*n*-hexane/AcOEt 2:1); mp 67.9–68.6 °C (colorless needles from *n*-hexane/AcOEt 4:1); IR (KBr) ν_{max}/cm^{-1} 2932, 2870, 1792, 1751, 1683, 1525, 1408, 1338, 1105, 1045, 1033, 1007, 993; δ_H (500 MHz; $CDCl_3$) 4.72 (2 H, q, J = 2.1 Hz, OCH_2C=), 2.96–2.92 (2 H, m, CH_2CH_2CO), 2.90–2.86 (2 H, m, CH_2CH_2CO), 1.90 (3 H, t, J = 2.1 Hz, CH_3C=); δ_C (126 MHz; $CDCl_3$) 174.8 (C), 168.1 (C), 156.3 (C), 140.9 (ddq, J_{C-F} = 12.4, 251.8, 3.8 Hz, 2 × C), 139.7 (dtt, J_{C-F} = 253.7, 3.8, 13.4 Hz, C), 137.9 (dddt, J_{C-F} = 3.8, 4.8, 252.7, 13.4 Hz, 2 × C), 125.1 (C), 124.6 (m, C), 71.1 (CH_2), 30.9 (CH_2), 21.9 (CH_2), 8.6 (CH_3); HRMS (ESI) *m*/*z* $[M + Na]^+$ calcd for $C_{14}H_9F_5O_4Na$ 359.0319; found 359.0316.

Dimethyl 4-(4-Methyl-5-oxo-2,5-dihydrofuran-3-yl)-2-oxobutylphosphonate (S31). BuLi in *n*-hexane (1.52 M, 2.47 mL, 3.75 mmol) was added to a cooled (−78 °C) solution of dimethyl (trimethylsilyl)methylphosphonate[78] (624 μL, 3.92 mmol) in THF (15 mL). After 30 min of stirring, a solution of pentafluorophenyl ester **S30** (573 mg, 1.70 mmol) in THF (7 mL plus a 3 mL rinse) was

added. After 10 min of stirring, the reaction was quenched with saturated aqueous NH_4Cl (20 mL), and the mixture was extracted with AcOEt (3 × 50 mL). The combined organic extracts were washed with brine (2 × 10 mL) and dried over anhydrous Na_2SO_4. Filtration and evaporation in vacuo furnished the crude product (1.33 g), which was purified by column chromatography (silica gel 35 g, CH_2Cl_2/acetone 9:1 → 4:1 → 2:1) to give phosphonate **S31** (343 mg, 73%) as a colorless oil. R_f = 0.37 (CH_2Cl_2/AcOEt 2:1); IR (neat) ν_{max}/cm^{-1} 2959, 2857, 1736, 1719, 1676, 1449, 1406, 1341, 1252, 1030; δ_H (500 MHz; $CDCl_3$) 4.66 (2 H, q, J = 1.1 Hz, OCH_2C=), 3.78 (6 H, d, J_{H-P} = 10.9 Hz, (CH_3O)$_2$P(O)), 3.11 (2 H, d, J_{H-P} = 22.9 Hz, P(O)CH_2), 2.90 (2 H, t, J = 6.8 Hz, CH_2CH$_2$CO), 2.69 (2 H, J = 6.8 Hz, CH$_2$CH_2CO), 1.86 (3 H, t, J = 1.1 Hz, CH_3C=); δ_C (126 MHz; $CDCl_3$) 199.8 (d, J_{C-P} = 5.7 Hz, C), 175.2 (C), 158.4 (C), 123.9 (C), 71.5 (CH_2), 53.2 (d, J_{C-P} = 6.7 Hz, 2 × CH_3), 41.4 (d, J_{C-P} = 127.8 Hz, CH_2), 41.1 (CH_2), 20.5 (CH_2), 8.6 (CH_3); HRMS (ESI) *m/z* $[M + Na]^+$ calcd for $C_{11}H_{17}O_6PNa$ 299.0660; found 299.0659.

1-{4,5-Methylenedioxy-2-[(tetrahydropyran-2-yl)oxymethyl]phenyl}-5-(4-methyl-5-oxo-2,5-dihydrofuran-3-yl)pent-1-en-3-one (S33). A solution of aldehyde **S32**[79] (532 mg, 2.01 mmol) in MeCN (3 mL plus a 2 mL rinse) was added to a mixture of phosphonate **S31** (315 mg, 1.14 mmol), LiCl (169 mg, 3.99 mmol) and *i*-Pr$_2$NEt (694 μL, 3.99 mmol) in MeCN (6 mL). After 72 h of stirring, the reaction was quenched with saturated aqueous NH_4Cl (12 mL), and the mixture was extracted with AcOEt (3 × 30 mL). The combined organic extracts were washed with brine (2 × 5 mL) and dried over anhydrous Na_2SO_4. Filtration and evaporation in vacuo furnished the crude product (730 mg), which was purified by column chromatography (silica gel 25 g, *n*-hexane/AcOEt 4:1 → 2:1) to give enone **S33** (273 mg, 58%) as a white solid. R_f = 0.73 (*n*-hexane/AcOEt 1:2); mp 148.2−149.0 °C (colorless needles from AcOEt); IR (KBr) ν_{max}/cm^{-1} 2941, 2837, 1749, 1645, 1506, 1487, 1346, 1331, 1273, 1251, 1190, 1126, 1037; δ_H (500 MHz; $CDCl_3$) 7.95 (1 H, d, J = 15.9 Hz, CH=CHCO), 7.10 (1 H, s, ArH), 6.93 (1 H, s, ArH), 6.55 (1 H, d, J = 15.9 Hz, CH=CHCO), 6.02 (2 H, s, OCH_2O), 4.86 (1 H, d, J = 12.0 Hz, one of ArCH_2OTHP), 4.70−4.66 (3 H, m, OCH_2C=, THP ether OCHO), 4.53 (1 H, d, J = 12.0 Hz, one of ArCH_2OTHP), 3.89 (1 H, ddd, J = 3.1, 8.2, 11.0 Hz, one of THP ether OCH_2), 3.56 (1 H, ddd, J = 3.6, 5.1, 11.0 Hz, one of THP ether OCH_2), 2.91 (2 H, t, J = 7.0 Hz, CH_2CH$_2$CO), 2.79 (2 H, t, J = 7.0 Hz, CH$_2$CH_2CO), 1.87 (3 H, t, J = 1.9 Hz, CH_3C=),

1.83 (1 H, m, one of THP ether CH_2), 1.73 (1 H, m, one of THP ether CH_2), 1.65−1.48 (4 H, m, four of THP ether (CH_2)$_3$); δ_C (126 MHz; $CDCl_3$) 197.7 (C), 175.4 (C), 159.3 (C), 150.0 (C), 148.1 (C), 140.4 (CH), 133.6 (C), 127.3 (C), 125.0 (CH), 123.8 (C), 110.4 (CH), 105.9 (CH), 101.9 (CH_2), 98.2 (CH), 71.8 (CH_2), 66.5 (CH_2), 62.9 (CH_2), 37.9 (CH_2), 30.8 (CH_2), 25.5 (CH_2), 21.3 (CH_2), 19.7 (CH_2), 8.8 (CH_3); HRMS (ESI) *m/z* $[M + Na]^+$ calcd for $C_{23}H_{26}O_7Na$ 437.1576; found 437.1565.

1-{2-[2-(4-methyl-5-oxo-2,5-dihydrofuran-3-yl)ethyl]-1,3-dioxolan-2-yl}methyl-1,3-dihydro-5,6-methylenedioxyisobenzofuran (12a). A 1 M aqueous hydrochloric acid (5 mL) was added to a solution of enone **S33** (133 mg, 0.321 mmol) in THF (20 mL). After 14 h of stirring, the reaction mixture was neutralized with saturated aqueous $NaHCO_3$ (20 mL) and extracted with AcOEt (3 × 60 mL). The combined organic extracts were washed with brine (2 × 20 mL) and dried over anhydrous Na_2SO_4. Filtration and evaporation in vacuo furnished the crude product (134 mg), which was used without further purification.

Et_3N (534 μL, 3.85 mmol) was added to a solution of the crude alcohol (134 mg) in toluene (14 mL), and the resulting solution was heated at 60 °C for 24 h. After cooling to room temperature, the volatile elements were removed in vacuo to furnish the crude product (127 mg), which was used without further purification.

p-Toluenesulfonic acid (6.10 mg, 32.1 μmol) was added to a mixture of the crude ketone **S34** (127 mg), ethylene glycol (72 μL 1.28 mmol) and trimethyl orthoformate (176 μL, 1.60 mmol) in benzene (1.1 mL). After 17 h of stirring, the reaction was quenched with saturated aqueous $NaHCO_3$ (10 mL), and the mixture was extracted with AcOEt (3 × 15 mL). The combined organic extracts were washed with brine (2 × 5 mL) and dried over anhydrous Na_2SO_4. Filtration and evaporation in vacuo furnished the crude product (155 mg), which was purified by column chromatography (silica gel 4.5 g, *n*-hexane/AcOEt 9:1 → 4:1 → 2:1) to give acetal **12a** (91.1 mg, 76%) as a white amorphous solid. R_f = 0.56 (*n*-hexane/AcOEt 1:2); IR (KBr) ν_{max}/cm^{-1} 2927, 2897, 1734, 1676, 1638, 1475, 1336, 1271, 1153, 1080, 1037; δ_H (500 MHz; C_6D_6) 6.51 (1 H, s, Ar*H*), 6.33 (1 H, s, Ar*H*), 5.35 (1 H, d, *J* = 1.3 Hz, one of OCH_2O), 5.34 (1 H, d, *J* = 1.3 Hz, one of OCH_2O), 5.33 (1 H, dddd, *J* = 2.2, 2.5, 2.9, 8.7 Hz, ArC*H*CH_2), 4.70 (1 H, dd, *J* = 2.9, 11.7 Hz, one of ArCH_2O), 4.64 (1 H, dd, *J* = 2.2, 11.7 Hz, one of ArCH_2O), 4.04 (2 H, q, *J* = 2.0 Hz, OCH_2C=), 3.52−3.38 (4 H, m,

OCH_2CH_2O), 2.17 (1 H, ddd, J = 6.2, 10.1, 14.5 Hz, one of =C$CH_2$$CH_2$), 2.12 (1 H, ddd, J = 6.4, 9.8, 14.5 Hz, one of =C$CH_2$$CH_2$), 2.01 (1 H, dd, J = 8.7, 14.8 Hz, one of ArCHCH_2), 1.88 (1 H, dd, J = 2.5, 14.8 Hz, one of ArCHCH_2), 1.81 (1 H, ddd, J = 6.2, 9.8, 14.1 Hz, one of =CCH_2CH_2), 1.75 (1 H, ddd, J = 6.4, 10.1, 14.1 Hz, one of =CCH_2CH_2), 1.66 (3 H, t, J = 2.0 Hz, CH_3C=); δ_C (126 MHz; $CDCl_3$) 175.6 (C), 160.5 (C), 147.7 (C), 147.6 (C), 134.8 (C), 131.7 (C), 122.7 (C), 109.8 (C), 101.8 (CH), 101.6 (CH), 101.5 (CH_2), 80.1 (CH), 72.5 (CH_2), 71.5 (CH_2), 65.1 (CH_2), 65.0 (CH_2), 43.7 (CH_2), 35.1 (CH_2), 21.5 (CH_2), 8.5 (CH_3); HRMS (ESI) m/z $[M + Na]^+$ calcd for $C_{20}H_{22}O_7Na$ 397.1263; found 397.1291.

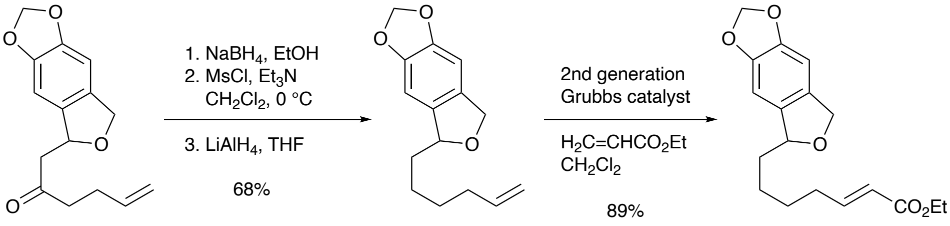

1-(Hex-5-enyl)-1,3-dihydro-5,6-methylenedioxyisobenzofuran (S35). Ketone **S6** (500 mg, 1.92 mmol) was added to an ice-cooled (0 °C) solution of $NaBH_4$ (37.3 mg, 0.960 mmol) in EtOH (10 mL). After 4 h of stirring at room temperature, half-saturated brine (10 mL) was added, and the mixture was extracted with AcOEt (2 × 90 mL). The combined organic extracts were washed with brine (2 × 30 mL) and dried over anhydrous Na_2SO_4. Filtration and evaporation in vacuo furnished the crude product (509 mg), which was used without further purification.

MsCl (0.23 mL, 2.88 mmol) was added to an ice-cooled (0 °C) mixture of the crude alcohol (509 mg) and Et_3N (0.80 mL, 5.76 mmol) in CH_2Cl_2 (10 mL). After 1 h of stirring, the volatile elements were removed in vacuo, and the residue was partitioned between AcOEt (30 mL) and saturated aqueous $NaHCO_3$ (15 mL). The aqueous layer was extracted with AcOEt (2 × 80 mL), and the combined organic extracts were washed with brine (2 × 30 mL) and dried over anhydrous Na_2SO_4. Filtration and evaporation in vacuo furnished the crude product (665 mg), which was used without further purification.

A solution of the crude mesylate (665 mg) in THF (2 mL plus 2 × 1 mL rinse) was added to an ice-cooled (0 °C) suspension of $LiAlH_4$ (303 mg, 7.98 mmol) in THF (3 mL). After 25 h of stirring at room temperature, the reaction was quenched with H_2O (0.3 mL), and 15% aqueous NaOH (0.3 mL) and H_2O (0.9 mL) were added. The mixture was filtered through a Celite pad, and the filtrate

was evaporated in vacuo. Purification of the residue (654 mg) by column chromatography (silica gel 15 g, *n*-hexane/AcOEt 40:1) afforded alkene **S35** (321 mg, 68% for three steps) as a colorless oil. R_f = 0.42 (*n*-hexane/AcOEt 10:1); IR (neat) ν_{max}/cm^{-1} 2930, 2855, 1476, 1342, 1269, 1155, 1042, 941; δ_H (500 MHz; $CDCl_3$) 6.64 (1 H, s, Ar*H*), 6.59 (1 H, s, Ar*H*), 5.97 (2 H, s, OCH_2O), 5.80 (1 H, ddt, *J* = 10.1, 16.8, 6.8 Hz, C*H*=CH_2), 5.13 (1 H, m, ArC*H*CH_2), 5.01 (1 H, dd, *J* = 2.9, 11.7 Hz, one of ArCH_2O), 4.99 (1 H, dq, *J* = 16.8, 1.1 Hz, one of CH=CH_2), 4.95 (1 H, dd, *J* = 1.6, 11.7 Hz, one of ArCH_2O), 4.93 (1 H, dq, *J* = 10.1, 1.1 Hz, one of CH=CH_2), 2.06 (2 H, tq, *J* = 1.1, 6.8 Hz, $CH_2CH_2CH{=}CH_2$), 1.80 (1 H, m, one of ArCHCH_2), 1.65 (1 H, m, one of ArCHCH_2), 1.47−1.40 (4 H, m, ArCHCH_2CH_2, $CH_2CH_2CH{=}CH_2$); δ_C (126 MHz; $CDCl_3$) 147.5 (C), 147.4 (C), 138.9 (CH), 135.1 (C), 132.0 (C), 114.3 (CH_2), 101.8 (CH), 101.5 (CH), 101.4 (CH_2), 83.9 (CH), 72.5 (CH_2), 36.2 (CH_2), 33.7 (CH_2), 29.0 (CH_2), 24.6 (CH_2); HRMS (EI) *m*/*z* $[M]^+$ calcd for $C_{15}H_{18}O_3$ 246.1256; found 246.1270.

Ethyl (2*E*)-7-(1,3-Dihydro-5,6-methylenedioxyisobenzofuran-1-yl)hept-2-enoate (13a). The second-generation Grubbs catalyst (34.9 mg, 41.1 μmol) was added to a mixture of alkene **S35** (202 mg, 0.821 mmol) and ethyl acrylate (0.90 mL, 8.21 mmol) in CH_2Cl_2 (8.2 mL). After 51 h of stirring, the volatile elements were removed in vacuo, and the residue (480 mg) was purified by column chromatography (silica gel 15 g, *n*-hexane/AcOEt 10:1) to give enoate **13a** (234 mg, 89%) as a colorless oil. R_f = 0.47 (n-hexane/AcOEt 4:1); IR (neat) ν_{max}/cm^{-1} 2934, 2857, 1717, 1653, 1476, 1342, 1267, 1182, 1155, 1040, 939; δ_H (500 MHz; $CDCl_3$) 6.95 (1 H, dt, *J* = 15.7, 7.0 Hz, C*H*=$CHCO_2Et$), 6.64 (1 H, s, Ar*H*), 6.58 (1 H, s, Ar*H*), 5.97 (2 H, s, OCH_2O), 5.80 (1 H, dt, *J* = 15.7, 1.5 Hz, CH=C*H*CO_2Et), 5.13 (1 H, m, ArC*H*CH_2), 5.00 (1 H, dd, *J* = 2.9, 11.6 Hz, one of ArCH_2O), 4.94 (1 H, dd, *J* = 1.6, 11.6 Hz, one of ArCH_2O), 4.18 (2 H, q, *J* = 7.1 Hz, OCH_2CH_3), 2.20 (2 H, dq, *J* = 1.5, 7.0 Hz, $CH_2CH_2CH{=}CH$), 1.81 (1 H, m, one of ArCHCH_2), 1.64 (1 H, m, one of ArCHCH_2), 1.54−1.40 (4 H, m, ArCHCH_2CH_2, $CH_2CH_2CH{=}CH$), 1.28 (3 H, t, *J* = 7.1 Hz, OCH_2CH_3); δ_C (126 MHz; $CDCl_3$) 166.7 (C), 149.1 (CH), 147.6 (C), 147.5 (C), 134.8 (C), 132.0 (C), 121.4 (CH), 101.7 (CH), 101.6 (CH), 101.4 (CH_2), 83.7 (CH), 72.6 (CH_2), 60.1 (CH_2), 36.1 (CH_2), 32.2 (CH_2), 28.1 (CH_2), 24.6 (CH_2), 14.3 (CH_3); HRMS (ESI) *m*/*z* $[M + Na]^+$ calcd for $C_{18}H_{22}O_5Na$ 341.1359; found 341.1343.

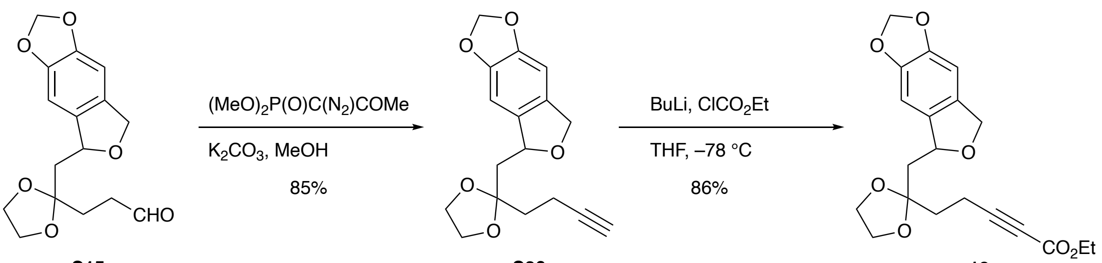

S15 S36 18

1-[2-(But-3-ynyl)-1,3-dioxolan-2-yl]methyl-1,3-dihydro-5,6-methylenedioxyisobenzofuran (S36). A solution of Ohira–Bestmann reagent[80] (167 mg, 0.872 mmol) in MeOH (1.8 mL) was added to a cooled (5 °C) mixture of aldehyde **S15** (178 mg, 0.581 mmol) and K_2CO_3 (185 mg, 1.34 mmol) in MeOH (4.0 mL). After 4 h of stirring at room temperature, the reaction was quenched with H_2O (5 mL), and the resulting mixture was extracted with AcOEt (2 × 20 mL). The combined organic extracts were washed with brine (30 mL) and dried over Na_2SO_4. Filtration and evaporation in vacuo furnished the crude product (196 mg, yellow solid), which was purified by column chromatography (silica gel 4 g, *n*-hexane/AcOEt 4:1) to give alkyne **S36** (149 mg, 85%) as a white solid. R_f = 0.52 (*n*-hexane/AcOEt 2:1); mp 86.0–87.0 °C (colorless needles from *n*-hexane); IR (KBr) ν_{max}/cm^{-1} 3418, 3285, 2918, 2850, 1614, 1506, 1476, 1350, 1287, 1265, 1148, 1040, 937, 868, 679; δ_H (500 MHz; $CDCl_3$) 6.64 (1 H, s, Ar*H*), 6.63 (1 H, s, Ar*H*), 5.97 (2 H, s, OCH_2O), 5.27 (1 H, m, ArC*H*CH_2), 5.02 (1 H, dd, *J* = 2.9, 11.8 Hz, one of ArCH_2O), 4.93 (1 H, dd, *J* = 1.9, 11.8 Hz, one of ArCH_2O), 4.09–3.98 (4 H, m, OCH_2CH_2O), 2.35 (1 H, dddd, *J* = 2.6, 7.0, 8.9, 16.7 Hz, one of CH_2C≡CH), 2.30 (1 H, dddd, *J* = 2.6, 6.2, 8.9, 16.7 Hz, one of CH_2C≡CH), 2.10 (1 H, ddd, *J* = 6.2, 8.9, 14.4 Hz, one of C$H_2$$CH_2$C≡CH), 2.08 (1 H, ddd, *J* = 7.0, 8.9, 14.4 Hz, one of C$H_2$$CH_2$C≡CH), 2.05–2.00 (2 H, m, ArCHCH_2), 1.94 (1 H, t, *J* = 2.6 Hz, CH_2C≡C*H*); δ_C (126 MHz; $CDCl_3$) 147.6 (C), 147.5 (C), 135.2 (C), 131.9 (C), 109.7 (C), 101.9 (CH), 101.5 (CH), 101.4 (CH_2), 84.5 (C), 79.9 (CH), 72.4 (CH_2), 67.9 (CH), 65.1 (CH_2), 65.0 (CH_2), 43.9 (CH_2), 36.7 (CH_2), 13.1 (CH_2); HRMS (ESI) *m/z* $[M + Na]^+$ calcd for $C_{17}H_{18}O_5Na$ 325.1052; found 325.1043.

Ethyl 5-[2-(1,3-Dihydro-5,6-methylenedioxyisobenzofuran-1-yl)methyl-1,3-dioxoran-2-yl]pent-2-ynoate (18). BuLi in *n*-hexane (1.53 M, 0.20 mL, 0.306 mmol) was added to a cooled (−78 °C) solution of alkyne **S36** (76.5 mg, 0.253 mmol) in THF (1.0 mL). After 30 min of stirring, $ClCO_2Et$ (30 μL, 0.304 mmol) was added, and the mixture was stirred for 3 h. The reaction was quenched

with saturated aqueous NH_4Cl (5 mL), and the resulting mixture was extracted with AcOEt (2 × 10 mL). The combined organic extracts were washed with brine (10 mL) and dried over Na_2SO_4. Filtration and evaporation in vacuo furnished the crude product (99.0 mg, yellow oil), which was purified by column chromatography (silica gel 3 g, *n*-hexane/AcOEt 5:1) to give ester **18** (81.6 mg, 86%) as a pale yellow oil. R_f = 0.49 (*n*-hexane/AcOEt 2:1); IR (neat) ν_{max}/cm^{-1} 3080, 2938, 2864, 1714, 1614, 1416, 1371, 1325, 1221, 1163, 1120, 1069, 1016, 924, 833, 826; δ_H (500 MHz; $CDCl_3$) 6.63 (1 H, s, Ar*H*), 6.60 (1 H, s, Ar*H*), 5.96 (2 H, s, OCH_2O), 5.24 (1 H, m, ArC*H*CH_2), 5.01 (1 H, dd, *J* = 2.8, 11.7 Hz, one of $ArCH_2O$), 4.92 (1 H, dd, *J* = 1.7, 11.7 Hz, one of $ArCH_2O$), 4.20 (2 H, q, *J* = 7.1 Hz, $CO_2CH_2CH_3$), 4.08−3.96 (4 H, m, OCH_2CH_2O), 2.49 (1 H, ddd, *J* = 6.7, 9.4, 17.1 Hz, one of $CH_2C{\equiv}CCO_2Et$), 2.44 (1 H, ddd, *J* = 6.0, 9.4, 17.1 Hz, one of $CH_2C{\equiv}CCO_2Et$), 2.14 (1 H, ddd, *J* = 6.0, 9.4, 14.2 Hz, one of $CH_2CH_2C{\equiv}CCO_2Et$), 2.10 (1 H, ddd, *J* = 6.7, 9.4, 14.2 Hz, one of $CH_2CH_2C{\equiv}CCO_2Et$), 2.02−1.95 (2 H, m, $ArCHCH_2$), 1.29 (3 H, t, *J* = 7.1 Hz, $CO_2CH_2CH_3$); δ_C (126 MHz; $CDCl_3$) 153.8 (C), 147.7 (C), 147.5 (C), 135.0 (C), 131.8 (C), 109.4 (C), 101.9 (CH), 101.5 (CH), 101.4 (CH_2), 89.3 (C), 79.9 (CH), 72.8 (C), 72.5 (CH_2), 65.1 (CH_2), 65.0 (CH_2), 61.8 (CH_2), 43.9 (CH_2), 35.5 (CH_2), 14.0 (CH_3), 13.3 (CH_2); HRMS (ESI) *m/z* $[M + Na]^+$ calcd for $C_{20}H_{22}O_7Na$ 397.1263; found 397.1265.

2-2. Tandem Oxidation/Intramolecular Diels−Alder Reaction

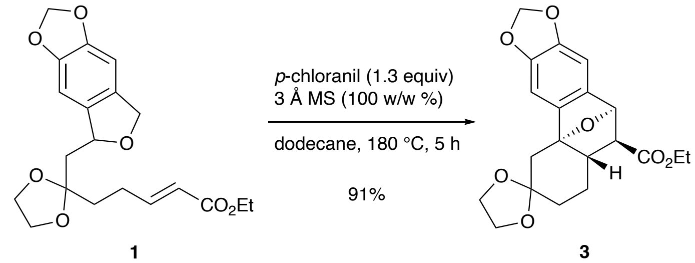

1 3

Typical Procedure for Tandem Oxidation/Intramolecular Diels−Alder Reaction with Phthalans: Ethyl (4a*S,9*R**,10*S**,10a*S**)-6,7-Methylenedioxy-[1,2,3,4,4a,9,10,10a-octahydro-4a,9-epoxyphenanthrene-3-spiro-2'-(1',3'-dioxolane)]-10-carboxylate (3).** *p*-Chloranil (42.3 mg, 0.173 mmol) was added to a mixture of phthalan **1** (50.3 mg, 0.133 mmol) and 3 Å MS (50.8 mg) in *n*-dodecane (13 mL), and the mixture was heated at 180 °C for 5 h. After cooling, the reaction was quenched with saturated aqueous $NaHCO_3$/0.5 M aqueous $Na_2S_2O_3$ (1:1, 20 mL). The resulting mixture was filtered through a Celite pad, and the filtrate was extracted with AcOEt (3 × 60 mL).

The combined organic extracts were successively washed with saturated aqueous $NaHCO_3$ (40 mL) and brine (2 × 50 mL), and dried over anhydrous Na_2SO_4. Filtration and evaporation in vacuo furnished the crude product, which was purified by column chromatography (silica gel 10 g, *n*-hexane → *n*-hexane/AcOEt 2:1) to give hydrophenanthrene **3** (45.5 mg, 91%) as a pale orange solid. R_f = 0.45 (*n*-hexane/AcOEt 1:1); mp 131.0−134.0 °C (colorless needles from *n*-hexane/CH_2Cl_2 10:1); IR (KBr) ν_{max}/cm^{-1} 2956, 2887, 1732, 1471, 1381, 1309, 1288, 1267, 1244, 1213, 1035, 1008, 731; δ_H (500 MHz; C_6D_6) 6.66 (1 H, s, Ar*H*), 6.42 (1 H, s, Ar*H*), 5.32 (1 H, d, *J* = 1.4 Hz, one of OC*H*$_2$O), 5.29 (1 H, d, *J* = 5.3 Hz, OC*H*CHCO$_2$Et), 5.25 (1 H, d, *J* = 1.4 Hz, one of OC*H*$_2$O), 3.73 (2 H, q, *J* = 7.2 Hz, OC*H*$_2$CH$_3$), 3.57−3.45 (4 H, m, OC*H*$_2$C*H*$_2$O), 2.85 (1 H, dd, *J* = 3.2, 5.3 Hz, C*H*CO$_2$Et), 2.49 (1 H, dd, *J* = 2.2, 15.2 Hz, one of ArCC*H*$_2$), 2.41 (1 H, d, *J* = 15.2 Hz, one of ArCC*H*$_2$), 2.16 (1 H, ddd, *J* = 3.2, 6.3, 11.6 Hz, OCC*H*CHCO$_2$Et), 1.91 (1 H, dddd, *J* = 3.2, 3.9, 6.3, 13.4 Hz, one of C*H*$_2$CHCHCO$_2$Et), 1.87 (1 H, dddd, *J* = 3.3, 11.6, 13.4, 13.5 Hz, one of C*H*$_2$CHCHCO$_2$Et), 1.77 (1 H, dddd, *J* = 2.2, 3.2, 3.3, 13.4 Hz, one of C*H*$_2$CH$_2$CHCHCO$_2$Et), 1.59 (1 H, ddd, *J* = 3.9, 13.4, 13.5 Hz, one of C*H*$_2$CH$_2$CHCHCO$_2$Et), 0.83 (3 H, t, *J* = 7.2 Hz, OCH$_2$C*H*$_3$); δ_C (126 MHz; C_6D_6) 170.6 (C), 147.4 (C), 146.8 (C), 142.9 (C), 137.7 (C), 108.4 (C), 103.0 (CH), 101.3 (CH_2), 101.0 (CH), 88.7 (C), 80.2 (CH), 64.9 (CH_2), 64.3 (CH_2), 60.4 (CH_2), 55.7 (CH), 41.8 (CH), 36.1 (CH_2), 34.6 (CH_2), 28.9 (CH_2), 14.4 (CH_3); HRMS (ESI) *m*/*z* $[M + Na]^+$ calcd for $C_{20}H_{22}O_7Na$ 397.1258; found 397.1228.

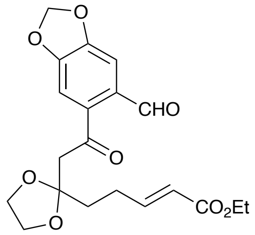

4

Data for ethyl (2*E*)-5-{2-[2-(2-formyl-4,5-methylenedioxyphenyl)-2-oxoethyl]-1,3-dioxolan-2-yl}-pent-2-enoate (**4**). Pale yellow oil; R_f = 0.13 (*n*-hexane/AcOEt 3:1); IR (neat) ν_{max}/cm^{-1} 2957, 2924, 2855, 1719, 1655, 1609, 1508, 1474, 1369, 1271, 1180, 1155, 1094, 1078, 1036; δ_H (500 MHz; $CDCl_3$) 9.96 (1 H, s, C*H*O), 7.37 (1 H, s, Ar*H*), 7.16 (1 H, s, Ar*H*), 6.96 (1 H, dt, *J* = 15.6, 6.9 Hz, C*H*=CHCO$_2$Et), 6.14 (2 H, s, OC*H*$_2$O), 5.82 (1 H, dt, *J* = 15.6, 1.5 Hz, CH=C*H*CO$_2$Et), 4.18 (2 H, q, *J* = 7.1 Hz, OC*H*$_2$CH$_3$), 3.96−3.88 (4 H, m, OC*H*$_2$C*H*$_2$O), 3.23 (2 H, s, ArCOC*H*$_2$), 2.33 (2 H, ddt, *J* = 1.5, 6.9, 8.0 Hz, CH$_2$C*H*$_2$CH=CH), 1.97 (2 H, t, *J* = 8.0 Hz, C*H*$_2$CH$_2$CH=CH), 1.28 (3 H, t, *J* = 7.1

Hz, OCH_2CH_3); δ_C (126 MHz; $CDCl_3$) 198.3 (C), 190.1 (C), 166.6 (C), 151.2 (C), 150.2 (C), 148.1 (CH), 138.6 (C), 132.4 (C), 121.5 (CH), 109.5 (C), 108.5 (CH), 108.4 (CH), 102.7 (CH_2), 65.2 (2 × CH_2), 60.2 (CH_2), 48.4 (CH_2), 36.3 (CH_2), 26.3 (CH_2), 14.3 (CH_3); HRMS (ESI) *m*/*z* $[M + Na]^+$ calcd for $C_{20}H_{22}O_8Na$ 413.1207; found 413.1206.

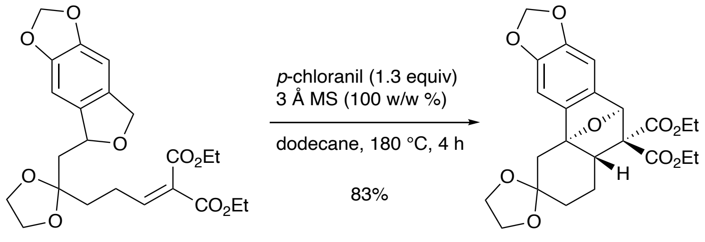

Diethyl (4a*S,9*S**,10a*S**)-6,7-Methylenedioxy-[1,2,3,4,4a,9,10,10a-octahydro-4a,9-epoxyphenanthrene-3-spiro-2'-(1',3'-dioxolane)]-10,10-dicarboxylate (5).** The tandem oxidation/intramolecular Diels−Alder reaction was performed according to the typical procedure (13 mL *n*-dodecane, 180 °C, 4 h) employing phthalan **5a** (59.6 mg, 0.133 mmol), *p*-chloranil (43.3 mg, 0.173 mmol) and 3 Å MS (59.9 mg). Purification by column chromatography (silica gel 8 g, *n*-hexane → *n*-hexane/AcOEt 2:1) afforded cycloadduct **5** (49.4 mg, 83%) as a white solid. R_f = 0.38 (*n*-hexane/AcOEt 1:1); mp 205.0−206.0 °C (colorless needles from *n*-hexane/CH_2Cl_2 2:1); IR (KBr) ν_{max}/cm^{-1} 1728, 1472, 1294, 1252, 1198, 1144, 1030, 939; δ_H (500 MHz; $CDCl_3$) 6.64 (1 H, s, Ar*H*), 6.63 (1 H, s, Ar*H*), 5.95 (1 H, d, *J* = 1.3 Hz, one of OCH_2O), 5.93 (1 H, d, *J* = 1.3 Hz, one of OCH_2O), 5.67 (1 H, s, $ArCHC(CO_2Et)_2$), 4.29 (1 H, dq, *J* = 10.8, 7.1 Hz, one of OCH_2CH_3), 4.24 (1 H, dq, *J* = 10.8, 7.1 Hz, one of OCH_2CH_3), 4.08−3.90 (5 H, m, one of OCH_2CH_3, OCH_2CH_2O), 3.87 (1 H, dq, *J* = 10.8, 7.1 Hz, one of OCH_2CH_3), 2.56 (1 H, dd, *J* = 5.7, 12.0 Hz, $CH_2CHC(CO_2Et)_2$), 2.49 (1 H, d, *J* = 15.5 Hz, one of $ArCCH_2$), 2.38 (1 H, dd, *J* = 2.3, 15.5 Hz, one of $ArCCH_2$), 1.94 (1 H, ddt, *J* = 5.7, 12.6, 2.9 Hz, one of $CH_2CHC(CO_2Et)_2$), 1.86 (1 H, ddt, *J* = 2.3, 13.2, 2.9 Hz, one of $CH_2CH_2CHC(CO_2Et)_2$), 1.71 (1 H, ddd, *J* = 2.9, 13.2, 13.8 Hz, one of $CH_2CH_2CHC(CO_2Et)_2$), 1.63 (1 H, dddd, *J* = 2.9, 12.0, 12.6, 13.8 Hz, one of $CH_2CHC(CO_2Et)_2$), 1.27 (3 H, t, *J* = 7.1 Hz, OCH_2CH_3), 1.19 (3 H, t, *J* = 7.1 Hz, OCH_2CH_3); δ_C (126 MHz; $CDCl_3$) 169.3 (C), 167.9 (C), 147.2 (C), 146.6 (C), 141.6 (C), 135.7 (C), 107.3 (C), 102.3 (CH), 101.4 (CH_2), 101.1 (CH), 88.1 (C), 82.6 (CH), 67.8 (C), 65.1 (CH_2), 64.3 (CH_2), 61.551 (CH_2), 61.549 (CH_2), 44.4 (CH), 35.9 (CH_2), 33.4 (CH_2), 23.9 (CH_2), 14.2 (CH_3), 14.0 (CH_3); HRMS (ESI) *m*/*z* $[M + Na]^+$ calcd for $C_{23}H_{26}O_9Na$ 469.1469; found 469.1460.

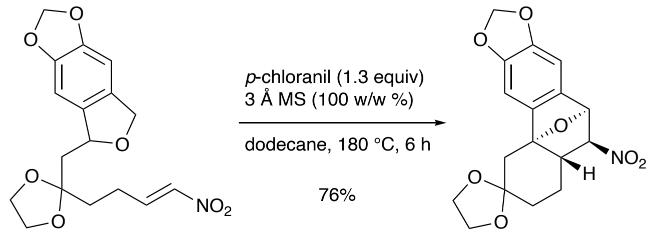

(4a*S,9*S**,10*S**,10a*S**)-6,7-Methylenedioxy-10-nitro-1,2,3,4,4a,9,10,10a-octahydro-4a,9-epoxy-phenanthrene-3-spiro-2'-(1',3'-dioxolane) (6).** The tandem oxidation/intramolecular Diels−Alder reaction was performed according to the typical procedure (13 mL *n*-dodecane, 180 °C, 6 h) employing phthalan **6a** (46.6 mg, 0.133 mmol), *p*-chloranil (43.2 mg, 0.173 mmol) and 3 Å MS (47.3 mg). Purification by column chromatography (silica gel 8 g, *n*-hexane → *n*-hexane/AcOEt 1:1) afforded cycloadduct **6** (35.2 mg, 76%) as a pale brown solid. R_f = 0.40 (*n*-hexane/AcOEt 1:1); mp 210.0−212.0 °C (pale brown needles from *n*-hexane/CH_2Cl_2 2:1); IR (KBr) ν_{max}/cm^{-1} 2895, 1531, 1474, 1385, 1292, 1267, 1140, 1128, 1032, 1009, 989, 934; δ_H (500 MHz; $CDCl_3$) 6.75 (1 H, s, Ar*H*), 6.66 (1 H, s, Ar*H*), 5.99 (1 H, d, *J* = 1.4 Hz, one of OCH_2O), 5.94 (1 H, d, *J* = 1.4 Hz, one of OCH_2O), 5.63 (1 H, d, *J* = 5.2 Hz, ArC*H*CHNO$_2$), 4.85 (1 H, dd, *J* = 2.4, 5.2 Hz, C*H*NO$_2$), 4.08−3.96 (4 H, m, OCH_2CH_2O), 2.53 (1 H, d, *J* = 15.2 Hz, one of ArCCH_2), 2.39 (1 H, dd, *J* = 3.0, 15.2 Hz, one of ArCCH_2), 2.38 (1 H, ddd, *J* = 2.4, 6.3, 12.9 Hz, CH$_2$C*H*CHNO$_2$), 2.21 (1 H, ddt, *J* = 6.3, 12.9, 3.0 Hz, one of CH_2CHCHNO$_2$), 1.90 (1 H, dq, *J* = 12.9, 3.0 Hz, one of CH_2CH$_2$CHCHNO$_2$), 1.85 (1 H, dq, *J* = 3.0, 12.9 Hz, one of CH_2CHCHNO$_2$), 1.72 (1 H, dt, *J* = 3.0, 12.9 Hz, one of CH_2CH$_2$CHCHNO$_2$); δ_C (126 MHz; $CDCl_3$) 147.9 (C), 147.2 (C), 141.0 (C), 133.0 (C), 107.2 (C), 104.0 (CH), 101.6 (CH_2), 100.3 (CH), 91.0 (CH), 89.1 (C), 79.9 (CH), 65.2 (CH_2), 64.4 (CH_2), 42.5 (CH), 35.7 (CH_2), 33.2 (CH_2), 27.0 (CH_2); HRMS (ESI) *m/z* $[M + Na]^+$ calcd for $C_{17}H_{17}NO_7Na$ 370.0897; found 370.0883.

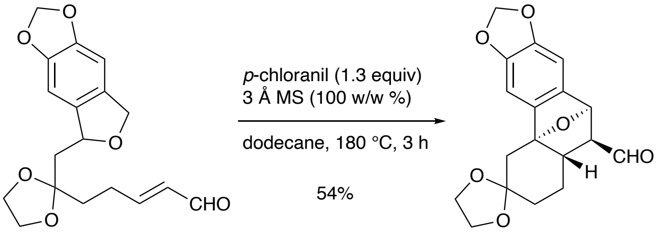

(4a*S,9*R**,10*S**,10a*S**)-6,7-Methylenedioxy-[1,2,3,4,4a,9,10,10a-octahydro-4a,9-epoxyphenanthrene-3-spiro-2'-(1',3'-dioxolane)]-10-carbaldehyde (7).** The tandem oxidation/intramolecular Diels−Alder reaction was performed according to the typical procedure (13 mL *n*-dodecane, 180 °C,

3 h) employing phthalan **7a** (44.2 mg, 0.133 mmol), *p*-chloranil (42.7 mg, 0.173 mmol) and 3 Å MS (44.6 mg). Purification by column chromatography (silica gel 8 g, *n*-hexane → *n*-hexane/AcOEt 1:1) afforded cycloadduct **7** (23.9 mg, 54%) as a pale yellow solid. R_f = 0.21 (*n*-hexane/AcOEt 1:1); mp 236.0−240.0 °C (pale yellow needles from *n*-hexane/CH_2Cl_2 2:1); IR (KBr) ν_{max}/cm^{-1} 2949, 2828, 1719, 1472, 1379, 1350, 1306, 1288, 1260, 1238, 1125, 1086, 1043, 932; δ_H (500 MHz; $CDCl_3$) 9.00 (1 H, d, *J* = 2.9 Hz, C*H*O), 6.73 (1 H, s, Ar*H*), 6.67 (1 H, s, Ar*H*), 5.97 (1 H, d, *J* = 1.4 Hz, one of OC*H*₂O), 5.94 (1 H, d, *J* = 1.4 Hz, one of OC*H*₂O), 5.49 (1 H, d, *J* = 5.1 Hz, ArC*H*CHCHO), 4.09−3.95 (4 H, m, OC*H*₂C*H*₂O), 2.94 (1 H, dt, *J* = 5.1, 2.9 Hz, C*H*CHO), 2.52 (1 H, d, *J* = 15.1 Hz, one of ArCC*H*₂), 2.43 (1 H, dd, *J* = 2.5, 15.1 Hz, one of ArCC*H*₂), 2.03 (1 H, dddd, *J* = 3.0, 3.6, 5.8, 13.1 Hz, one of C*H*₂CHCHCHO), 1.95 (1 H, ddd, *J* = 2.9, 5.8, 11.4 Hz, CH_2C*H*CHCHO), 1.87 (1 H, ddt, *J* = 2.5, 13.1, 3.0 Hz, one of C*H*₂CH_2CHCHCHO), 1.81 (1 H, ddt, *J* = 3.0, 11.4, 13.1 Hz, one of C*H*₂CHCHCHO), 1.70 (1 H, dt, *J* = 3.6, 13.1 Hz, one of C*H*₂CH_2CHCHCHO); δ_C (126 MHz; $CDCl_3$) 200.4 (CH), 147.2 (C), 146.7 (C), 140.9 (C), 136.0 (C), 107.7 (C), 102.2 (CH), 101.4 (CH_2), 100.7 (CH), 88.5 (C), 79.4 (CH), 65.2 (CH_2), 64.3 (CH_2), 62.3 (CH), 40.2 (CH), 35.7 (CH_2), 33.6 (CH_2), 28.3 (CH_2); HRMS (ESI) *m*/*z* $[M + Na]^+$ calcd for $C_{18}H_{18}O_6Na$ 353.0996; found 353.0998.

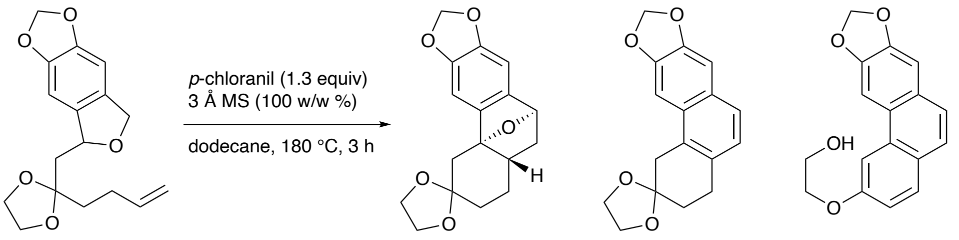

(4a*S,9*R**,10a*S**)-6,7-Methylenedioxy-1,2,3,4,4a,9,10,10a-octahydro-4a,9-epoxyphenanthrene-3-spiro-2'-(1',3'-dioxolane) (8).** The tandem oxidation/intramolecular Diels−Alder reaction was performed according to the typical procedure (16 mL *n*-dodecane, 180 °C, 3 h) employing phthalan **8a** (48.5 mg, 0.159 mmol), *p*-chloranil (50.9 mg, 0.207 mmol) and 3 Å MS (49.0 mg). Purification by column chromatography (silica gel 10 g, *n*-hexane → *n*-hexane/AcOEt 4:1) afforded cycloadduct **8** (24.8 mg, 52%) as a white solid, along with naphthalene **16** (8.6 mg, 19%, white solid), phenanthrene **17** (3.2 mg, 7%, pale orange solid), and recovered phthalan **8a** (9.2 mg, 19%). R_f = 0.38 (*n*-hexane/AcOEt 1:1); mp 151.0−153.0 °C (colorless needles from *n*-hexane/Et_2O 3:1); IR (KBr) ν_{max}/cm^{-1} 2941, 2882, 1470, 1377, 1310, 1265, 1151, 1103, 1042, 1011, 939; δ_H (500 MHz; C_6D_6)

6.46 (1 H, s, Ar*H*), 6.43 (1 H, s, Ar*H*), 5.351 (1 H, d, *J* = 1.2 Hz, one of OC*H*$_2$O), 5.348 (1 H, d, *J* = 1.2 Hz, one of OC*H*$_2$O), 4.92 (1 H, d, *J* = 5.0 Hz, ArC*H*CH$_2$CH), 3.61−3.48 (4 H, m, OC*H*$_2$C*H*$_2$O), 2.56 (1 H, dd, *J* = 2.4, 15.0 Hz, one of ArCC*H*$_2$), 2.42 (1 H, d, *J* = 15.0 Hz, one of ArCC*H*$_2$), 1.93 (1 H, dq, *J* = 3.1, 13.0 Hz, one of CH$_2$C*H*$_2$CHCH$_2$), 1.82 (1 H, ddt, *J* = 2.4, 13.0, 3.1 Hz, one of C*H*$_2$CH$_2$CHCH$_2$), 1.68 (1 H, dq, *J* = 13.0, 3.1 Hz, one of CH$_2$C*H*$_2$CHCH$_2$), 1.61 (1 H, dt, *J* = 3.1, 13.0 Hz, one of C*H*$_2$CH$_2$CHCH$_2$), 1.45 (1 H, m, one of ArCHC*H*$_2$), 1.35−1.29 (2 H, m, one of ArCHC*H*$_2$, CH$_2$CH$_2$C*H*CH$_2$); δ_C (126 MHz; C_6D_6) 146.6 (C), 146.5 (C), 142.2 (C), 141.5 (C), 108.5 (C), 101.0 (2 × CH), 100.9 (CH$_2$), 87.4 (C), 78.6 (CH), 64.7 (CH$_2$), 64.1 (CH$_2$), 38.2 (CH), 37.8 (CH$_2$), 35.8 (CH$_2$), 34.9 (CH$_2$), 29.3 (CH$_2$); HRMS (ESI) *m*/*z* $[M + Na]^+$ calcd for $C_{17}H_{18}O_5Na$ 325.1046; found 325.1061.

Data for 6,7-methylenedioxy-1,2,3,4-tetrahydrophenanthrene-3-spiro-2'-(1',3'-dioxolane) (**16**). R_f = 0.78 (*n*-hexane/AcOEt 1:1); mp 171.0−172.0 °C (colorless needles from *n*-hexane/Et$_2$O 5:1); IR (KBr) ν_{max}/cm^{-1} 2902, 2873, 1473, 1249, 1220, 1120, 1062, 1037, 995, 939, 866; δ_H (500 MHz; CDCl$_3$) 7.46 (1 H, d, *J* = 8.3 Hz, Ar*H*), 7.17 (1 H, s, Ar*H*), 7.10 (1 H, d, *J* = 8.3 Hz, Ar*H*), 7.08 (1 H, s, Ar*H*), 6.03 (2 H, s, OC*H*$_2$O), 4.11−4.05 (4 H, m, OC*H*$_2$C*H*$_2$O), 3.20 (2 H, s, ArC*H*$_2$CO$_2$), 3.09 (2 H, t, *J* = 6.7 Hz, ArC*H*$_2$CH$_2$), 2.02 (2 H, t, *J* = 6.7 Hz, ArCH$_2$C*H*$_2$); δ_C (126 MHz; CDCl$_3$) 148.0 (C), 146.7 (C), 131.5 (C), 129.4 (C), 129.0 (C), 128.5 (C), 125.9 (CH), 125.7 (CH), 108.8 (C), 104.7 (CH), 101.1 (CH$_2$), 99.7 (CH), 64.8 (2 × CH$_2$), 36.9 (CH$_2$), 31.7 (CH$_2$), 28.8 (CH$_2$); HRMS (ESI) *m*/*z* $[M + Na]^+$ calcd for $C_{17}H_{16}O_4Na$ 307.0946; found 307.0938.

Data for 6-(2-hydroxyethyl)oxy-2,3-methylenedioxyphenanthrene (**17**). R_f = 0.31 (*n*-hexane/AcOEt 1:1); IR (KBr) ν_{max}/cm^{-1} 3304, 2922, 2852, 1614, 1504, 1469, 1259, 1037, 939; δ_H (500 MHz; CDCl$_3$) 7.92 (1 H, s, Ar*H*), 7.83 (1 H, d, *J* = 2.4 Hz, Ar*H*), 7.78 (1 H, d, *J* = 8.7 Hz, Ar*H*), 7.58 (1 H, d, *J* = 8.8 Hz, Ar*H*), 7.50 (1 H, d, *J* = 8.8 Hz, Ar*H*), 7.21 (1 H, s, Ar*H*), 7.20 (1 H, dd, *J* = 2.4, 8.7 Hz, Ar*H*), 6.10 (2 H, s, OC*H*$_2$O), 4.31 (2 H, t, *J* = 4.8 Hz, OCH$_2$C*H*$_2$O), 4.07 (2 H, t, *J* = 4.8 Hz, OC*H*$_2$CH$_2$O); δ_C (126 MHz; CDCl$_3$) 157.4 (C), 148.0 (C), 147.8 (C), 131.4 (C), 130.3 (C), 129.0 (C), 126.6 (C), 125.9 (C), 125.1 (CH), 124.5 (CH), 116.5 (CH), 105.9 (CH), 104.7 (CH), 101.5 (CH$_2$), 100.9 (CH), 69.6 (CH$_2$), 61.8 (CH$_2$); HRMS (ESI) *m*/*z* $[M + Na]^+$ calcd for $C_{17}H_{14}O_4Na$ 305.0790;

found 305.0782.

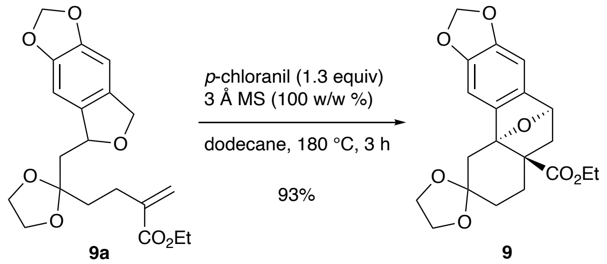

9a **9**

Ethyl (4a*R,9*R**,10a*R**)-6,7-Methylenedioxy-[1,2,3,4,4a,9,10,10a-octahydro-4a,9-epoxyphenanthrene-3-spiro-2'-(1',3'-dioxolane)]-10a-carboxylate (9).** The tandem oxidation/intramolecular Diels−Alder reaction was performed according to the typical procedure (13 mL *n*-dodecane, 180 °C, 3 h) employing phthalan **9a** (49.9 mg, 0.133 mmol), *p*-chloranil (42.3 mg, 0.172 mmol) and 3 Å MS (50.6 mg). Purification by column chromatography (silica gel 15 g, *n*-hexane → *n*-hexane/AcOEt 3:2) afforded cycloadduct **9** (46.3 mg, 93%) as a colorless amorphous solid. R_f = 0.29 (*n*-hexane/AcOEt 1:1); IR (neat) ν_{max}/cm^{-1} 3011, 2980, 2886, 1722, 1470, 1377, 1312, 1279, 1244, 1180, 1099, 1040, 937; δ_H (500 MHz; C_6D_6) 6.75 (1 H, s, Ar*H*), 6.45 (1 H, s, Ar*H*), 5.33 (1 H, br s, one of OCH_2O), 5.27 (1 H, br s, one of OCH_2O), 4.87 (1 H, d, *J* = 5.0 Hz, $ArCHCH_2$), 3.69−3.50 (6 H, m, OCH_2CH_3, OCH_2CH_2O), 3.11 (1 H, d, *J* = 14.7 Hz, one of $ArCCH_2$), 2.52 (1 H, br d, *J* = 14.7 Hz, one of $ArCCH_2$), 2.44 (1 H, br t, *J* = 14.0 Hz, one of $CH_2CH_2CCO_2Et$), 2.28 (1 H, br t, *J* = 14.0 Hz, one of $CH_2CH_2CCO_2Et$), 2.12 (1 H, br d, *J* = 11.9 Hz, one of $ArCHCH_2$), 2.00 (1 H, br d, *J* = 14.0 Hz, one of $CH_2CH_2CCO_2Et$), 1.95 (1 H, dd, *J* = 5.0, 11.9 Hz, one of $ArCHCH_2$), 1.75 (1 H, br d, *J* = 14.0 Hz, one of $CH_2CH_2CCO_2Et$), 0.74 (3 H, t, *J* = 7.1 Hz, OCH_2CH_3); δ_C (126 MHz; C_6D_6) 173.6 (C), 147.0 (C), 146.3 (C), 141.6 (C), 139.9 (C), 108.4 (C), 103.6 (CH), 101.1 (CH_2), 100.8 (CH), 88.4 (C), 78.4 (CH), 64.7 (CH_2), 64.1 (CH_2), 60.3 (CH_2), 51.7 (C), 43.3 (CH_2), 35.1 (CH_2), 34.0 (CH_2), 32.3 (CH_2), 14.0 (CH_3); HRMS (ESI) *m*/*z* $[M + Na]^+$ calcd for $C_{20}H_{22}O_7Na$ 397.1258; found 397.1252.

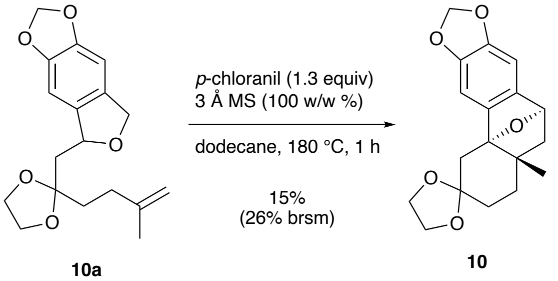

10a **10**

(4a*S,9*R**,10a*S**)-10a-Methyl-6,7-methylenedioxy-1,2,3,4,4a,9,10,10a-octahydro-4a,9-epoxy-**

phenanthrene-3-spiro-2'-(1',3'-dioxolane) (10). The tandem oxidation/intramolecular Diels−Alder reaction was performed according to the typical procedure (13 mL *n*-dodecane, 180 °C, 1 h) employing phthalan **10a** (41.4 mg, 0.130 mmol), *p*-chloranil (41.6 mg, 0.169 mmol) and 3 Å MS (41.4 mg). *p*-Chloranil was completely consumed within 1 h, and the mixture was chromatographed (silica gel 10 g, *n*-hexane → *n*-hexane/AcOEt 9:1 → 4:1 → 2:1) to give a mixture containing cycloadduct **10** (17.2 mg), along with recovered phthalan **10a** (17.4 mg, 42%). Purification of the mixture by column chromatography (silica gel 5 g, toluene → toluene/AcOEt 19:1 → 9:1 → 4:1) and preparative thin layer chromatography (toluene/AcOEt 9:1) afforded cycloadduct **10** (6.2 mg, 15%) as an orange oil. R_f = 0.21 (*n*-hexane/AcOEt 2:1); IR (neat) ν_{max}/cm^{-1} 2953, 2928, 2882, 1468, 1373, 1348, 1310, 1273, 1242, 1111, 1045, 937; δ_H (500 MHz; C_6D_6) 6.46 (1 H, s, Ar*H*), 6.42 (1 H, s, Ar*H*), 5.36 (1 H, d, *J* = 1.4 Hz, one of OC*H*$_2$O), 5.34 (1 H, d, *J* = 1.4 Hz, one of OC*H*$_2$O), 4.88 (1 H, d, *J* = 5.2 Hz, ArC*H*CH$_2$), 3.64−3.51 (4 H, m, OC*H*$_2$C*H*$_2$O), 2.44 (1 H, dd, *J* = 2.5, 14.9 Hz, one of ArCC*H*$_2$), 2.38 (1 H, dt, *J* = 3.4, 13.8 Hz, one of C*H*$_2$CH$_2$CMe), 2.35 (1 H, d, *J* = 14.9 Hz, one of ArCC*H*$_2$), 1.84 (1 H, dt, *J* = 3.4, 13.8 Hz, one of CH$_2$C*H*$_2$CMe), 1.77 (1 H, dd, *J* = 5.2, 11.3 Hz, one of ArCHC*H*$_2$), 1.75 (1 H, ddt, *J* = 2.5, 13.8, 3.4 Hz, one of C*H*$_2$CH$_2$CMe), 1.53 (1 H, dt, *J* = 13.8, 3.4 Hz, one of CH$_2$C*H*$_2$CMe), 0.92 (1 H, d, *J* = 11.3 Hz, one of ArCHC*H*$_2$), 0.48 (3 H, s, ArCHCH$_2$CC*H*$_3$); δ_C (126 MHz; C_6D_6) 146.8 (C), 146.2 (C), 141.3 (C), 140.8 (C), 108.7 (C), 102.5 (CH), 101.1 (CH$_2$), 101.0 (CH), 90.2 (C), 78.4 (CH), 64.7 (CH$_2$), 64.1 (CH$_2$), 46.6 (C), 38.8 (CH$_2$), 36.3 (CH$_2$), 34.0 (CH$_2$), 32.3 (CH$_2$), 22.1 (CH$_3$); HRMS (ESI) *m*/*z* [M + Na]$^+$ calcd for $C_{18}H_{20}O_5Na$ 339.1208; found 339.1221.

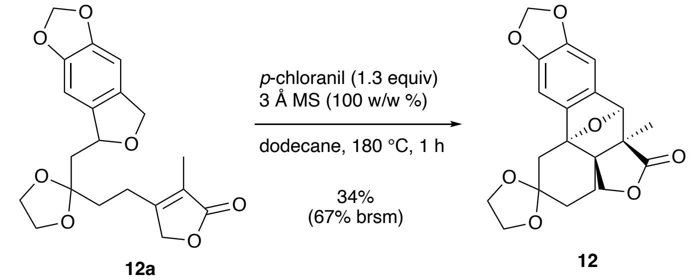

(4a*S,7a*S**,8*S**,12b*R**)-7a-Methyl-10,11-methylenedioxy-[1,2,3,4,4a,5,7,7a,8,12b-decahydro-8,12b-epoxyphenanthro[8a,9-*c*]furan-2-spiro-2'-(1',3'-dioxolane)]-7-one (12).** The tandem oxidation/intramolecular Diels−Alder reaction was performed according to the typical procedure (130 mL *n*-dodecane, 180 °C, 1 h) employing phthalan **12a** (48.5 mg, 0.130 mmol), *p*-chloranil (41.4 mg,

0.168 mmol) and 3 Å MS (48.4 mg). Purification by column chromatography (silica gel 10 g, *n*-hexane → *n*-hexane/AcOEt 9:1 → 4:1 → 3:1 →2:1 → 1:1) afforded cycloadduct **12** (16.5 mg, 34%) as a yellow oil, along with recovered phthalan **12a** (24.0 mg, 49%). R_f = 0.39 (*n*-hexane/AcOEt 1:2); IR (neat) ν_{max}/cm^{-1} 2960, 2899, 2855, 1761, 1471, 1375, 1310, 1258, 1099, 1038, 941; δ_H (500 MHz; $CDCl_3$) 6.80 (1 H, s, Ar*H*), 6.70 (1 H, s, Ar*H*), 6.00 (1 H, d, *J* = 1.5 Hz, one of OC*H*$_2$O), 5.95 (1 H, d, *J* = 1.5 Hz, one of OC*H*$_2$O), 4.92 (1 H, s, ArC*H*), 4.09–3.96 (4 H, m, OC*H*$_2$C*H*$_2$O), 3.81 (1 H, d, *J* = 9.9 Hz, one of COOC*H*$_2$), 3.80 (1 H, d, *J* = 9.9 Hz, one of COOC*H*$_2$), 2.48 (1 H, d, *J* = 15.4 Hz, one of ArCC*H*$_2$), 2.39 (1 H, dd, *J* = 2.4, 15.4 Hz, one of ArCC*H*$_2$), 2.24 (1 H, dt, *J* = 3.4, 14.1 Hz, one of C*H*$_2$CH$_2$CCH$_2$O), 1.92 (1 H, dddd, *J* = 2.4, 3.1, 3.4, 14.1 Hz, one of C*H*$_2$CH$_2$CCH$_2$O), 1.78 (1 H, dt, *J* = 13.6, 3.4 Hz, one of CH$_2$C*H*$_2$CCH$_2$O), 1.58 (1 H, ddd, *J* = 3.1, 13.6, 14.1 Hz, one of CH$_2$C*H*$_2$CCH$_2$O), 1.53 (3 H, s, ArCHCC*H*$_3$); δ_C (126 MHz; $CDCl_3$) 177.9 (C), 147.5 (C), 147.3 (C), 136.9 (C), 136.7 (C), 107.0 (C), 102.9 (CH), 101.8 (CH), 101.6 (CH_2), 91.5 (C), 86.9 (CH), 73.5 (CH_2), 65.2 (CH_2), 64.4 (CH_2), 58.7 (C), 49.9 (C), 35.2 (CH_2), 31.2 (CH_2), 28.8 (CH_2), 17.3 (CH_3); HRMS (ESI) *m*/*z* [M + Na]$^+$ calcd for $C_{20}H_{20}O_7Na$ 395.1107; found 395.1104.

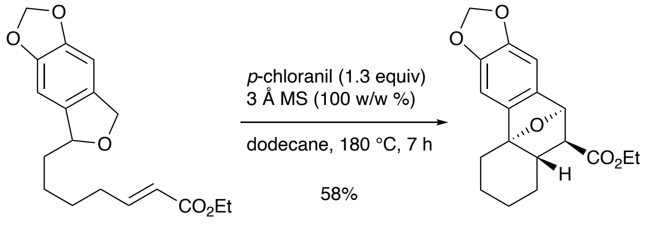

Ethyl (4b*S,8a*S**,9*S**,10*R**)-2,3-Methylenedioxy-4b,5,6,7,8,8a,9,10-octahydro-4b,10-epoxyphenanthrene-9-carboxylate (13).** The tandem oxidation/intramolecular Diels−Alder reaction was performed according to the typical procedure (13 mL *n*-dodecane, 180 °C, 7 h) employing phthalan **13a** (42.3 mg, 0.133 mmol), *p*-chloranil (44.5 mg, 0.180 mmol) and 3 Å MS (44.3 mg). Purification by column chromatography (silica gel 10 g, *n*-hexane → *n*-hexane/AcOEt 8:1) afforded cycloadduct **13** (24.5 mg, 58%) as a colorless oil. R_f = 0.61 (*n*-hexane/AcOEt 2:1); IR (neat) ν_{max}/cm^{-1} 2934, 1734, 1470, 1379, 1312, 1269, 1240, 1198, 1040, 970; δ_H (500 MHz; $CDCl_3$) 6.66 (1 H, s, Ar*H*), 6.65 (1 H, s, Ar*H*), 5.94 (1 H, d, *J* = 1.3 Hz, one of OC*H*$_2$O), 5.91 (1 H, d, *J* = 1.3 Hz, one of OC*H*$_2$O), 5.38 (1 H, d, *J* = 5.3 Hz, ArC*H*CHCO$_2$Et), 3.98 (1 H, dq, *J* = 10.9, 7.3 Hz, one of OC*H*$_2$CH$_3$), 3.94 (1 H, dq, *J* = 10.9, 7.3 Hz, one of OC*H*$_2$CH$_3$), 2.93 (1 H, dd, *J* = 3.0, 5.3 Hz, C*H*CO$_2$Et), 2.25 (1 H, br d, *J* = 14.7 Hz, one of ArCC*H*$_2$), 2.19 (1 H, ddd, *J* = 4.8, 13.1, 14.7 Hz, one

of ArCC*H*2), 2.06 (1 H, m, one of C*H*2CHCHCO2Et), 1.98 (1 H, ddd, *J* = 3.0, 6.0, 11.3 Hz, CH2C*H*CHCO2Et), 1.83−1.75 (2 H, m, one of ArCCH2C*H*2, one of C*H*2CH2CHCHCO2Et), 1.58 (1 H, m, one of ArCCH2C*H*2), 1.37−1.30 (2 H, m, one of C*H*2CH2CHCHCO2Et, one of C*H*2CHCHCO2Et), 1.19 (3 H, t, *J* = 7.3 Hz, OCH2C*H*3); δ_C (126 MHz; CDCl3) 171.0 (C), 146.7 (C), 146.1 (C), 141.8 (C), 137.1 (C), 102.3 (CH), 101.1 (CH2), 100.6 (CH), 87.1 (C), 79.5 (CH), 60.5 (CH2), 56.0 (CH), 41.2 (CH), 32.1 (CH2), 27.2 (CH2), 25.0 (CH2), 21.9 (CH2), 14.2 (CH3); HRMS (ESI) *m*/*z* [M + Na]$^+$ calcd for $C_{18}H_{20}O_5Na$ 339.1203; found 339.1209.

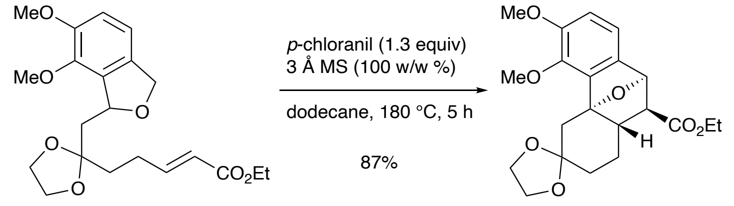

14a 14

Ethyl (4a*S,9*R**,10*S**,10a*S**)-5,6-Dimethoxy-[1,2,3,4,4a,9,10,10a-octahydro-4a,9-epoxyphenanthrene-3-spiro-2'-(1',3'-dioxolane)]-10-carboxylate (14).** The tandem oxidation/intramolecular Diels−Alder reaction was performed according to the typical procedure (13 mL *n*-dodecane, 180 °C, 5 h) employing phthalan **14a** (52.3 mg, 0.133 mmol), *p*-chloranil (42.7 mg, 0.173 mmol) and 3 Å MS (52.2 mg). Purification by column chromatography (silica gel 8 g, *n*-hexane → *n*-hexane/AcOEt 2:1) afforded cycloadduct **14** (45.3 mg, 87%) as a white solid. R_f = 0.37 (*n*-hexane/AcOEt 2:1); mp 136.0−137.0 °C (colorless needles from *n*-hexane/Et2O 10:1); IR (KBr) ν_{max}/cm^{-1} 2940, 1734, 1485, 1375, 1246, 1180, 1152, 1098, 999; δ_H (500 MHz; CDCl3) 6.81 (1 H, d, *J* = 7.7 Hz, Ar*H*), 6.64 (1 H, d, *J* = 7.7 Hz, Ar*H*), 5.43 (1 H, d, *J* = 5.3 Hz, ArC*H*CHCO2Et), 4.09−3.97 (4 H, m, OC*H*2C*H*2O), 3.93 (1 H, dq, *J* = 10.7, 7.2 Hz, one of OC*H*2CH3), 3.89 (1 H, dq, *J* = 10.7, 7.2 Hz, one of OC*H*2CH3), 3.824 (3 H, s, OC*H*3), 3.816 (3 H, s, OC*H*3), 3.28 (1 H, d, *J* = 15.5 Hz, one of ArCC*H*2), 3.00 (1 H, dd, *J* = 3.1, 5.3 Hz, C*H*CO2Et), 2.48 (1 H, dd, *J* = 2.6, 15.5 Hz, one of ArCC*H*2), 2.18 (1 H, m, CH2C*H*CHCO2Et), 2.07 (1 H, m, one of C*H*2CHCHCO2Et), 1.85 (1 H, m, one of C*H*2CH2CHCHCO2Et), 1.81−1.70 (2 H, m, one of C*H*2CH2CHCHCO2Et, one of C*H*2CHCHCO2Et), 1.15 (3 H, t, *J* = 7.2 Hz, OCH2C*H*3); δ_C (126 MHz; CDCl3) 170.8 (C), 152.6 (C), 143.0 (C), 138.3 (C), 136.5 (C), 115.7 (CH), 110.5 (CH), 107.8 (C), 89.3 (C), 79.6 (CH), 65.0 (CH2), 64.0 (CH2), 61.0 (CH3), 60.5 (CH2), 56.0 (CH3), 55.5 (CH), 40.8 (CH), 36.1 (CH2), 33.4 (CH2), 28.6 (CH2), 14.2 (CH3); HRMS (ESI) *m*/*z* [M + Na]$^+$ calcd for $C_{21}H_{26}O_7Na$ 413.1571; found 413.1571.

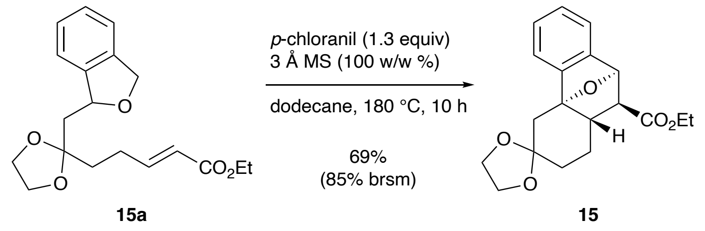

Ethyl (4a*S,9*R**,10*S**,10a*S**)-[1,2,3,4,4a,9,10,10a-Octahydro-4a,9-epoxyphenanthrene-3-spiro-2'-(1',3'-dioxolane)]-10-carboxylate (15).** The tandem oxidation/intramolecular Diels–Alder reaction was performed according to the typical procedure (15 mL *n*-dodecane, 180 °C, 10 h) employing phthalan **15a** (50.2 mg, 0.151 mmol), *p*-chloranil (48.3 mg, 0.196 mmol) and 3 Å MS (51.2 mg). Purification by column chromatography (silica gel 10 g, *n*-hexane → *n*-hexane/AcOEt 3:1) afforded cycloadduct **15** (34.5 mg, 69%) as a pale orange solid, along with recovered phthalan **15a** (9.4 mg, 19%). R_f = 0.52 (*n*-hexane/AcOEt 1:1); mp 136.0–138.0 °C (colorless needles from *n*-hexane/Et_2O 10:1); IR (KBr) ν_{max}/cm^{-1} 2953, 2884, 1732, 1462, 1377, 1244, 1209, 1182, 1167, 1140, 1084, 1057, 1011, 980; δ_H (500 MHz; $CDCl_3$) 7.21–7.11 (4 H, m, Ar*H*), 5.53 (1 H, d, *J* = 5.4 Hz, ArC*H*CHCO$_2$Et), 4.08–3.96 (4 H, m, OC*H*$_2$C*H*$_2$O), 3.94 (1 H, dq, *J* = 10.8, 7.1 Hz, one of OC*H*$_2$CH$_3$), 3.91 (1 H, dq, *J* = 10.8, 7.1 Hz, one of OC*H*$_2$CH$_3$), 3.05 (1 H, dd, *J* = 2.5, 5.4 Hz, C*H*CO$_2$Et), 2.62 (1 H, d, *J* = 15.2 Hz, one of ArCC*H*$_2$), 2.42 (1 H, dd, *J* = 2.4, 15.2 Hz, one of ArCC*H*$_2$), 2.10–2.03 (2 H, m, one of C*H*$_2$CHCHCO$_2$Et, CH$_2$C*H*CHCO$_2$Et), 1.87 (1 H, dq, *J* = 12.7, 2.4 Hz, one of C*H*$_2$CH$_2$CHCHCO$_2$Et), 1.81 (1 H, m, one of C*H*$_2$CHCHCO$_2$Et), 1.73 (1 H, br t, *J* = 12.7 Hz, one of C*H*$_2$CH$_2$CHCHCO$_2$Et), 1.15 (3 H, t, *J* = 7.1 Hz, OCH$_2$C*H*$_3$); δ_C (126 MHz; $CDCl_3$) 170.7 (C), 147.1 (C), 142.7 (C), 127.2 (CH), 126.6 (CH), 120.4 (CH), 118.0 (CH), 107.8 (C), 88.2 (C), 80.0 (CH), 65.1 (CH_2), 64.2 (CH_2), 60.5 (CH_2), 54.8 (CH), 40.7 (CH), 35.5 (CH_2), 33.6 (CH_2), 28.5 (CH_2), 14.1 (CH_3); HRMS (ESI) *m/z* $[M + Na]^+$ calcd for $C_{19}H_{22}O_5Na$ 353.1359; found 353.1353.

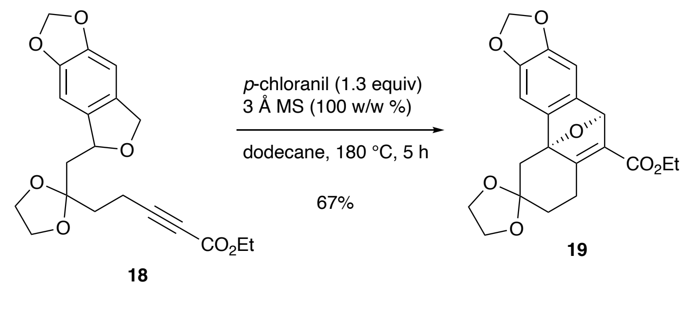

Ethyl (4a*S,9*S**)-6,7-Methylenedioxy-[1,2,3,4,4a,9-hexahydro-4a,9-epoxyphenanthrene-3-spi-**

ro-2'-(1',3'-dioxolane)]-10-carboxylate (19). The tandem oxidation/intramolecular Diels−Alder reaction was performed according to the typical procedure (13 mL *n*-dodecane, 180 °C, 5 h) employing phthalan **18** (49.1 mg, 0.133 mmol), *p*-chloranil (41.6 mg, 0.173 mmol) and 3 Å MS (48.7 mg). Purification by column chromatography (silica gel 10 g, *n*-hexane → *n*-hexane/AcOEt 4:1) afforded cycloadduct **19** (32.4 mg, 67%) as a gray amorphous solid. R_f = 0.50 (*n*-hexane/AcOEt 2:1); IR (neat) ν_{max}/cm^{-1} 3034, 2955, 2895, 1740, 1606, 1508, 1454, 1406, 1287, 1246, 1172, 1070, 1026, 970, 854, 831, 735, 696; δ_H (500 MHz; $CDCl_3$) 7.10 (1 H, s, Ar*H*), 6.85 (1 H, s, Ar*H*), 5.94 (1 H, d, *J* = 1.4 Hz, one of OC*H*$_2$O), 5.90 (1 H, d, *J* = 1.4 Hz, one of OC*H*$_2$O), 5.75 (1 H, s, ArC*H*C(CO_2Et)=C), 4.19 (1 H, m, one of OC*H*$_2$C*H*$_2$O), 4.18 (2 H, q, *J* = 7.2 Hz, OC*H*$_2$$CH_3$), 4.14−4.01 (3 H, m, three of OC*H*$_2$C*H*$_2$O), 3.34 (1 H, ddd, *J* = 2.4, 4.7, 17.4 Hz, one of C*H*$_2$C=CCO_2Et), 2.67 (1 H, dd, *J* = 2.4, 13.9 Hz, one of ArCC*H*$_2$), 2.36 (1 H, d, *J* = 13.9 Hz, one of ArCC*H*$_2$), 2.28 (1 H, ddd, *J* = 5.5, 13.9, 17.4 Hz, one of C*H*$_2$C=CCO_2Et), 1.95 (1 H, ddt, *J* = 5.5, 13.3, 2.4 Hz, one of C*H*$_2$$CH_2$C=CC$O_2$Et), 1.84 (1 H, ddd, *J* = 4.7, 13.3, 13.9 Hz, one of C*H*$_2$$CH_2$C=CC$O_2$Et), 1.28 (3 H, t, *J* = 7.2 Hz, OCH_2C*H*$_3$); δ_C (126 MHz; $CDCl_3$) 166.2 (C), 164.0 (C), 144.8 (C), 144.6 (C), 144.0 (C), 142.9 (C), 138.5 (C), 108.0 (C), 104.8 (CH), 103.0 (CH), 101.2 (CH_2), 92.1 (C), 81.7 (CH), 65.0 (CH_2), 64.6 (CH_2), 60.3 (CH_2), 38.2 (CH_2), 31.8 (CH_2), 22.7 (CH_2), 14.3 (CH_3); HRMS (ESI) *m*/*z* $[M + Na]^+$ calcd for $C_{20}H_{20}O_7Na$ 395.1107; found 395.1094.

2-3. Formal Synthesis of (±)-Morphine

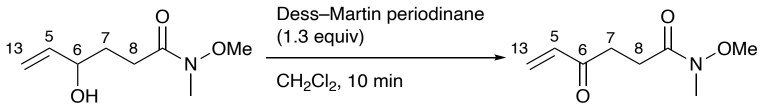

S37 91% **21**

***N*-Methoxy-*N*-methyl-4-oxohex-5-enamide (21).** Dess−Martin periodinane[57] (554 mg, 1.31 mmol) was added to a solution of alcohol **S37**[58] (174 mg, 1.00 mmol) in CH_2Cl_2 (2 mL). After 10 min of stirring, the reaction was quenched by addition of a mixture of 1 M aqueous $Na_2S_2O_3$ (5 mL) and saturated aqueous $NaHCO_3$ (5 mL), and the resulting mixture was extracted with AcOEt (30 mL). The organic extract was successively washed with water (10 mL) and brine (30 mL) and dried over anhydrous Na_2SO_4. Filtration and evaporation in vacuo furnished the crude product (352 mg), which was purified by column chromatography (silica gel 10 g, *n*-hexane/AcOEt 1:1) to give enone **21** (156 mg, 91%) as a yellow oil. R_f = 0.59 (*n*-hexane/AcOEt 1:3); IR (neat) ν_{max}/cm^{-1} 2970, 2940,

1663, 1616, 1404, 1389, 1179, 993, 756; δ_H (500 MHz; $CDCl_3$) 6.39 (1 H, dd, J = 10.3, 17.8 Hz, C(5)H), 6.29 (1 H, d, J = 17.8 Hz, one of C(13)H_2), 5.86 (1 H, d, J = 10.3 Hz, one of C(13)H_2), 3.74 (3 H, s, NOCH_3), 3.19 (3 H, s, NCH_3), 2.95 (2 H, t, J = 6.9 Hz, C(7)H_2), 2.78 (2 H, t, J = 6.9 Hz, C(8)H_2); δ_C (126 MHz; $CDCl_3$) 199.4 (C), 173.1 (C), 136.4 (CH), 128.4 (CH_2), 61.2 (CH_3), 33.7 (CH_2), 32.2 (CH_3), 25.8 (CH_2); HRMS (ESI) m/z $[M + Na]^+$ calcd for $C_8H_{13}NO_3Na$ 194.0793; found 194.0825.

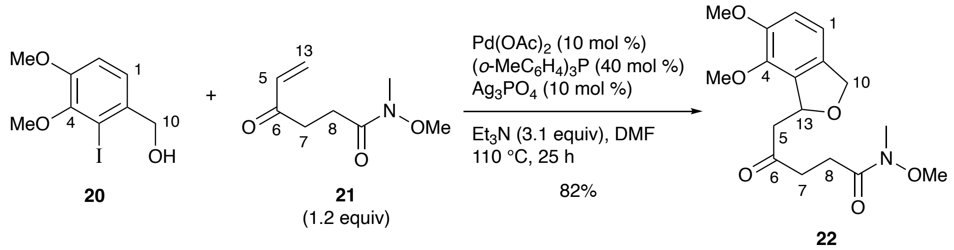

5-(4,5-Dimethoxy-1,3-dihydroisobenzofuran-3-yl)-*N*-methoxy-*N*-methyl-4-oxopentanamide (22). $Pd(OAc)_2$ (63.8 mg, 0.284 mmol) was added to a mixture of enone **21** (580 mg, 3.37 mmol), iodide **20**[55] (826 mg, 2.81 mmol), tri(*o*-tolyl)phosphine (344 mg, 1.13 mmol), Et_3N (1.2 mL, 8.66 mmol) and Ag_3PO_4 (118 mg, 0.282 mmol) in DMF (7 mL). After 25 h of stirring at 110 °C, the reaction was quenched with 1 M aqueous hydrochloric acid (4 mL), and the mixture was extracted with *n*-hexane/AcOEt (1:4, 3 × 50 mL). The combined organic extracts were successively washed with water (60 mL), saturated aqueous $NaHCO_3$ (60 mL) and brine (2 × 60 mL) and dried over anhydrous Na_2SO_4. Filtration and evaporation in vacuo furnished the crude product (860 mg), which was purified by column chromatography (silica gel 30 g, *n*-hexane/AcOEt 1:1) to give phthalan **22** (774 mg, 82%) as a yellow oil. R_f = 0.21 (*n*-hexane/AcOEt 1:1); IR (neat) ν_{max}/cm^{-1} 2940, 2860, 2837, 1715, 1649, 1493, 1271, 1038, 805, 733; δ_H (500 MHz; $CDCl_3$) 6.85 (2 H, s, ArH), 5.79 (1 H, dddd, J = 1.2, 2.4, 2.9, 9.3 Hz, C(13)H), 5.06 (1 H, dd, J = 2.4, 11.7 Hz, one of C(10)H_2), 4.97 (1 H, dd, J = 1.2, 11.7 Hz, one of C(10)H_2), 3.86 (3 H, s, OCH_3), 3.84 (3 H, s, OCH_3), 3.74 (3 H, s, NOCH_3), 3.18 (3 H, s, NCH_3), 3.16 (1 H, dd, J = 2.9, 15.9 Hz, one of C(5)H_2), 2.91 (1 H, dt, J = 18.1, 6.8 Hz, one of C(7)H_2), 2.825 (1 H, dd, J = 9.3, 15.9 Hz, one of C(5)H_2), 2.824 (1 H, dt, J = 18.1, 6.1 Hz, one of C(7)H_2), 2.78 (1 H, m, one of C(8)H_2), 2.71 (1 H, m, one of C(8)H_2); δ_C (126 MHz; $CDCl_3$) 207.4 (C), 173.1 (C), 151.7 (C), 143.4 (C), 134.1 (C), 132.9 (C), 115.9 (CH), 113.1 (CH), 79.0 (CH), 72.4 (CH_2), 61.2 (CH_3), 60.5 (CH_3), 56.3 (CH_3), 48.0 (CH_2), 37.8 (CH_2), 32.2 (CH_3), 26.6 (CH_2); HRMS (ESI) m/z $[M + Na]^+$ calcd for $C_{17}H_{23}NO_6Na$ 360.1418; found 360.1435.

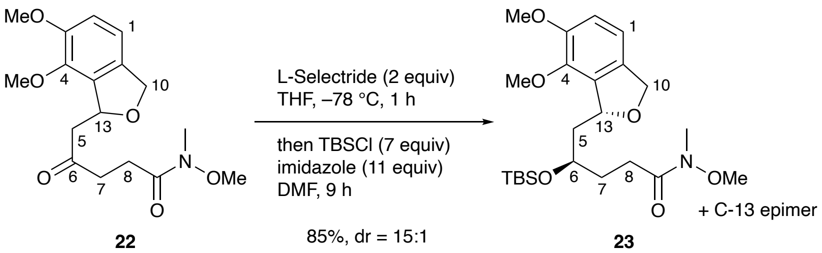

22 23

4-(*tert*-Butyldimethylsilyl)oxy-5-(4,5-dimethoxy-1,3-dihydroisobenzofuran-3-yl)-*N*-methoxy-*N*-methylpentanamide (23). L-Selectride in THF (1.02 M, 17.8 mL, 18.2 mmol) was added to a cooled (−78 °C) solution of ketone **22** (3.07 g, 9.10 mmol) in THF (30 mL). After 1 h of stirring, a mixture of TBSCl (9.60 g, 63.7 mmol) and imidazole (6.83 g, 100 mmol) in DMF (50 mL) was added, and the resulting mixture was stirred at room temperature for 9 h. The reaction was quenched with H_2O (100 mL), and AcOEt (40 mL) was added. After another 1 h of stirring, the mixture was extracted with AcOEt (60 mL), and the organic extract was washed with brine (100 mL) and dried over anhydrous Na_2SO_4. Filtration and evaporation in vacuo furnished the crude product (12.3 g, yellow oil), which was purified by column chromatography (silica gel 120 g, *n*-hexane/AcOEt 5:2) to give TBS ether **23** (3.52 g, 85%, dr = 15:1) as a yellow oil. R_f = 0.76 (*n*-hexane/AcOEt 1:2); IR (neat) ν_{max}/cm^{-1} 2953, 2855, 1665, 1491, 1271, 1090, 837, 775; δ_H (600 MHz; 50 °C; $CDCl_3$; spectrum contains a 15:1 mixture of diastereomers, only the major isomer signals are reported) 6.84 (2 H, s, Ar*H*), 5.40 (1 H, dt, *J* = 10.9, 2.3 Hz, C(13)*H*), 5.04 (1 H, dd, *J* = 2.3, 12.0 Hz, one of C(10)H_2), 4.93 (1 H, d, *J* = 12.0 Hz, one of C(10)H_2), 4.10 (1 H, dddd, *J* = 3.4, 4.0, 8.0, 9.2 Hz, C(6)*H*), 3.86 (3 H, s, OCH_3), 3.85 (3 H, s, OCH_3), 3.69 (3 H, s, NOCH_3), 3.19 (3 H, s, NCH_3), 2.67 (1 H, ddd, *J* = 5.2, 10.9, 15.8 Hz, one of C(8)H_2), 2.50 (1 H, ddd, *J* = 5.2, 10.9, 15.8 Hz, one of C(8)H_2), 2.18 (1 H, ddd, *J* = 2.3, 9.2, 13.7 Hz, one of C(5)H_2), 2.09 (1 H, dddd, *J* = 4.0, 5.2, 10.9, 13.7 Hz, one of C(7)H_2), 1.89 (1 H, dddd, *J* = 5.2, 8.0, 10.9, 13.7 Hz, one of C(7)H_2), 1.82 (1 H, ddd, *J* = 3.4, 10.9, 13.7 Hz, one of C(5)H_2), 0.90 (9 H, s, SiC(CH_3)$_3$), 0.08 (3 H, s, SiCH_3), 0.07 (3 H, s, SiCH_3); δ_C (150 MHz; 50 °C; $CDCl_3$) 175.0 (C), 151.8 (C), 143.8 (C), 135.8 (C), 133.0 (C), 115.8 (CH), 113.2 (CH), 80.2 (CH), 71.9 (CH_2), 69.5 (CH), 61.2 (CH_3), 60.5 (CH_3), 56.5 (CH_3), 42.4 (CH_2), 32.8 (CH_3), 31.0 (CH_2), 28.1 (CH_2), 25.9 (3 × CH_3), 18.1 (C), −4.5 (CH_3), −4.7 (CH_3); HRMS (ESI) *m*/*z* $[M + Na]^+$ calcd for $C_{23}H_{39}NO_6SiNa$ 476.2439; found 476.2462.

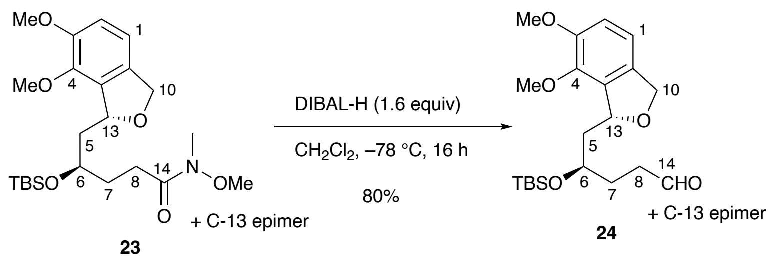

4-(*tert*-Butyldimethylsilyl)oxy-5-(4,5-dimethoxy-1,3-dihydroisobenzofuran-3-yl)pentanal (24). DIBAL-H in *n*-hexane (1.0 M, 3.1 mL, 3.1 mmol) was added to a cooled (−78 °C) solution of amide **23** (883 mg, 1.95 mmol) in CH_2Cl_2 (8 mL). After 14 h of stirring, the reaction mixture was poured into a two-layer mixture of Et_2O (20 mL) and saturated aqueous potassium sodium tartrate (50 mL), and the whole mixture was extracted with AcOEt (2 × 100 mL). The combined organic extracts were washed with brine (100 mL) and dried over anhydrous Na_2SO_4. Filtration and evaporation in vacuo furnished the crude product (720 mg, yellow oil), which was purified by column chromatography (silica gel 20 g, *n*-hexane/AcOEt 6:1) to give aldehyde **24** (615 mg, 80%, dr 15:1) as a colorless oil. R_f = 0.64 (CH_2Cl_2/acetone 30:1); IR (neat) ν_{max}/cm^{-1} 2953, 2854, 1724, 1490, 1269, 1085, 837, 775; δ_H (500 MHz; $CDCl_3$; spectrum contains a 15:1 mixture of diastereomers, only the major isomer signals are reported) 9.82 (1 H, t, *J* = 1.7 Hz, C(14)*H*), 6.86 (1 H, d, *J* = 8.2 Hz, Ar*H*), 6.84 (1 H, d, *J* = 8.2 Hz, Ar*H*), 5.38 (1 H, br d, *J* = 9.2 Hz, C(13)*H*), 5.04 (1 H, dd, *J* = 2.3, 11.5 Hz, one of C(10)H_2), 4.95 (1 H, d, *J* = 11.5 Hz, one of C(10)H_2), 4.05 (1 H, dddd, *J* = 1.7, 4.0, 7.4, 10.9 Hz, C(6)*H*), 3.861 (3 H, s, OCH_3), 3.858 (3 H, s, OCH_3), 2.59 (1 H, dddd, *J* = 1.7, 6.3, 8.6, 17.2 Hz, one of C(8)H_2), 2.53 (1 H, dddd, *J* = 1.7, 6.3, 8.6, 17.2 Hz, one of C(8)H_2), 2.15 (1 H, ddd, *J* = 1.7, 9.2, 14.3 Hz, one of C(5)H_2), 2.08 (1 H, dddd, *J* = 4.0, 6.3, 8.6, 13.7 Hz, one of C(7)H_2), 1.91 (1 H, dddd, *J* = 6.3, 7.4, 8.6, 13.7 Hz, one of C(7)H_2), 1.81 (1 H, ddd, *J* = 2.9, 10.9, 14.3 Hz, one of C(5)H_2), 0.88 (9 H, s, SiC(CH_3)$_3$), 0.06 (3 H, s, SiCH_3), 0.05 (3 H, s, SiCH_3); δ_C (126 MHz; $CDCl_3$) 202.7 (CH), 151.7 (C), 143.5 (C), 135.4 (C), 132.8 (C), 115.8 (CH), 112.7 (CH), 79.8 (CH), 71.7 (CH_2), 68.5 (CH), 60.4 (CH_3), 56.3 (CH_3), 42.0 (CH_2), 39.2 (CH_2), 30.1 (CH_2), 25.9 (3 × CH_3), 18.1 (C), −4.3 (CH_3), −4.7 (CH_3); HRMS (ESI) *m*/*z* $[M + Na]^+$ calcd for $C_{21}H_{34}O_5SiNa$ 417.2068; found 417.2069.

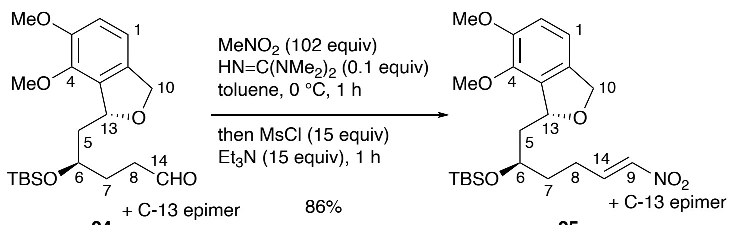

3-[(5*E*)-2-(*tert*-Butyldimethylsilyl)oxy-6-nitrohex-5-enyl]-4,5-dimethoxy-1,3-dihydroisobenzofuran (25). 1,1,3,3-Tetramethylguanidine (10 μL, 7.99 μmol) was added to an ice-cooled (0 °C) solution of aldehyde **24** (31.1 mg, 78.8 μmol) and $MeNO_2$ (0.43 mL, 8.03 mmol) in toluene (1.7 mL). After 1 h of stirring, Et_3N (0.17 mL, 1.22 mmol) and MsCl (90 μL, 1.16 mmol) were added, and the resulting mixture was stirred at room temperature for 15 min. The reaction was quenched with saturated aqueous NH_4Cl (4 mL), and the mixture was extracted with AcOEt (20 mL). The organic extract was washed brine (10 mL) and dried over anhydrous Na_2SO_4. Filtration and evaporation in vacuo furnished the crude product (56.2 mg, yellow oil), which was purified by column chromatography (silica gel 3 g, *n*-hexane/AcOEt 5:1) to give nitroalkene **25** (29.5 mg, 86%, dr 15:1) as a colorless oil. R_f = 0.75 (*n*-hexane/AcOEt 2:1); IR (neat) ν_{max}/cm^{-1} 2953, 2854, 1647, 1525, 1490, 1352, 1269, 1082, 1047, 835; δ_H (500 MHz; $CDCl_3$; spectrum contains a 15:1 mixture of diastereomers, only the major isomer signals are reported) 7.35 (1 H, dt, *J* = 13.5, 7.3 Hz, C(14)*H*), 7.01 (1 H, dt, *J* = 13.5, 1.5 Hz, C(9)*H*), 6.87 (1 H, d, *J* = 8.2 Hz, Ar*H*), 6.85 (1 H, d, *J* = 8.2 Hz, Ar*H*), 5.37 (1 H, br d, *J* = 10.5 Hz, C(13)*H*), 5.04 (1 H, dd, *J* = 2.3, 11.7 Hz, one of C(10)H_2), 4.95 (1 H, d, *J* = 11.7 Hz, one of C(10)H_2), 4.04 (1 H, dddd, *J* = 3.5, 3.8, 7.6, 9.0 Hz, C(6)*H*), 3.87 (3 H, s, OCH_3), 3.86 (3 H, s, OCH_3), 2.47 (1 H, ddddd, *J* = 1.5, 5.2, 7.3, 10.1, 15.5 Hz, one of C(8)H_2), 2.34 (1 H, ddddd, *J* = 1.5, 5.6, 7.3, 10.1, 15.5 Hz, one of C(8)H_2), 2.17 (1 H, ddd, *J* = 2.1, 9.0, 14.1 Hz, one of C(5)H_2), 1.89 (1 H, dddd, *J* = 3.8, 5.6, 10.1, 13.4 Hz, one of C(7)H_2), 1.83 (1 H, ddd, *J* = 3.5, 10.5, 14.1 Hz, one of C(5)H_2), 1.78 (1 H, dddd, *J* = 5.2, 7.6, 10.1, 13.4 Hz, one of C(7)H_2), 0.89 (9 H, s, SiC(CH_3)$_3$), 0.07 (3 H, s, SiCH_3), 0.06 (3 H, s, SiCH_3); δ_C (126 MHz; $CDCl_3$; spectrum contains a 15:1 mixture of diastereomers, only the major isomer signals are reported) 151.7 (C), 143.34 (C), 143.25 (CH), 139.4 (CH), 135.2 (C), 132.5 (C), 115.9 (CH), 112.9 (CH), 80.0 (CH), 72.0 (CH_2), 69.1 (CH), 60.5 (CH_3), 56.3 (CH_3), 42.1 (CH_2), 34.1 (CH_2), 25.8 (3 × CH_3), 24.7 (CH_2), 18.2 (C), −4.4 (CH_3), −4.7 (CH_3); HRMS (ESI) *m*/*z* $[M + Na]^+$ calcd for $C_{22}H_{35}NO_6SiNa$ 460.2126; found 460.2115.

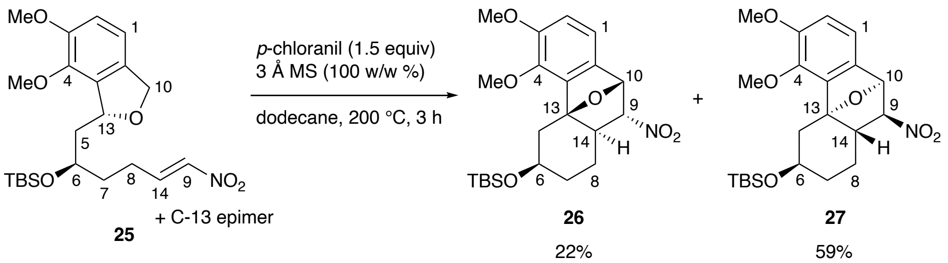

25 26 22% 27 59%

(4b*S,6*R**,8a*S**,9*S**,10*S**)-6-(*tert*-Butyldimethylsilyl)oxy-3,4-dimethoxy-9-nitro-4b,5,6,7,8,8a,9,10-octahydro-4b,10-epoxyphenanthrene (27).** A mixture of phthalan **25** (392 mg, 0.897 mmol), *p*-chloranil (332 mg, 1.35 mmol), and 3 Å MS (392.5 mg) in *n*-dodecane (45 mL) was heated at 200 °C for 3 h. After cooling, the reaction was quenched with saturated aqueous $NaHCO_3$ (20 mL) and 1.0 M aqueous $Na_2S_2O_3$ (10 mL), and the resulting mixture was partitioned between AcOEt (100 mL) and H_2O (10 mL). The organic extract was successively washed with saturated aqueous $NaHCO_3$ (2 × 100 mL) and brine (100 mL) and dried over anhydrous Na_2SO_4. Filtration and evaporation in vacuo followed by column chromatography (silica gel 45 g, *n*-hexane → *n*-hexane/CH_2Cl_2 1:1) afforded cycloadduct **27** (231 mg, 59%) as a pale orange solid and cycloadduct **26** (84.2 mg, 22%) as a green solid. R_f = 0.46 (CH_2Cl_2); mp 77.0−78.0 °C (colorless needles from *n*-hexane); IR (KBr) ν_{max}/cm^{-1} 2930, 2856, 1546, 1487, 1384, 1259, 1097, 1004, 991, 835; δ_H (500 MHz; $CDCl_3$) 6.89 (1 H, d, *J* = 7.9 Hz, Ar*H*), 6.70 (1 H, d, *J* = 7.9 Hz, Ar*H*), 5.57 (1 H, d, *J* = 5.3 Hz, C(10)*H*), 4.76 (1 H, dd, *J* = 2.2, 5.3 Hz, C(9)*H*), 3.89 (1 H, tt, *J* = 4.3, 10.6 Hz, C(6)*H*), 3.85 (3 H, s, OCH_3), 3.83 (3 H, s, OCH_3), 2.88 (1 H, dd, *J* = 10.6, 14.8 Hz, one of C(5)H_2), 2.53 (1 H, ddd, *J* = 2.3, 4.3, 14.8 Hz, one of C(5)H_2), 2.45 (1 H, ddd, *J* = 2.2, 6.8, 11.8 Hz, C(14)*H*), 2.21 (1 H, ddt, *J* = 6.8, 13.6, 2.8 Hz, one of C(8)H_2), 1.95 (1 H, dddt, *J* = 2.3, 4.3, 12.5, 2.8 Hz, one of C(7)H_2), 1.54 (1 H, dddd, *J* = 2.8, 10.6, 10.9, 12.5 Hz, one of C(7)H_2), 1.45 (1 H, dddd, *J* = 2.8, 10.9, 11.8, 13.6 Hz, one of C(8)H_2), 0.91 (9 H, s, SiC(CH_3)$_3$), 0.095 (3 H, s, SiCH_3), 0.093 (3 H, s, SiCH_3); δ_C (126 MHz; $CDCl_3$) 153.6 (C), 143.2 (C), 138.0 (C), 133.0 (C), 117.5 (CH), 111.5 (CH), 92.1 (CH), 91.2 (C), 79.6 (CH), 67.8 (CH), 61.3 (CH_3), 56.2 (CH_3), 42.1 (CH), 37.6 (CH_2), 34.5 (CH_2), 28.9 (CH_2), 26.0 (3 × CH_3), 18.4 (C), −4.46 (CH_3), −4.50 (CH_3); HRMS (ESI) *m*/*z* [M + Na]$^+$ calcd for $C_{22}H_{33}NO_6SiNa$ 458.1969; found 458.1957.

Data for ethyl (4b*R**,6*R**,8a*R**,9*R**,10*R**)-6-(*tert*-butyldimethylsilyl)oxy-3,4-dimethoxy-9-nitro-4b,5,6,7,8,8a,9,10-octahydro-4b,10-epoxyphenanthrene (**26**). R_f = 0.70 (CH_2Cl_2); mp 119.1−120.1 °C

(colorless needles from *n*-hexane); IR (KBr) ν_{max}/cm^{-1} 2924, 2850, 1550, 1485, 1375, 1255, 1246, 1111, 1082, 1033, 1008, 875; δ_H (500 MHz; $CDCl_3$) 6.88 (1 H, d, *J* = 8.0 Hz, Ar*H*), 6.67 (1 H, d, *J* = 8.0 Hz, Ar*H*), 5.54 (1 H, d, *J* = 5.2 Hz, C(10)*H*), 4.82 (1 H, dd, *J* = 2.4, 5.2 Hz, C(9)*H*), 4.25 (1 H, tt, *J* = 2.8, 3.7 Hz, C(6)*H*), 3.824 (3 H, s, OCH_3), 3.817 (3 H, s, OCH_3), 3.10 (1 H, dd, *J* = 3.7, 15.6 Hz, one of C(5)H_2), 2.45 (1 H, ddd, *J* = 2.4, 6.3, 13.0 Hz, C(14)*H*), 2.33 (1 H, ddd, *J* = 2.3, 2.8, 15.6 Hz, one of C(5)H_2), 1.99 (1 H, dq, *J* = 3.4, 13.0 Hz, one of C(8)H_2), 1.89 (1 H, ddt, *J* = 6.3, 13.0, 3.4 Hz, one of C(8)H_2), 1.80 (1 H, dddt, *J* = 2.3, 2.8, 13.0, 3.4 Hz, one of C(7)H_2), 1.65 (1 H, ddt, *J* = 3.4, 3.7, 13.0 Hz, one of C(7)H_2), 0.90 (9 H, s, SiC(CH_3)$_3$), 0.062 (3 H, s, SiCH_3), 0.056 (3 H, s, SiCH_3); δ_C (126 MHz; $CDCl_3$) 153.4 (C), 142.9 (C), 139.3 (C), 133.6 (C), 117.6 (CH), 111.3 (CH), 92.1 (CH), 88.3 (C), 79.1 (CH), 64.6 (CH), 61.2 (CH_3), 56.2 (CH_3), 43.2 (CH), 34.5 (CH_2), 31.7 (CH_2), 25.9 (3 × CH_3), 23.1 (CH_2), 18.2 (C), −4.7 (CH_3), −4.8 (CH_3); HRMS (ESI) *m*/*z* [M + Na]$^+$ calcd for $C_{22}H_{33}NO_6SiNa$ 458.1969; found 458.1981.

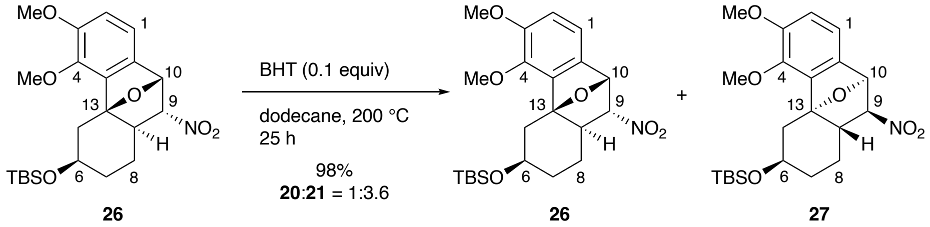

Isomerization of Cycloadduct 26. A mixture of cycloadduct **26** (40.9 mg, 93.9 μmol) and BHT (2.0 mg, 9.2 μmol) in *n*-dodecane (2 mL) was heated at 200 °C for 25 h. After cooling, the mixture was purified by column chromatography (silica gel 10 g, *n*-hexane → *n*-hexane/AcOEt 5:1) to give a mixture of hydrophenanthrenes **26** and **27** (40.1 mg, 98%) as a pale orange solid. The diastereomeric ratio (**26**:**27**) was determined to be 1:3.6 by ^{1}H NMR.

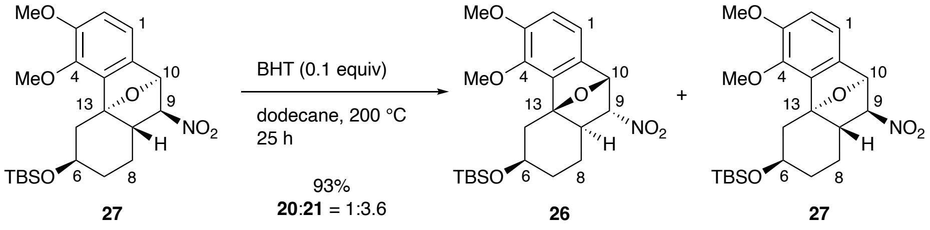

Isomerization of Cycloadduct 27. A mixture of cycloadduct **27** (40.5 mg, 93.0 μmol) and BHT (2.0 mg, 9.2 μmol) in *n*-dodecane (2 mL) was heated at 200 °C for 25 h. After cooling, the mixture was purified by column chromatography (silica gel 10 g, *n*-hexane → *n*-hexane/AcOEt 5:1) to give a mixture of hydrophenanthrenes **26** and **27** (37.8 mg, 93%) as a pale orange solid. The diastereomeric

ratio (**26**:**27**) was determined to be 1:3.6 by ^{1}H NMR.

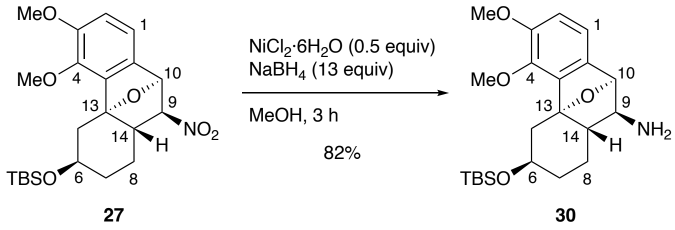

(4b*S,6*R**,8a*S**,9*S**,10*S**)-9-Amino-6-(*tert*-butyldimethylsilyl)oxy-3,4-dimethoxy-4b,5,6,7,8,8a,9,10-octahydro-4b,10-epoxyphenanthrene (30).** A mixture of $NiCl_2 \cdot 6H_2O$ (86.6 mg, 0.364 mmol) and $NaBH_4$ (41.5 mg, 1.10 mmol) in MeOH (15 mL) was sonicated for 30 min. To the black suspention of Ni_2B was added a solution of nitro compound **27** (312 mg, 0.716 mmol) in MeOH (5 mL), followed by $NaBH_4$ (300 mg, 7.93 mmol). After 3 h of stirring, the reaction was quenched with 28% aqueous NH_3 (10 mL), and the resulting mixture was filtered through a Celite pad. The filtrate was extracted with AcOEt (2 × 50 mL), and the combined organic extracts were washed with brine (2 × 100 mL) and dried over anhydrous Na_2SO_4. Filtration and evaporation in vacuo furnished the crude product (318 mg, yellow oil), which was purified by column chromatography (silica gel 10 g, CH_2Cl_2/MeOH 20:1) to give amine **30** (237 mg, 82%) as a colorless oil. R_f = 0.42 (CH_2Cl_2/MeOH 10:1); IR (neat) ν_{max}/cm^{-1} 3373, 2929, 2856, 1483, 1463, 1382, 1259, 1095, 1002, 962; δ_H (500 MHz; $CDCl_3$) 7.00 (1 H, d, *J* = 8.0 Hz, Ar*H*), 6.72 (1 H, d, *J* = 8.0 Hz, Ar*H*), 5.01 (1 H, d, *J* = 4.6 Hz, C(10)*H*), 3.88 (1 H, m, C(6)*H*), 3.85 (3 H, s, OCH_3), 3.84 (3 H, s, OCH_3), 3.23 (1 H, dd, *J* = 2.3, 4.6 Hz, C(9)*H*), 2.80 (1 H, dd, *J* = 10.9, 14.3 Hz, one of C(5)H_2), 2.47 (1 H, ddd, *J* = 2.3, 4.6, 14.3 Hz, one of C(5)H_2), 2.17 (1 H, ddd, *J* = 4.0, 6.3, 10.3 Hz, one of C(8)H_2), 1.88 (1 H, m, one of C(7)H_2), 1.49−1.38 (2 H, m, one of C(7)H_2, one of C(8)H_2), 1.12 (1 H, ddd, *J* = 2.3, 6.3, 10.9 Hz, C(14)*H*), 0.91 (9 H, s, $SiC(CH_3)_3$), 0.08 (6 H, s, $Si(CH_3)_2$); δ_C (126 MHz; $CDCl_3$) 153.1 (C), 143.3 (C), 139.1 (C), 135.7 (C), 117.5 (CH), 110.5 (CH), 90.3 (C), 82.2 (CH), 68.5 (CH), 61.4 (CH_3), 61.2 (CH), 56.3 (CH_3), 49.0 (CH), 38.1 (CH_2), 35.0 (CH_2), 29.5 (CH_2), 26.1 (3 × CH_3), 18.4 (C), −4.4 (CH_3), −4.5 (CH_3); HRMS (ESI) *m*/*z* $[M + H]^+$ calcd for $C_{22}H_{36}NO_4Si$ 406.2408; found 406.2403.

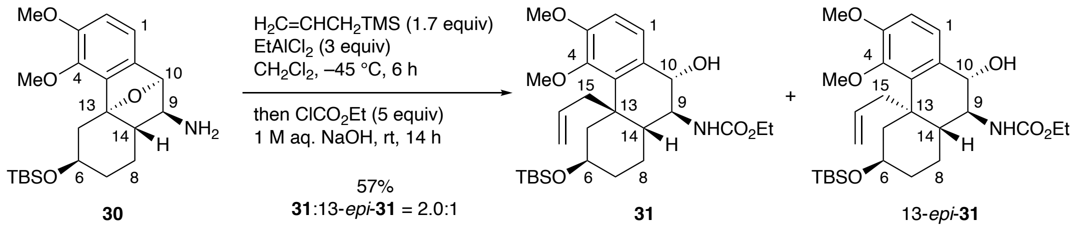

Ethyl [(4b*S,6*R**,8a*R**,9*S**,10*S**)-4b-Allyl-6-(*tert*-butyldimethylsilyl)oxy-10-hydroxy-3,4-dimethoxy-4b,5,6,7,8,8a,9,10-octahydrophenanthren-9-yl]carbamate (31).** $EtAlCl_2$ in *n*-hexane (1.0 M, 0.45 mL, 0.45 mmol) was added to a cooled (−45 °C) mixture of amine **30** (59.8 mg, 0.147 mmol) and allyltrimethylsilane (40 μL, 0.252 mmol) in CH_2Cl_2 (1 mL). After 6 h of stirring, 1 M aqueous NaOH (1 mL) was added, and the mixture was allowed to warm to room temperature. Ethyl chloroformate (70 μL, 0.735 mmol) was added, and the resulting mixture was stirred for 14 h. The resulting mixture was extracted with AcOEt (10 mL), and the organic extract was successively washed with H_2O (5 mL) and brine (2 × 5 mL) and dried over anhydrous Na_2SO_4. Filtration and evaporation in vacuo furnished the crude product (79.0 mg, yellow solid), which was purified by column chromatography (silica gel 10 g, *n*-hexane/AcOEt 3:1) to give alcohol **31** (28.8 mg, 38%) and 13-*epi*-**31** (14.8 mg, 19%) as white solids. R_f = 0.24 (*n*-hexane/AcOEt 1:1); IR (KBr) ν_{max}/cm^{-1} 3439, 2953, 2928, 1697, 1524, 1473, 1375, 1290, 1053, 910, 835; δ_H (500 MHz; $CDCl_3$) 7.37 (1 H, d, *J* = 8.6 Hz, Ar*H*), 6.87 (1 H, d, *J* = 8.6 Hz, Ar*H*), 5.20 (1 H, ddt, *J* = 3.5, 16.9, 10.0 Hz, C(16)*H*), 4.94 (1 H, d, *J* = 16.9 Hz, (*E*)-CH=CH_2), 4.82 (1 H, d, *J* = 10.0 Hz, (*Z*)-CH=CH_2), 4.60 (1 H, d, *J* = 9.3 Hz, C(9)N*H*CO_2Et), 4.32 (1 H, d, *J* = 9.5 Hz, C(10)*H*), 4.15 (2 H, q, *J* = 6.9 Hz, $CO_2CH_2CH_3$), 4.05 (1 H, br s, C(6)*H*), 3.88−3.82 (7 H, m, 2 × OCH_3, C(9)*H*), 3.32 (1 H, br d, *J* = 15.2 Hz, one of C(15)H_2), 3.11 (1 H, dd, *J* = 10.0, 15.2 Hz, one of C(15)H_2), 2.49 (1 H, br d, *J* = 14.3 Hz, one of C(5)H_2), 2.16 (1 H, br t, *J* = 13.8 Hz, one of C(8)H_2), 1.89 (1 H, br d, *J* = 11.8 Hz, C(14)*H*), 1.78 (1 H, br d, *J* = 14.3 Hz, one of C(5)H_2), 1.64 (1 H, br t, *J* = 13.8 Hz, one of C(7)H_2), 1.57 (1 H, br d, *J* = 13.8 Hz, one of C(8)H_2), 1.43 (1 H, br d, *J* = 13.8 Hz, one of C(7)H_2), 1.24 (3 H, t, *J* = 6.9 Hz, $CO_2CH_2CH_3$), 0.93 (9 H, s, SiC$(CH_3)_3$), 0.10 (3 H, s, SiCH_3), 0.05 (3 H, s, SiCH_3); δ_C (126 MHz; $CDCl_3$) 158.0 (C), 152.4 (C), 147.2 (C), 137.6 (CH), 136.0 (C), 131.6 (C), 122.0 (CH), 116.5 (CH_2), 111.3 (CH), 75.0 (CH), 67.6 (CH), 61.3 (CH_2), 60.3 (CH_3), 55.9 (CH_3), 53.2 (CH), 43.7 (C), 41.5 (CH_2), 39.5 (CH_2), 38.7 (CH), 28.1 (CH_2), 26.0 (3 × CH_3), 18.2 (C), 16.8 (CH_2), 14.7 (CH_3), −4.7 (CH_3), −4.9 (CH_3); HRMS (ESI) *m*/*z* $[M + Na]^+$ calcd for $C_{28}H_{45}NO_6SiNa$ 542.2908; found 542.2906.

Data for ethyl [(4b*R**,6*R**,8a*R**,9*S**,10*S**)-4b-allyl-6-(*tert*-butyldimethylsilyl)oxy-10-hydroxy-3,4-dimethoxy-4b,5,6,7,8,8a,9,10-octahydrophenanthren-9-yl]carbamate (13-*epi*-**31**). R_f = 0.21 (*n*-hex-

ane/AcOEt 1:1); mp 185.8−190.8 °C (colorless needles from *n*-hexane/$CHCl_3$); IR (KBr) ν_{max}/cm^{-1} 3431, 2954, 2934, 1688, 1541, 1476, 1290, 1260, 1086, 1024, 775; δ_H (500 MHz; $CDCl_3$) 7.31 (1 H, d, J = 8.6 Hz, Ar*H*), 6.90 (1 H, d, J = 8.6 Hz, Ar*H*), 5.56 (1 H, m, C(16)*H*), 5.05 (1 H, d, J = 16.9 Hz, (*E*)-CH=CH_2), 4.94 (1 H, d, J = 10.1 Hz, (*Z*)-CH=CH_2), 4.58 (1 H, d, J = 8.9 Hz, C(9)N*H*CO_2Et), 4.46 (1 H, dd, J = 5.4, 8.9 Hz, C(10)*H*), 4.14 (2 H, q, J = 7.1 Hz, $CO_2CH_2CH_3$), 4.06 (1 H, dt, J = 11.2, 8.9 Hz, C(9)*H*), 3.92 (1 H, m, C(6)*H*), 3.87 (3 H, s, OCH_3), 3.82 (3 H, s, OCH_3), 3.33 (1 H, dd, J = 1.9, 12.7 Hz, one of C(5)H_2), 2.70 (1 H, d, J = 5.4 Hz, C(10)O*H*), 2.69−2.59 (2 H, m, C(15)H_2), 2.02 (1 H, m, one of C(7)H_2), 1.83 (1 H, m, one of C(8)H_2), 1.62−1.56 (2 H, m, one of C(8)H_2, C(14)*H*), 1.32−1.24 (5 H, m, one of C(5)H_2, one of C(7)H_2, $CO_2CH_2CH_3$), 0.90 (9 H, s, SiC(CH_3)$_3$), 0.09 (6 H, s, Si(CH_3)$_2$); δ_C (126 MHz; $CDCl_3$) 157.8 (C), 152.8 (C), 147.5 (C), 136.6 (CH), 136.4 (C), 131.2 (C), 124.1 (CH), 117.0 (CH_2), 111.6 (CH), 76.0 (CH), 67.6 (CH), 61.3 (CH_2), 60.7 (CH_3), 55.9 (CH_3), 55.3 (CH), 47.0 (CH), 45.5 (C), 45.2 (CH_2), 38.8 (CH_2), 35.6 (CH_2), 26.0 (3 × CH_3), 22.6 (CH_2), 18.4 (C), 14.8 (CH_3), −4.5 (2 × CH_3); HRMS (ESI) *m*/*z* $[M + Na]^+$ calcd for $C_{28}H_{45}NO_6SiNa$ 542.2908; found 542.2903.

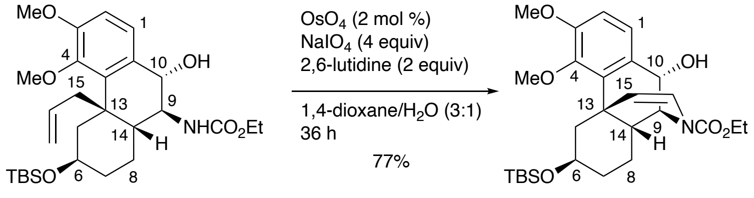

31 **32**

Ethyl (4b*R,6*R**,8a*R**,9*S**,10*S**)-6-(*tert*-Butyldimethylsilyl)oxy-10-hydroxy-3,4-dimethoxy-6,7,8,8a,9,10-hexahydro-5*H*-9,4b-(epiminoetheno)phenanthrene-11-carboxylate (32).** A 0.157 M solution of OsO_4 in *t*-BuOH (10 μL, 1.6 μmol) was added to a mixture of alkene **31** (39.5 mg, 76.0 μmol), $NaIO_4$ (65.5 mg, 0.306 mmol), and 2,6-lutidine (20 μL, 173 μmol) in dioxane/H_2O (3:1, 0.8 mL). After 36 h of stirring, the reaction was quenched with 1 M aqueous $Na_2S_2O_3$ (1 mL), and the resulting mixture was extracted with AcOEt (10 mL). The organic extract was washed with brine (2 × 10 mL) and dried over anhydrous Na_2SO_4. Filtration and evaporation in vacuo furnished the crude product (51.2 mg, yellow oil), which was purified by column chromatography (silica gel 4 g, *n*-hexane/AcOEt 2:3) to give encarbamate **32** (29.6 mg, 77%) as a white solid. R_f = 0.77 (*n*-hexane/AcOEt 1:3); IR (KBr) ν_{max}/cm^{-1} 3435, 3320, 2930, 2858, 1697, 1672, 1483, 1283, 1098, 835; δ_H (500 MHz; $CDCl_3$; spectrum contains a 2:1 mixture of rotamers) 7.22 (0.67 H, d, J = 8.6 Hz, Ar*H*), 7.20 (0.33 H,

d, J = 8.6 Hz, ArH), 6.84 (0.67 H, d, J = 8.6 Hz, ArH), 6.83 (0.33 H, d, J = 8.6 Hz, ArH), 6.68 (0.33 H, d, J = 8.0 Hz, C(16)H), 6.56 (0.67 H, d, J = 8.0 Hz, C(16)H), 5.05 (0.33 H, d, J = 8.0 Hz, C(15)H), 4.98 (0.67 H, d, J = 8.0 Hz, C(15)H), 4.50 (0.67 H, br s, C(10)H), 4.47 (0.33 H, br s, C(10)H), 4.42 (0.67 H, d, J = 2.3 Hz, C(9)H), 4.38 (0.33 H, d, J = 2.3 Hz, C(9)H), 4.31−4.24 (0.67 H, m, $CO_2CH_2CH_3$), 4.20 (0.67 H, dq, J = 10.9, 7.4 Hz, one of $CO_2CH_2CH_3$), 4.18 (0.67 H, dq, J = 10.9, 7.4 Hz, one of $CO_2CH_2CH_3$), 3.842 (1 H, s, OCH_3), 3.838 (2 H, s, OCH_3), 3.80 (3 H, s, OCH_3), 3.58 (1 H, tt, J = 4.0, 10.9 Hz, C(6)H), 3.44 (1 H, m, one of C(5)H_2), 1.87 (1 H, m, one of C(7)H_2), 1.79−1.70 (2 H, m, one of C(8)H_2, C(14)H), 1.64 (0.67 H, dq, J = 3.4, 13.7 Hz, one of C(8)H_2), 1.56 (0.33 H, dq, J = 3.4, 13.7 Hz, one of C(8)H_2), 1.38−1.24 (5 H, m, one of C(5)H_2, one of C(7)H_2, $CO_2CH_2CH_3$), 0.87 (9 H, s, $SiC(CH_3)_3$), 0.06 (1 H, s, $SiCH_3$), 0.05 (2 H, s, $SiCH_3$), 0.01 (3 H, s, $SiCH_3$); δ_C (126 MHz; $CDCl_3$; spectrum contains a 2:1 mixture of rotamers, only the major rotamer signals are reported) 171.2 (C), 152.8 (C), 148.0 (C), 133.6 (C), 129.2 (C), 127.0 (CH), 120.6 (CH), 115.5 (CH), 111.4 (CH), 70.9 (CH), 68.3 (CH), 62.2 (CH_2), 60.4 (CH_3), 57.0 (CH), 56.1 (CH_3), 44.1 (CH_2), 40.6 (CH), 38.0 (C), 36.6 (CH_2), 25.9 (3 × CH_3), 25.5 (CH_2), 18.0 (C), 14.2 (CH_3), −4.4 (CH_3), −4.5 (CH_3); HRMS (ESI) m/z $[M + Na]^+$ calcd for $C_{27}H_{41}NO_6SiNa$ 526.2595; found 526.2598.

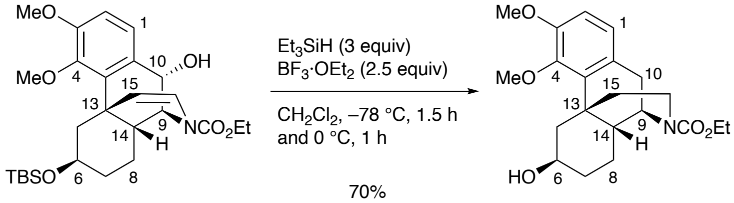

Ethyl (4bS*,6R*,8aR*,9R*)-6-Hydroxy-3,4-dimethoxy-6,7,8,8a,9,10-hexahydro-5H-9,4b-(epiminoethano)phenanthrene-11-carboxylate (33). $BF_3·OEt_2$ (20 μL, 0.159 mmol) was added to a cooled (−78 °C) mixture of encarbamate **32** (31.5 mg, 62.5 μmol) and triethylsilane (30 μL, 0.188 mmol) in CH_2Cl_2 (1 mL). After 1.5 h of stirring, the reaction mixture was allowed to warm to 0 °C, and stirred for another 1 h. The reaction was quenched with saturated aqueous $NaHCO_3$ (5 mL), and the resulting mixture was extracted with AcOEt (30 mL). The organic extract was successively washed with brine (2 × 20 mL) and dried over anhydrous Na_2SO_4. Filtration and evaporation in vacuo furnished the crude product (19.8 mg), which was purified by column chromatography (silica gel 3 g, n-hexane/AcOEt 2:3) to give alcohol **33** (16.5 mg, 70%) as a white solid. R_f = 0.46 (n-hex-

ane/AcOEt 1:1); mp 188.0−189.0 °C (colorless prisms from acetone); IR (KBr) ν_{max}/cm^{-1} 3448, 2926, 2847, 1664, 1481, 1440, 1330, 1275, 1049, 1026; δ_H (500 MHz; $CDCl_3$) 6.77 (2 H, s, Ar*H*), 4.40 (1 H, br s, C(6)O*H*), 4.28 (1 H, m, C(9)*H*), 4.18−4.09 (2 H, m, OCH_2CH_3), 3.86 (3 H, s, OCH_3), 3.84 (1 H, m, one of C(16)H_2), 3.83 (3 H, s, OCH_3), 3.66 (1 H, ddt, *J* = 2.9, 4.5, 11.5 Hz, C(6)*H*), 3.48 (1 H, ddd, *J* = 2.7, 2.9, 12.5 Hz, one of C(5)H_2), 3.11 (1 H, dd, *J* = 6.0, 18.1 Hz, one of C(10)H_2), 2.62 (1 H, d, *J* = 18.1 Hz, one of C(10)H_2), 2.58 (1 H, m, one of C(16)H_2), 1.96 (1 H, m, one of C(7)H_2), 1.75 (1 H, d, *J* =13.3 Hz, one of C(8)H_2), 1.61−1.50 (3 H, m, one of C(8)H_2, C(14)*H*, one of C(15)H_2), 1.38−1.22 (5 H, m, one of C(7)H_2, one of C(15)H_2, OCH_2CH_3), 1.18 (1 H, dd, *J* = 11.5, 12.5 Hz, one of C(5)H_2); δ_C (126 MHz; $CDCl_3$) 151.6 (C), 149.0 (C), 131.7 (C), 123.6 (CH), 111.4 (CH), 67.8 (CH), 61.2 (CH_2), 60.1 (CH_3), 55.9 (CH_3), 46.4 (CH_2), 45.0 (CH), 39.4 (CH_2), 38.9 (C), 37.9 (CH_2), 35.7 (CH_2), 32.0 (CH_2), 25.8 (CH_2), 14.7 (CH_3) [three carbons were not detected]; HRMS (ESI) *m*/*z* [M + Na]$^+$ calcd for $C_{21}H_{29}NO_5Na$ 398.1938; found 398.1952.

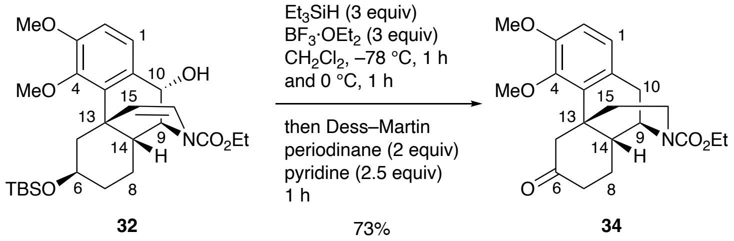

Ethyl (4b*S,8a*R**,9*R**)-3,4-Dimethoxy-6-oxo-6,7,8,8a,9,10-hexahydro-5*H*-9,4b-(epiminoethano)phenanthrene-11-carboxylate (34).** $BF_3 \cdot OEt_2$ (20 µL, 0.159 mmol) was added to a cooled (−78 °C) mixture of encarbamate **32** (25.5 mg, 50.6 µmol) and triethylsilane (25 µL, 0.157 mmol) in CH_2Cl_2 (1 mL). After 1 h of stirring, the reaction mixture was allowed to warm to 0 °C and stirred for another 1 h. Pyridine (10 µL, 0.124 mmol) and Dess−Martin periodinane[57] (42.9 mg, 0.101 mmol) were added, and the mixture was stirred for 1 h. The reaction was quenched by addition of a mixture of 1 M aqueous $Na_2S_2O_3$ (5 mL) and saturated aqueous $NaHCO_3$ (5 mL), and the resulting mixture was extracted with AcOEt (20 mL). The organic extract was successively washed with 1 M aqueous hydrochloric acid (2 × 10 mL), H_2O (10 mL), saturated aqueous $NaHCO_3$ (10 mL), and brine (2 × 10 mL), and dried over anhydrous Na_2SO_4. Filtration and evaporation in vacuo furnished the crude product (21.5 mg, orange solid), which was purified by column chromatography (silica gel 2 g, *n*-hexane/AcOEt 3:2) to give ketone **34** (13.9 mg, 73%) as a white solid. R_f = 0.50 (*n*-hexane/ AcOEt 1:1); IR (KBr) ν_{max}/cm^{-1} 2893, 2881, 1717, 1684, 1418, 1277, 1131, 1055, 910, 768; δ_H (500

MHz; 50 °C; $CDCl_3$; spectrum contains a 2:1 mixture of rotamers) 6.76 (2 H, s, Ar*H*), 4.56 (0.66 H, br s, C(9)*H*), 4.42 (0.34 H, br s, C(9)*H*), 4.16 (2 H, m, OCH_2CH_3), 3.99 (1 H, dd, J = 2.1, 14.1 Hz, one of C(5)H_2), 3.96 (3 H, s, OCH_3), 3.85 (1 H, m, one of C(16)H_2), 3.81 (3 H, s, OCH_3), 3.17 (1 H, dd, J = 5.9, 18.2 Hz, one of C(10)H_2), 2.66 (1 H, d, J = 18.2 Hz, one of C(10)H_2), 2.54 (1 H, m, one of C(16)H_2), 2.42 (1 H, dt, J = 6.7, 14.0 Hz, one of C(7)H_2), 2.29 (1 H, ddd, J = 2.1, 2.5, 4.7, 14.0 Hz, one of C(7)H_2), 2.26 (1 H, br d, J = 14.1 Hz, one of C(5)H_2), 2.04 (1 H, dt, J = 12.6, 3.2 Hz, C(14)*H*), 1.97−1.86 (2 H, m, one of C(8)H_2, one of C(15)H_2), 1.72−1.61 (2 H, m, one of C(8)H_2, one of C(15)H_2), 1.27 (3 H, m, OCH_2CH_3); δ_C (150 MHz; 50 °C; $CDCl_3$; spectrum contains a 2:1 mixture of rotamers, only the major rotamer signals are reported) 209.6 (C), 155.8 (C), 151.9 (C), 149.2 (C), 129.4 (C), 129.0 (C), 123.4 (CH), 112.1 (CH), 61.3 (CH_2), 60.4 (CH_3), 55.9 (CH_3), 51.0 (CH_2), 49.0 (CH), 44.4 (CH), 41.9 (C), 41.0 (CH_2), 39.6 (CH_2), 37.8 (CH_2), 31.8 (CH_2), 26.9 (CH_2), 14.7 (CH_3); HRMS (ESI) *m*/*z* $[M + Na]^+$ calcd for $C_{21}H_{27}NO_5Na$ 396.1781; found 396.1757.

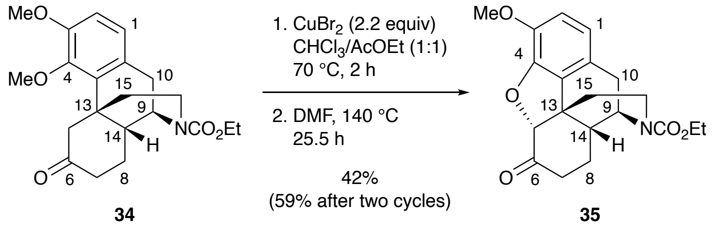

Ethyl (4*R,4a*R**,7a*R**,12b*S**)-9-Methoxy-7-oxo-2,3,4,4a,5,6,7,7a-octahydro-1*H*-4,12-methanobenzofuro[3,2-*e*]isoquinoline-3-carboxylate (35).** Copper(II) bromide (16.3 mg, 73.0 μmol) was added to a solution of ketone **34** (12.4 mg, 33.2 μmol) in 1:1 $CHCl_3$/AcOEt (1 mL). After 2 h of stirring at 70 °C, the reaction was quenched with saturated aqueous NH_4Cl (5 mL), and the resulting mixture was extracted with AcOEt (10 mL). The organic extract was washed with brine (2 × 10 mL), and dried over anhydrous Na_2SO_4. Filtration and evaporation in vacuo furnished the crude α-bromoketone (16.8 mg), which was used without further purification.

The crude α-bromoketone (16.8 mg) was dissolved in DMF (1 mL), and heated at 140 °C for 25.5 h. The solvent was removed in vacuo, and the residue was purified by column chromatography (silica gel 2 g, *n*-hexane/AcOEt 2:1) to give ether **35** (5.0 mg, 42%) as a yellow solid, along with recovered ketone **34** (6.5 mg, 52%). This sequence was repeated employing recovered ketone **34** (6.5 mg) to give ether **35** (2.0 mg, 17%) as a yellow solid. R_f = 0.37 (*n*-hexane/AcOEt 1:1); IR (KBr) ν_{max}/cm^{-1} 2953, 2884, 1732, 1695, 1462, 1377, 1244, 1209, 1182, 1167, 1140, 1084, 1057, 1011,

980; δ_H (500 MHz; $CDCl_3$; spectrum contains a 1:1 mixture of rotamers) 6.72 (1 H, d, *J* = 8.0 Hz, Ar*H*), 6.63 (1 H, d, *J* = 8.0 Hz, Ar*H*), 4.84 (0.5 H, br s, C(9)*H*), 4.65 (1 H, s, C(5)*H*), 4.61 (0.5 H, br s, C(9)*H*), 4.23−4.13 (2 H, m, OCH_2CH_3), 3.94 (1 H, m, one of C(16)H_2), 3.90 (3 H, s, OCH_3), 2.84 (1 H, dt, *J* = 4.6, 12.6 Hz, one of C(16)H_2), 2.76 (1 H, m, one of C(10)H_2), 2.70 (1 H, d, *J* = 18.3 Hz, one of C(10)H_2), 2.46−2.32 (3 H, m, C(7)H_2, C(14)*H*), 1.93 (1 H, dt, *J* = 5.2, 12.6 Hz, one of C(15)H_2), 1.91 (1 H, m, one of C(15)H_2), 1.85 (1 H, m, one of C(8)H_2), 1.30−1.25 (3 H, m, OCH_2CH_3), 1.21 (1 H, dq, *J* = 3.4, 13.8 Hz, one of C(8)H_2); δ_H (500 MHz; 80 °C; pyridine-d_5) 6.90 (1 H, d, *J* = 8.0 Hz, Ar*H*), 6.71 (1 H, d, *J* = 8.0 Hz, Ar*H*), 4.89 (1 H, s, C(5)*H*), 4.87 (1 H, br s, C(9)*H*), 4.35−4.28 (2 H, m, OCH_2CH_3), 4.12 (1 H, m, one of C(16)H_2), 3.98 (3 H, s, OCH_3), 2.88−2.81 (2 H, m, one of C(10)H_2, one of C(16)H_2), 2.74 (1 H, d, *J* = 18.3 Hz, one of C(10)H_2), 2.46−2.32 (3 H, m, C(7)H_2, C(14)*H*), 1.98 (1 H, dt, *J* = 5.7, 12.6 Hz, one of C(15)H_2), 1.79 (1 H, m, one of C(15)H_2), 1.73 (1 H, m, one of C(8)H_2), 1.30 (3 H, t, *J* = 6.9 Hz, OCH_2CH_3), 1.11 (1 H, dq, *J* = 3.4, 13.5 Hz, one of C(8)H_2); δ_C (150 MHz; 50 °C; $CDCl_3$) 207.1 (C), 155.6 (C), 145.5 (C), 143.1 (C), 126.1 (C), 125.0 (C), 120.3 (CH), 114.9 (CH), 91.2 (CH), 61.6 (CH_2), 56.7 (CH_3), 50.7 (CH), 47.3 (C), 41.4 (CH), 39.9 (CH_2), 37.9 (CH_2), 35.1 (CH_2), 28.5 (CH_2), 25.4 (CH_2), 14.7 (CH_3); HRMS (ESI) *m*/*z* $[M + Na]^+$ calcd for $C_{20}H_{23}NO_5Na$ 380.1468; found 380.1455.

3. X-Ray Chromatographic Information of Alcohol 33

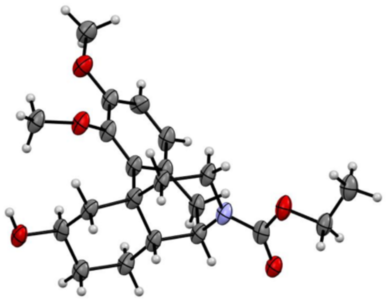

CCDC No.		2237321
formula		$C_{21}H_{29}NO_5$
formula weight [g/mol]		375.47
crystal size [mm^3]		0.297 × 0.218 × 0.096
crystal system		triclinic
space group		P$\bar{1}$
Unit cell dimensions	a [Å]	7.6082(4)
	b [Å]	11.0116(11)
	c [Å]	10.8744(13)
	α [Å]	89.490(9)
	β [Å]	98.254(7)
	γ [Å]	89.986(6)
volume [Å^3]		901.57(15)
T [K]		103
Z		2
μ(Cu Kα) [mm^{-1}]		0.098
Dcalc [g/cm^3]		1.383
Collected reflections		6935 (3.698° ≤ 2Θ ≤ 51.000°)
Independent reflections		3335 (R_{int} = 0.1089, R_{sigma} = 0.0590)
Goodness-of-fit on F2		1.322
Final R indices [I>2sigma(I)]	R_1	0.1083
	wR2	0.3177

4. Computational Analysis

Conformational searches were carried out using Spartan '20 (Wavefunction Inc.) with the MMFF force field without solvent. All conformations within 40 kJ/mol of the lowest energy structure were recorded at most 500 conformers. The structure of the most stable conformer for each molecule was optimized further at the DFT level with the M06-2X functional and the 6-311+G** basis set for all atoms.

The subsequent DFT calculations for the transition state were performed using the Gaussian 16, Revision C.01 software. Structure optimization and frequency calculation were carried out with the B3LYP functional and the 6-31G* basis set for all atoms. Single point energy was obtained via calculation of the B3LYP/6-31G* geometries with the B3LYP functional and the 6-311++G** basis set. Gibbs free energy (kcal/mol) was calculated based on B3LYP/6-311++G** single point energy and B3LYP/6-31G* frequency. Solvent effects were corrected by using Self-Consistent Reaction Field (SCRF) method using the Polarizable Continuum Model (PCM) together with heptane as a solvent in the single point energy calculations. All stationary points were characterized by frequency calculations to confirm their identity as either of the local minima (zero imaginary frequencies) or first-order saddle points (one imaginary frequency). For transition structures, an intrinsic reaction coordinate calculation (IRC) was conducted to ensure transit between desired reactant and product minima.

(PCM(heptane))/B3LYP/6-311++G**//B3LYP/6-31G*

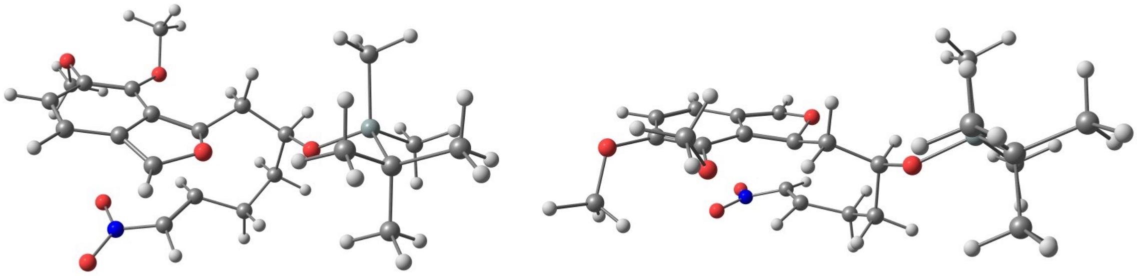

Minor TS **28** Major TS **29**

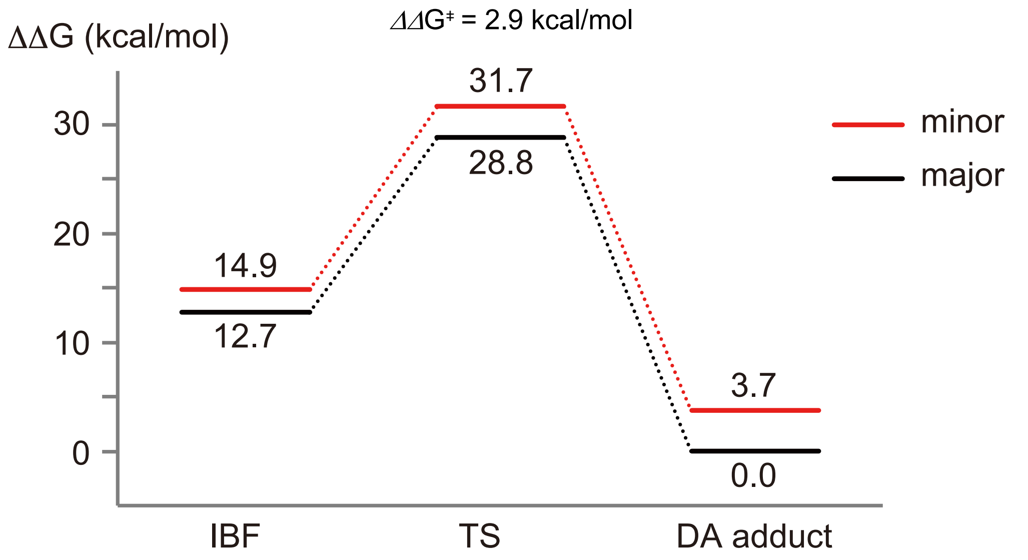

Figure S1. Calculated transition state structures **28** and **29**, and energy profile for Diels−Alder reaction at 200 °C. Energies are in kcal/mol.

Table S1. Calculated energies in Hartrees (B3LYP/6-31G*).

Structure	Hartrees
major IBF	−1653.73434353
minor IBF	−1653.73097126
major TS **29**	−1653.70878068
minor TS **28**	−1653.70417317
major DA adduct	−1653.75465720
minor DA adduct	−1653.74871038

Cartesian Coordinates of Calculated Structures

major IBF

H	4.761779000000	-0.875359000000	2.543636000000
C	4.160446000000	-0.226011000000	1.915490000000
C	2.591517000000	1.511747000000	0.230530000000
C	2.762315000000	-0.462269000000	1.746693000000
C	4.718387000000	0.823290000000	1.242335000000
C	3.948730000000	1.682564000000	0.386834000000
C	1.968100000000	0.413804000000	0.904827000000
H	5.779376000000	1.040779000000	1.318727000000
C	1.889569000000	-1.415157000000	2.220123000000
H	1.995074000000	-2.272440000000	2.866567000000
C	2.255023000000	-2.677222000000	-0.830562000000
H	2.097494000000	-3.579049000000	-0.255077000000
C	1.320417000000	-1.955734000000	-1.454491000000
H	1.643477000000	-1.069549000000	-1.995719000000
C	0.672958000000	-0.072956000000	0.930910000000
O	1.815957000000	2.295381000000	-0.594760000000
O	4.630390000000	2.689599000000	-0.267671000000
C	4.828521000000	2.436667000000	-1.666018000000
H	3.874230000000	2.423387000000	-2.204374000000
H	5.451689000000	3.253916000000	-2.037340000000
H	5.340771000000	1.479447000000	-1.818059000000
C	1.763753000000	3.688763000000	-0.256323000000
H	1.092907000000	4.149251000000	-0.985135000000
H	2.754276000000	4.147463000000	-0.313167000000
H	1.354693000000	3.824167000000	0.752781000000
O	0.639597000000	-1.172190000000	1.745125000000
C	-0.121980000000	-2.358055000000	-1.549884000000
H	-0.363856000000	-3.097665000000	-0.775897000000
H	-0.229117000000	-2.885231000000	-2.511172000000
C	-0.633504000000	0.423965000000	0.412540000000
H	-1.190146000000	0.906100000000	1.229773000000
H	-0.433752000000	1.201561000000	-0.329727000000
C	-1.584442000000	-0.629576000000	-0.192850000000
H	-1.697221000000	-1.456015000000	0.525497000000
C	-1.161813000000	-1.218502000000	-1.552356000000
H	-2.079390000000	-1.618588000000	-1.996557000000

H	-0.832177000000	-0.405048000000	-2.212420000000
O	-2.837756000000	0.028588000000	-0.396280000000
Si	-4.284753000000	-0.326281000000	0.384713000000
C	-5.531902000000	0.926231000000	-0.341552000000
C	-4.787860000000	-2.116210000000	0.027438000000
H	-4.924942000000	-2.296661000000	-1.044757000000
H	-5.726829000000	-2.374129000000	0.532182000000
H	-4.026777000000	-2.820125000000	0.386741000000
C	-4.081290000000	-0.126750000000	2.255493000000
H	-5.006621000000	-0.382800000000	2.785826000000
H	-3.811440000000	0.898859000000	2.531399000000
H	-3.295561000000	-0.788761000000	2.640318000000
C	-6.924526000000	0.713925000000	0.291054000000
H	-7.645134000000	1.433476000000	-0.123595000000
H	-6.910849000000	0.860086000000	1.378364000000
H	-7.319553000000	-0.290234000000	0.092498000000
C	-5.055994000000	2.365777000000	-0.049298000000
H	-4.066112000000	2.556912000000	-0.477960000000
H	-5.001535000000	2.569001000000	1.027344000000
H	-5.756043000000	3.094676000000	-0.483085000000
C	-5.634313000000	0.735283000000	-1.870291000000
H	-6.335194000000	1.465708000000	-2.300134000000
H	-6.003360000000	-0.263323000000	-2.135115000000
H	-4.663959000000	0.875840000000	-2.359021000000
N	3.656473000000	-2.296039000000	-0.875880000000
O	4.435705000000	-3.019798000000	-0.249663000000
O	3.990152000000	-1.302442000000	-1.525755000000

minor IBF

H	-3.540417000000	2.209235000000	-2.447763000000
C	-3.382017000000	1.288386000000	-1.895664000000
C	-2.988225000000	-1.152566000000	-0.413687000000
C	-2.061903000000	0.838385000000	-1.592037000000
C	-4.433775000000	0.539411000000	-1.450412000000
C	-4.246155000000	-0.668052000000	-0.696703000000
C	-1.857004000000	-0.391308000000	-0.849358000000
H	-5.459945000000	0.838764000000	-1.640391000000
C	-0.802393000000	1.328733000000	-1.850585000000

C	-0.760550000000	2.397342000000	1.238363000000
C	-0.685400000000	1.234193000000	1.890833000000
C	-0.487141000000	-0.553527000000	-0.714574000000
O	-2.766269000000	-2.300533000000	0.319087000000
O	-5.384740000000	-1.334201000000	-0.283448000000
C	-5.664226000000	-1.193261000000	1.114507000000
H	-6.613666000000	-1.705362000000	1.289197000000
H	-5.761545000000	-0.135227000000	1.387178000000
H	-4.878387000000	-1.653327000000	1.724512000000
C	-3.276911000000	-3.509405000000	-0.261872000000
H	-2.821063000000	-3.686716000000	-1.244116000000
H	-4.364118000000	-3.469691000000	-0.366459000000
H	-2.993947000000	-4.313853000000	0.421202000000
C	0.593256000000	0.656823000000	2.417910000000
H	0.597848000000	0.872244000000	3.498150000000
C	0.357218000000	-1.666036000000	-0.195139000000
H	-0.329724000000	-2.437808000000	0.164676000000
C	1.375697000000	-1.377113000000	0.931445000000
H	1.816310000000	-2.361976000000	1.157777000000
C	0.789420000000	-0.866583000000	2.267724000000
H	1.492820000000	-1.169372000000	3.052490000000
H	-0.150201000000	-1.396974000000	2.471478000000
O	2.391514000000	-0.487273000000	0.484361000000
Si	3.972690000000	-0.896885000000	0.075457000000
C	4.714821000000	0.707943000000	-0.647979000000
C	4.892715000000	-1.473797000000	1.625805000000
H	4.938209000000	-0.688165000000	2.388463000000
H	5.921356000000	-1.774824000000	1.393230000000
H	4.397072000000	-2.342736000000	2.076792000000
C	3.985037000000	-2.307916000000	-1.186241000000
H	3.454518000000	-2.038731000000	-2.106276000000
H	3.511588000000	-3.211008000000	-0.781419000000
H	5.011486000000	-2.580461000000	-1.460756000000
C	6.203749000000	0.490194000000	-0.995851000000
H	6.631982000000	1.407222000000	-1.425375000000
H	6.342828000000	-0.308174000000	-1.735567000000
H	6.801833000000	0.236540000000	-0.111767000000
C	3.945894000000	1.104732000000	-1.927303000000
H	4.333347000000	2.053949000000	-2.325485000000

H	2.875311000000	1.231015000000	-1.733143000000
H	4.056032000000	0.352784000000	-2.718324000000
C	4.600410000000	1.853892000000	0.380122000000
H	5.139210000000	1.629995000000	1.309253000000
H	3.555689000000	2.059785000000	0.637776000000
H	5.030290000000	2.778955000000	-0.030848000000
H	-0.431346000000	2.207304000000	-2.354669000000
H	0.071646000000	3.012504000000	0.924590000000
H	-1.617710000000	0.727987000000	2.130207000000
H	1.456590000000	1.177383000000	1.993670000000
H	0.926352000000	-2.105084000000	-1.027227000000
O	0.134119000000	0.488695000000	-1.341798000000
N	-2.040447000000	2.981610000000	0.878184000000
O	-1.995532000000	4.032380000000	0.229828000000
O	-3.080328000000	2.424195000000	1.235649000000

major TS 29

H	4.942530000000	-0.828045000000	2.247063000000
C	4.270322000000	-0.184158000000	1.689510000000
C	2.513816000000	1.501615000000	0.175927000000
C	2.932693000000	-0.560246000000	1.443835000000
C	4.694913000000	1.026715000000	1.186264000000
C	3.838277000000	1.860471000000	0.425358000000
C	2.056220000000	0.272832000000	0.692605000000
H	5.710621000000	1.375647000000	1.344044000000
C	2.181209000000	-1.715280000000	1.736342000000
H	2.409627000000	-2.567456000000	2.360617000000
C	2.313304000000	-2.546274000000	-0.577358000000
H	2.160746000000	-3.560396000000	-0.235490000000
C	1.294350000000	-1.734314000000	-1.083651000000
H	1.603114000000	-0.952979000000	-1.772719000000
C	0.826419000000	-0.456670000000	0.540217000000
O	1.671436000000	2.272653000000	-0.591881000000
O	4.352641000000	3.048942000000	-0.041216000000
C	4.592124000000	3.072463000000	-1.457579000000
H	3.659591000000	2.955064000000	-2.018872000000
H	5.035752000000	4.047392000000	-1.671279000000
H	5.290998000000	2.277289000000	-1.742379000000

C	1.333038000000	3.553392000000	-0.037295000000
H	0.672472000000	4.029664000000	-0.764814000000
H	2.226167000000	4.164853000000	0.116457000000
H	0.800441000000	3.431604000000	0.914219000000
O	0.881130000000	-1.505140000000	1.460322000000
C	-0.039033000000	-2.405453000000	-1.373179000000
H	-0.230425000000	-3.172350000000	-0.610474000000
H	0.067643000000	-2.947124000000	-2.324129000000
C	-0.532015000000	0.152495000000	0.330590000000
H	-0.868072000000	0.625893000000	1.263972000000
H	-0.423599000000	0.952369000000	-0.407523000000
C	-1.623147000000	-0.809492000000	-0.152259000000
H	-1.771600000000	-1.593322000000	0.607156000000
C	-1.254976000000	-1.476507000000	-1.477614000000
H	-2.118901000000	-2.056043000000	-1.823803000000
H	-1.080017000000	-0.693280000000	-2.227761000000
O	-2.815464000000	-0.044663000000	-0.319649000000
Si	-4.305208000000	-0.352001000000	0.398913000000
C	-5.450220000000	1.012801000000	-0.292145000000
C	-4.908612000000	-2.085042000000	-0.064471000000
H	-5.021920000000	-2.202341000000	-1.148036000000
H	-5.877962000000	-2.305931000000	0.398798000000
H	-4.205561000000	-2.853917000000	0.279483000000
C	-4.142938000000	-0.258119000000	2.282158000000
H	-5.098637000000	-0.471403000000	2.776272000000
H	-3.808586000000	0.731212000000	2.614231000000
H	-3.417789000000	-0.993049000000	2.653391000000
C	-6.869388000000	0.862833000000	0.297752000000
H	-7.532001000000	1.645213000000	-0.099492000000
H	-6.874595000000	0.958748000000	1.390713000000
H	-7.321947000000	-0.103833000000	0.043731000000
C	-4.890293000000	2.402691000000	0.080236000000
H	-3.880286000000	2.548800000000	-0.318141000000
H	-4.848478000000	2.550420000000	1.166504000000
H	-5.531585000000	3.195568000000	-0.331679000000
C	-5.525321000000	0.900743000000	-1.830316000000
H	-6.163068000000	1.697309000000	-2.240563000000
H	-5.954634000000	-0.056407000000	-2.151137000000
H	-4.535129000000	0.995371000000	-2.289359000000

N	3.686809000000	-2.269703000000	-0.842540000000
O	4.517339000000	-3.126557000000	-0.500866000000
O	3.991154000000	-1.186935000000	-1.368657000000

minor TS 28

H	-3.493862000000	2.006014000000	-2.591522000000
C	-3.326957000000	1.127628000000	-1.976721000000
C	-2.903072000000	-1.167548000000	-0.315335000000
C	-2.064403000000	0.864787000000	-1.405427000000
C	-4.343986000000	0.234385000000	-1.716341000000
C	-4.146954000000	-0.894812000000	-0.884399000000
C	-1.845610000000	-0.273463000000	-0.579229000000
H	-5.337611000000	0.376956000000	-2.129454000000
C	-0.841401000000	1.565175000000	-1.394774000000
C	-1.155172000000	2.373064000000	0.889619000000
C	-0.780923000000	1.187945000000	1.530136000000
C	-0.498136000000	-0.166320000000	-0.081701000000
O	-2.698115000000	-2.240606000000	0.524571000000
O	-5.221447000000	-1.730451000000	-0.674015000000
C	-5.795030000000	-1.631217000000	0.638924000000
H	-6.652468000000	-2.307892000000	0.644983000000
H	-6.131181000000	-0.606410000000	0.835670000000
H	-5.076665000000	-1.932038000000	1.408538000000
C	-2.832372000000	-3.532334000000	-0.087907000000
H	-2.086174000000	-3.661096000000	-0.882231000000
H	-3.835299000000	-3.670780000000	-0.499949000000
H	-2.649212000000	-4.260953000000	0.704938000000
C	0.574507000000	1.161320000000	2.214816000000
H	0.475858000000	1.716059000000	3.159252000000
C	0.349671000000	-1.329977000000	0.359718000000
H	-0.324398000000	-2.016857000000	0.880859000000
C	1.540191000000	-1.022064000000	1.280643000000
H	1.938036000000	-1.994679000000	1.611050000000
C	1.127881000000	-0.233665000000	2.527325000000
H	2.007579000000	-0.131783000000	3.173705000000
H	0.385378000000	-0.819129000000	3.087944000000
O	2.544614000000	-0.296305000000	0.577098000000
Si	4.045201000000	-0.873963000000	0.085521000000

C	4.801281000000	0.563876000000	-0.919965000000
C	5.089882000000	-1.312282000000	1.601436000000
H	5.252804000000	-0.442300000000	2.247435000000
H	6.072733000000	-1.703056000000	1.311129000000
H	4.601954000000	-2.085982000000	2.207470000000
C	3.832616000000	-2.434282000000	-0.966403000000
H	3.228898000000	-2.244404000000	-1.860987000000
H	3.341445000000	-3.234027000000	-0.398012000000
H	4.803018000000	-2.825189000000	-1.296003000000
C	6.208275000000	0.175468000000	-1.423929000000
H	6.644331000000	0.998427000000	-2.008306000000
H	6.184427000000	-0.706733000000	-2.075847000000
H	6.898817000000	-0.036290000000	-0.598013000000
C	3.896069000000	0.881425000000	-2.130150000000
H	4.300762000000	1.733655000000	-2.695587000000
H	2.878302000000	1.136738000000	-1.815875000000
H	3.830516000000	0.034150000000	-2.824069000000
C	4.911417000000	1.823187000000	-0.033562000000
H	5.566480000000	1.661117000000	0.831391000000
H	3.931594000000	2.138451000000	0.341463000000
H	5.333854000000	2.659282000000	-0.609786000000
H	-0.518803000000	2.429270000000	-1.958114000000
H	-0.479641000000	3.197799000000	0.710487000000
H	-1.582320000000	0.643414000000	2.022559000000
H	1.306444000000	1.706252000000	1.608920000000
H	0.721487000000	-1.859932000000	-0.528227000000
O	0.128457000000	0.796689000000	-0.869821000000
N	-2.522697000000	2.769507000000	0.800913000000
O	-2.758803000000	3.911544000000	0.375478000000
O	-3.406913000000	1.957769000000	1.119172000000

major DA adduct

H	5.087758000000	-0.585205000000	1.957713000000
C	4.345391000000	0.025040000000	1.452478000000
C	2.419116000000	1.606336000000	0.080151000000
C	3.083308000000	-0.461965000000	1.156547000000
C	4.643320000000	1.336603000000	1.061883000000
C	3.707879000000	2.113422000000	0.377164000000

C	2.123213000000	0.310792000000	0.488035000000
H	5.611396000000	1.779740000000	1.274385000000
C	2.416544000000	-1.809165000000	1.332315000000
H	2.762798000000	-2.456187000000	2.135900000000
C	2.347485000000	-2.487033000000	-0.082611000000
H	2.080420000000	-3.541635000000	0.015490000000
C	1.255836000000	-1.660094000000	-0.801568000000
H	1.657615000000	-1.175337000000	-1.693849000000
C	0.910140000000	-0.604796000000	0.316404000000
O	1.508990000000	2.352783000000	-0.635544000000
O	4.047754000000	3.402782000000	0.028355000000
C	4.291938000000	3.595928000000	-1.371062000000
H	3.402494000000	3.363492000000	-1.965757000000
H	4.552194000000	4.650075000000	-1.490246000000
H	5.130485000000	2.972523000000	-1.706297000000
C	0.977704000000	3.492988000000	0.056921000000
H	0.301028000000	3.982668000000	-0.646577000000
H	1.774889000000	4.181268000000	0.350978000000
H	0.414537000000	3.176268000000	0.943504000000
O	1.039862000000	-1.451268000000	1.503257000000
C	0.040064000000	-2.533591000000	-1.168628000000
H	-0.148030000000	-3.246993000000	-0.355068000000
H	0.279787000000	-3.128748000000	-2.058528000000
C	-0.459916000000	0.044938000000	0.232548000000
H	-0.676143000000	0.541131000000	1.185666000000
H	-0.421584000000	0.822062000000	-0.537208000000
C	-1.591127000000	-0.922373000000	-0.131918000000
H	-1.736816000000	-1.634262000000	0.695335000000
C	-1.233294000000	-1.710584000000	-1.394481000000
H	-2.067361000000	-2.368755000000	-1.664589000000
H	-1.101983000000	-1.002514000000	-2.224229000000
O	-2.776111000000	-0.160435000000	-0.351114000000
Si	-4.238866000000	-0.346909000000	0.456977000000
C	-5.387957000000	0.955672000000	-0.341026000000
C	-4.894299000000	-2.106717000000	0.219334000000
H	-5.047630000000	-2.345050000000	-0.839200000000
H	-5.850859000000	-2.249702000000	0.736648000000
H	-4.194550000000	-2.846952000000	0.626920000000
C	-3.998226000000	-0.040486000000	2.308652000000

H	-4.936225000000	-0.173347000000	2.861459000000
H	-3.632259000000	0.972717000000	2.509422000000
H	-3.270848000000	-0.743217000000	2.733730000000
C	-6.784010000000	0.903632000000	0.316647000000
H	-7.448695000000	1.651729000000	-0.139046000000
H	-6.742111000000	1.119760000000	1.391500000000
H	-7.263566000000	-0.075030000000	0.189927000000
C	-4.789926000000	2.366358000000	-0.150835000000
H	-3.794642000000	2.444711000000	-0.601932000000
H	-4.701213000000	2.634508000000	0.909235000000
H	-5.433895000000	3.121466000000	-0.625039000000
C	-5.527992000000	0.673140000000	-1.852475000000
H	-6.170788000000	1.430423000000	-2.324774000000
H	-5.983566000000	-0.305839000000	-2.046025000000
H	-4.556279000000	0.697205000000	-2.357799000000
N	3.707256000000	-2.521358000000	-0.738768000000
O	4.591470000000	-3.061201000000	-0.074781000000
O	3.856265000000	-2.043286000000	-1.857020000000

minor DA adduct

H	-3.604198000000	1.833808000000	-2.528131000000
C	-3.397707000000	0.979570000000	-1.890646000000
C	-2.876001000000	-1.229185000000	-0.177667000000
C	-2.195641000000	0.851773000000	-1.215354000000
C	-4.354308000000	-0.027550000000	-1.711844000000
C	-4.109386000000	-1.107379000000	-0.863159000000
C	-1.922540000000	-0.236279000000	-0.373441000000
H	-5.311853000000	0.009460000000	-2.222193000000
C	-0.973278000000	1.735185000000	-1.088287000000
C	-1.010811000000	2.393628000000	0.334187000000
C	-0.700780000000	1.202316000000	1.270341000000
C	-0.534615000000	0.043423000000	0.213817000000
O	-2.651941000000	-2.274255000000	0.694498000000
O	-5.078054000000	-2.079955000000	-0.732686000000
C	-5.779218000000	-2.055627000000	0.517167000000
H	-6.509923000000	-2.866295000000	0.470667000000
H	-6.302144000000	-1.099840000000	0.648481000000
H	-5.097666000000	-2.218219000000	1.358685000000

C	-2.523367000000	-3.562830000000	0.074746000000
H	-1.648897000000	-3.585882000000	-0.587705000000
H	-3.422909000000	-3.818267000000	-0.491808000000
H	-2.380896000000	-4.275849000000	0.889895000000
C	0.579519000000	1.433928000000	2.092018000000
H	0.362195000000	2.137081000000	2.905674000000
C	0.295335000000	-1.155341000000	0.648422000000
H	-0.363767000000	-1.803452000000	1.235647000000
C	1.529595000000	-0.827475000000	1.504304000000
H	1.894893000000	-1.771589000000	1.937634000000
C	1.153865000000	0.126365000000	2.646530000000
H	2.044167000000	0.331128000000	3.251610000000
H	0.422035000000	-0.369606000000	3.299966000000
O	2.553819000000	-0.218541000000	0.724199000000
Si	3.922471000000	-0.951604000000	0.082918000000
C	4.694181000000	0.390445000000	-1.036276000000
C	5.082574000000	-1.464732000000	1.488360000000
H	5.402662000000	-0.603296000000	2.085261000000
H	5.982747000000	-1.960643000000	1.105247000000
H	4.589849000000	-2.171196000000	2.168223000000
C	3.454440000000	-2.502551000000	-0.897601000000
H	2.774978000000	-2.272189000000	-1.725673000000
H	2.961186000000	-3.245289000000	-0.258085000000
H	4.344702000000	-2.984365000000	-1.320218000000
C	6.015088000000	-0.121480000000	-1.650467000000
H	6.454419000000	0.646364000000	-2.303560000000
H	5.864882000000	-1.019052000000	-2.263564000000
H	6.762354000000	-0.360379000000	-0.883354000000
C	3.706954000000	0.744258000000	-2.170408000000
H	4.113232000000	1.559078000000	-2.787851000000
H	2.735617000000	1.065005000000	-1.778835000000
H	3.531430000000	-0.109524000000	-2.836641000000
C	4.982023000000	1.660561000000	-0.207151000000
H	5.697135000000	1.469744000000	0.602866000000
H	4.067446000000	2.063917000000	0.240739000000
H	5.415553000000	2.443209000000	-0.846910000000
H	-0.742067000000	2.416013000000	-1.904942000000
H	-0.260233000000	3.184555000000	0.402296000000
H	-1.544069000000	1.006264000000	1.936668000000

H	1.343243000000	1.895480000000	1.457040000000
H	0.591730000000	-1.717925000000	-0.243607000000
O	0.075785000000	0.781617000000	-0.888953000000
N	-2.316417000000	3.112954000000	0.575055000000
O	-2.632033000000	3.927647000000	-0.291506000000
O	-2.962955000000	2.875661000000	1.588786000000

5. Additional References

74 J. Weinstock, D. L. Ladd, J. W. Wilson, C. K. Brush, N. C. F. Yim, G. Gallagher, Jr., M. E. McCarthy, J. Silvestri, H. M. Sarau, K. E. Flaim, D. M. Ackerman, P. E. Setler, A. J. Tobia and R. A. Hahn, *J. Med. Chem*., 1986, **29**, 2315.

75 Q. Zi, M. Li, J. Cong, G. Deng, S. Duan, M. Yin, W. Chen, H. Jing, X. Yang and P. J. Walsh, *Org. Lett*., 2022, **24**, 1786.

76 J. D. Vasta, J. J. Higgin, E. A. Kersteen, R. T. Raines, *Bioorg. Med. Chem*., 2013, **21**, 3597.

77 D. A. Fort, T. J. Woltering, A. M. Alker and T. Bach, *Heterocycles*, 2014, **88**, 1079.

78 S. Specklin, J. Cossy, *J. Org. Chem*., 2015, **80**, 3302.

79 S. Attaluri, C. R. Iden, R. R. Bonala, F. Johnson, *Chem. Res. Toxicol*., 2014, **27**, 1236.

80 P. Callant, L. D'Haenens, M. Vandewalle, *Synth. Commun*., 1984, **14**, 155.

6. Copies of ^{1}H and ^{13}C NMR Spectra

^{1}H NMR spectrum of Weinreb amide **S5** (500 MHz, $CDCl_3$)

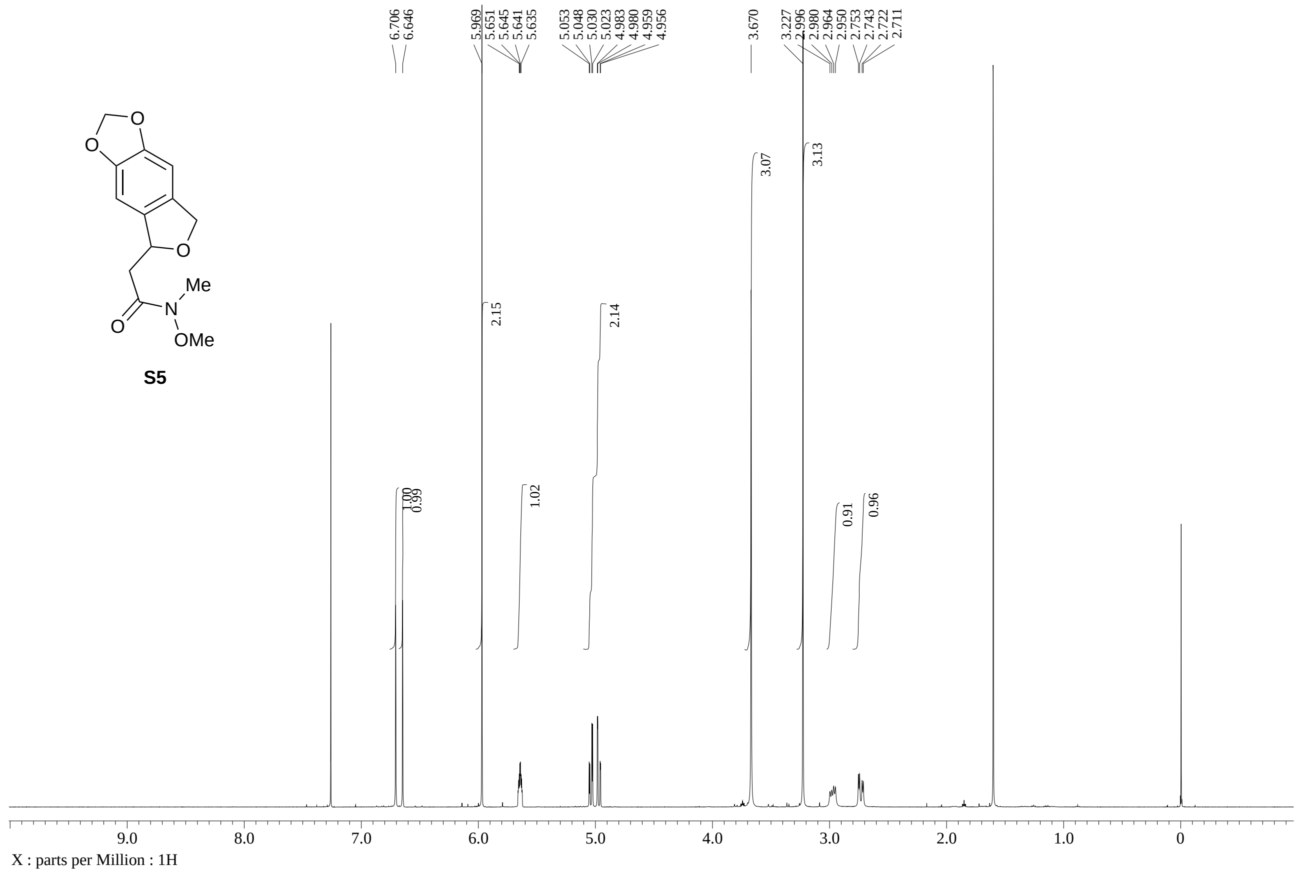

^{13}C NMR spectrum of Weinreb amide **S5** (126 MHz, $CDCl_3$)

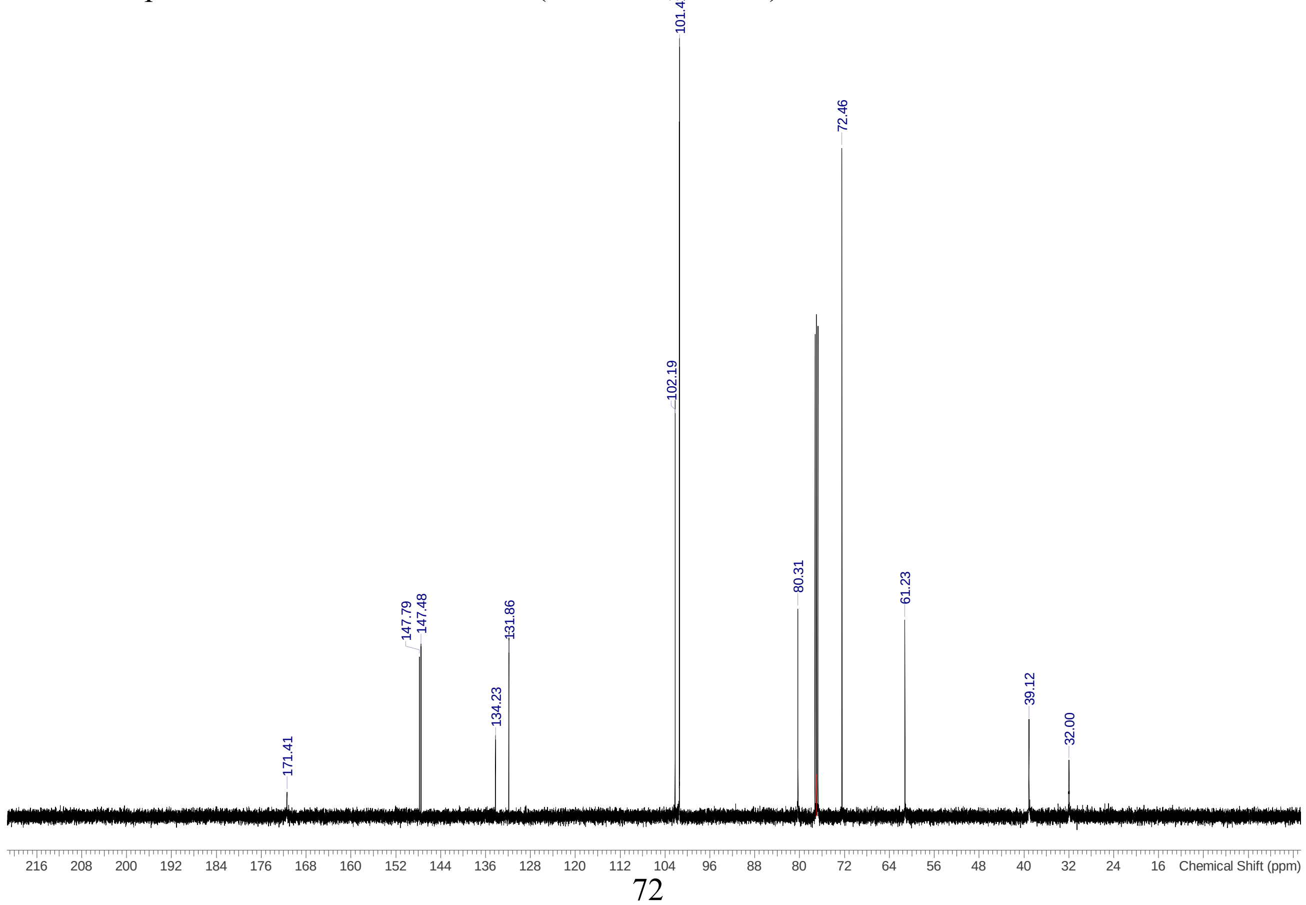

^{1}H NMR spectrum of Weinreb amide **S8** (500 MHz, $CDCl_3$)

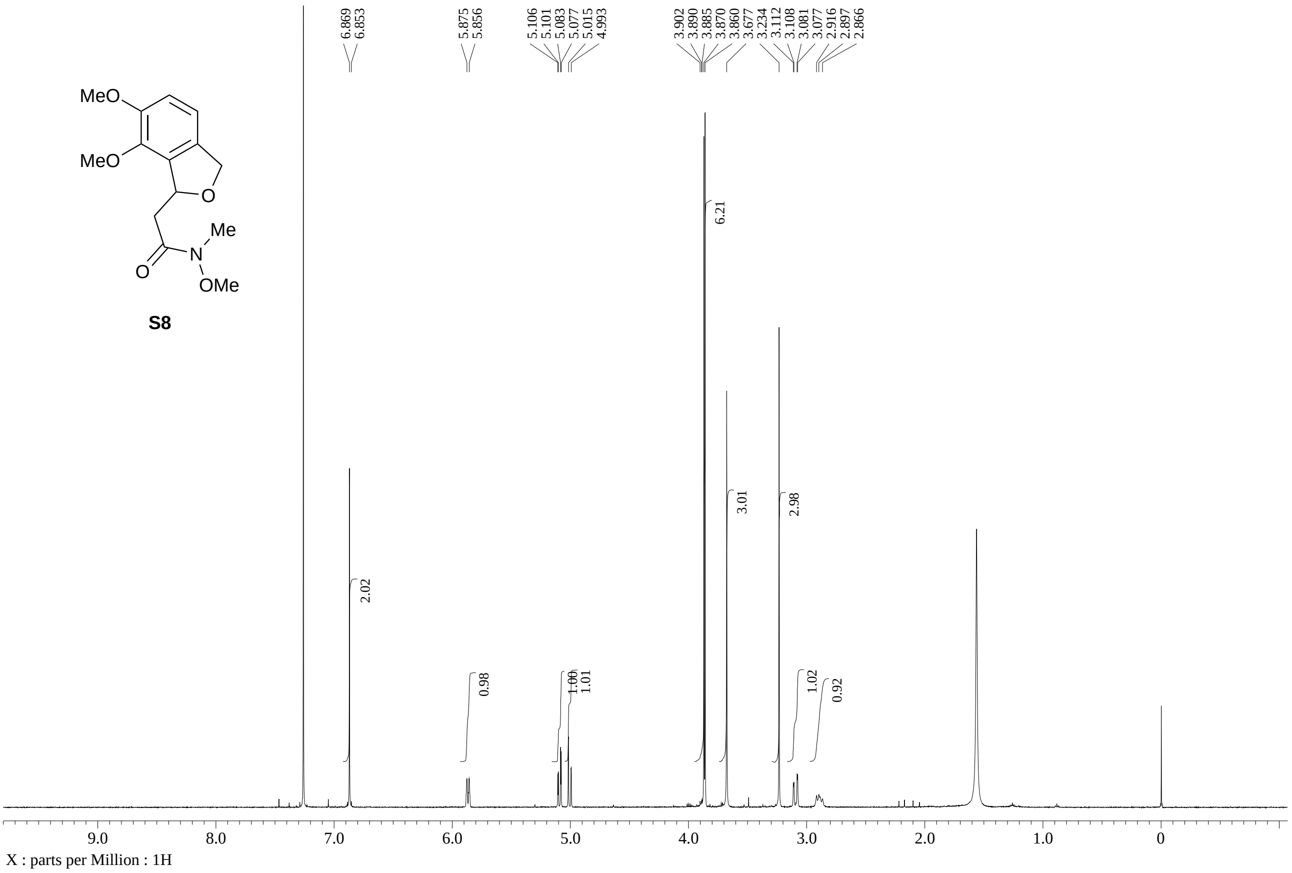

^{13}C NMR spectrum of Weinreb amide **S8** (126 MHz, $CDCl_3$)

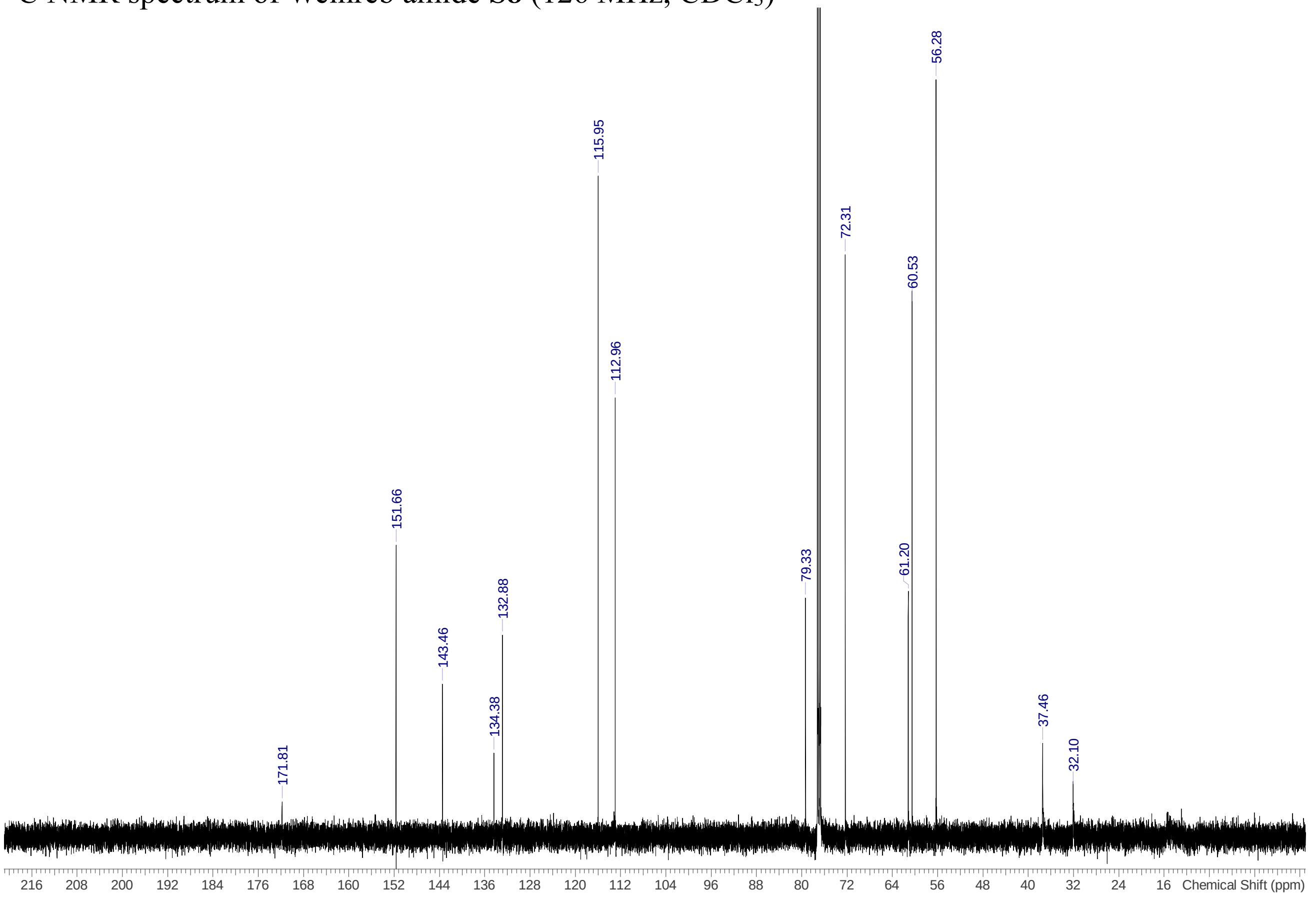

^{1}H NMR spectrum of Weinreb amide **S12** (500 MHz, $CDCl_3$)

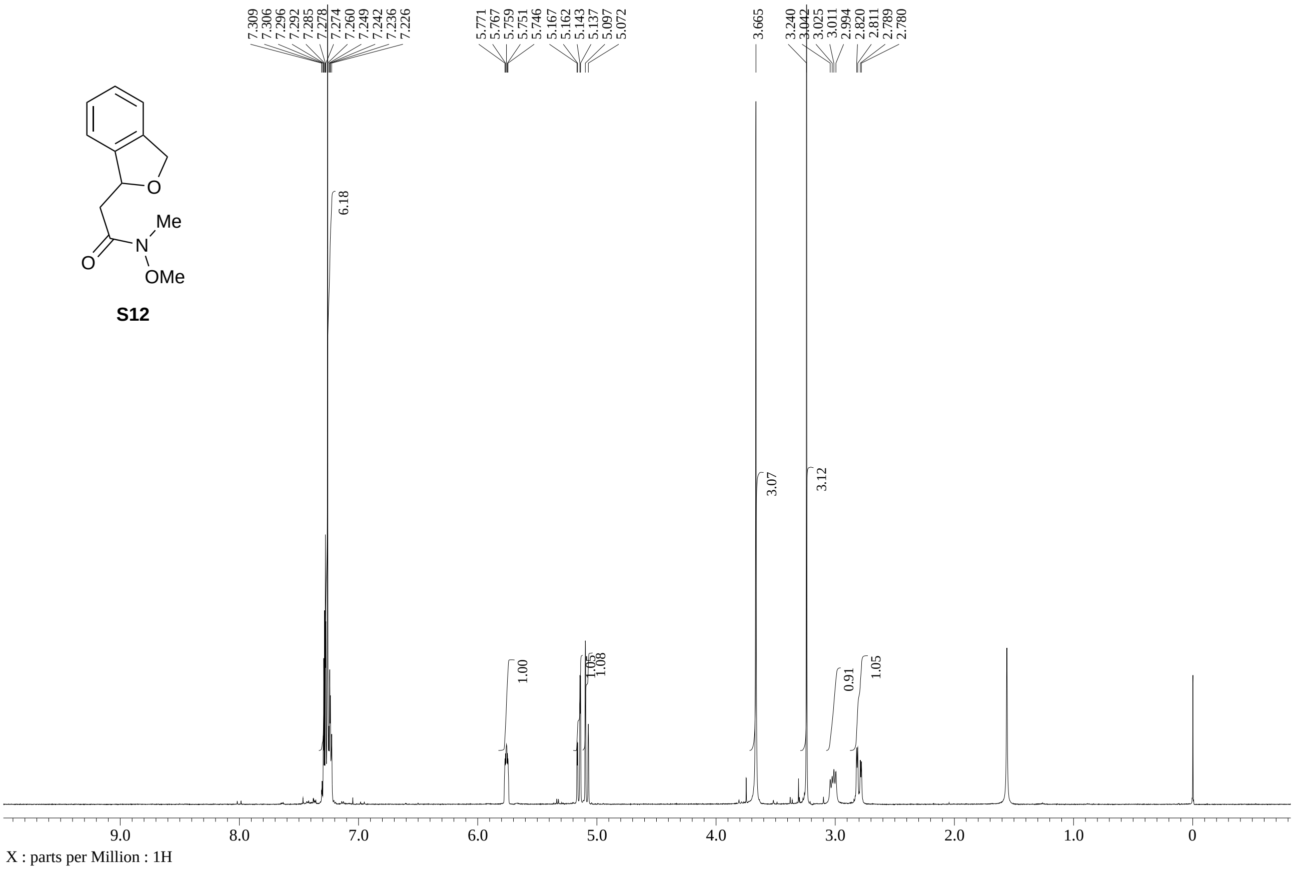

^{13}C NMR spectrum of Weinreb amide **S12** (126 MHz, $CDCl_3$)

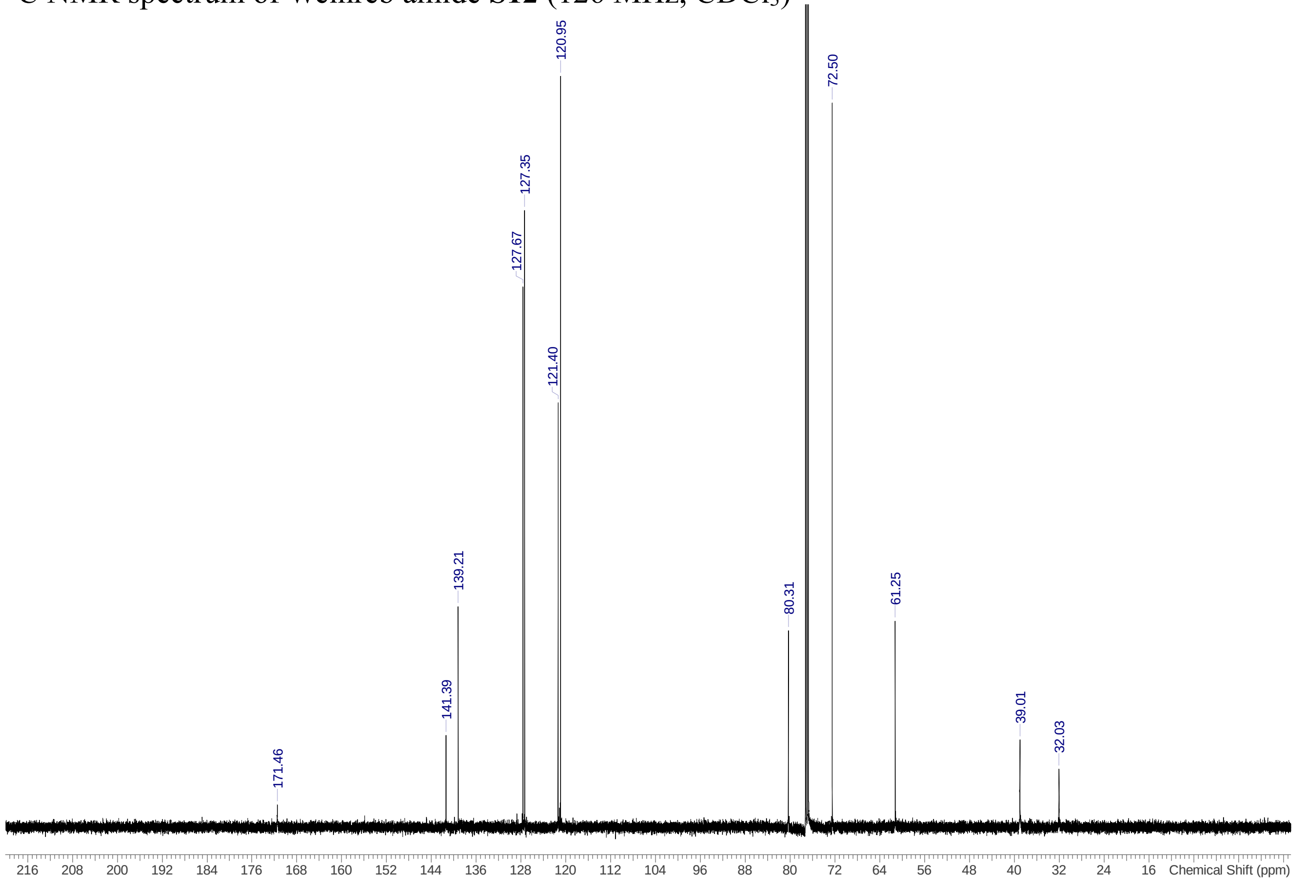

^{1}H NMR spectrum of ketone **S6** (500 MHz, $CDCl_3$)

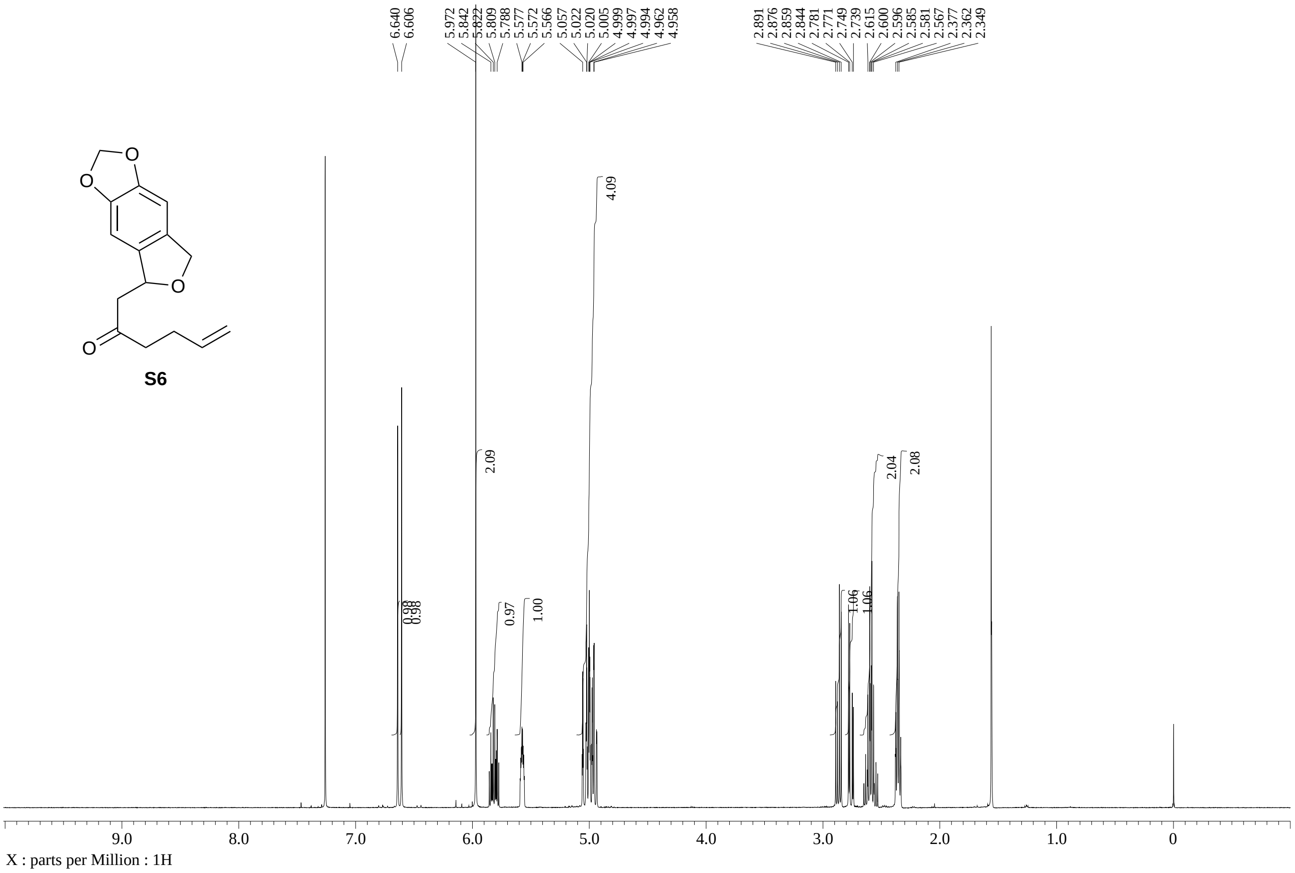

^{13}C NMR spectrum of ketone **S6** (126 MHz, $CDCl_3$)

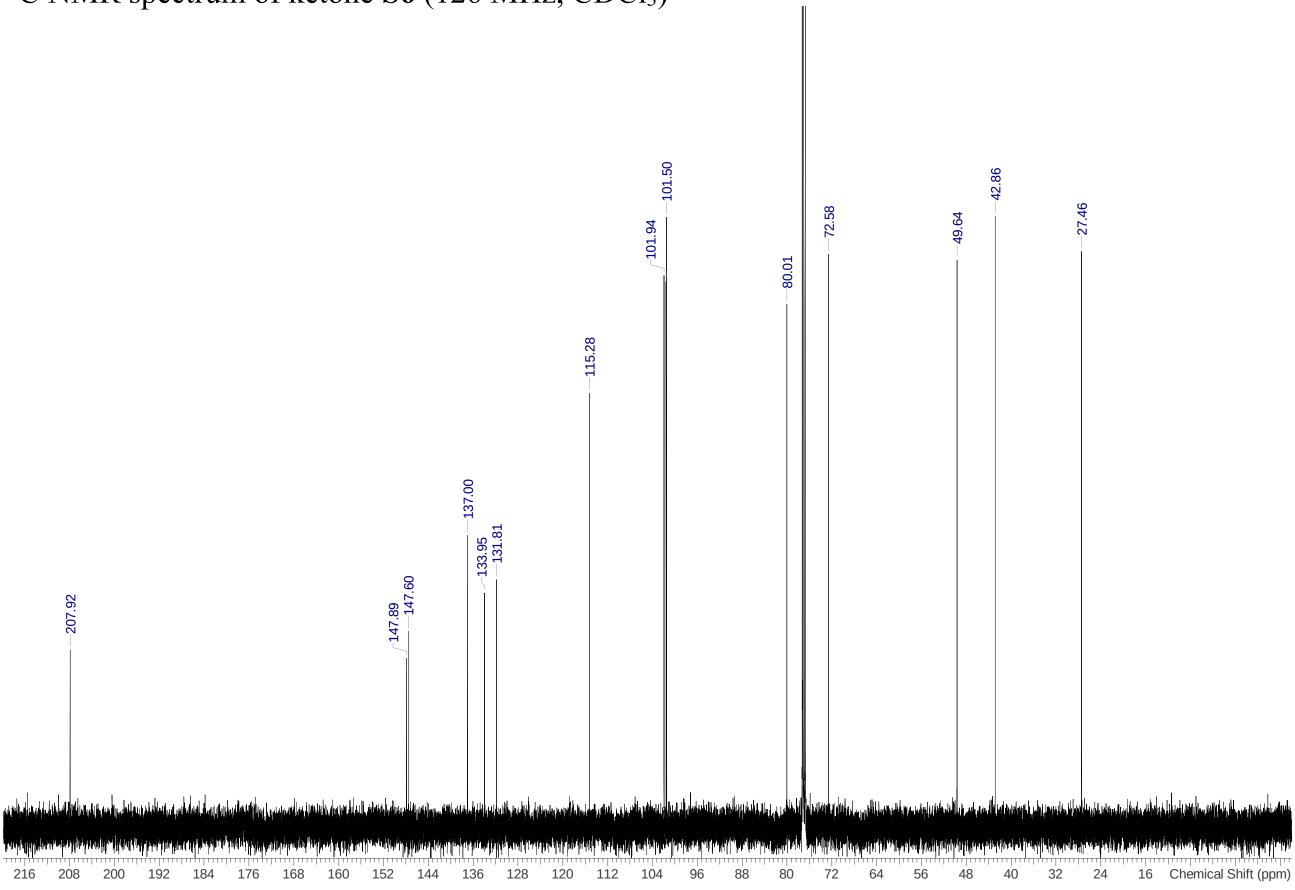

^{1}H NMR spectrum of ketone **S9** (500 MHz, $CDCl_3$)

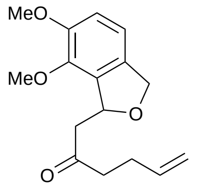

S9

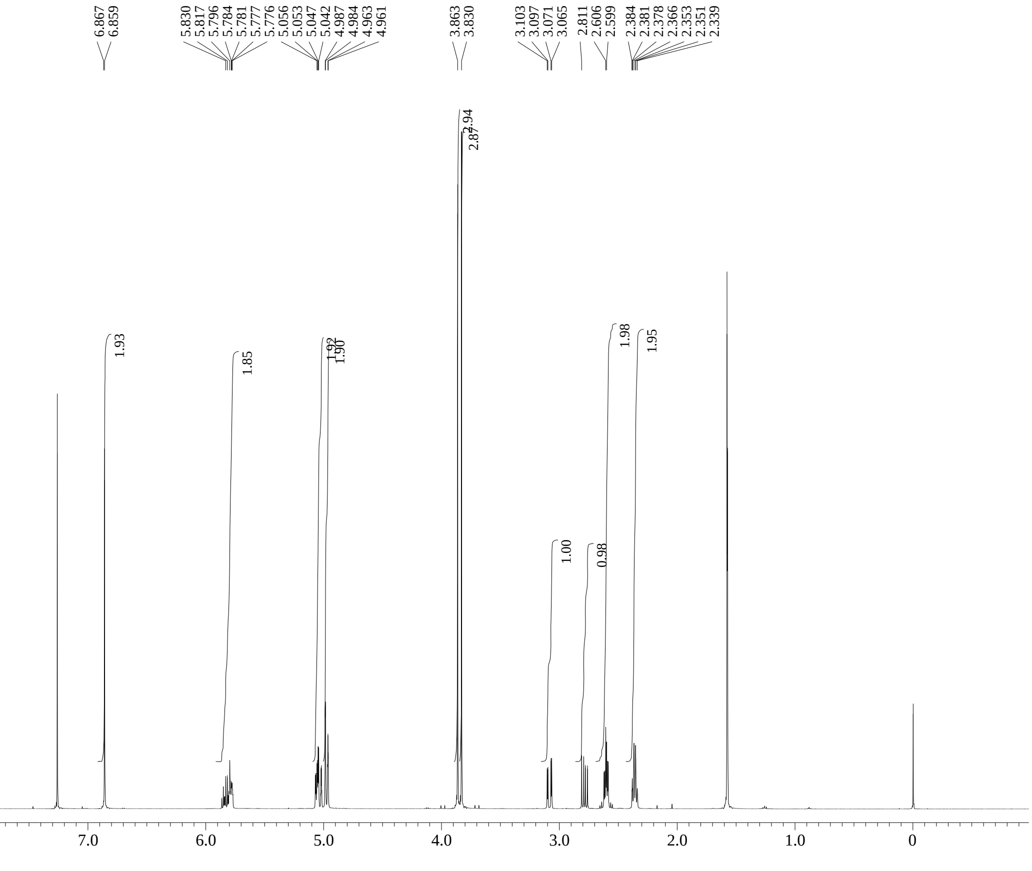

^{13}C NMR spectrum of ketone **S9** (126 MHz, $CDCl_3$)

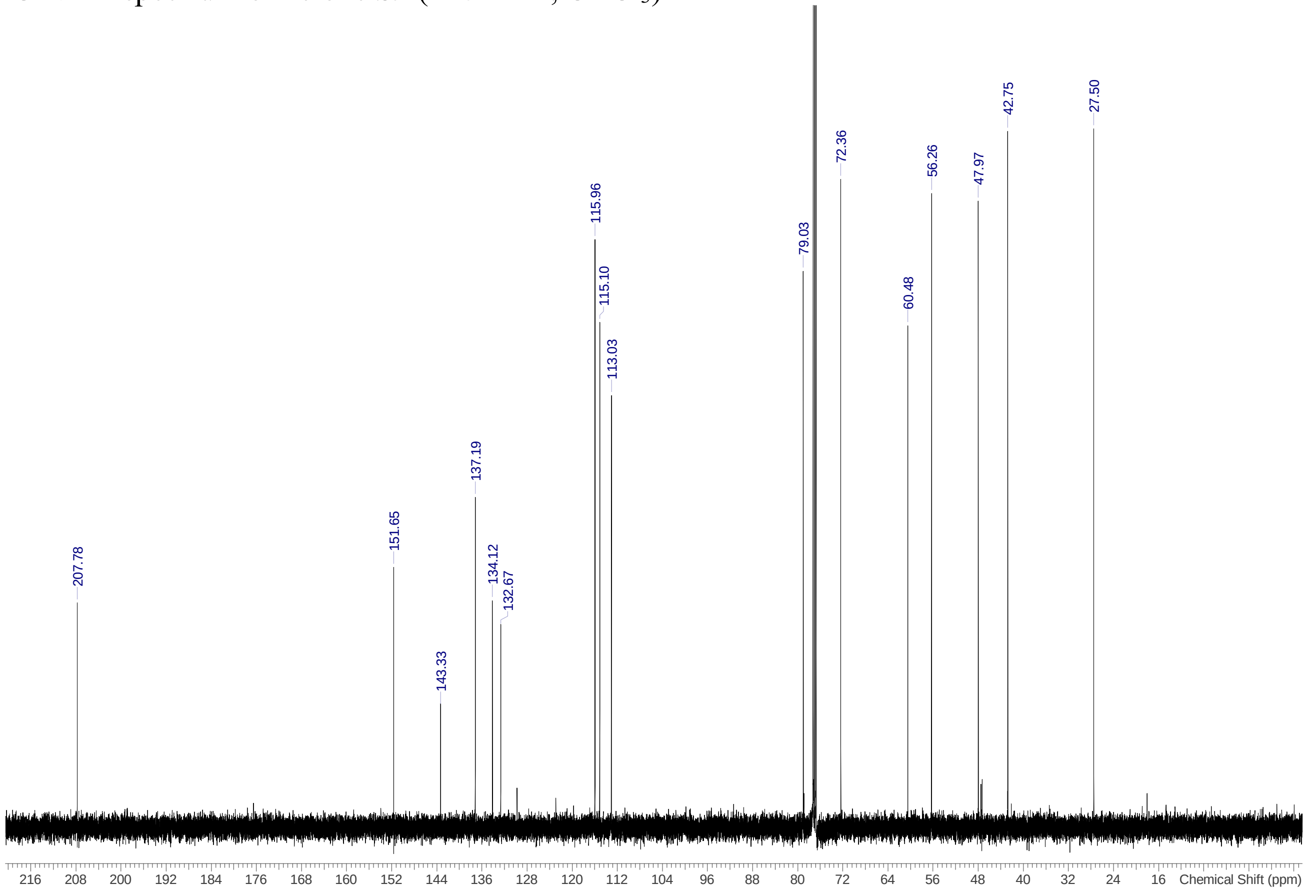

^{1}H NMR spectrum of ketone **S13** (500 MHz, $CDCl_3$)

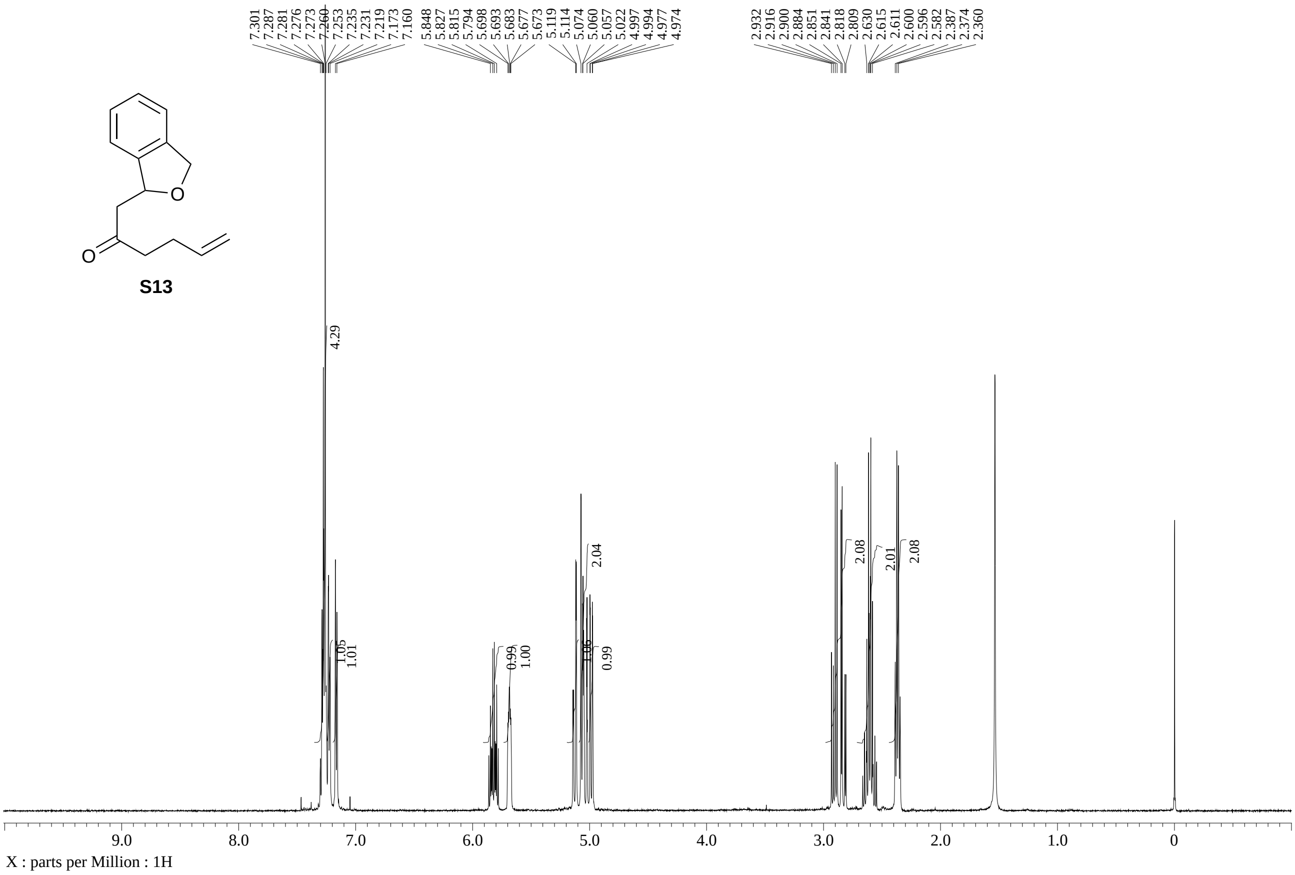

^{13}C NMR spectrum of ketone **S13** (126 MHz, $CDCl_3$)

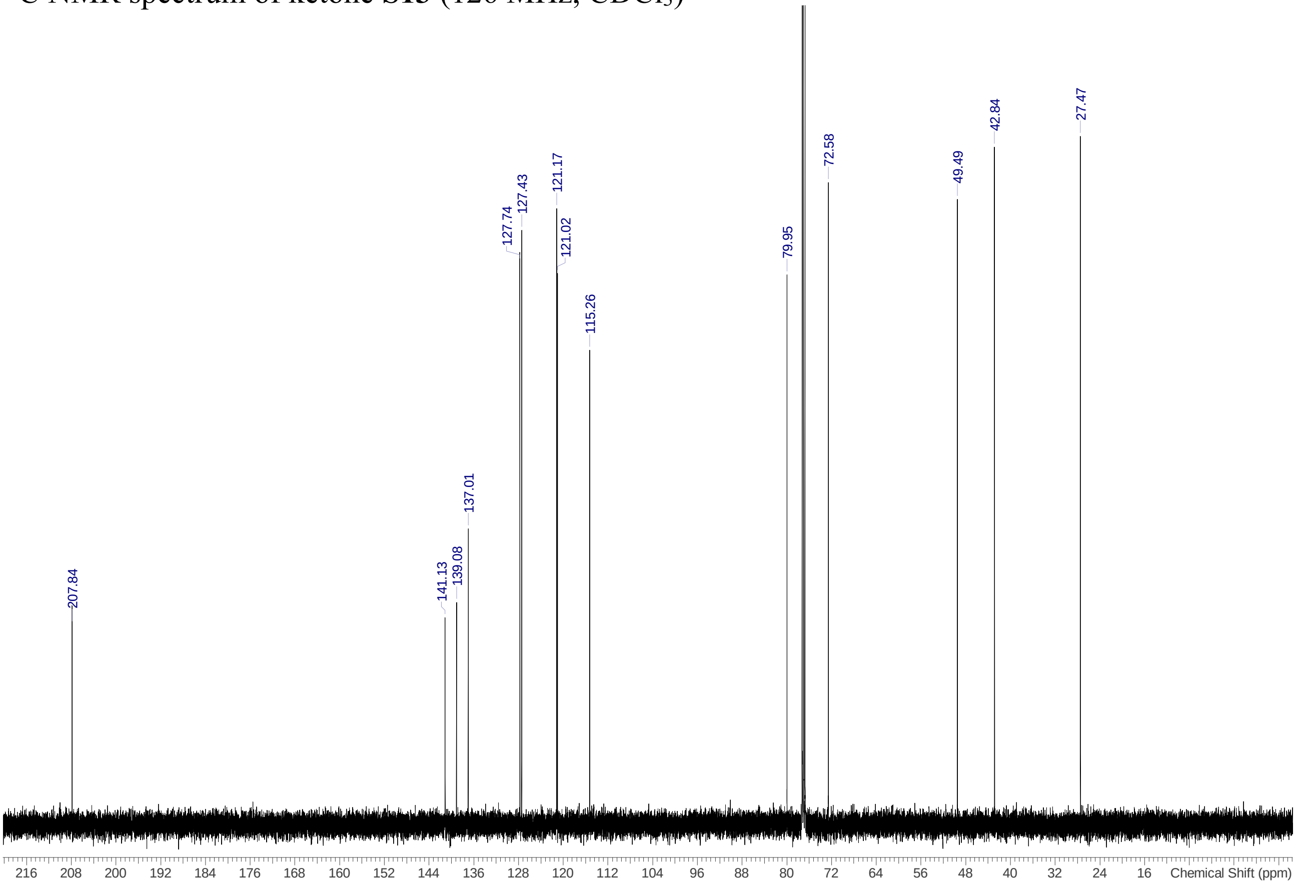

^{1}H NMR spectrum of phthalan **8a** (500 MHz, $CDCl_3$)

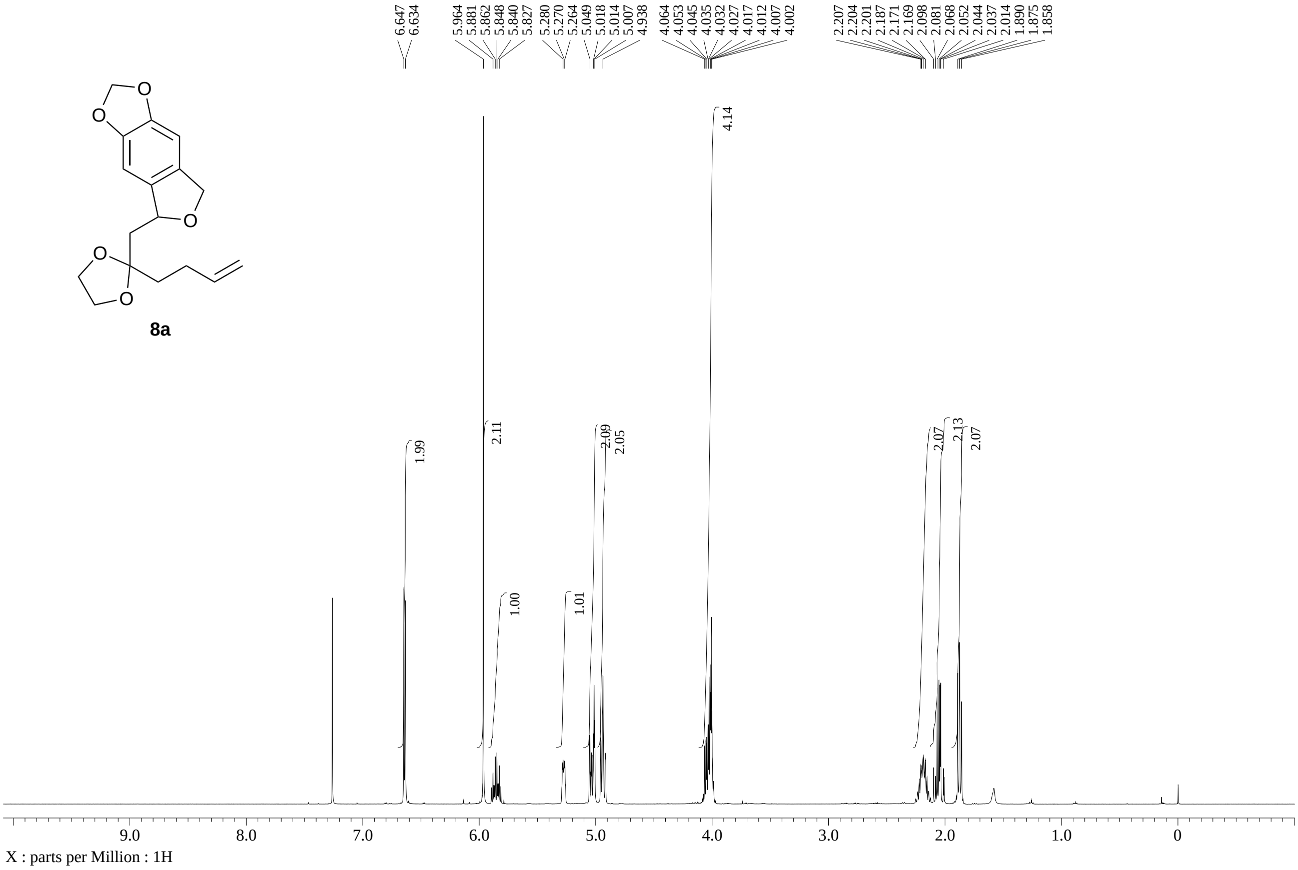

^{13}C NMR spectrum of phthalan **8a** (126 MHz, $CDCl_3$)

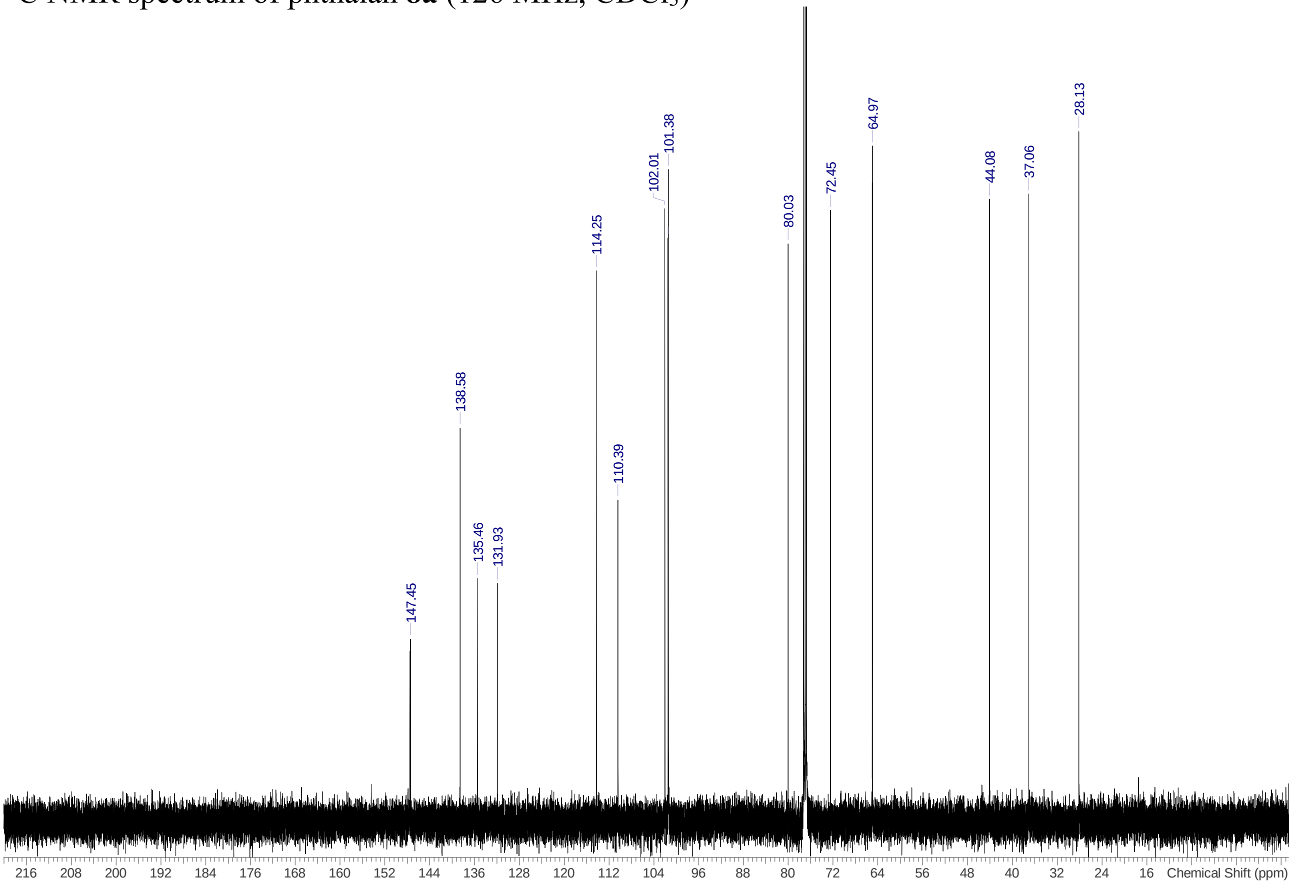

^{1}H NMR spectrum of acetal **S10** (500 MHz, $CDCl_3$)

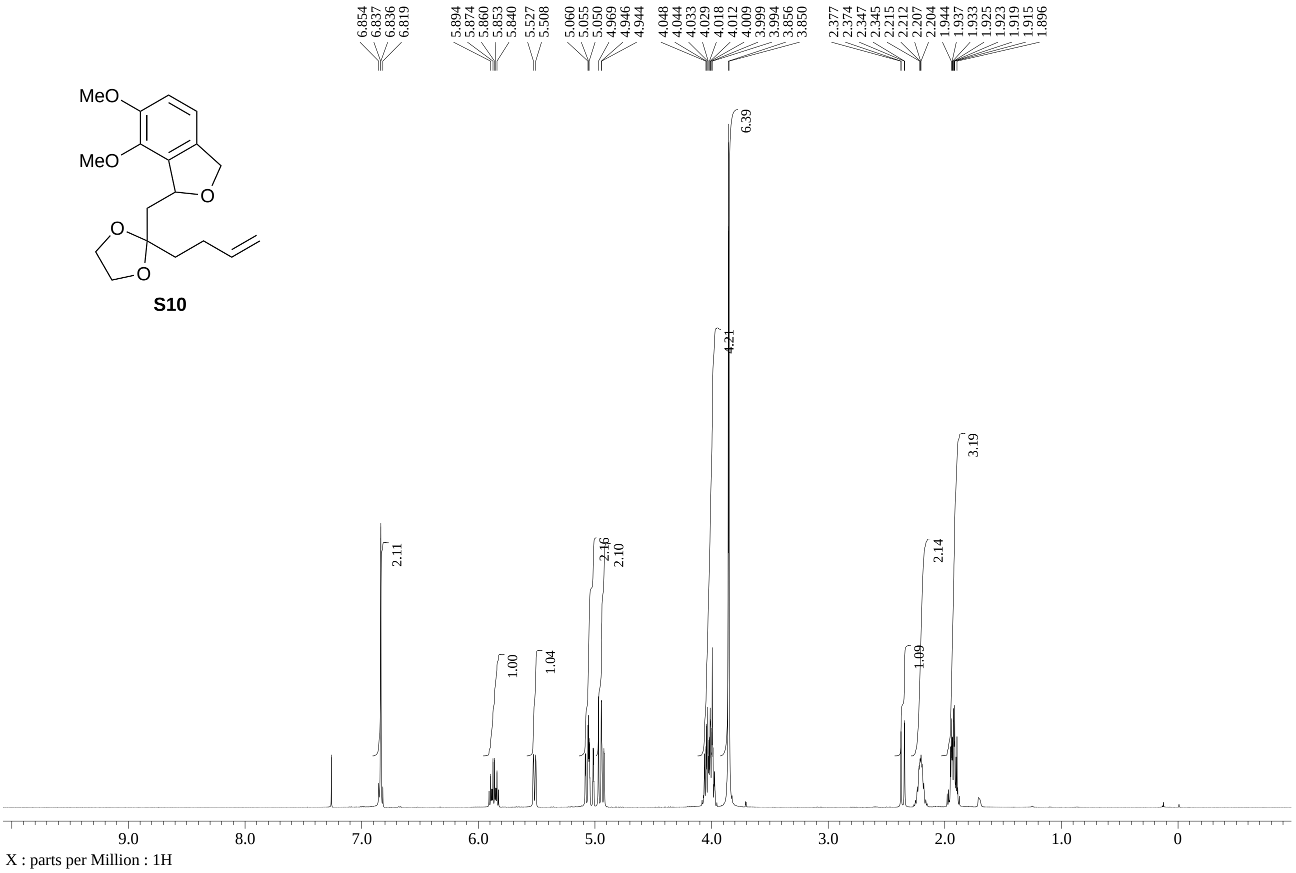

^{13}C NMR spectrum of acetal **S10** (126 MHz, $CDCl_3$)

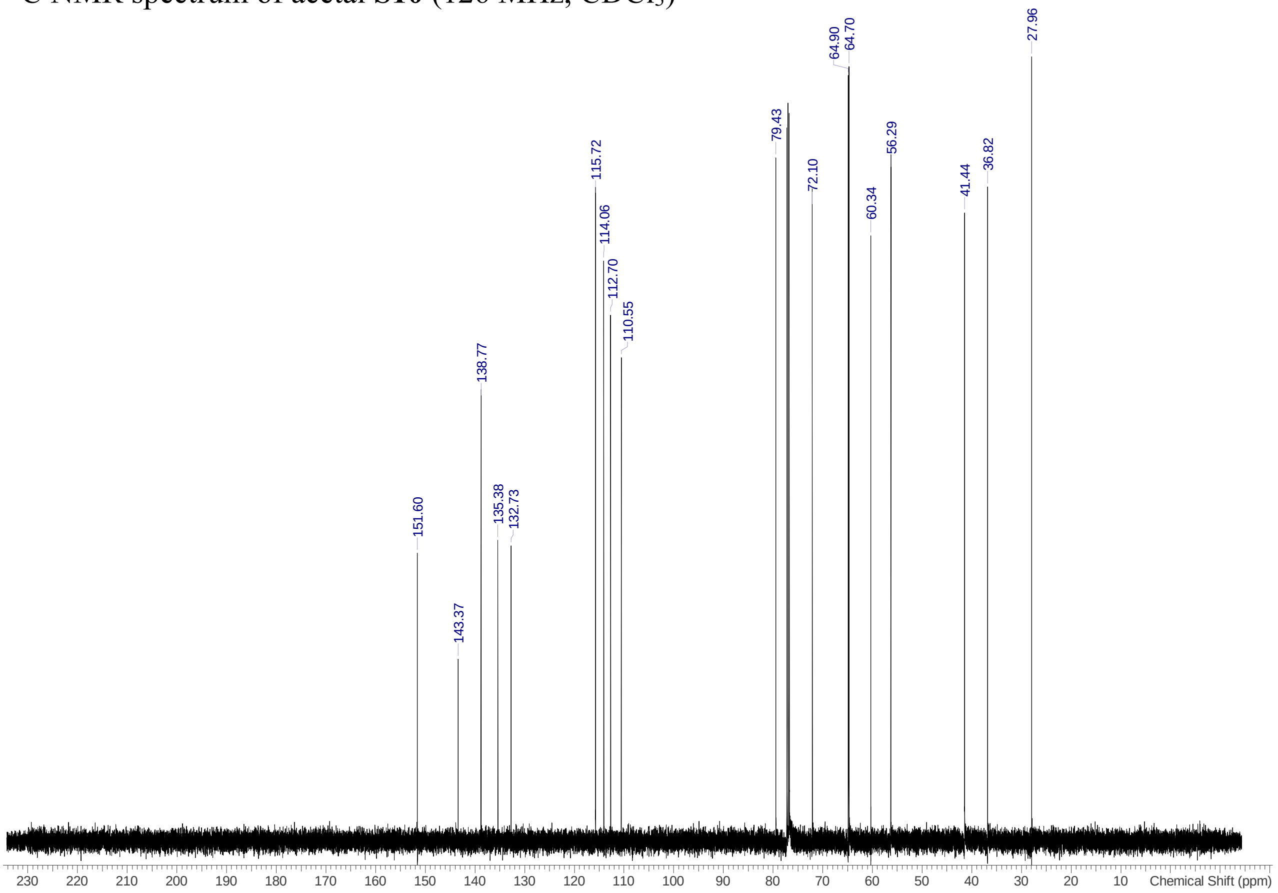

^{1}H NMR spectrum of acetal **S14** (500 MHz, $CDCl_3$)

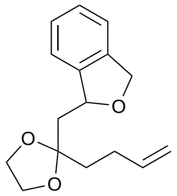

S14

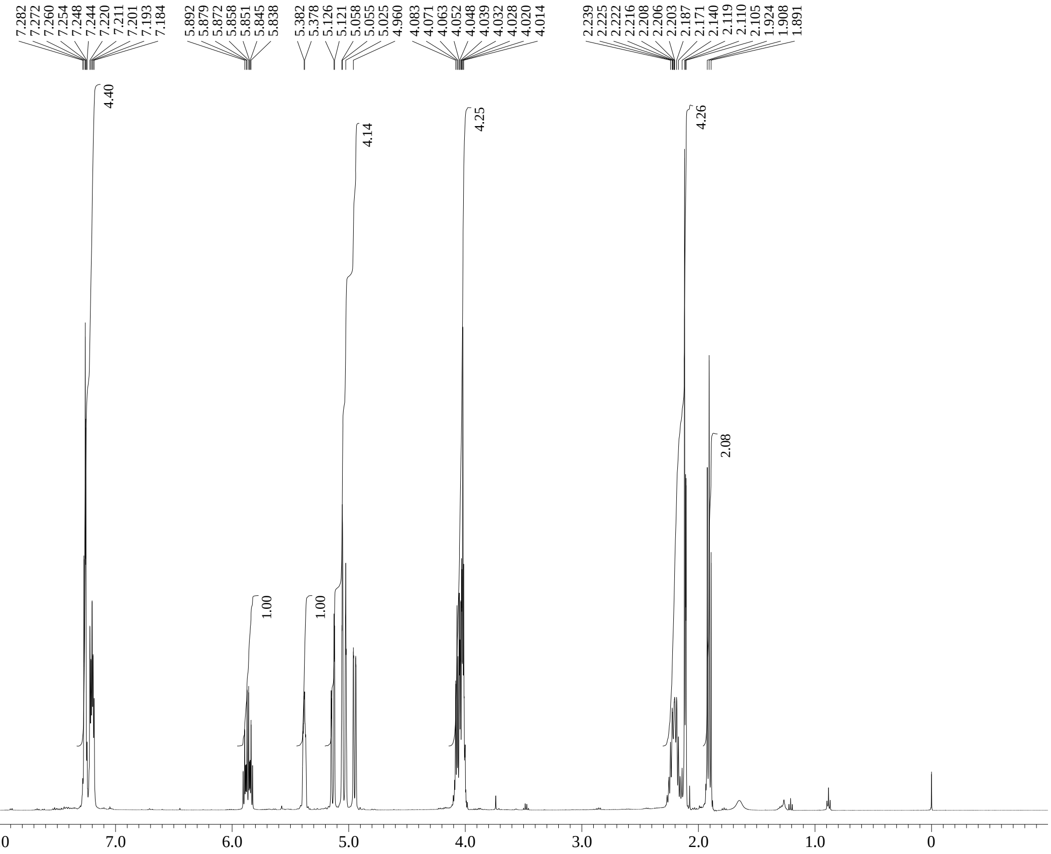

^{13}C NMR spectrum of acetal **S14** (126 MHz, $CDCl_3$)

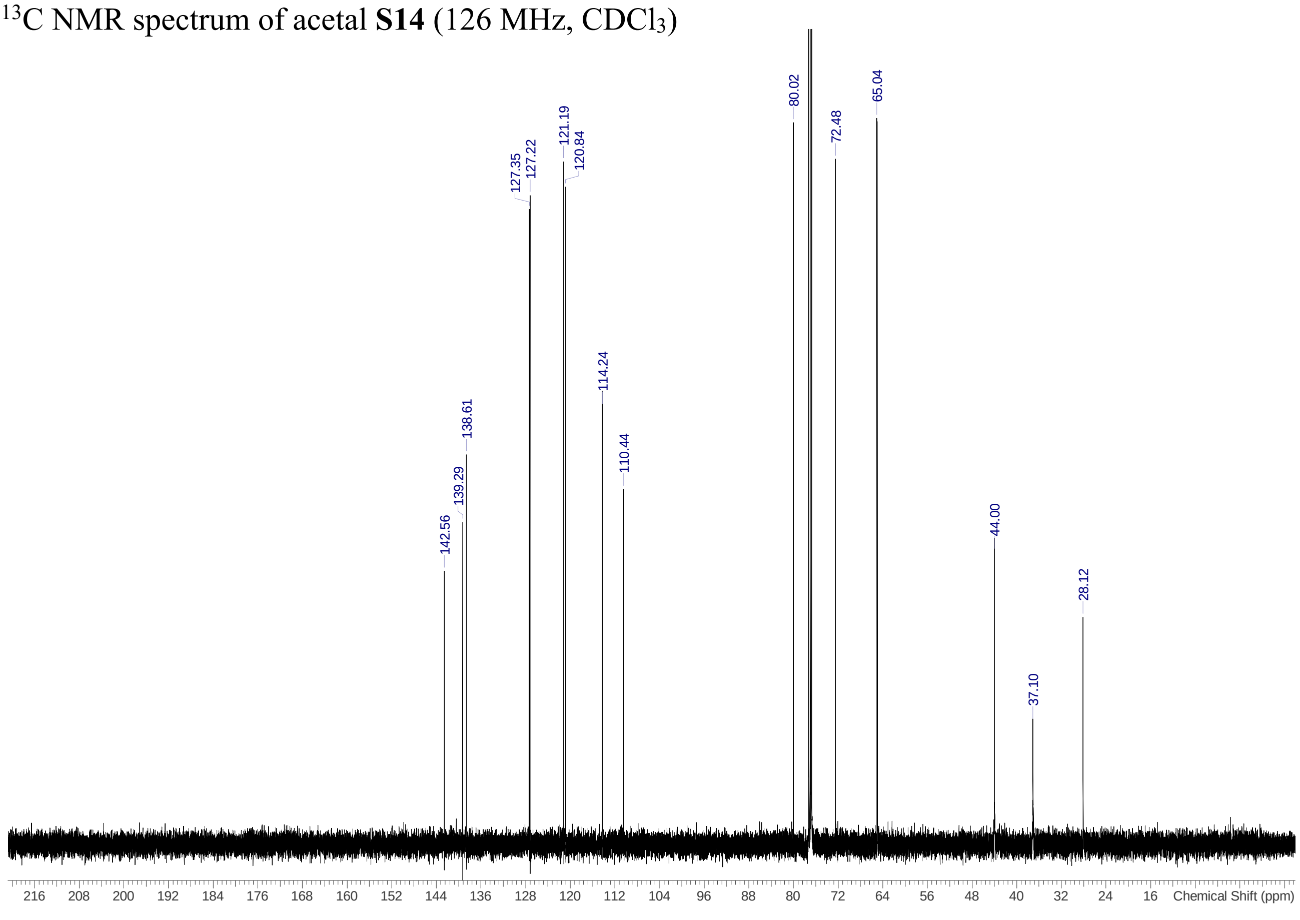

^{1}H NMR spectrum of phthalan **1** (500 MHz, $CDCl_3$)

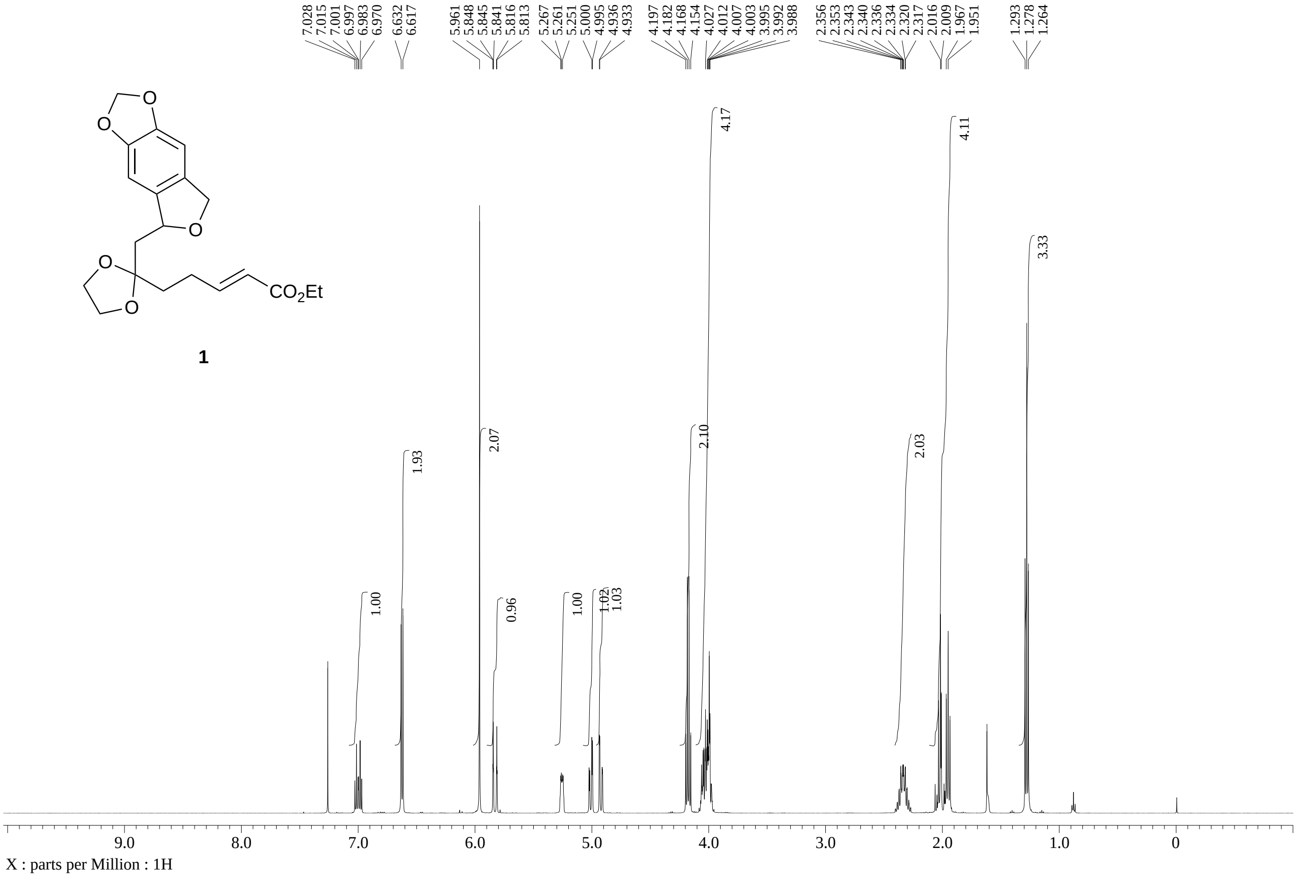

^{13}C NMR spectrum of phthalan **1** (126 MHz, $CDCl_3$)

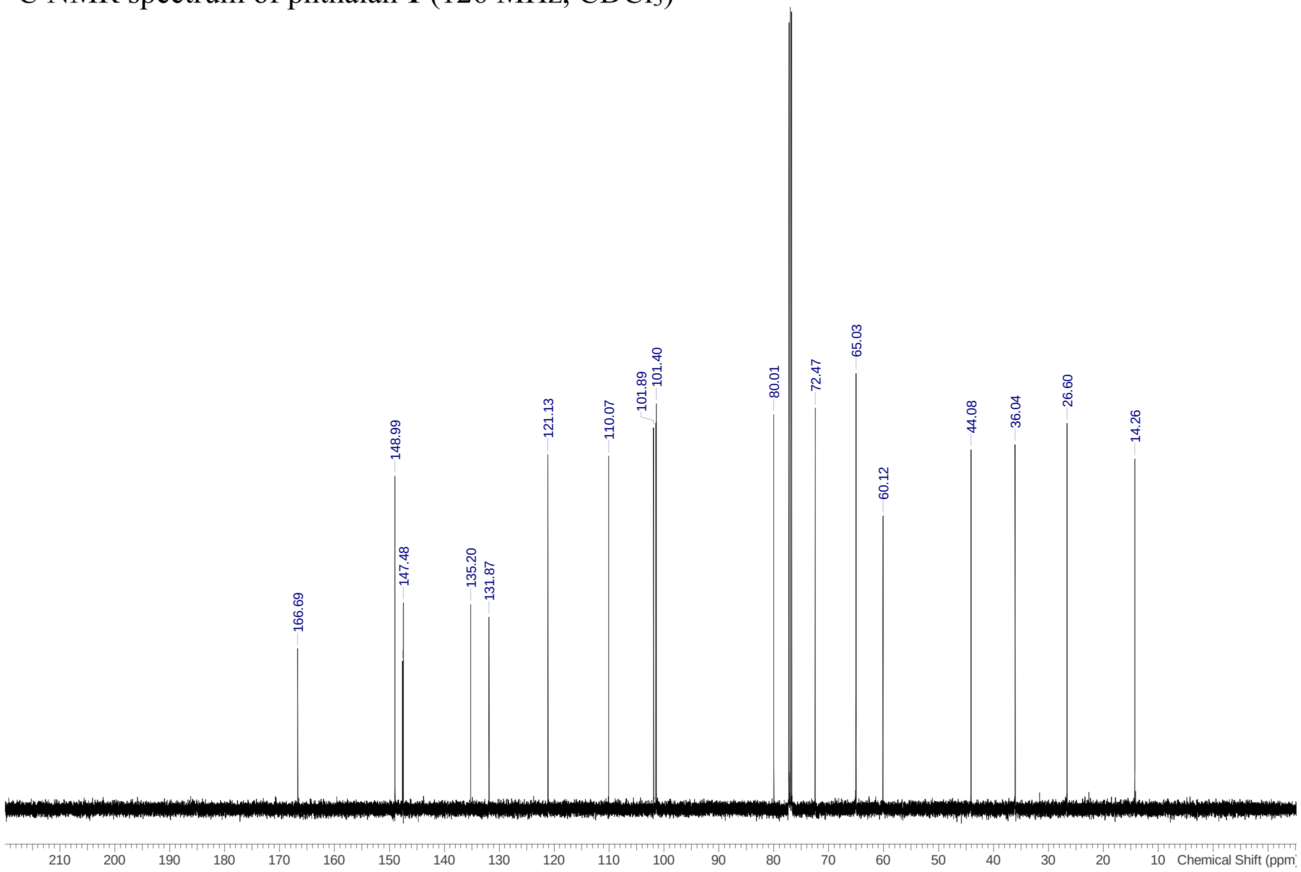

^{1}H NMR spectrum of phthalan **14a** (500 MHz, $CDCl_3$)

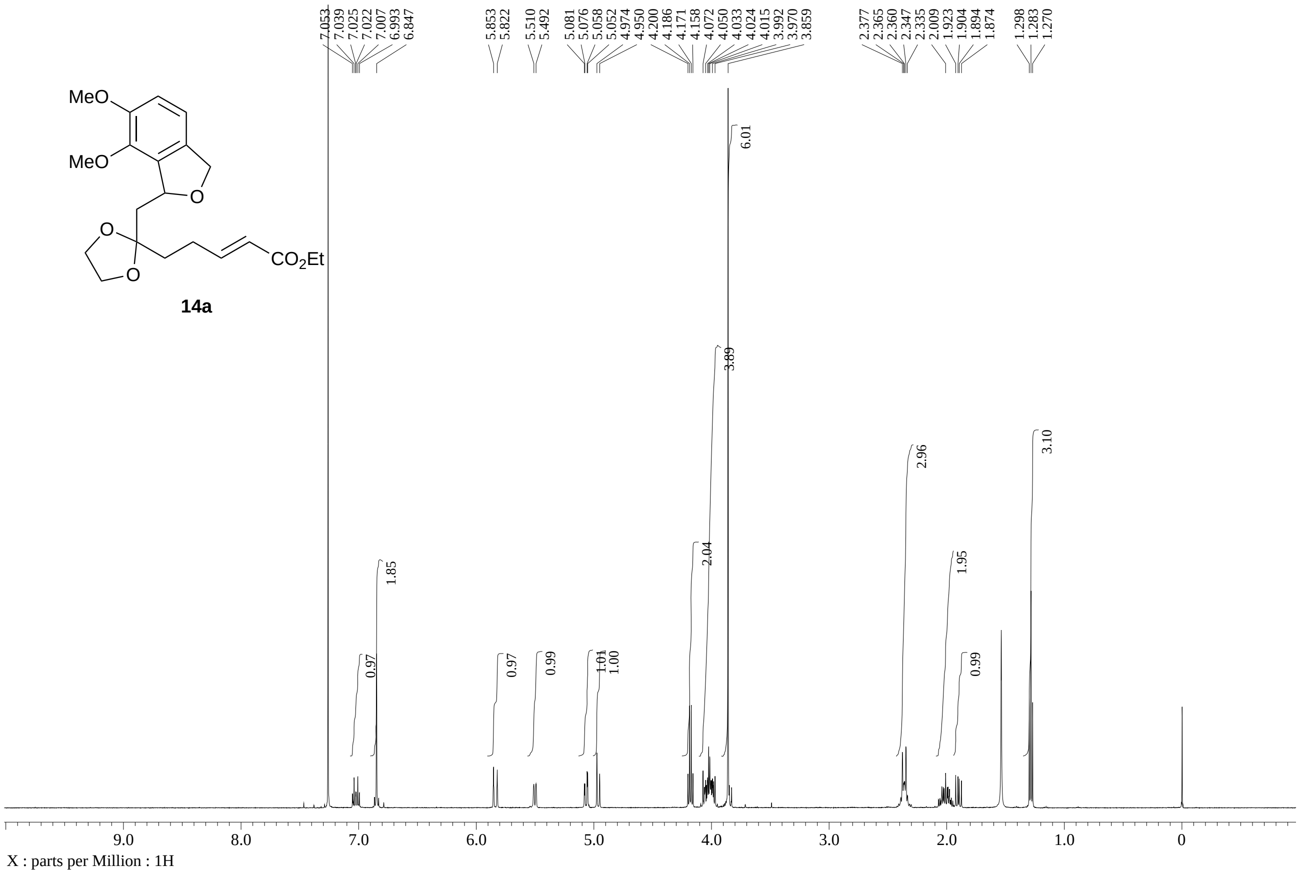

^{13}C NMR spectrum of phthalan **14a** (126 MHz, $CDCl_3$)

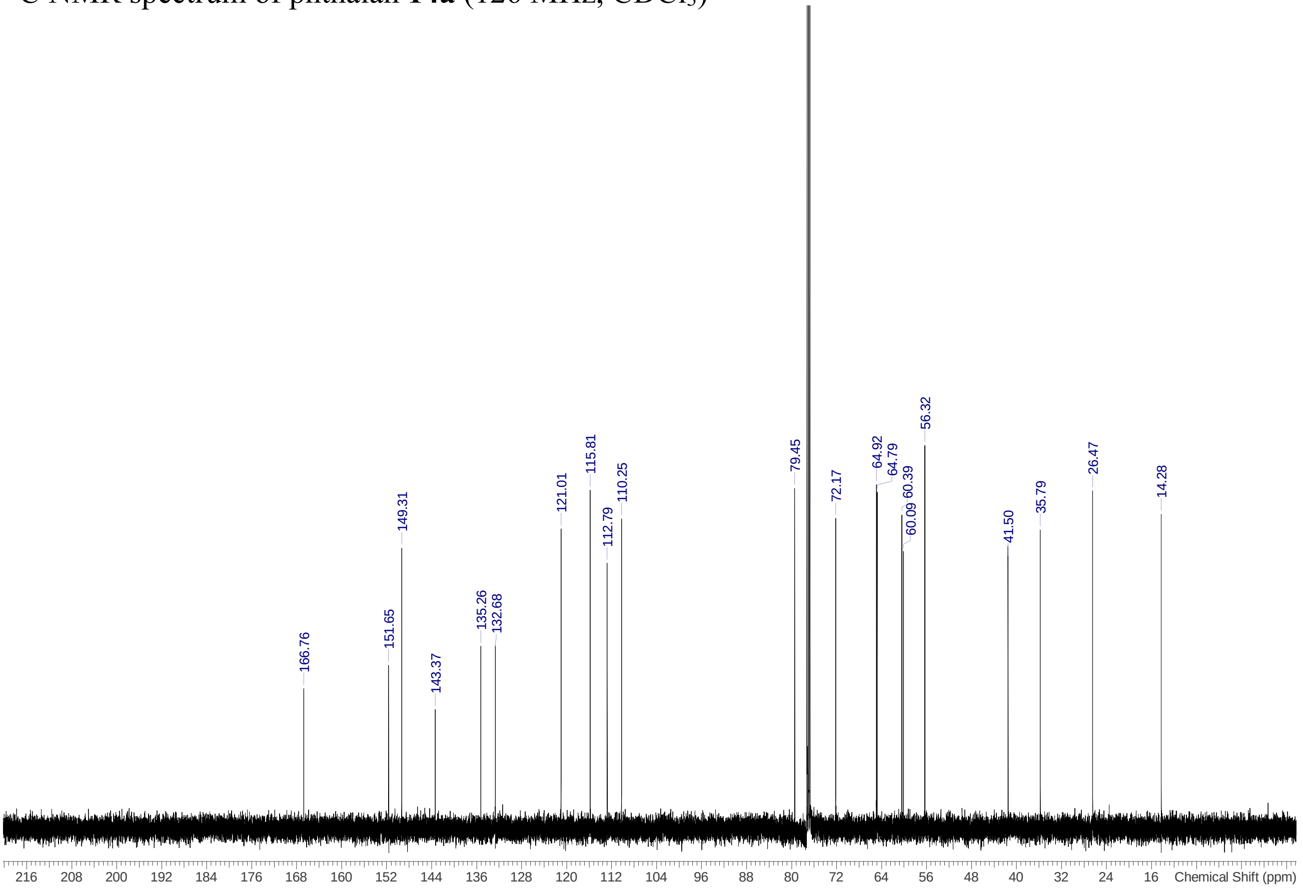

^{1}H NMR spectrum of phthalan **15a** (500 MHz, $CDCl_3$)

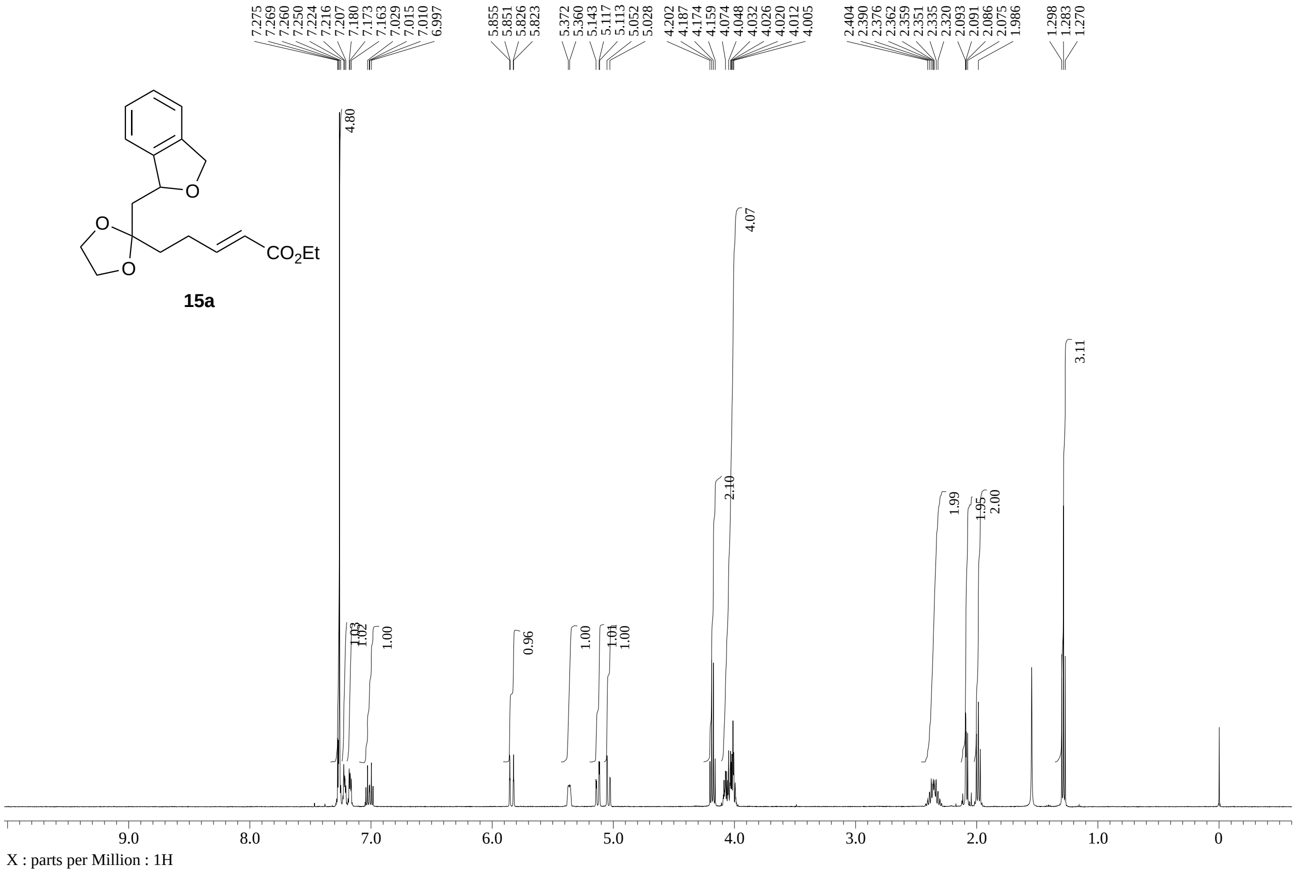

^{13}C NMR spectrum of phthalan **15a** (126 MHz, $CDCl_3$)

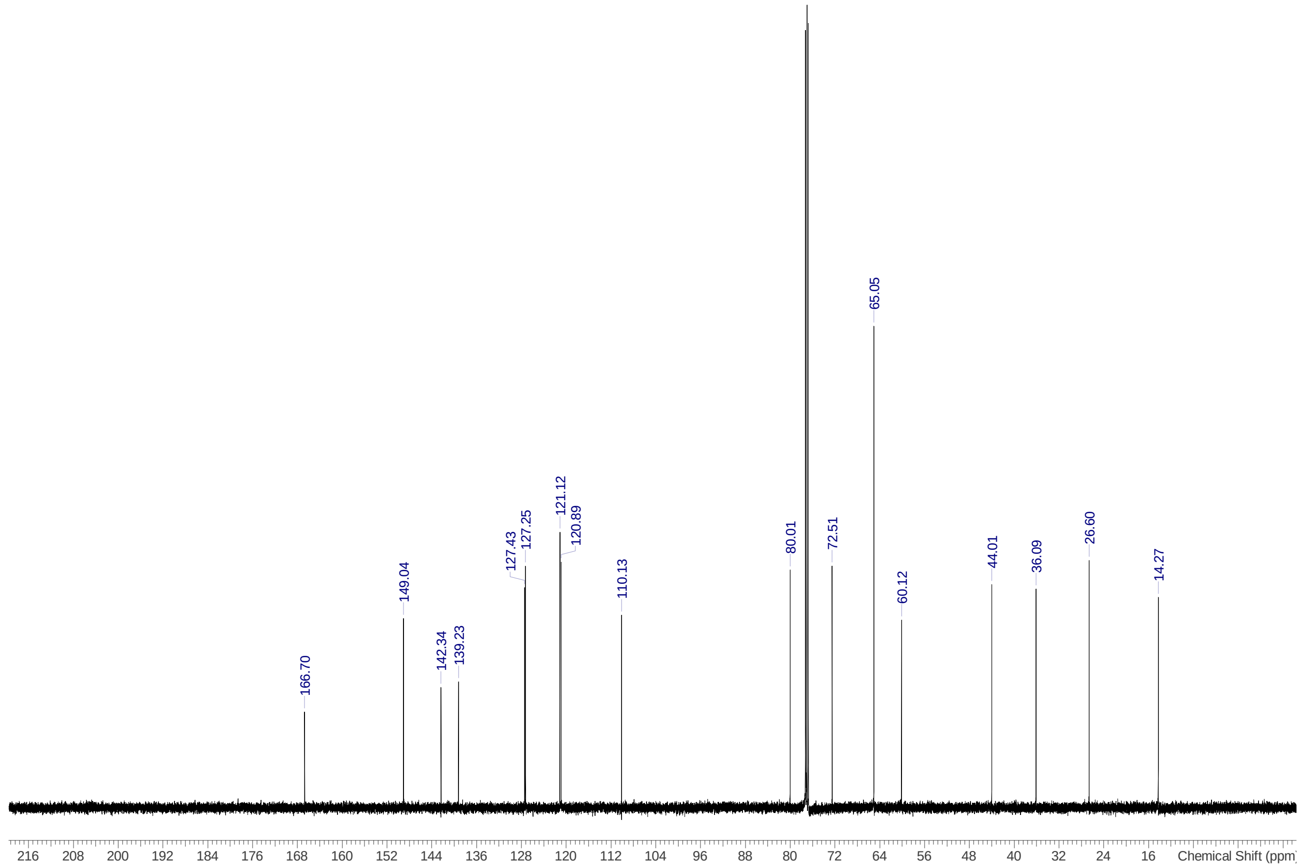

^{1}H NMR spectrum of aldehyde **S15** (500 MHz, $CDCl_3$)

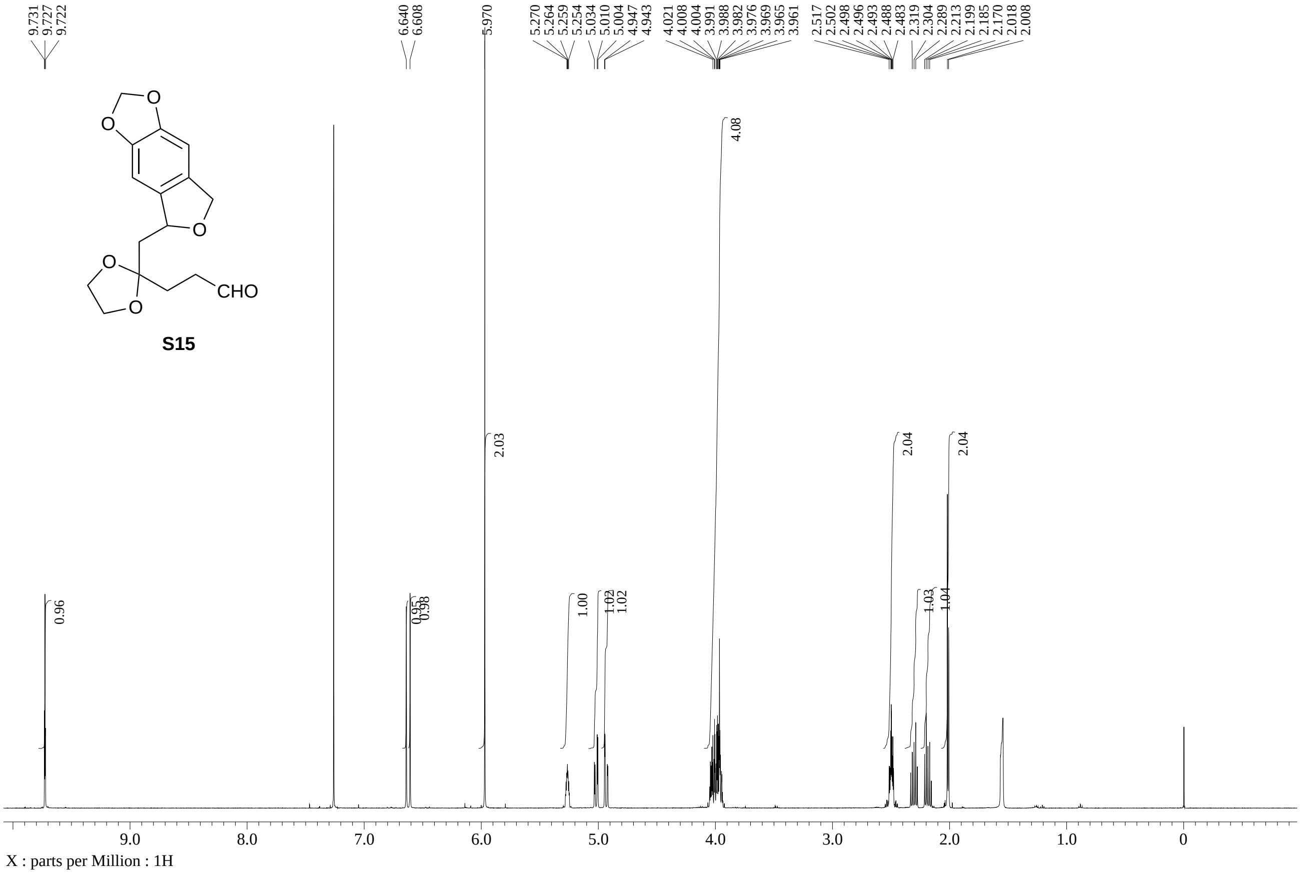

^{13}C NMR spectrum of aldehyde **S15** (126 MHz, $CDCl_3$)

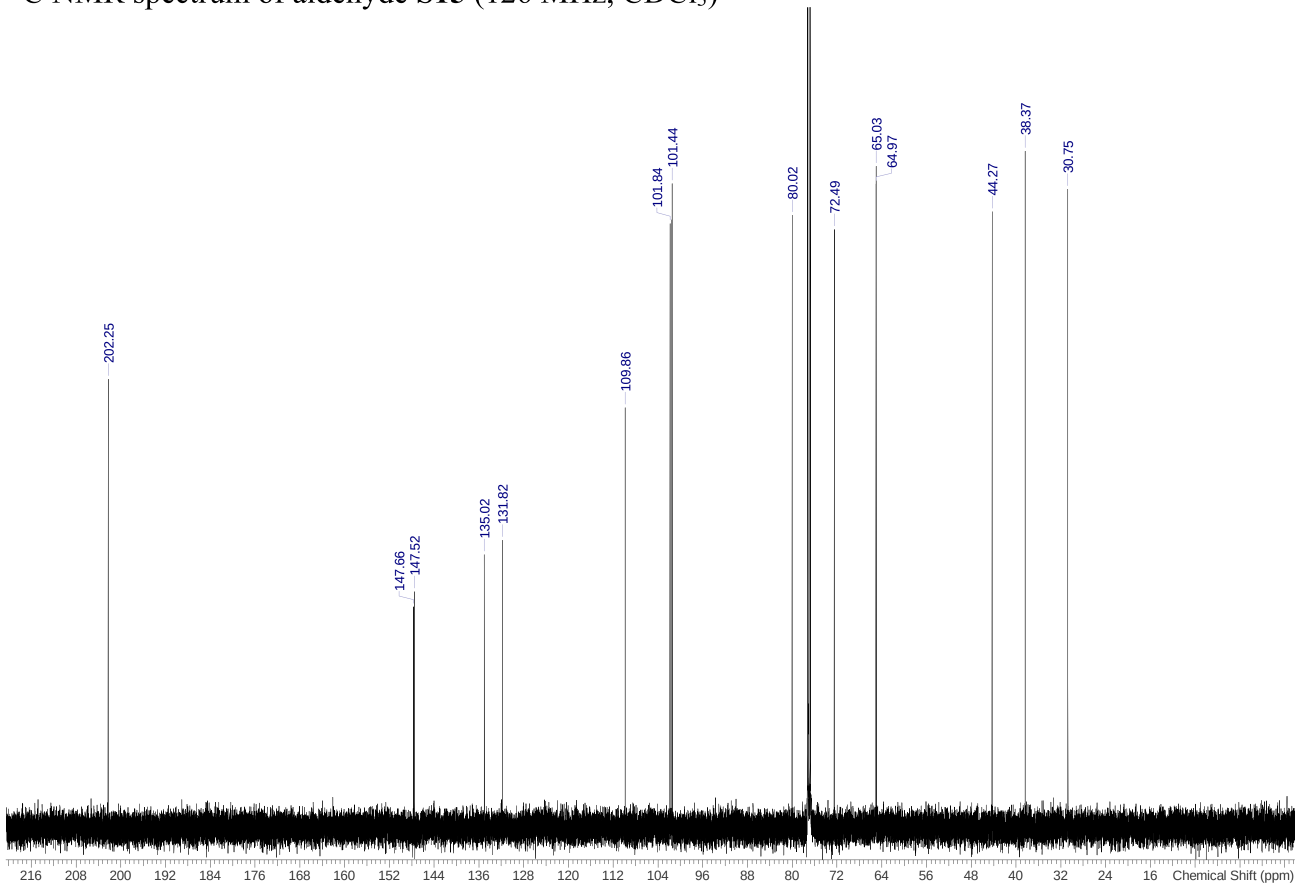

^{1}H NMR spectrum of phthalan **5a** (500 MHz, $CDCl_3$)

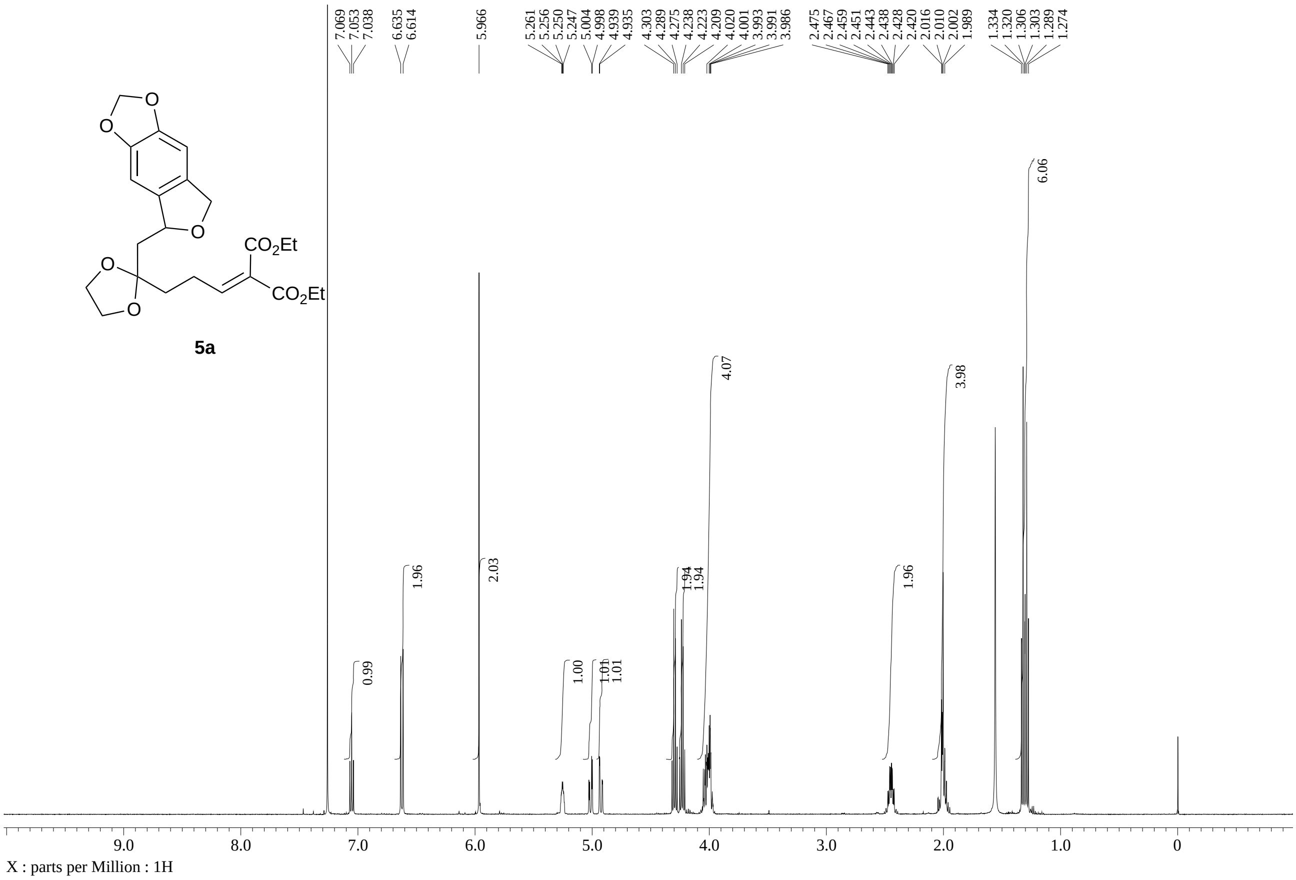

^{13}C NMR spectrum of phthalan **5a** (126 MHz, $CDCl_3$)

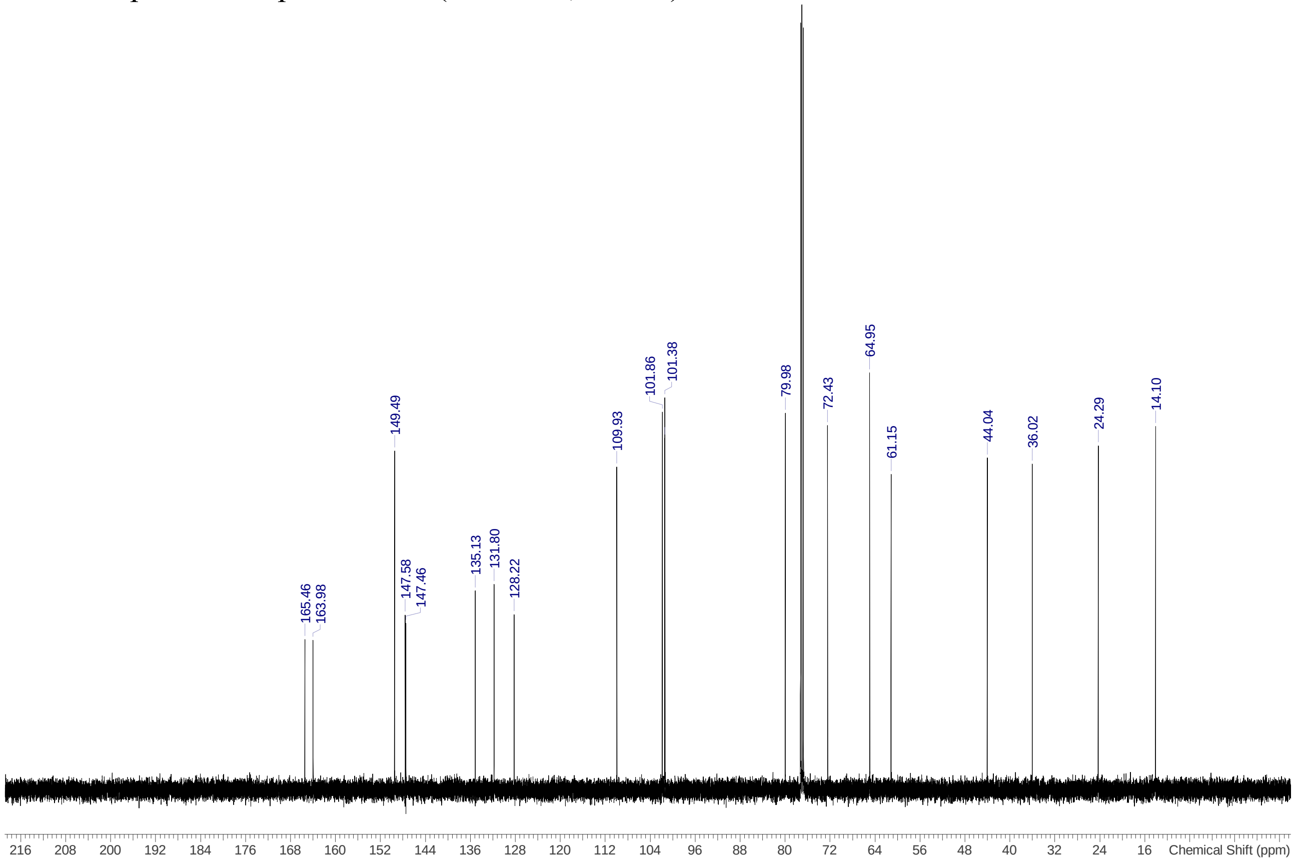

^{1}H NMR spectrum of phthalan **6a** (500 MHz, $CDCl_3$)

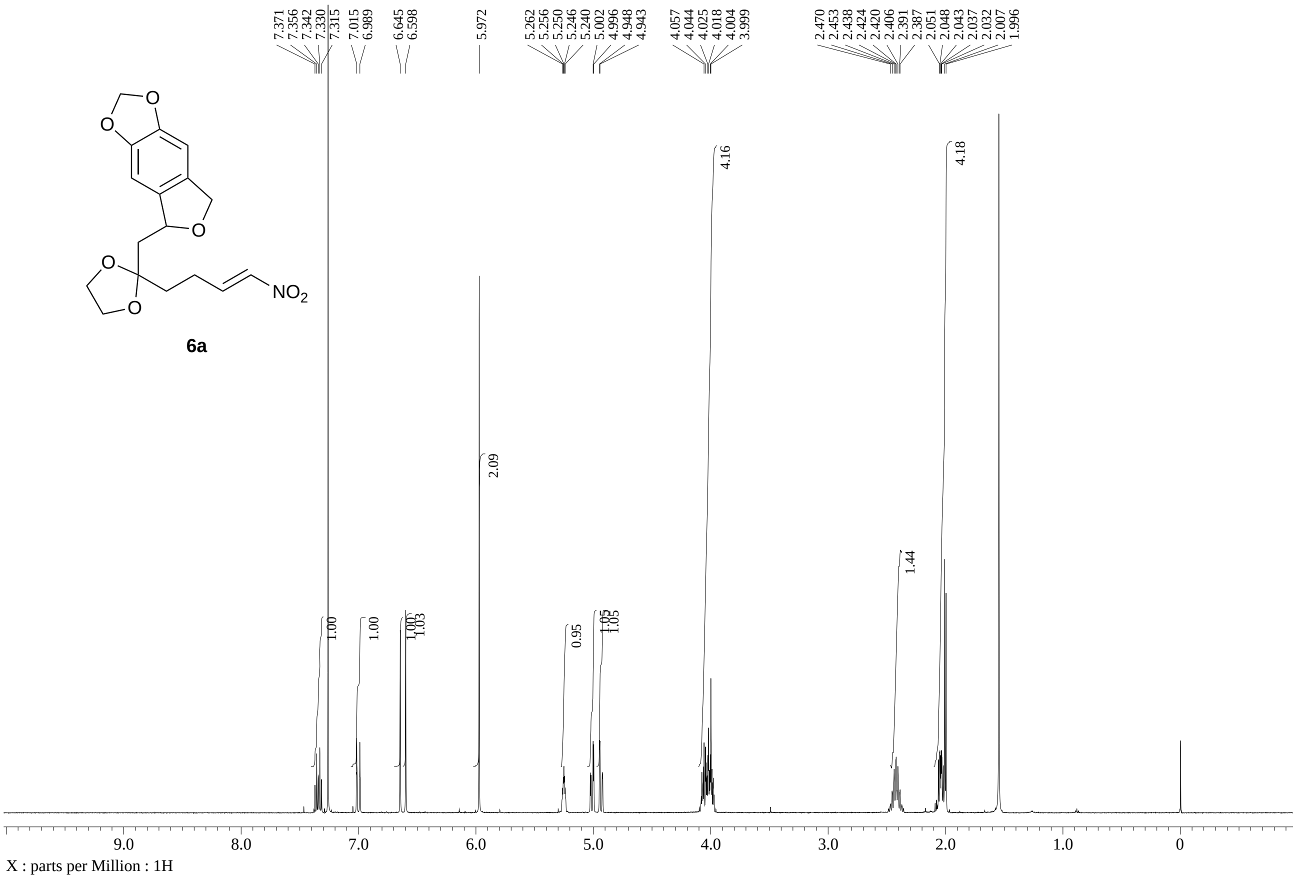

^{13}C NMR spectrum of phthalan **6a** (126 MHz, $CDCl_3$)

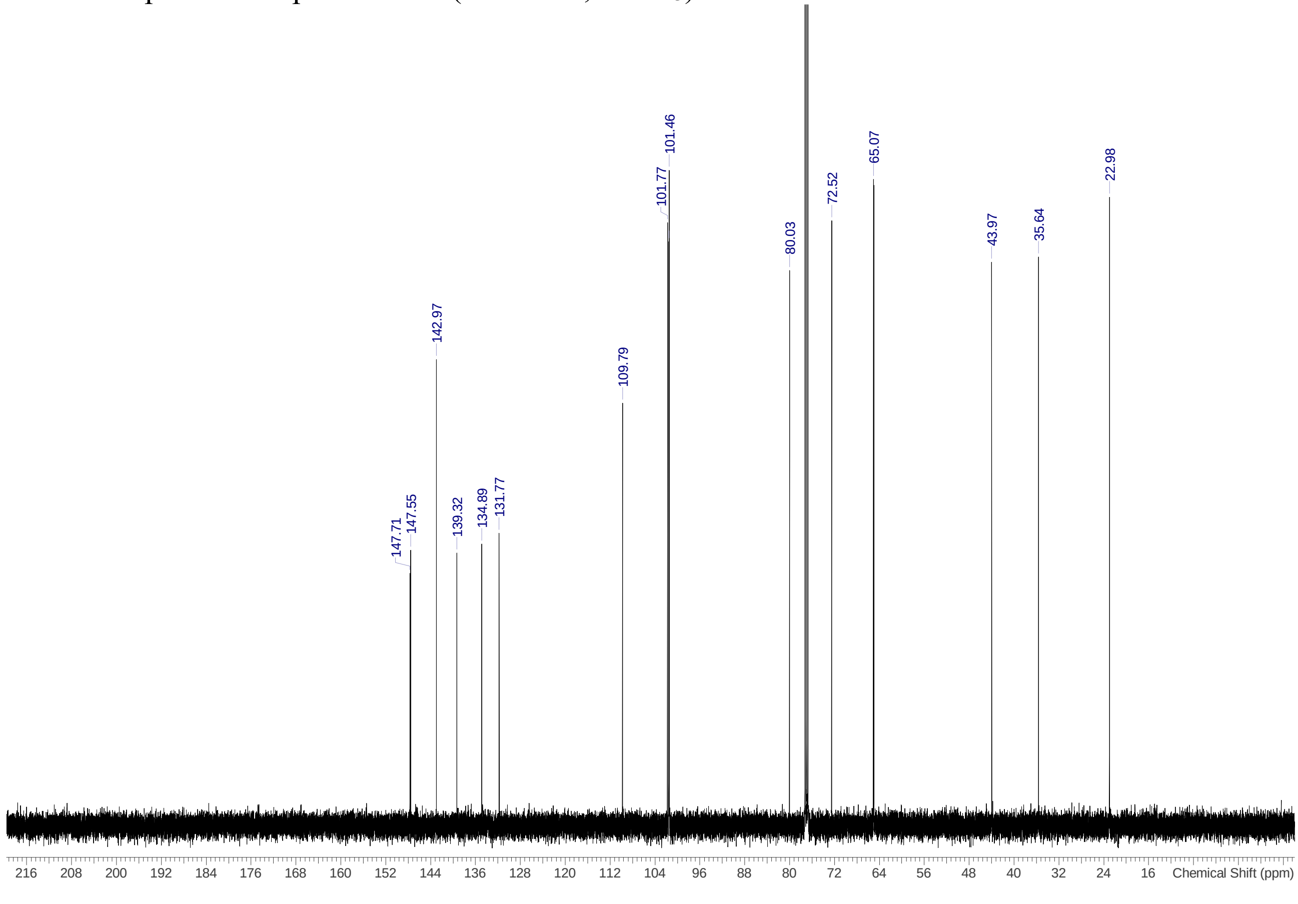

^{1}H NMR spectrum of phthalan **7a** (500 MHz, $CDCl_3$)

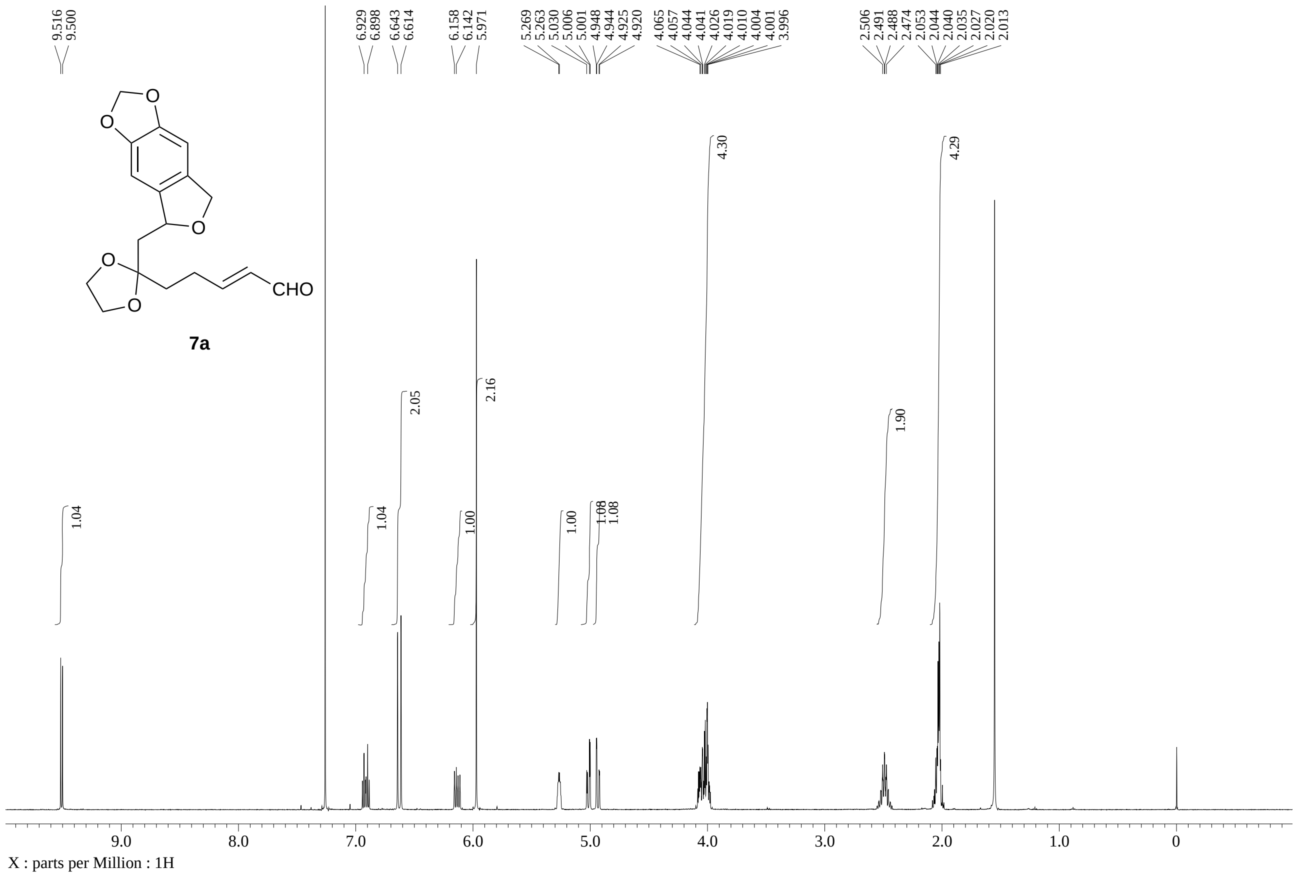

^{13}C NMR spectrum of phthalan **7a** (126 MHz, $CDCl_3$)

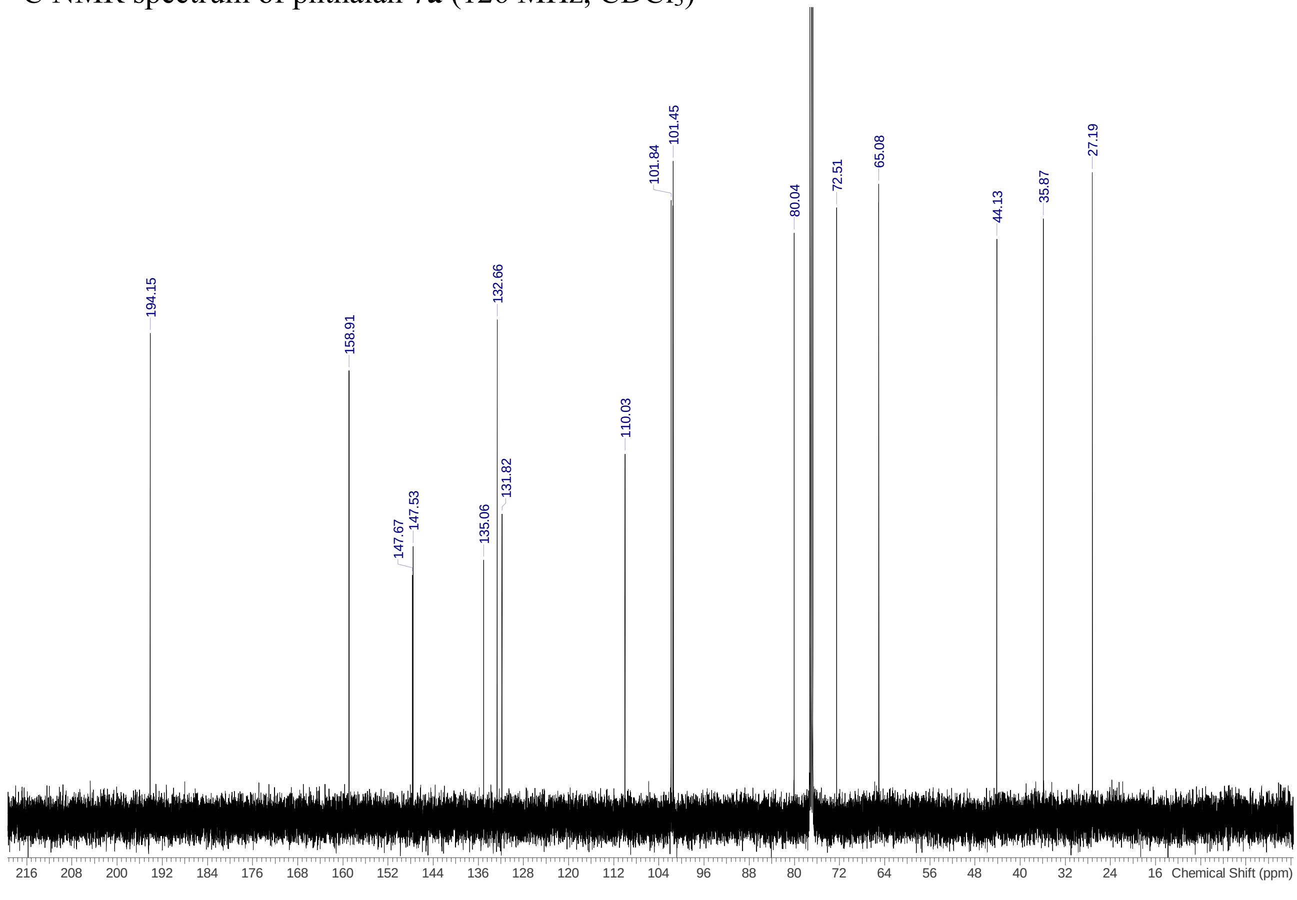

^{1}H NMR spectrum of ketone **S16** (500 MHz, $CDCl_3$)

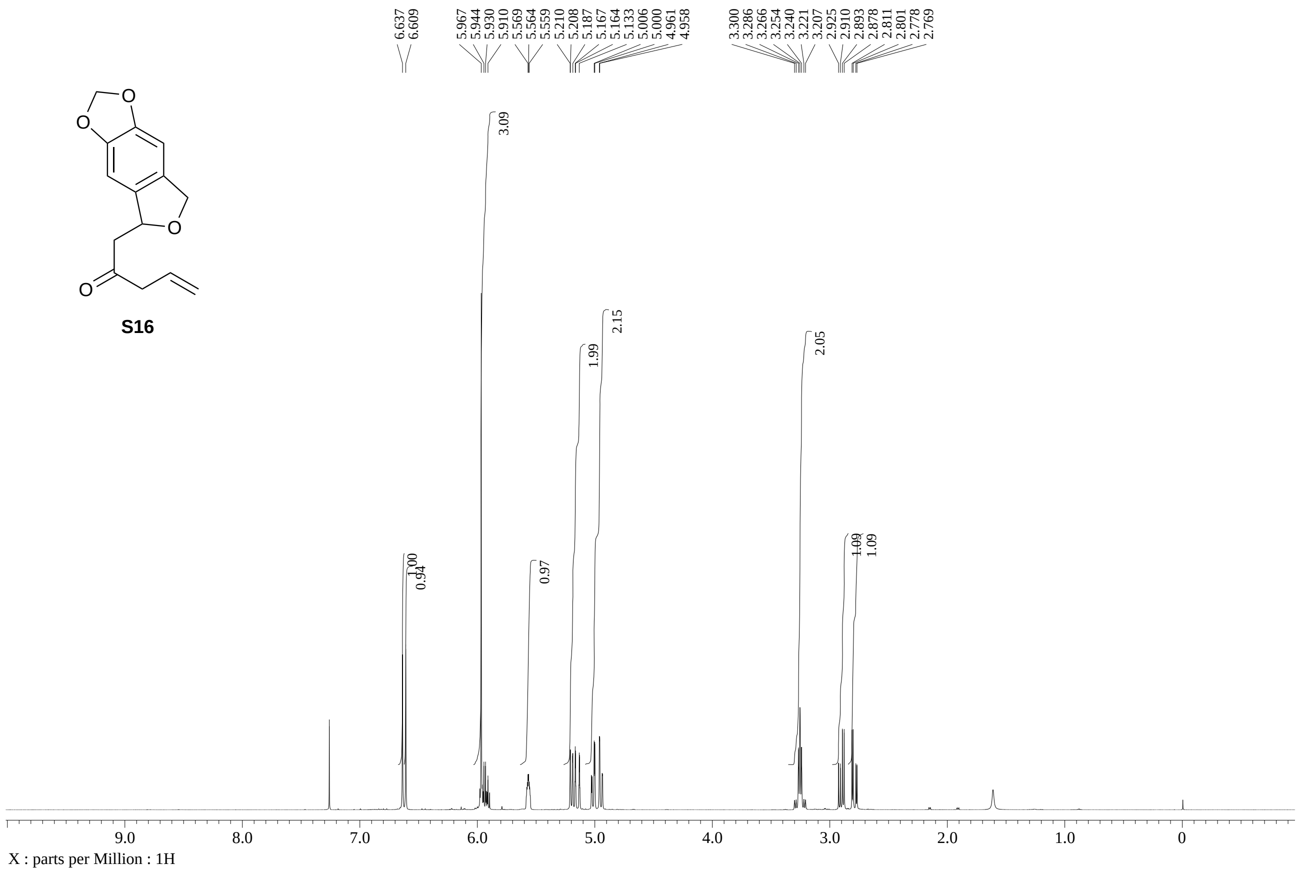

^{13}C NMR spectrum of ketone **S16** (126 MHz, $CDCl_3$)

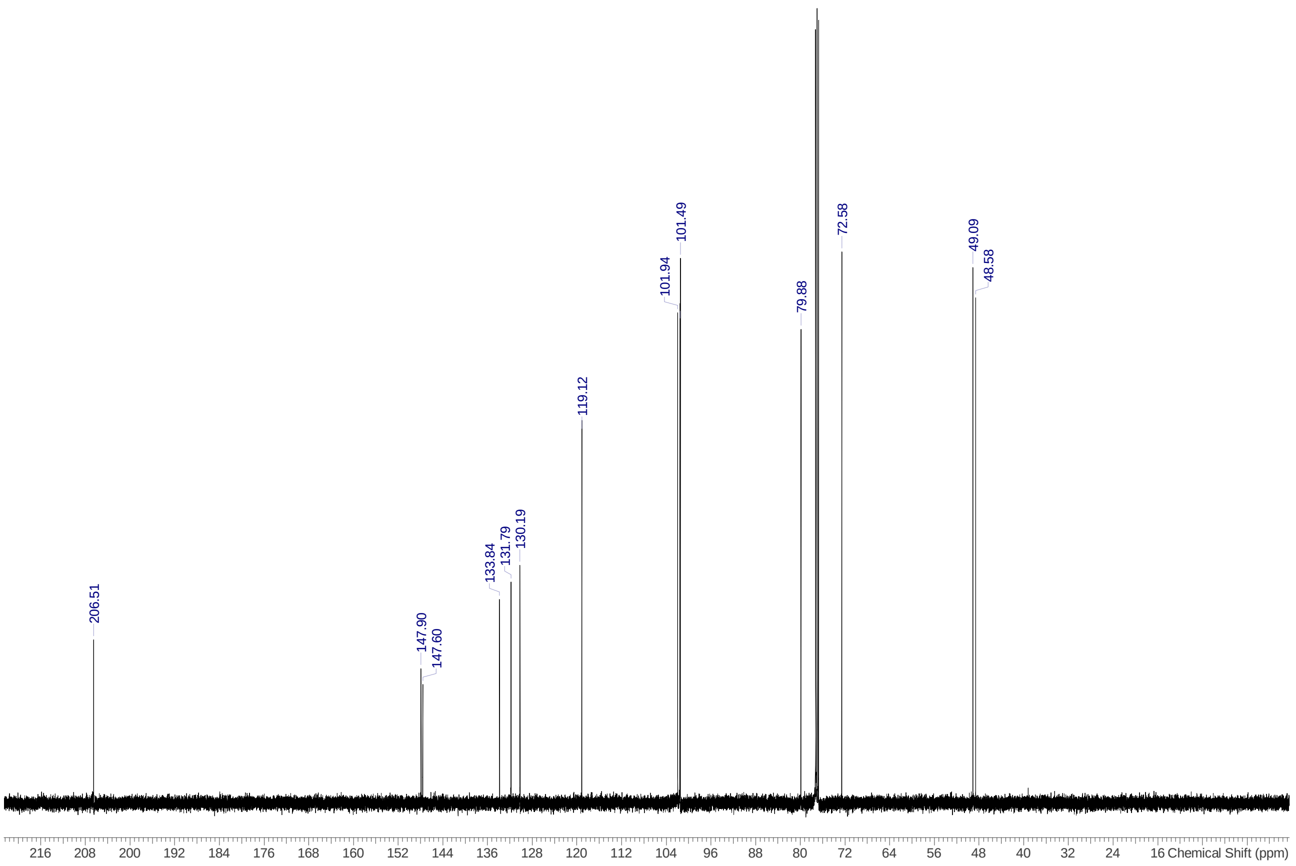

^{1}H NMR spectrum of acetal **S17** (500 MHz, $CDCl_3$)

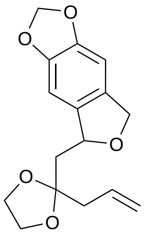

S17

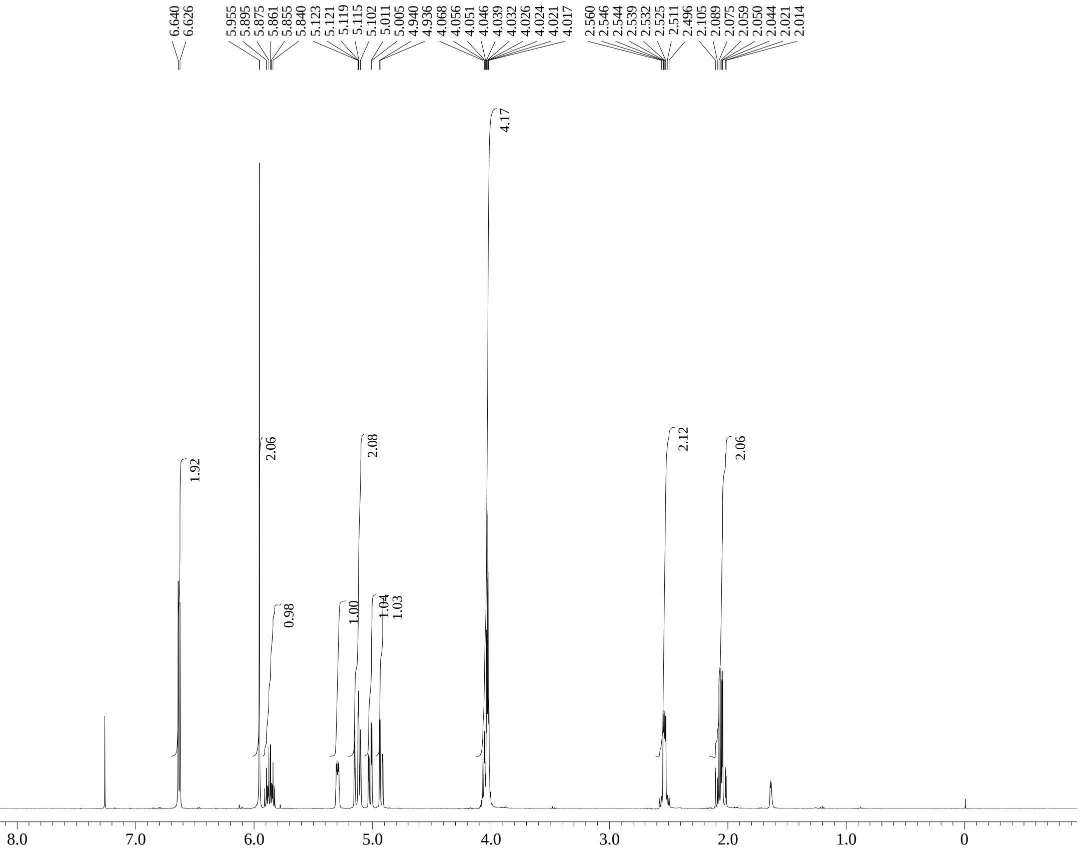

^{13}C NMR spectrum of acetal **S17** (126 MHz, $CDCl_3$)

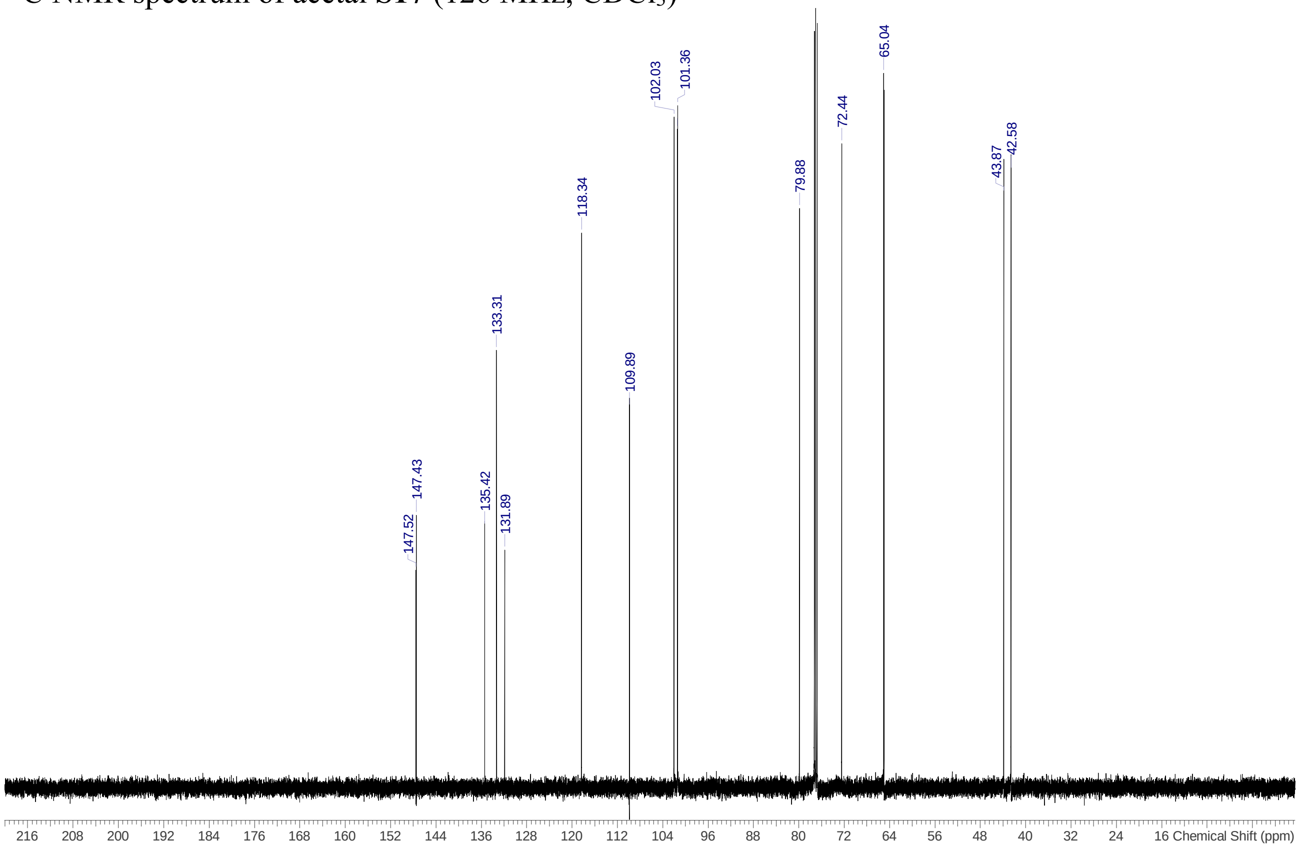

^{1}H NMR spectrum of aldehyde **S18** (500 MHz, $CDCl_3$)

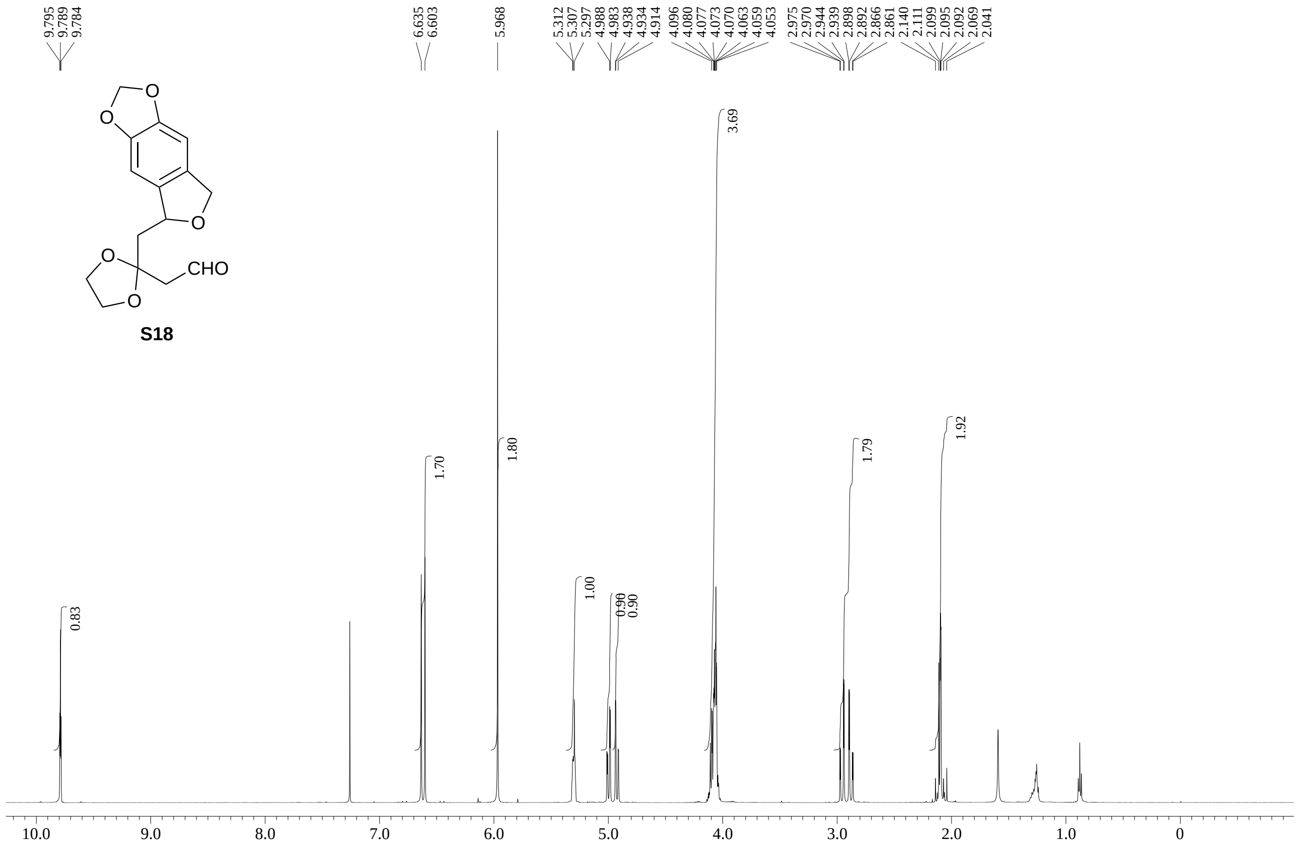

^{13}C NMR spectrum of aldehyde **S18** (126 MHz, $CDCl_3$)

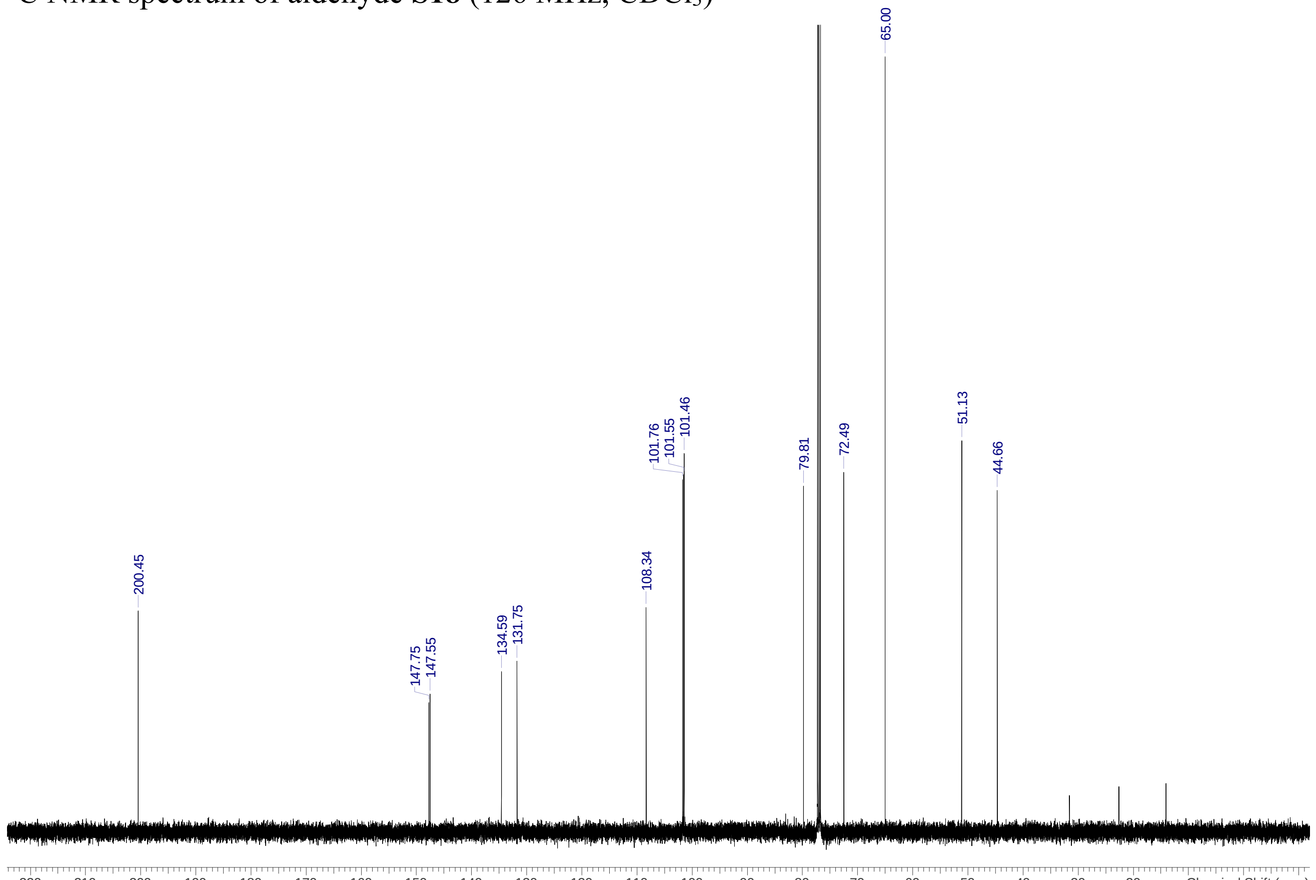

^{1}H NMR spectrum of alcohol **S19** (500 MHz, $CDCl_3$)

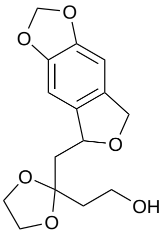

S19

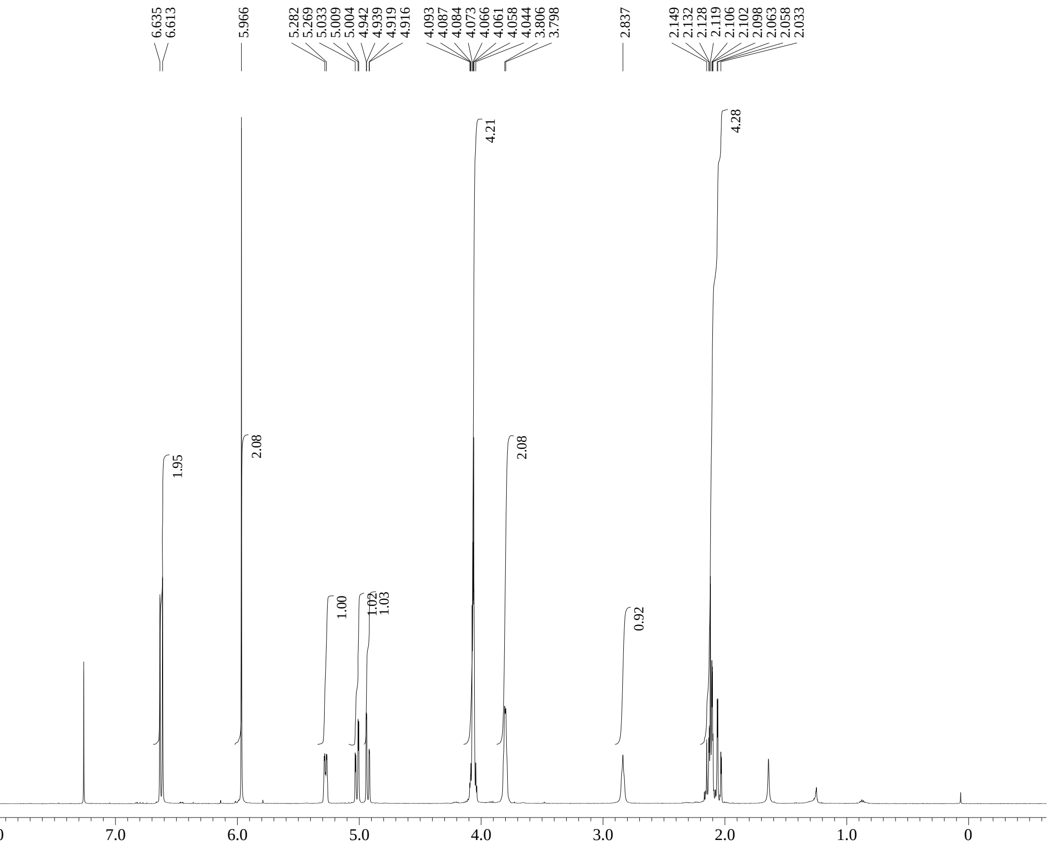

^{13}C NMR spectrum of alcohol **S19** (126 MHz, $CDCl_3$)

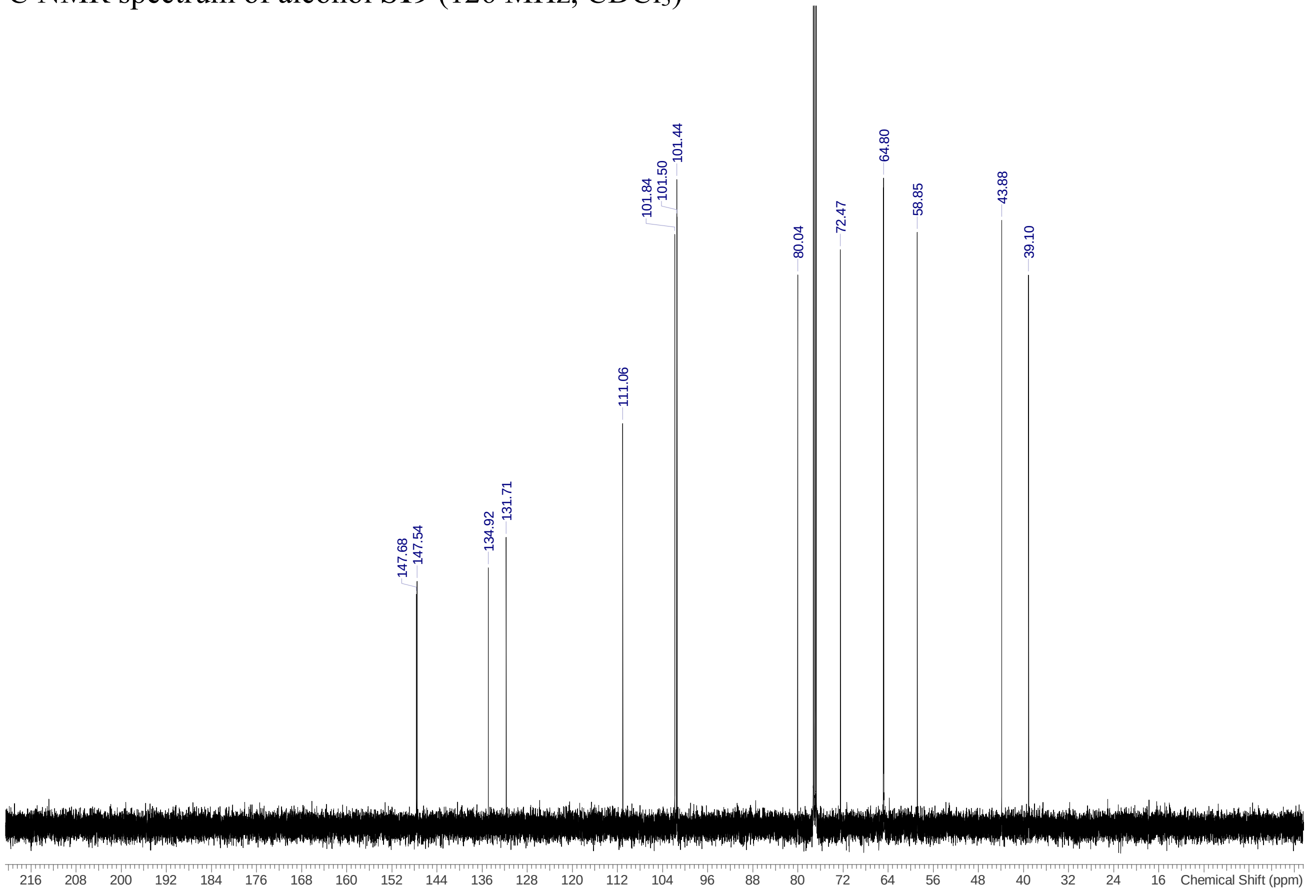

^{1}H NMR spectrum of iodide **S20** (500 MHz, $CDCl_3$)

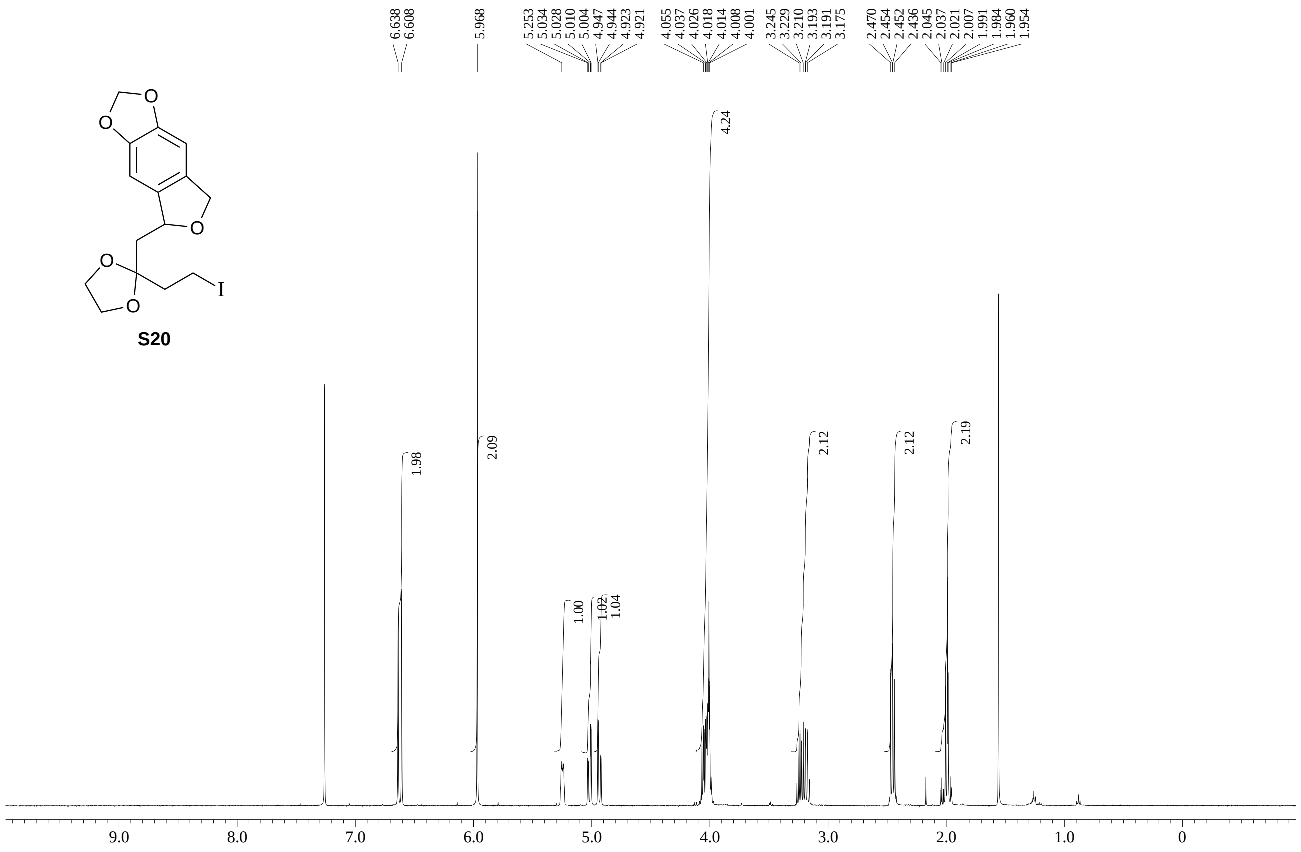

^{13}C NMR spectrum of iodide **S20** (126 MHz, $CDCl_3$)

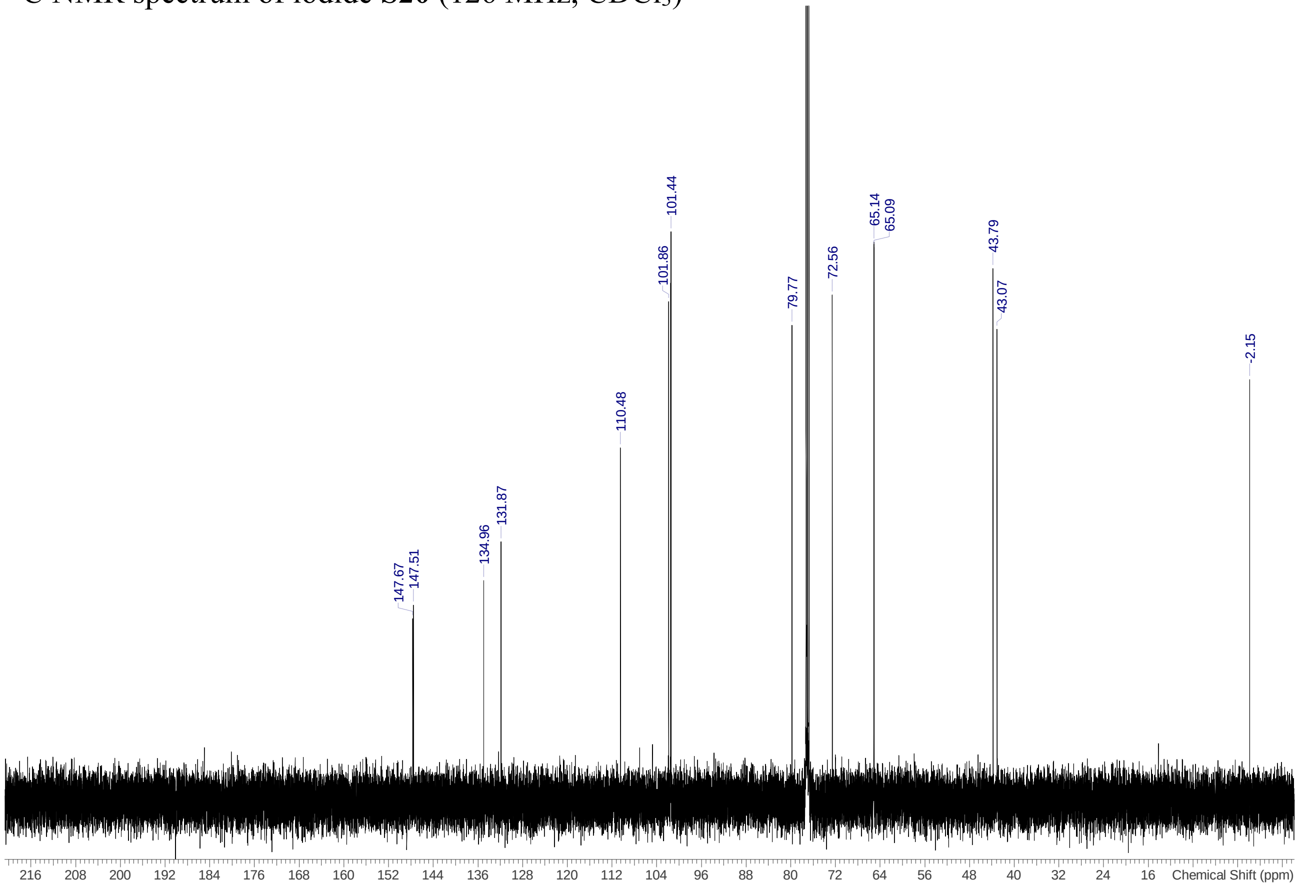

^{1}H NMR spectrum of phthalan **9a** (500 MHz, $CDCl_3$)

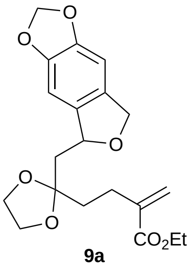

9a

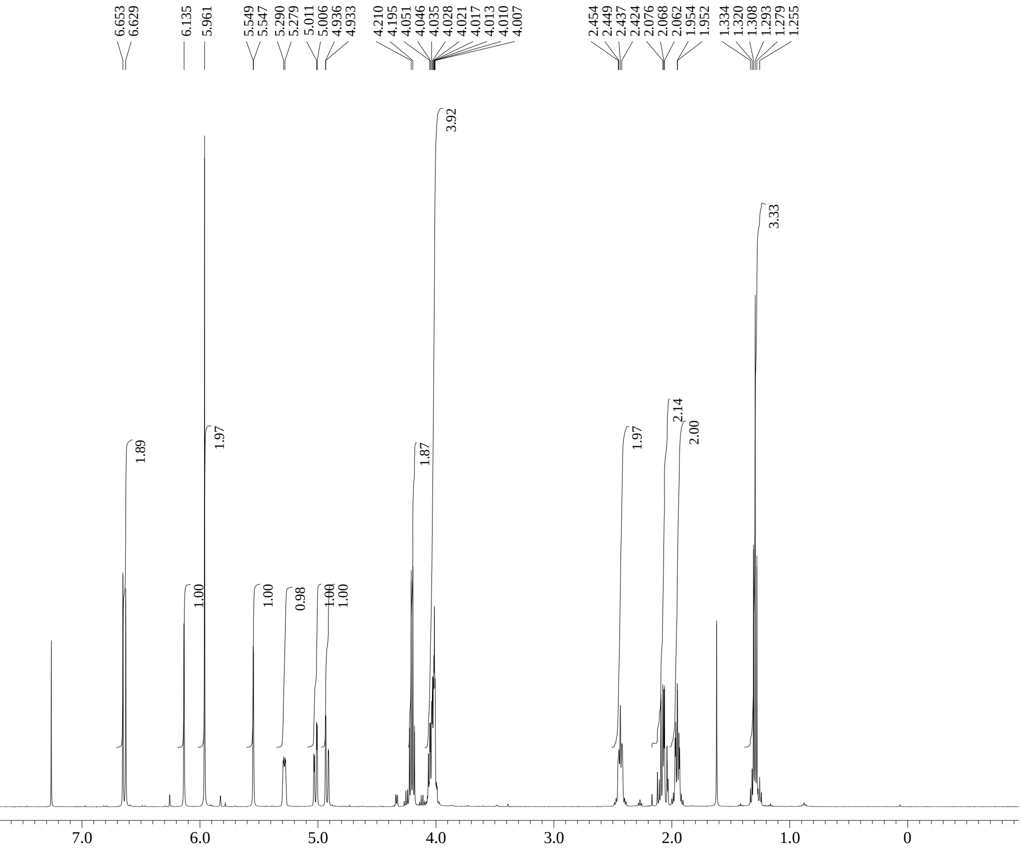

^{13}C NMR spectrum of phthalan **9a** (126 MHz, $CDCl_3$)

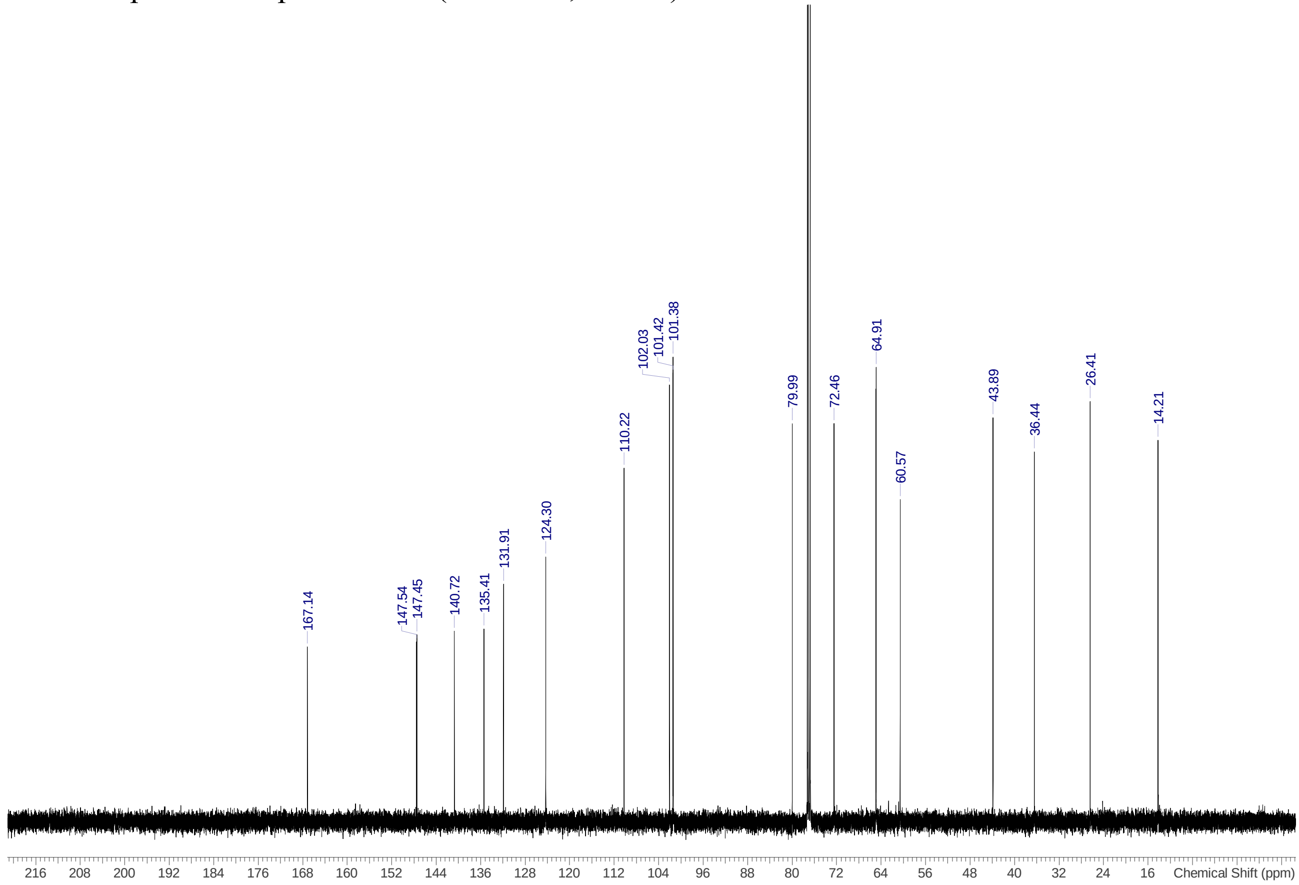

^{1}H NMR spectrum of γ-ketoester **S23** (500 MHz, $CDCl_3$)

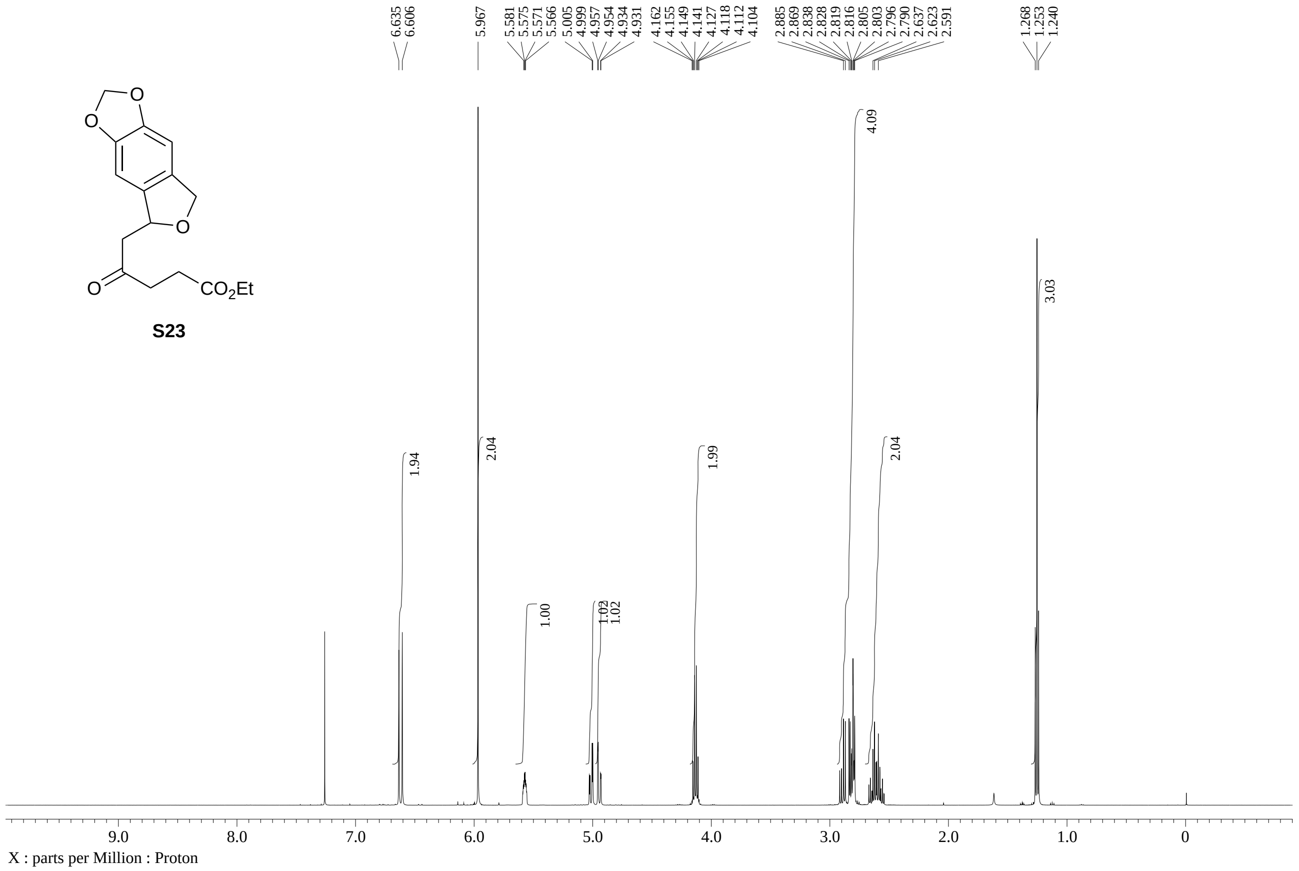

^{13}C NMR spectrum of γ-ketoester **S23** (126 MHz, $CDCl_3$)

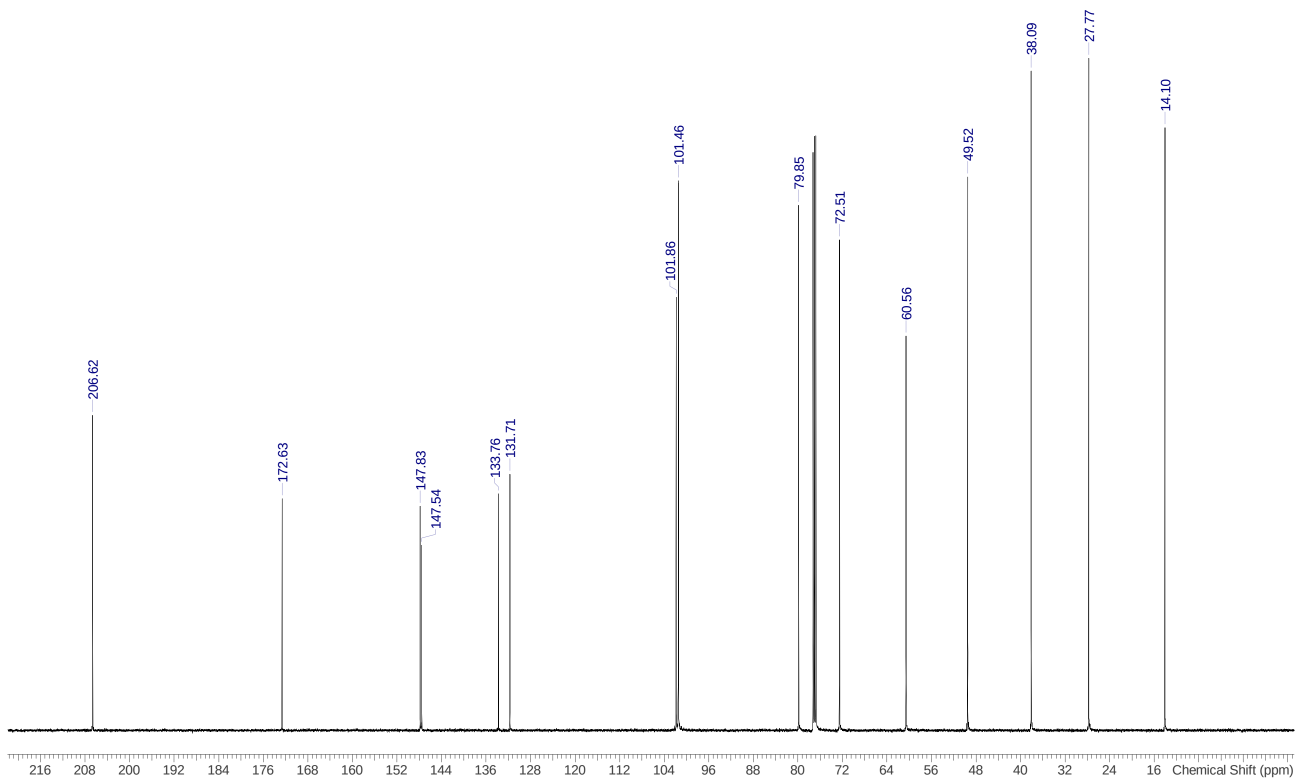

^{1}H NMR spectrum of hydroxyenone **S24** (500 MHz, $CDCl_3$)

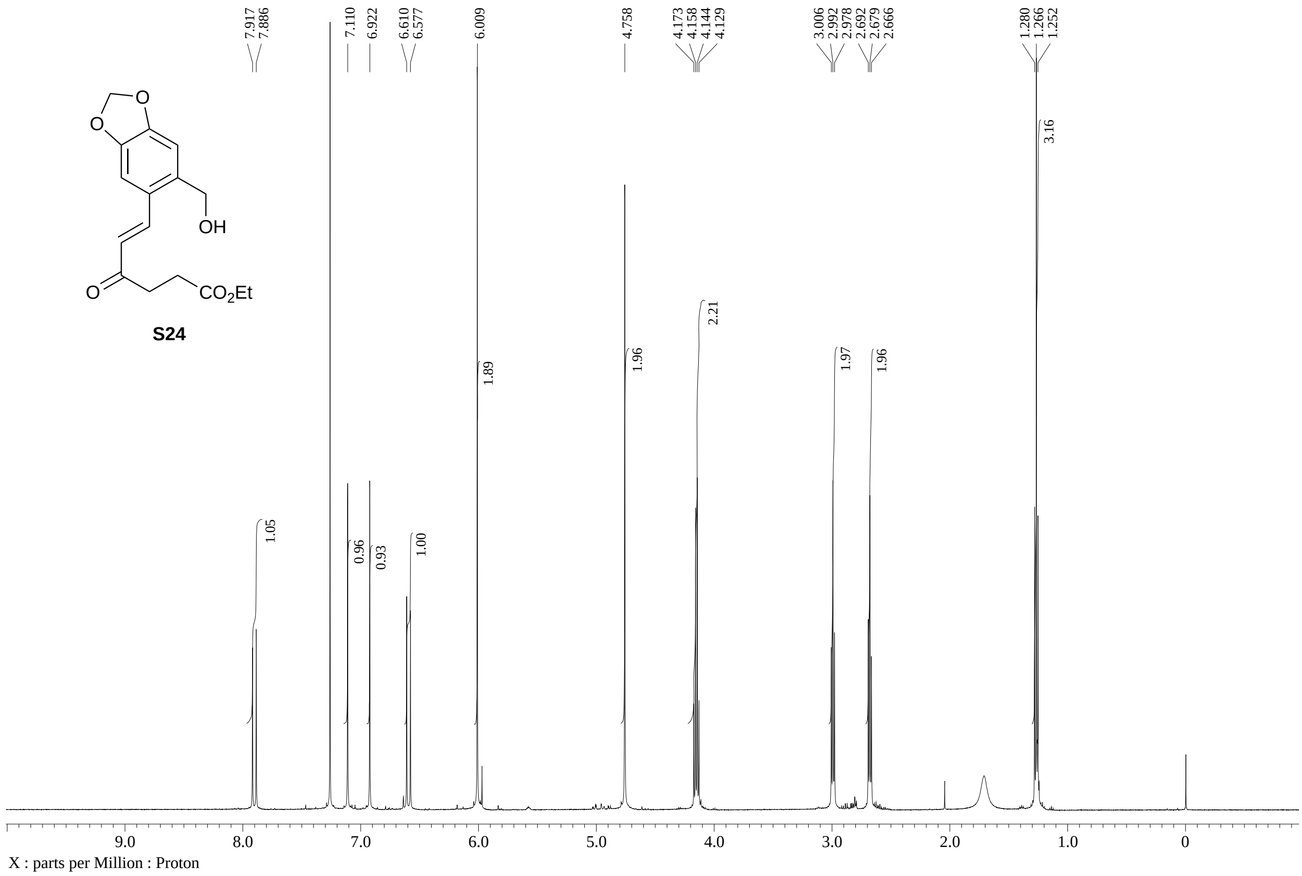

^{13}C NMR spectrum of hydroxyenone **S24** (126 MHz, $CDCl_3$)

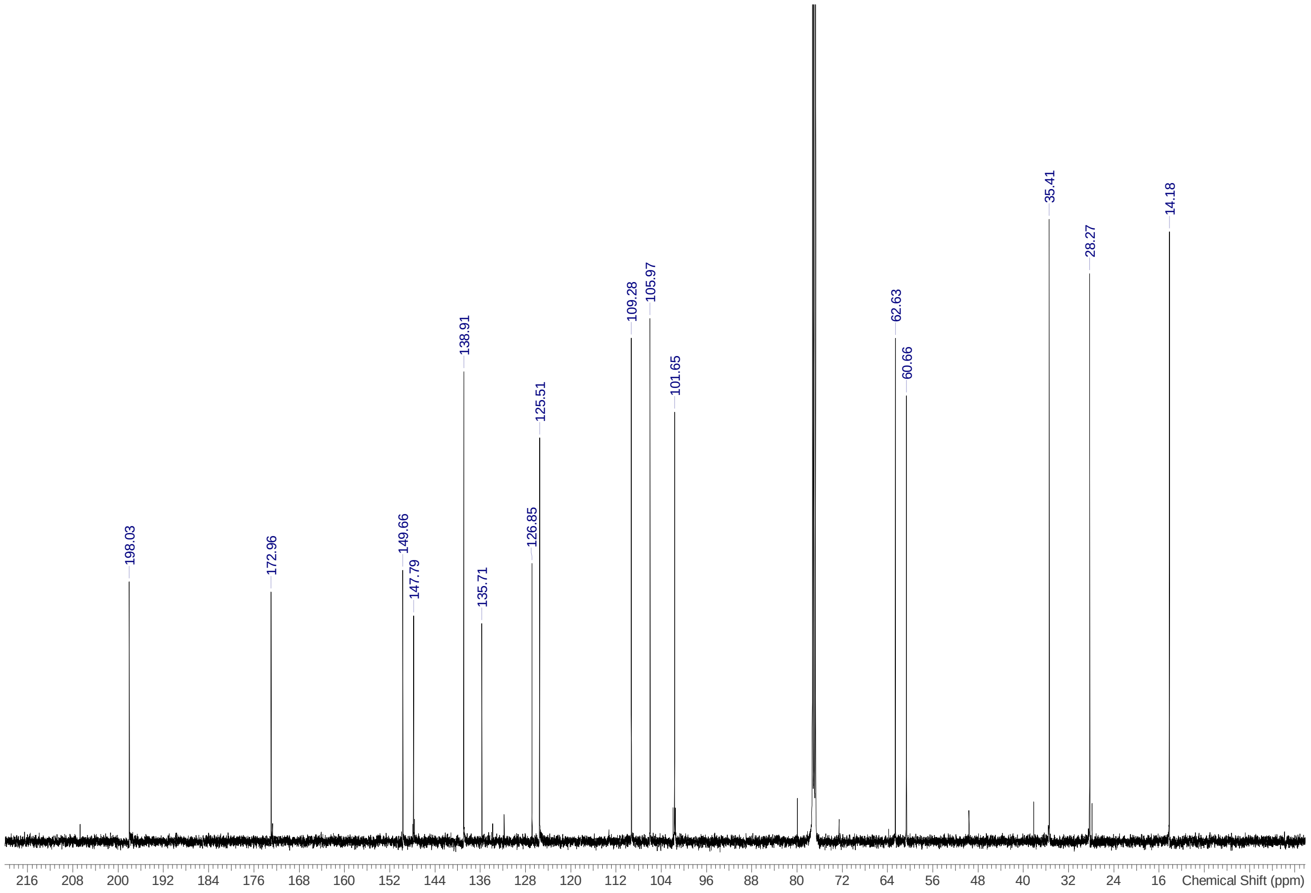

^{1}H NMR spectrum of acetal **S25** (500 MHz, $CDCl_3$)

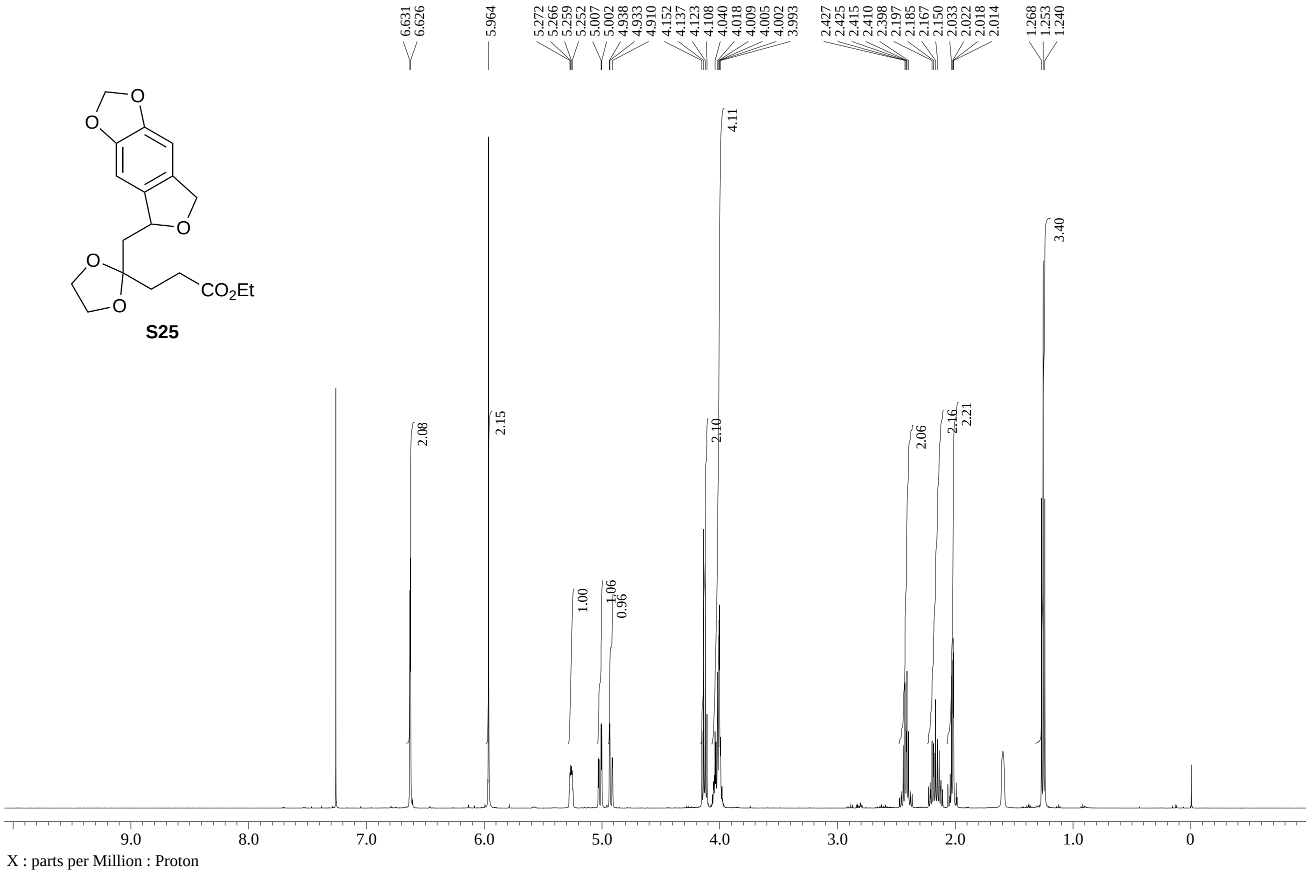

^{13}C NMR spectrum of acetal **S25** (126 MHz, $CDCl_3$)

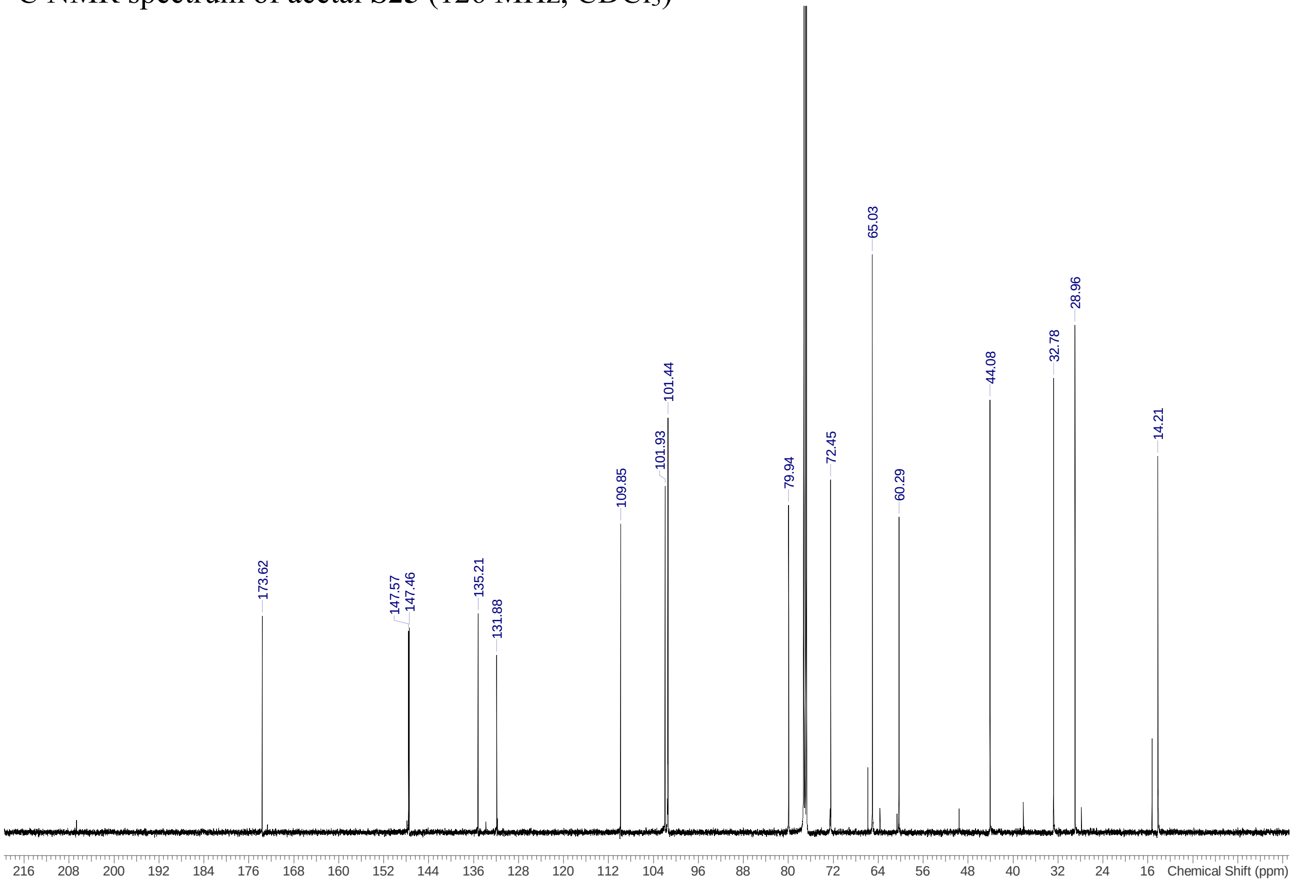

^{1}H NMR spectrum of Weinreb amide **S26** (500 MHz, $CDCl_3$)

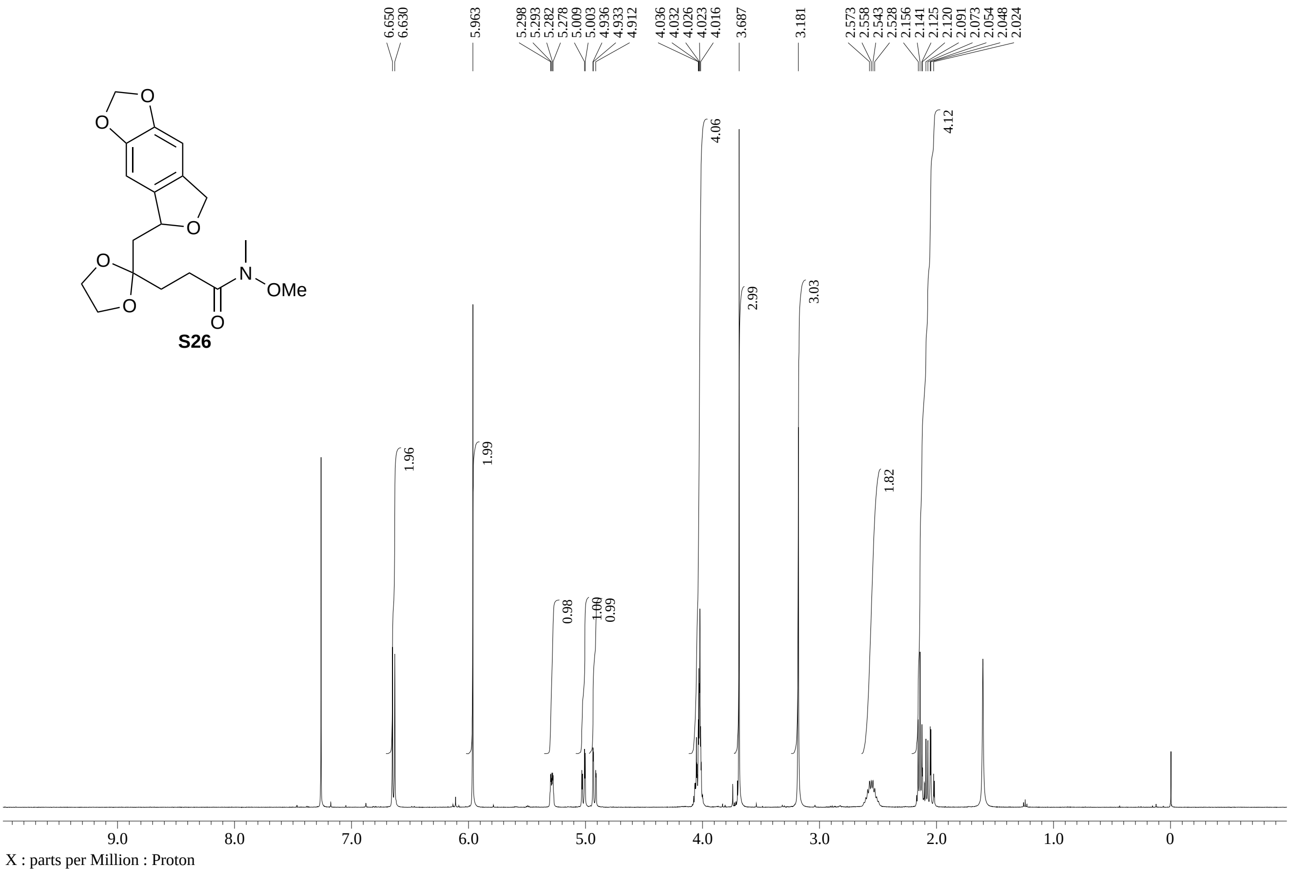

^{13}C NMR spectrum of Weinreb amide **S26** (126 MHz, $CDCl_3$)

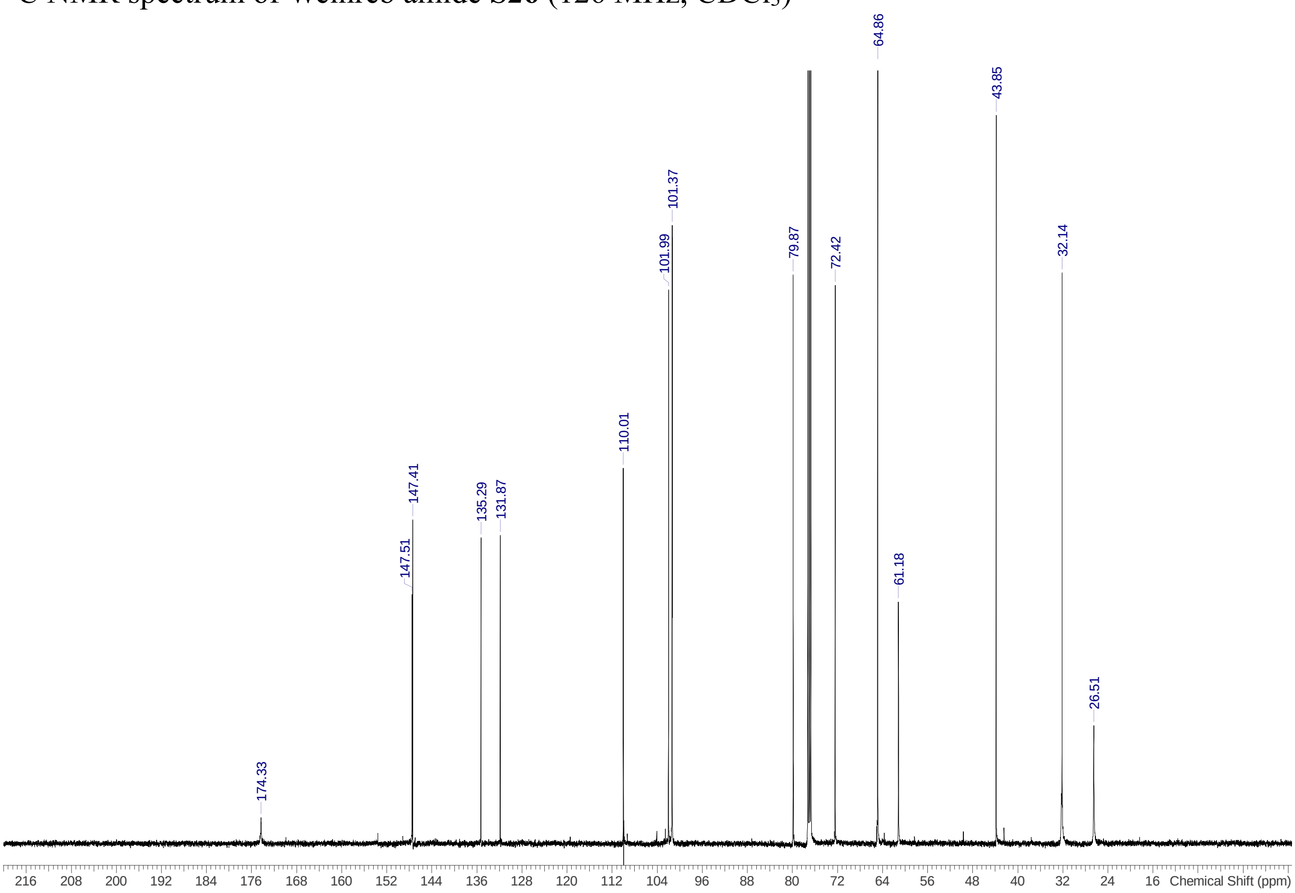

^{1}H NMR spectrum of phthalan **10a** (500 MHz, C_6D_6)

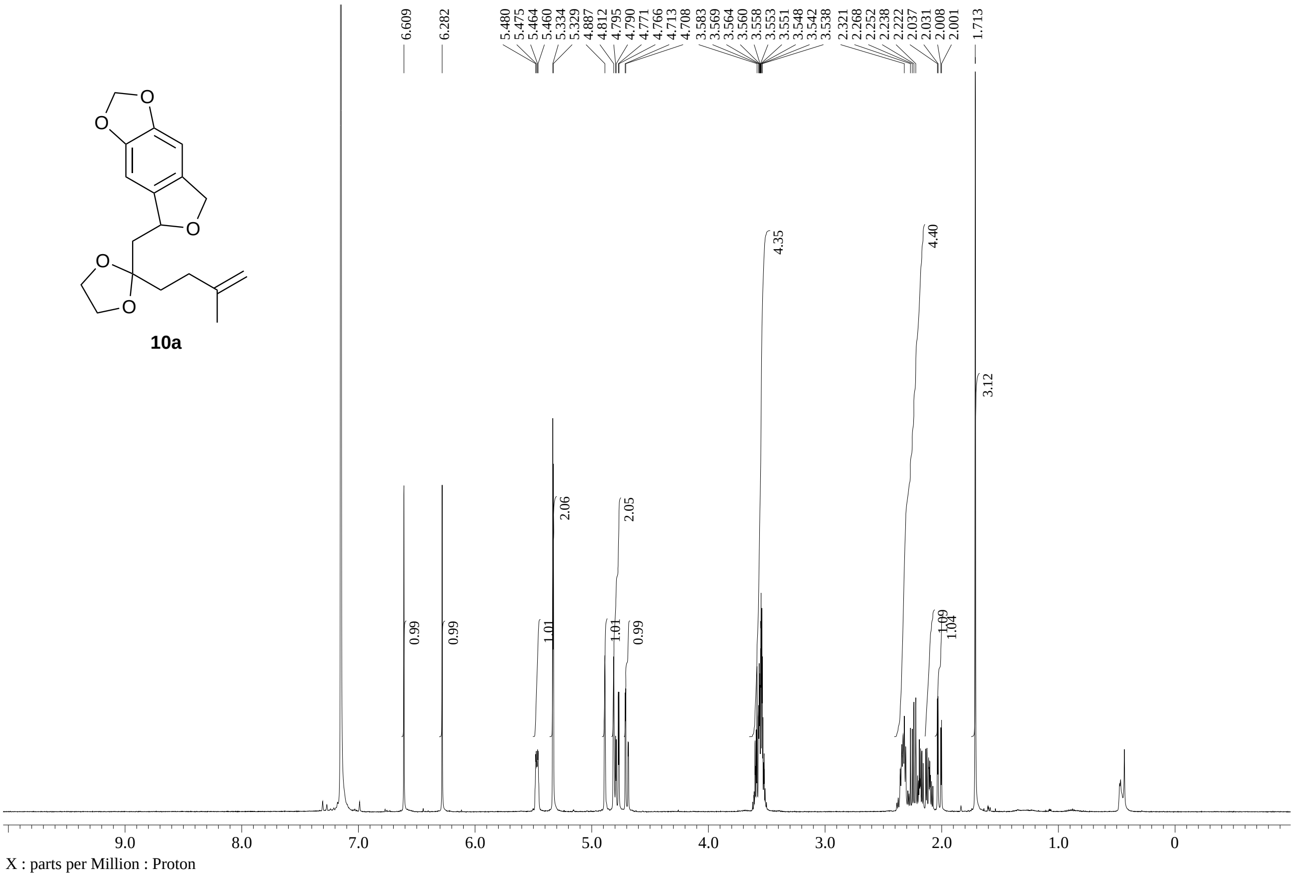

^{13}C NMR spectrum of phthalan **10a** (126 MHz, C_6D_6)

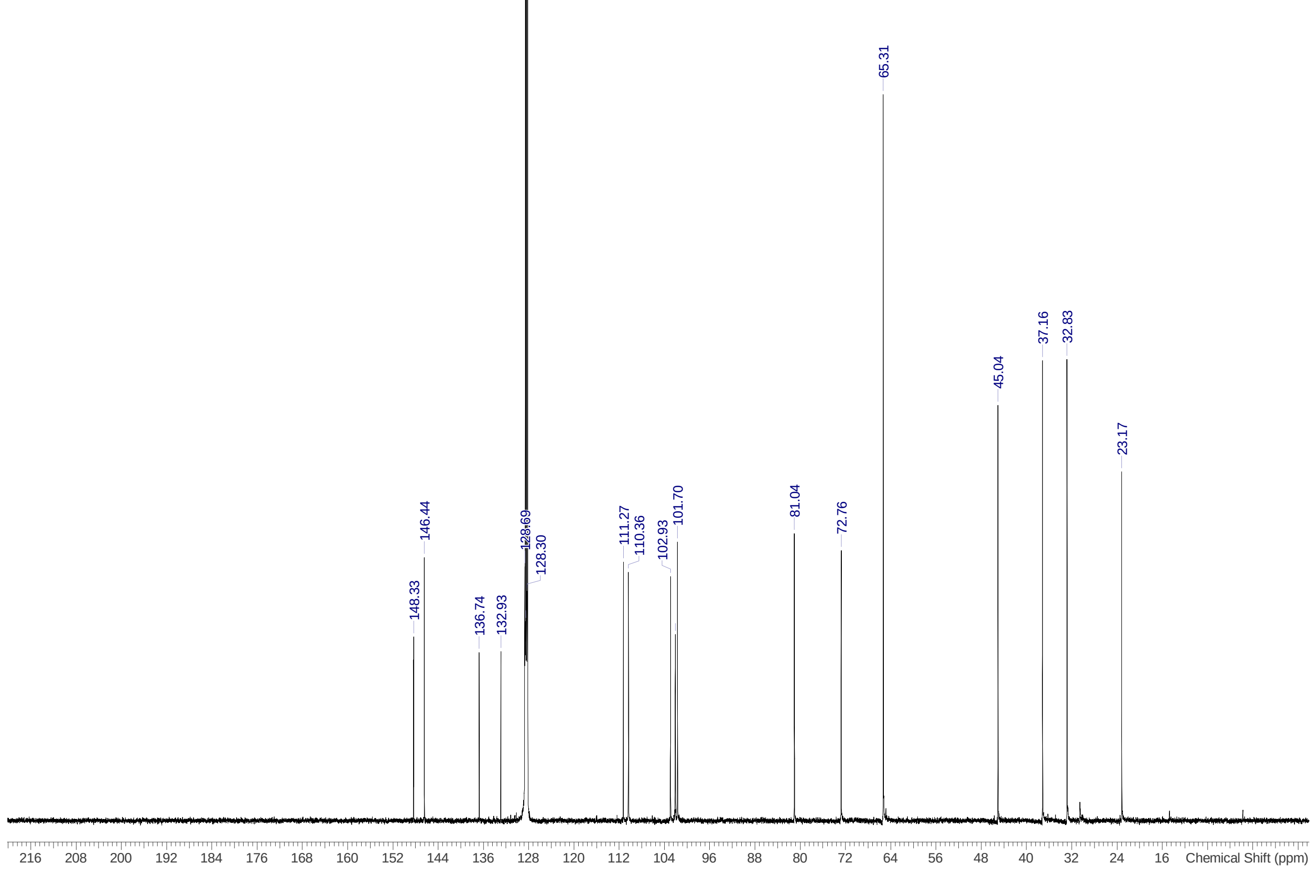

^{1}H NMR spectrum of phenyl ester **S27** (500 MHz, $CDCl_3$)

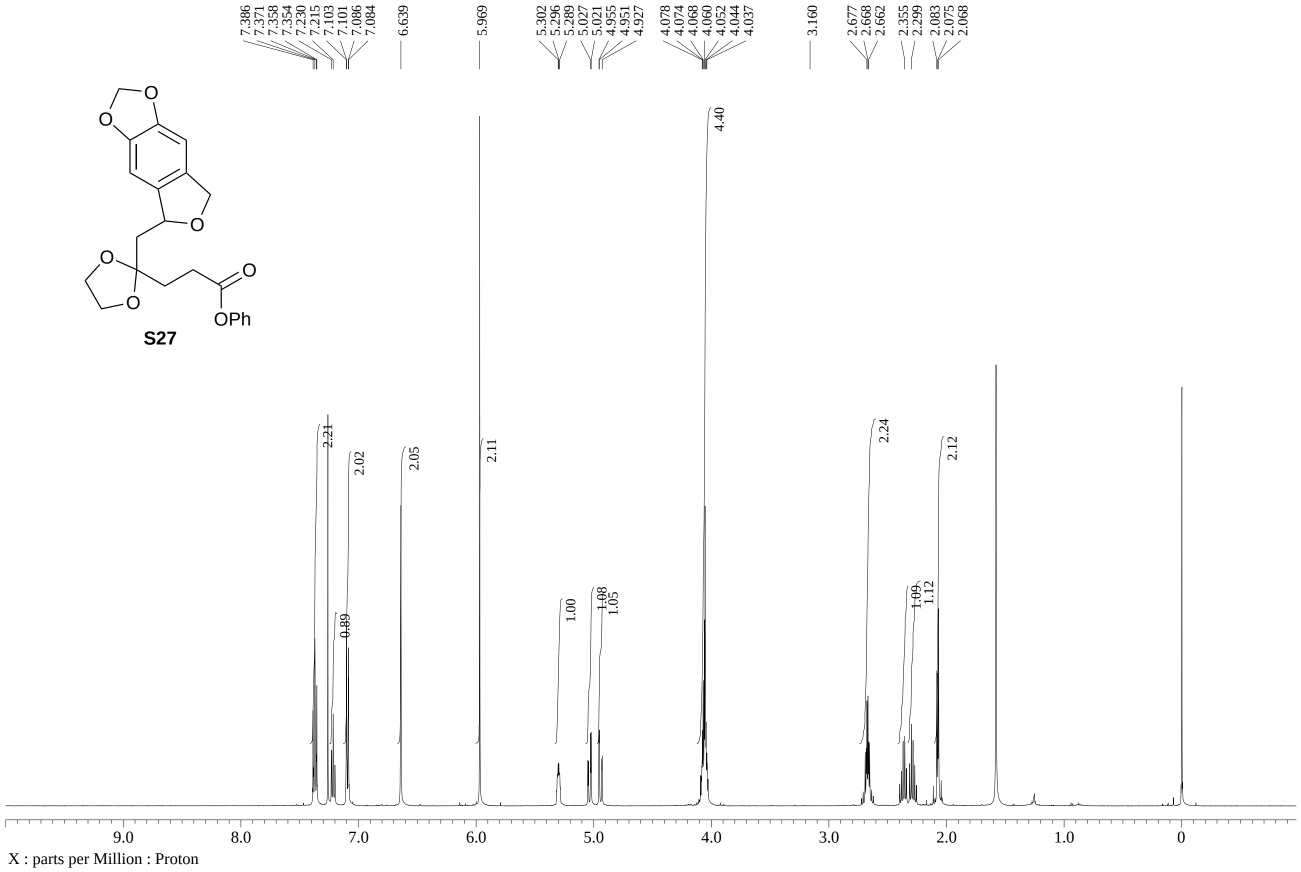

^{13}C NMR spectrum of phenyl ester **S27** (126 MHz, $CDCl_3$)

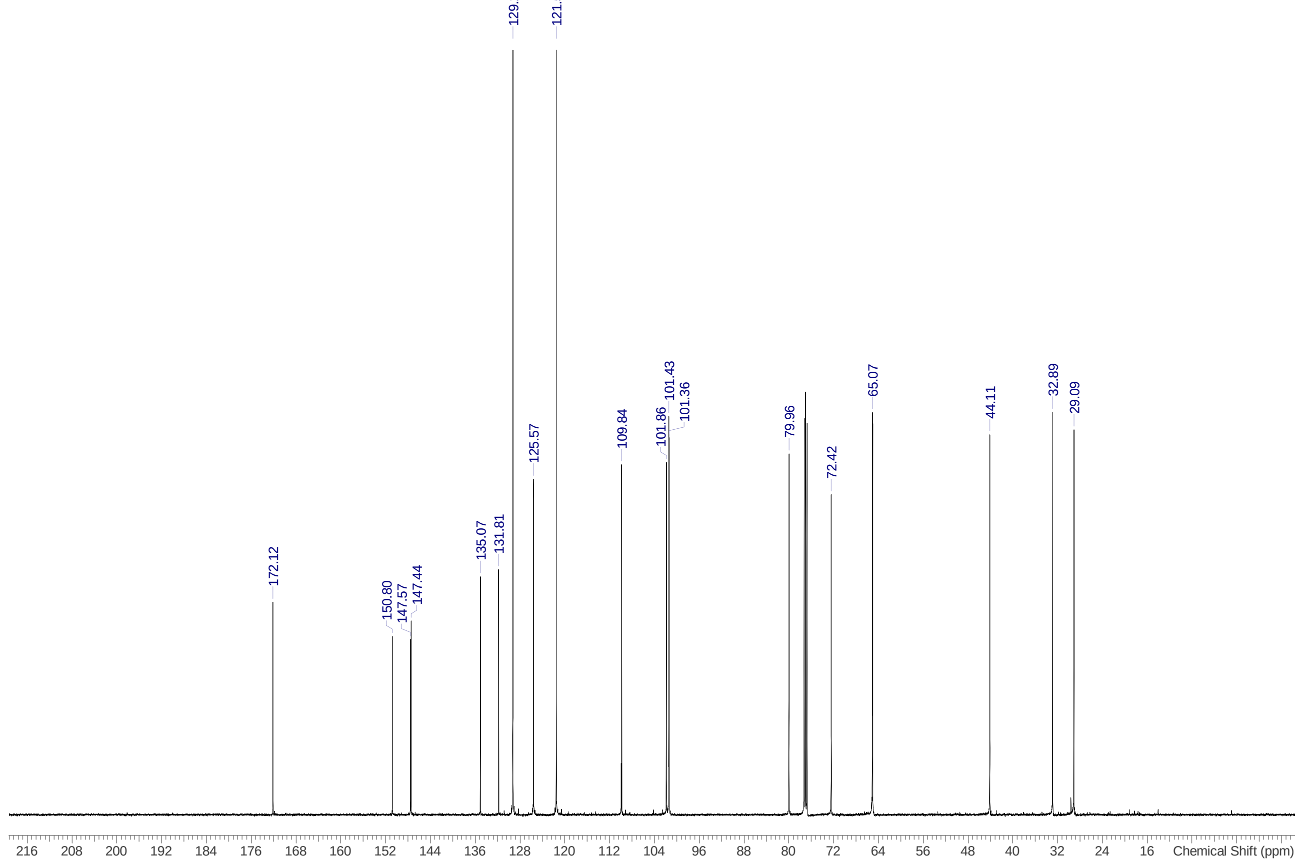

^{1}H NMR spectrum of phthalan **11a** (500 MHz, $CDCl_3$)

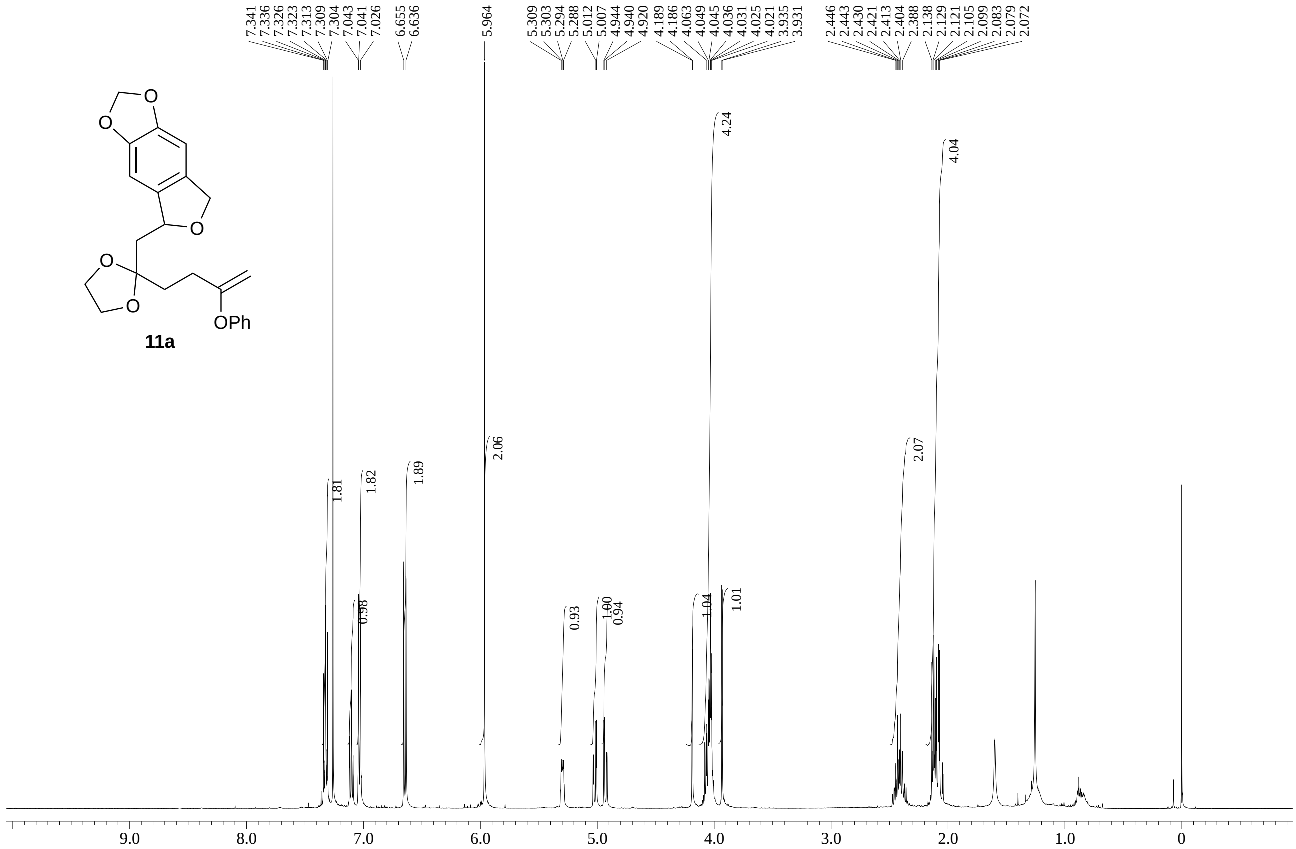

^{13}C NMR spectrum of phthalan **11a** (126 MHz, $CDCl_3$)

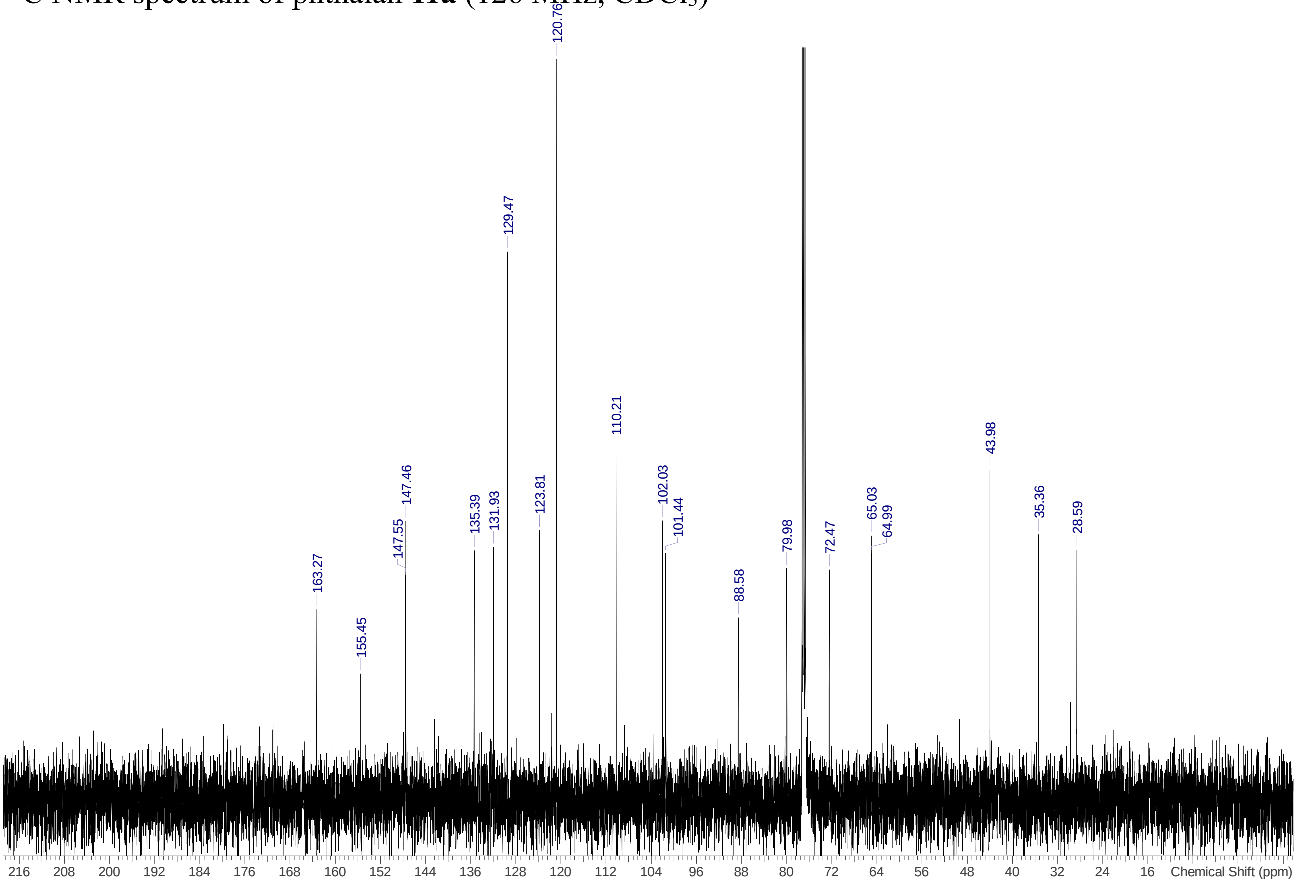

^{1}H NMR spectrum of diester **S29** (500 MHz, $CDCl_3$)

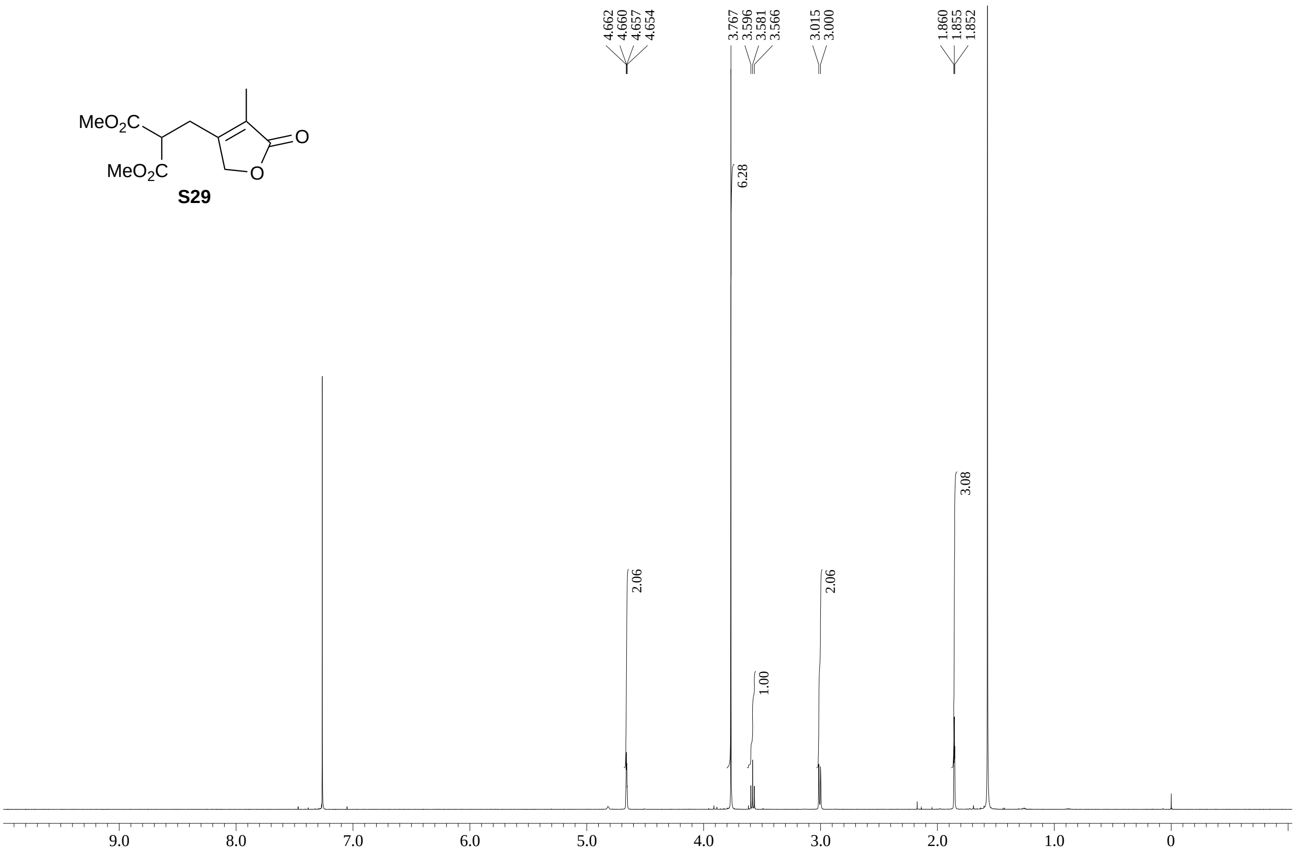

^{13}C NMR spectrum of diester **S29** (126 MHz, $CDCl_3$)

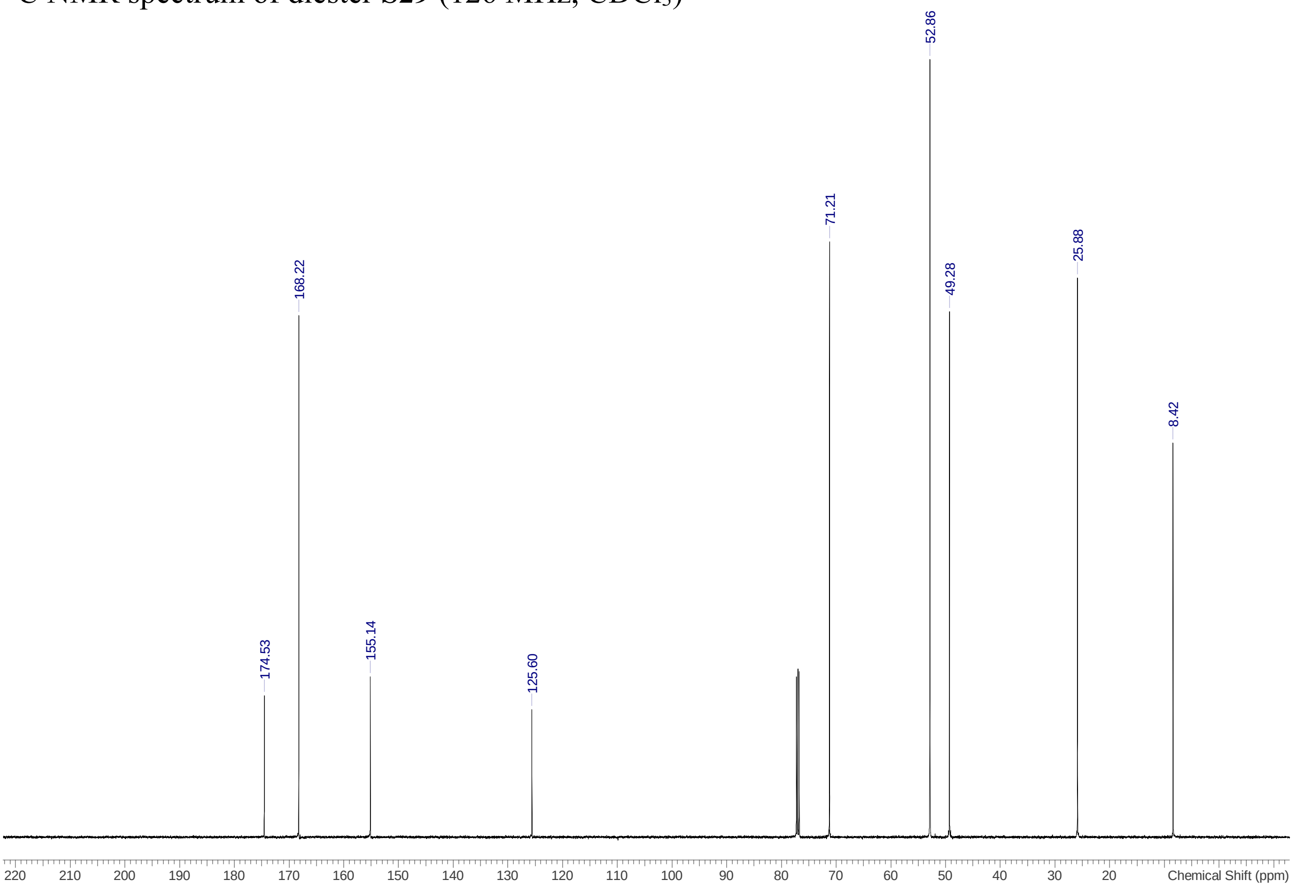

^{1}H NMR spectrum of pentafluorophenyl ester **S30** (500 MHz, $CDCl_3$)

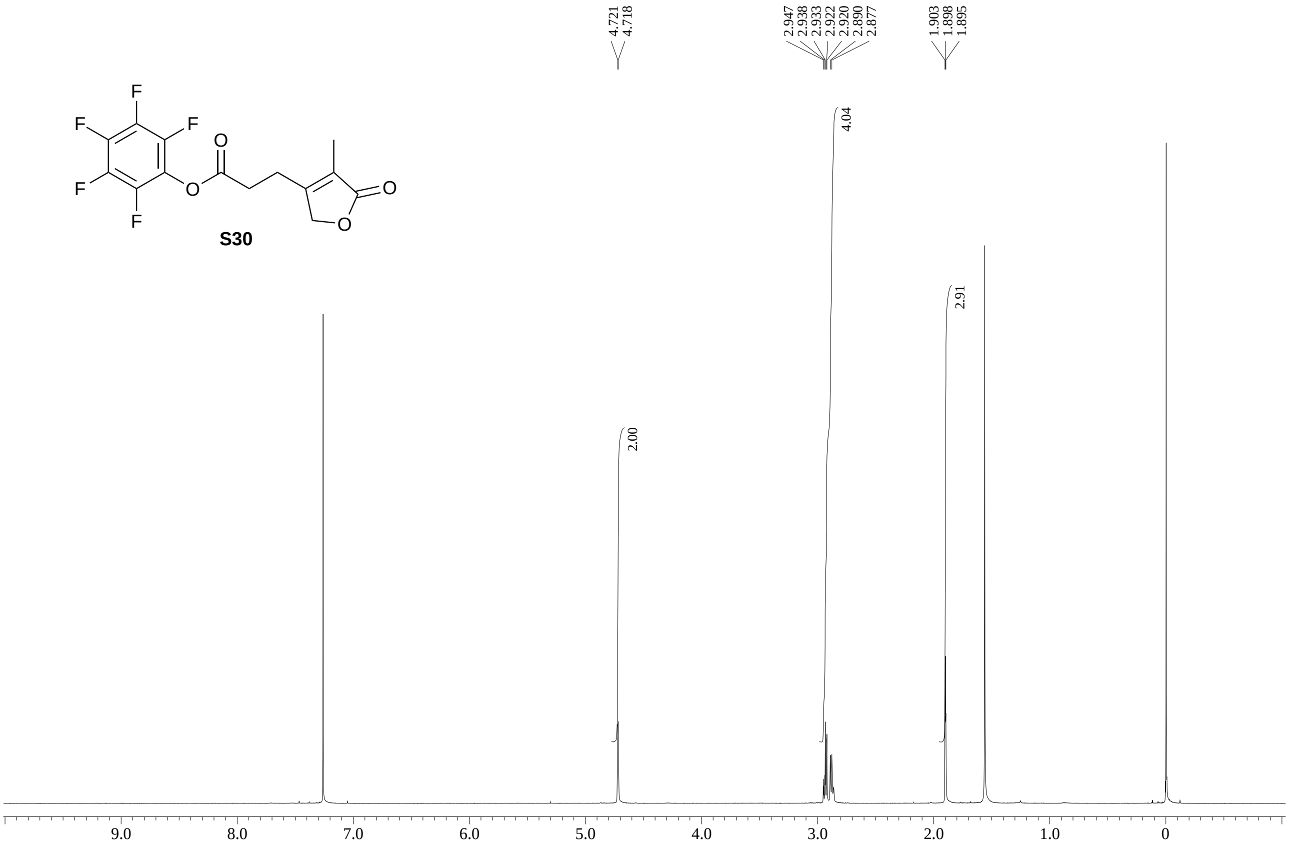

X : parts per Million : Proton

^{13}C NMR spectrum of pentafluorophenyl ester **S30** (126 MHz, $CDCl_3$)

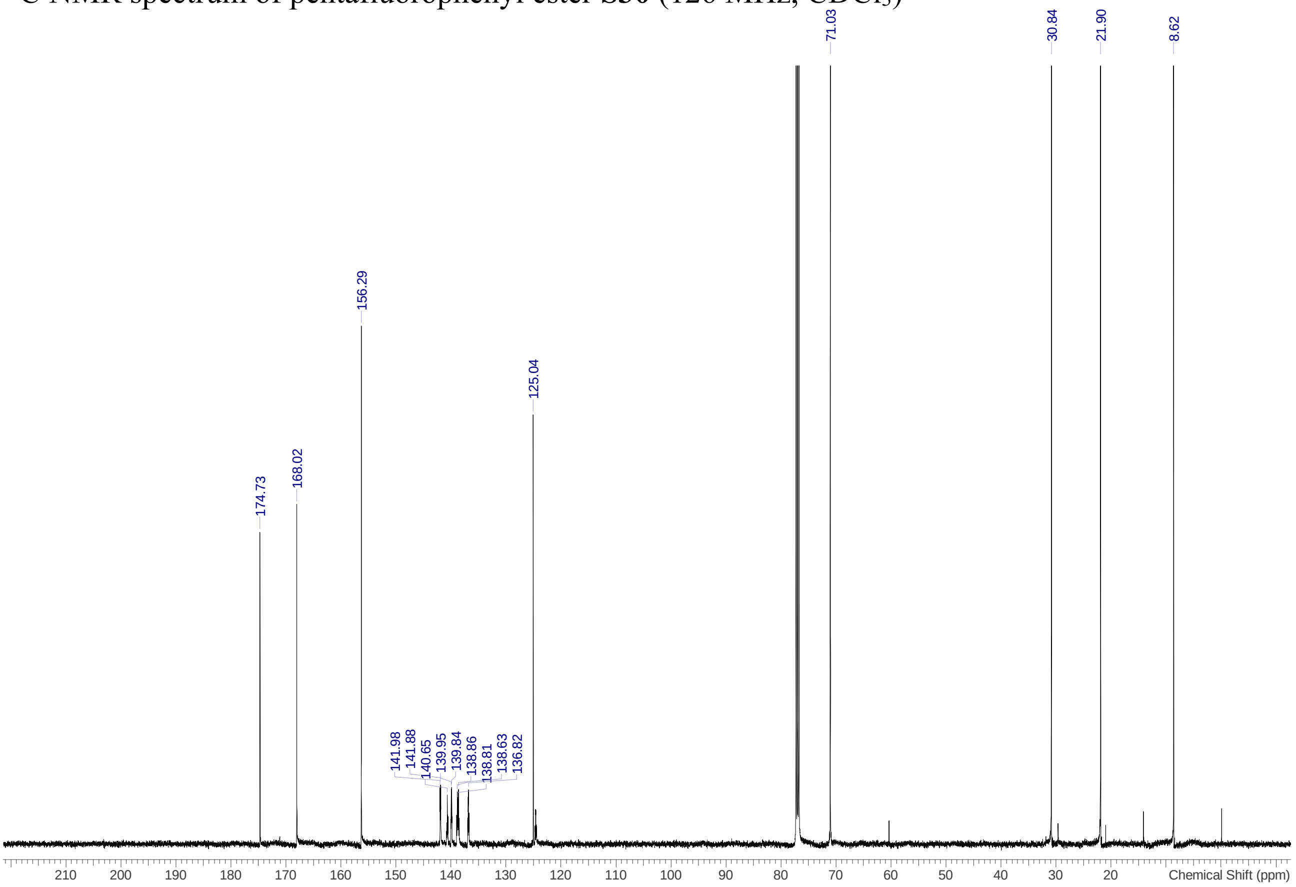

^{1}H NMR spectrum of phosphonate **S31** (500 MHz, $CDCl_3$)

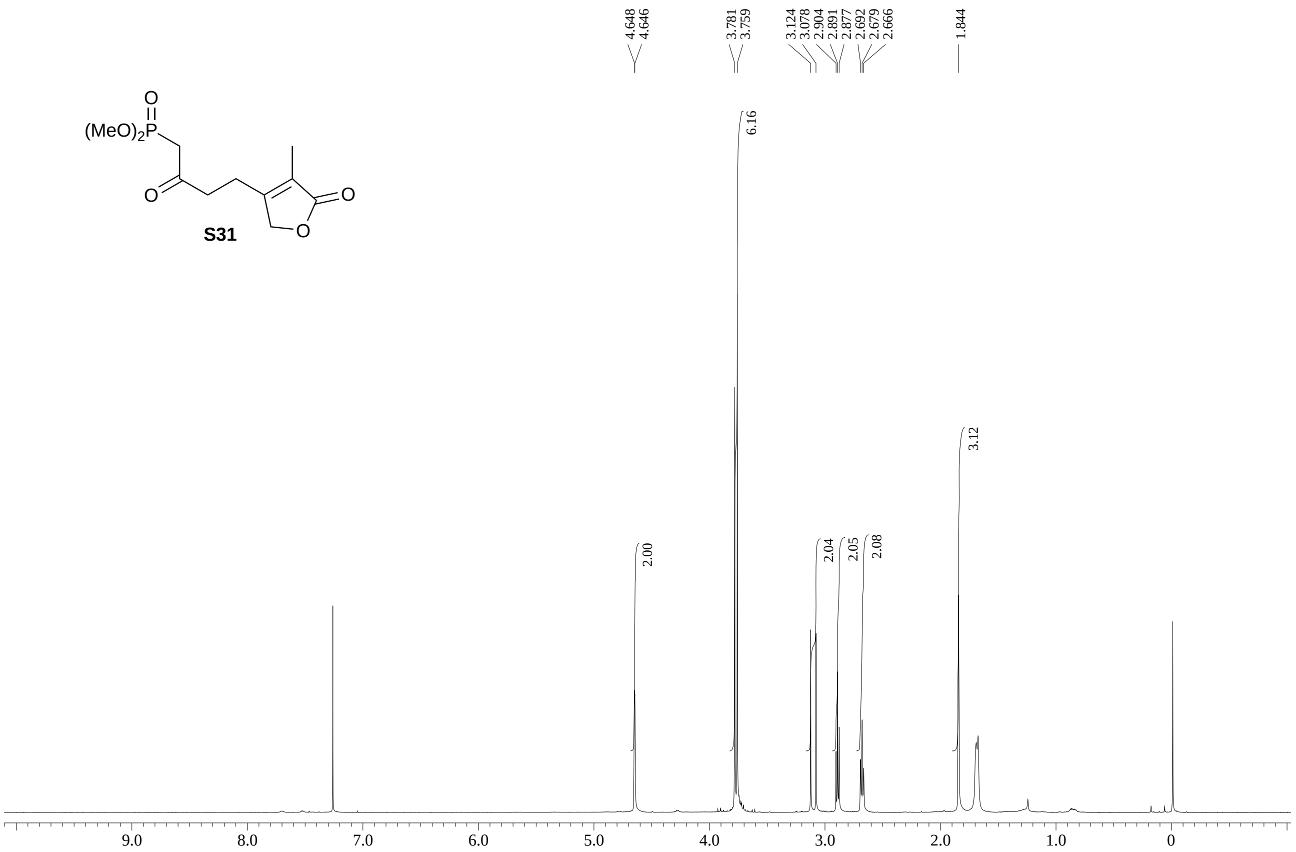

^{13}C NMR spectrum of phosphonate **S31** (126 MHz, $CDCl_3$)

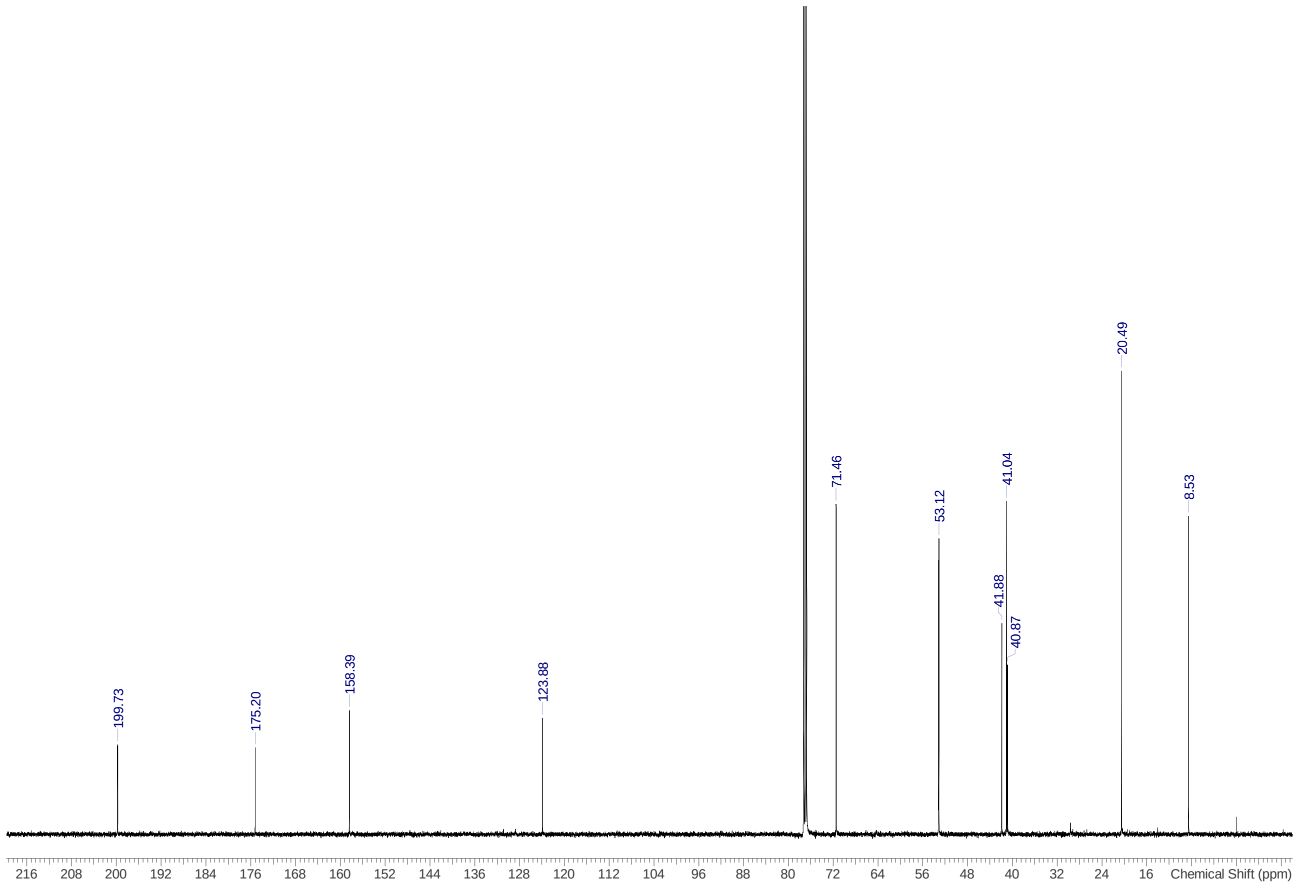

^{1}H NMR spectrum of enone **S33** (500 MHz, $CDCl_3$)

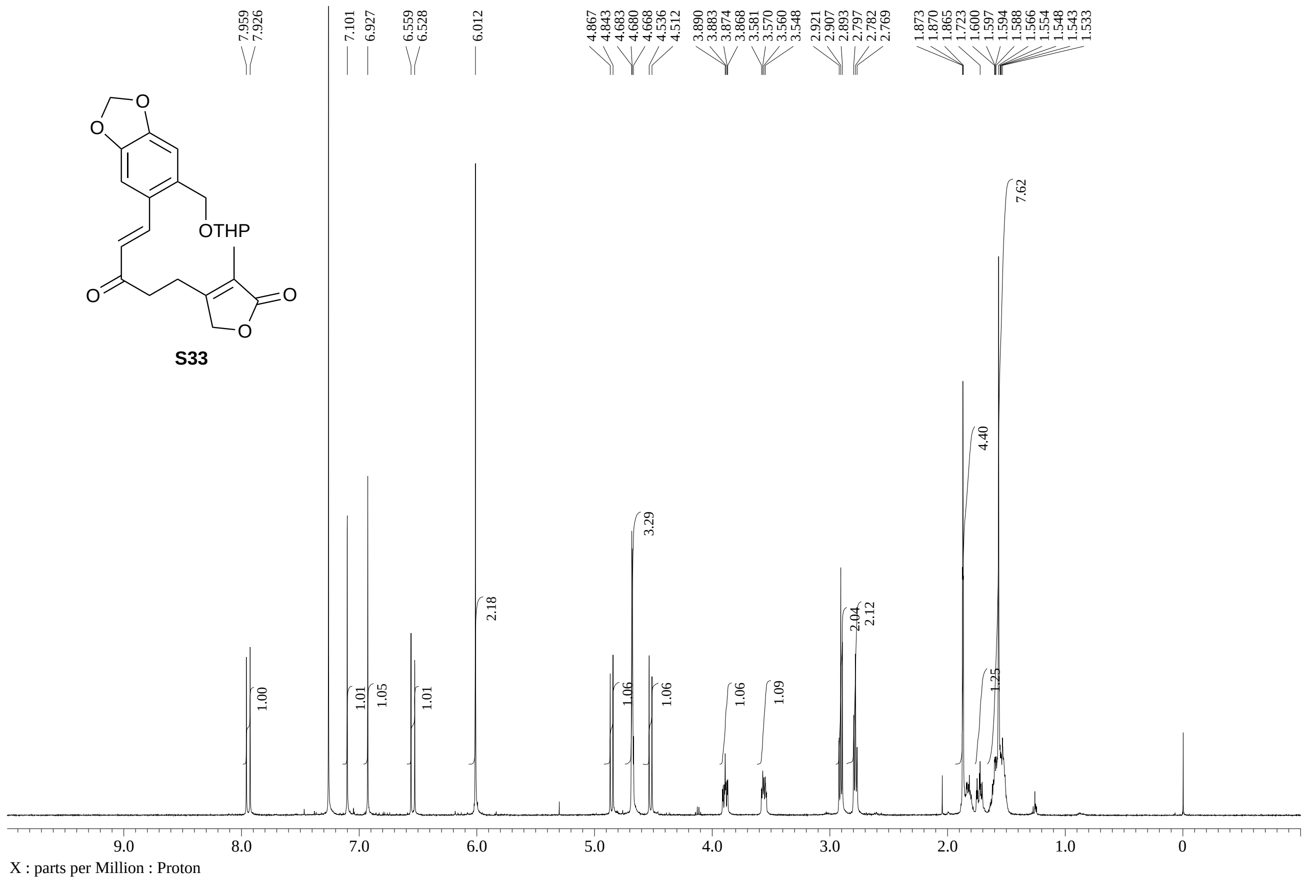

^{13}C NMR spectrum of enone **S33** (126 MHz, $CDCl_3$)

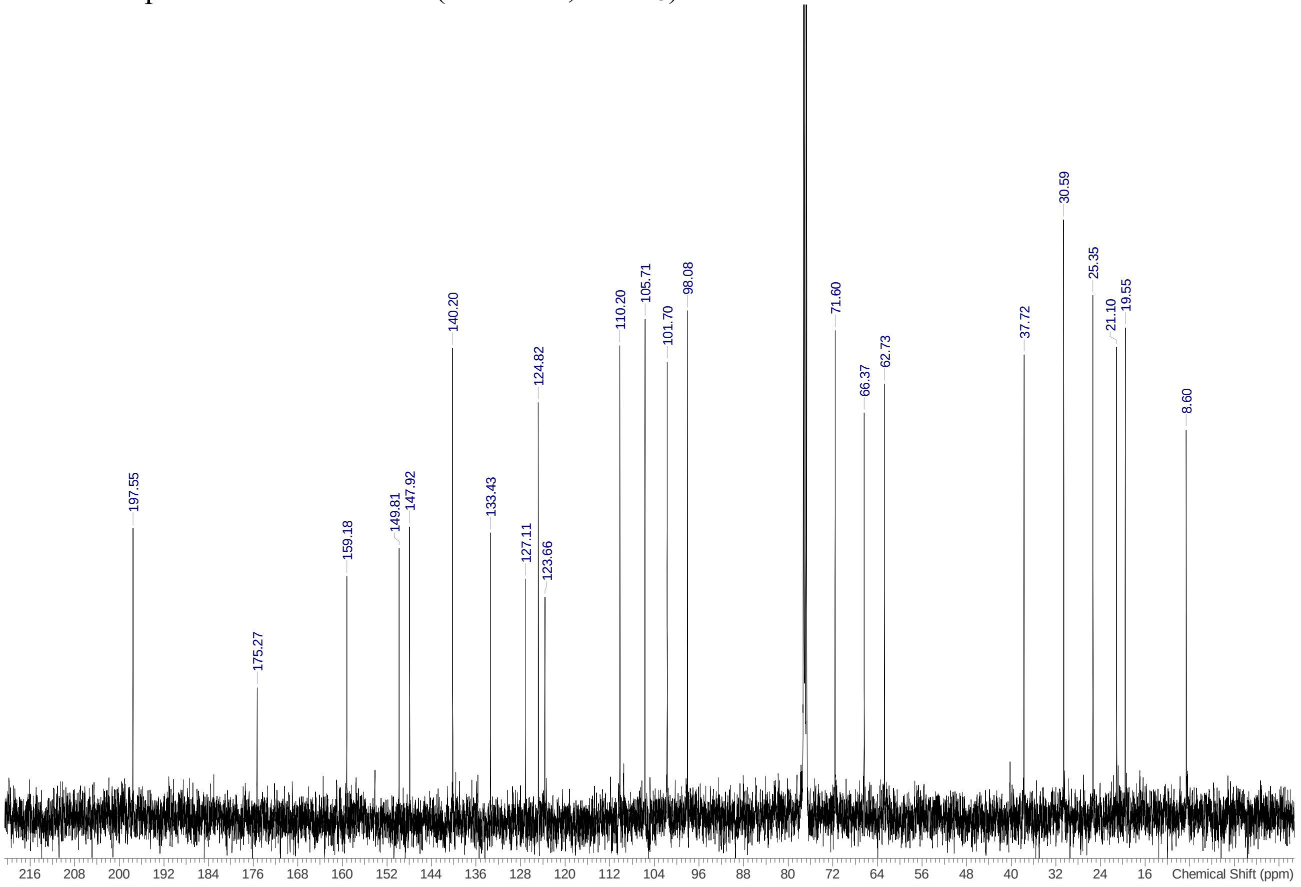

^{1}H NMR spectrum of phthalan **12a** (500 MHz, C_6D_6)

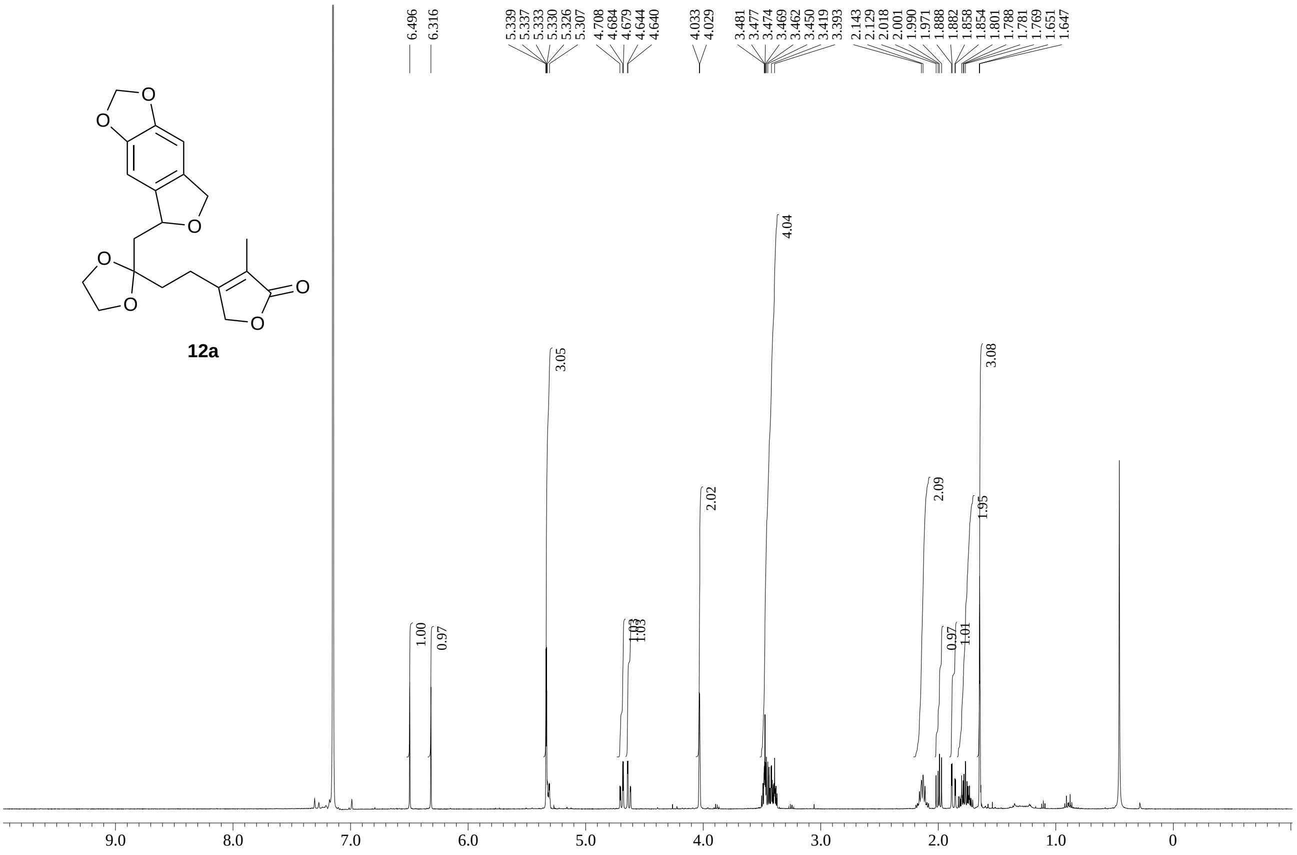

^{13}C NMR spectrum of phthalan **12a** (126 MHz, $CDCl_3$)

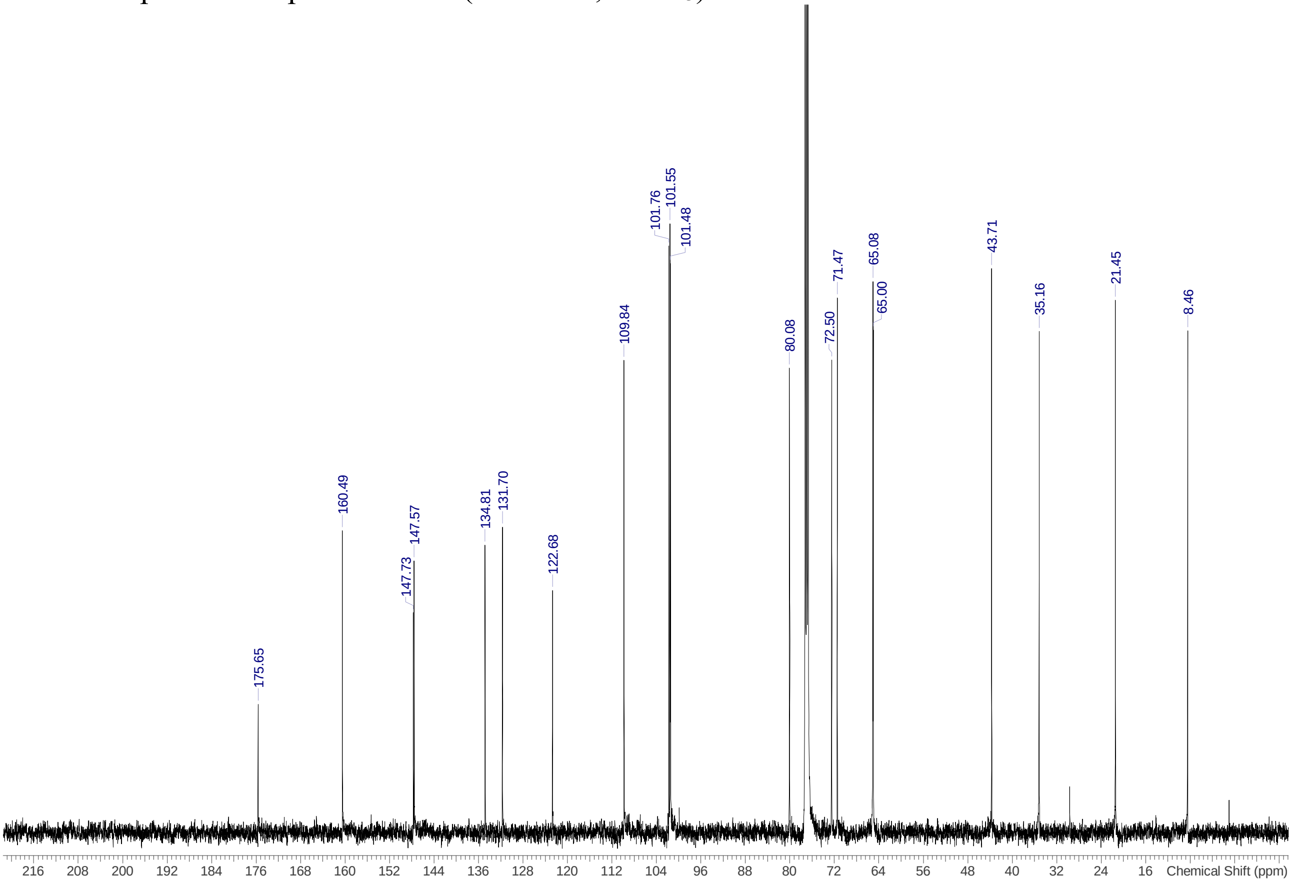

^{1}H NMR spectrum of alkene **S35** (500 MHz, $CDCl_3$)

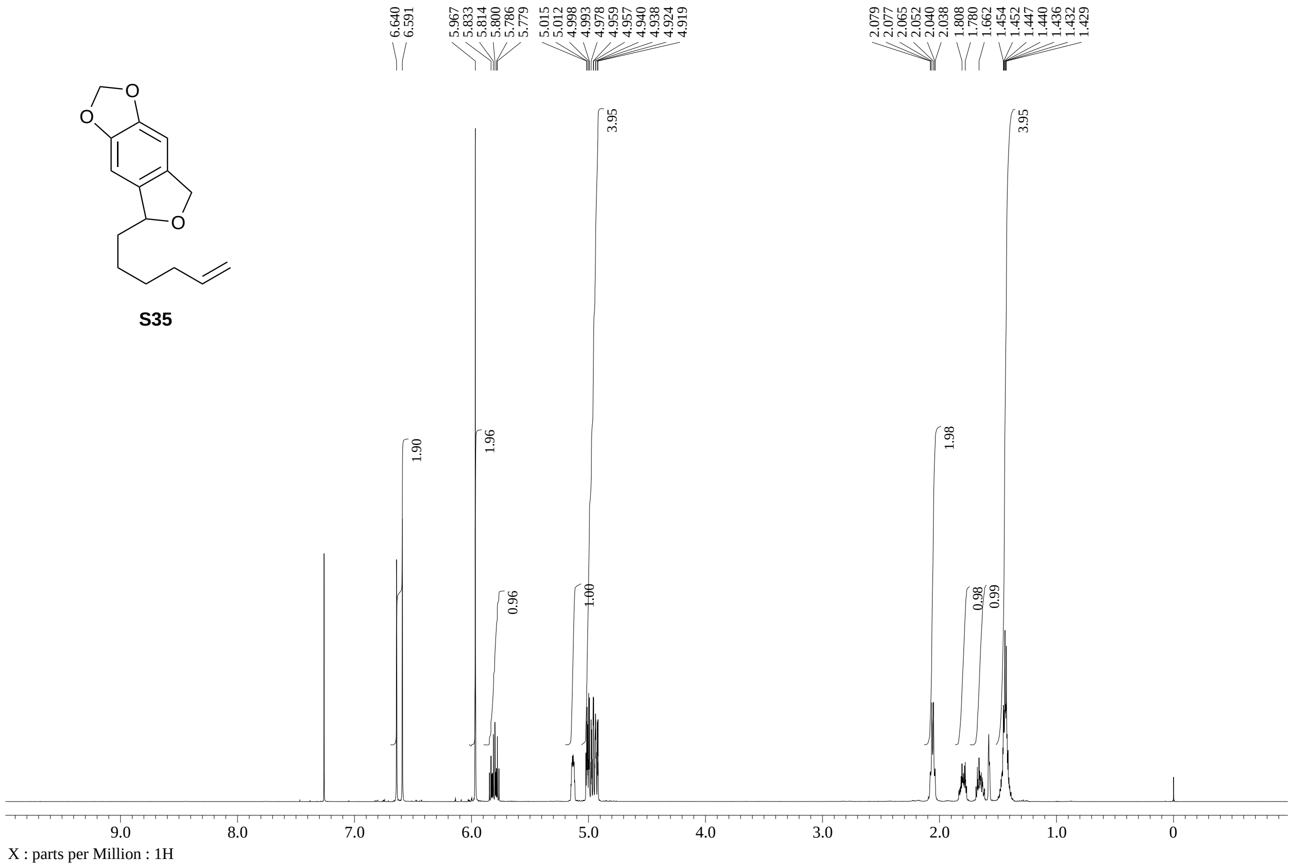
S35

^{13}C NMR spectrum of alkene **S35** (126 MHz, $CDCl_3$)

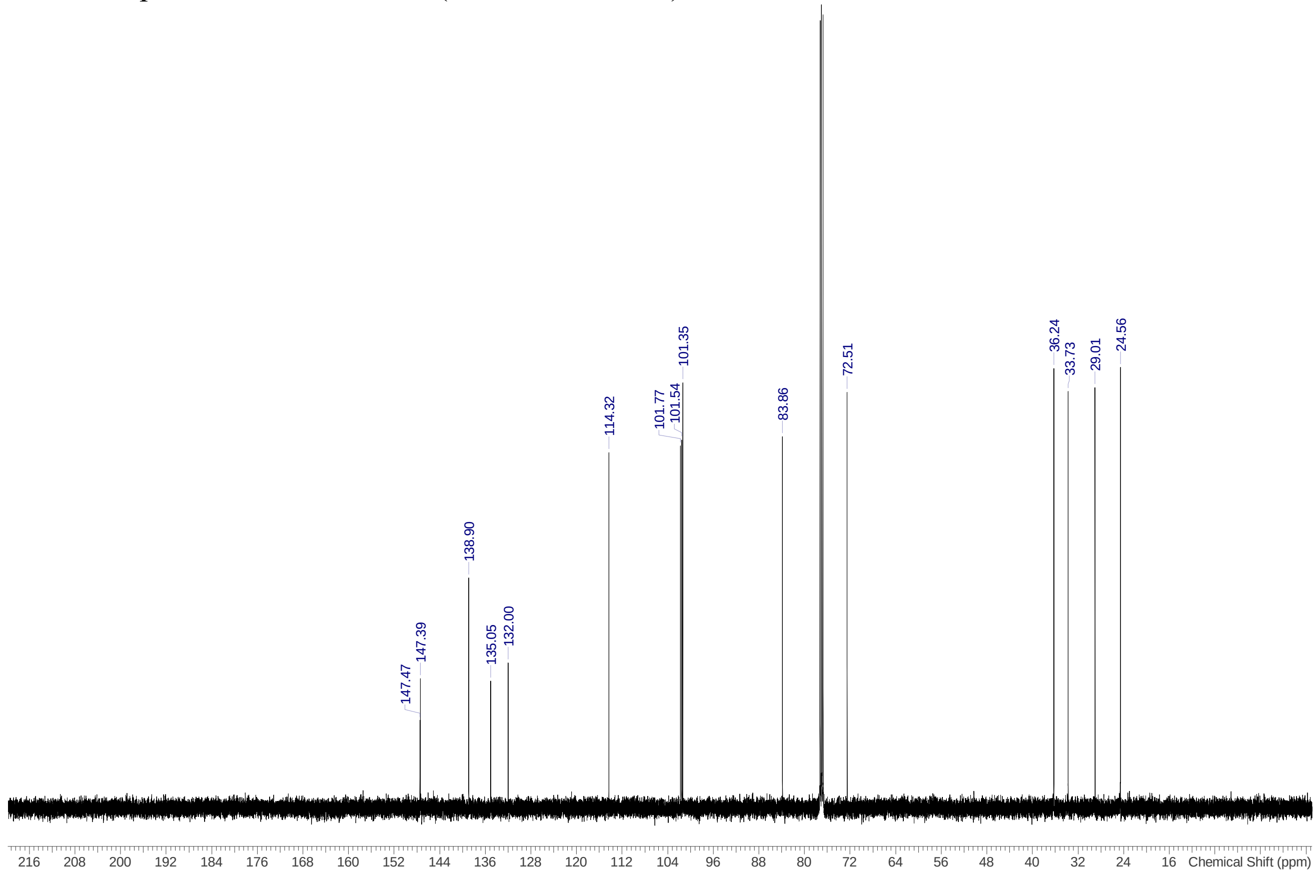

^{1}H NMR spectrum of phthalan **13a** (500 MHz, $CDCl_3$)

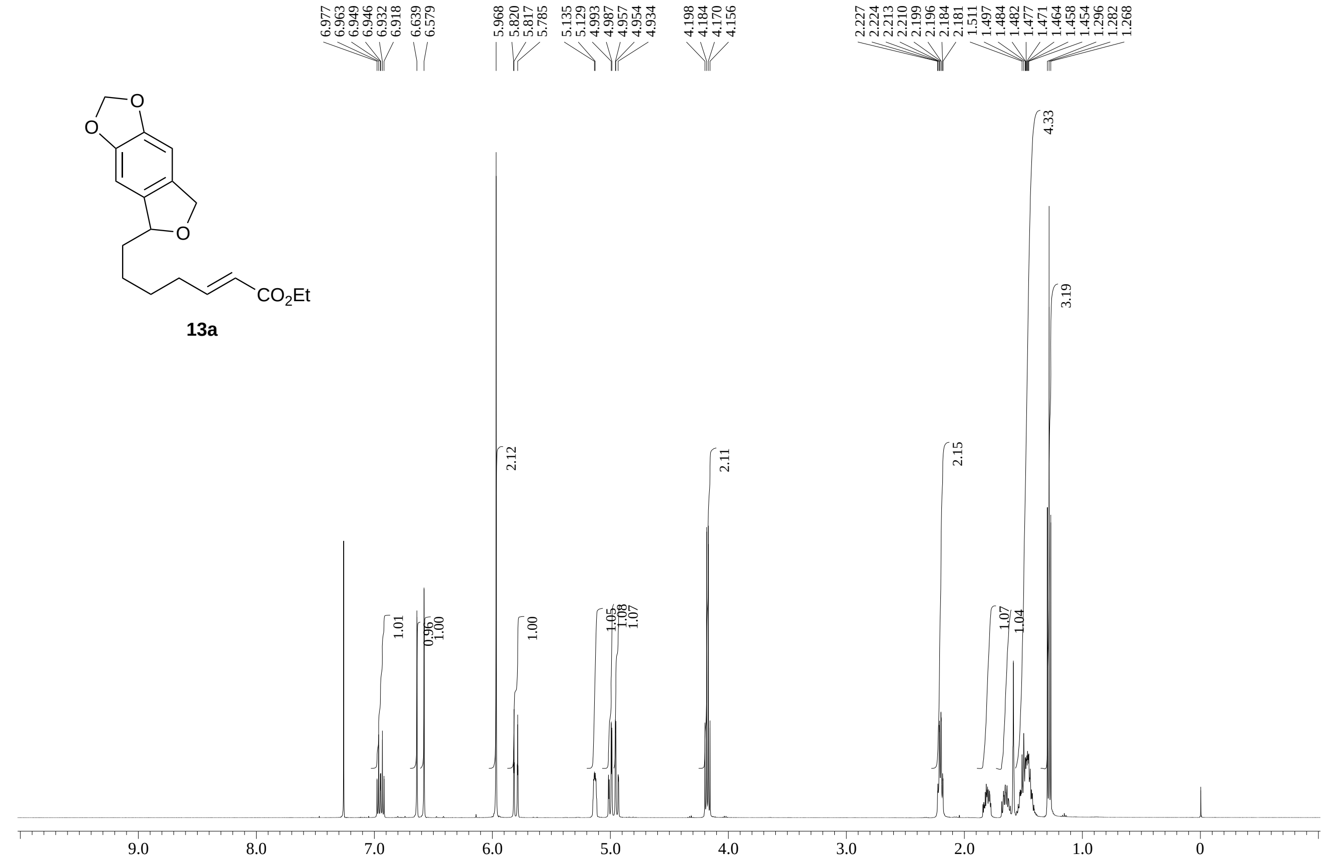

^{13}C NMR spectrum of phthalan **13a** (126 MHz, $CDCl_3$)

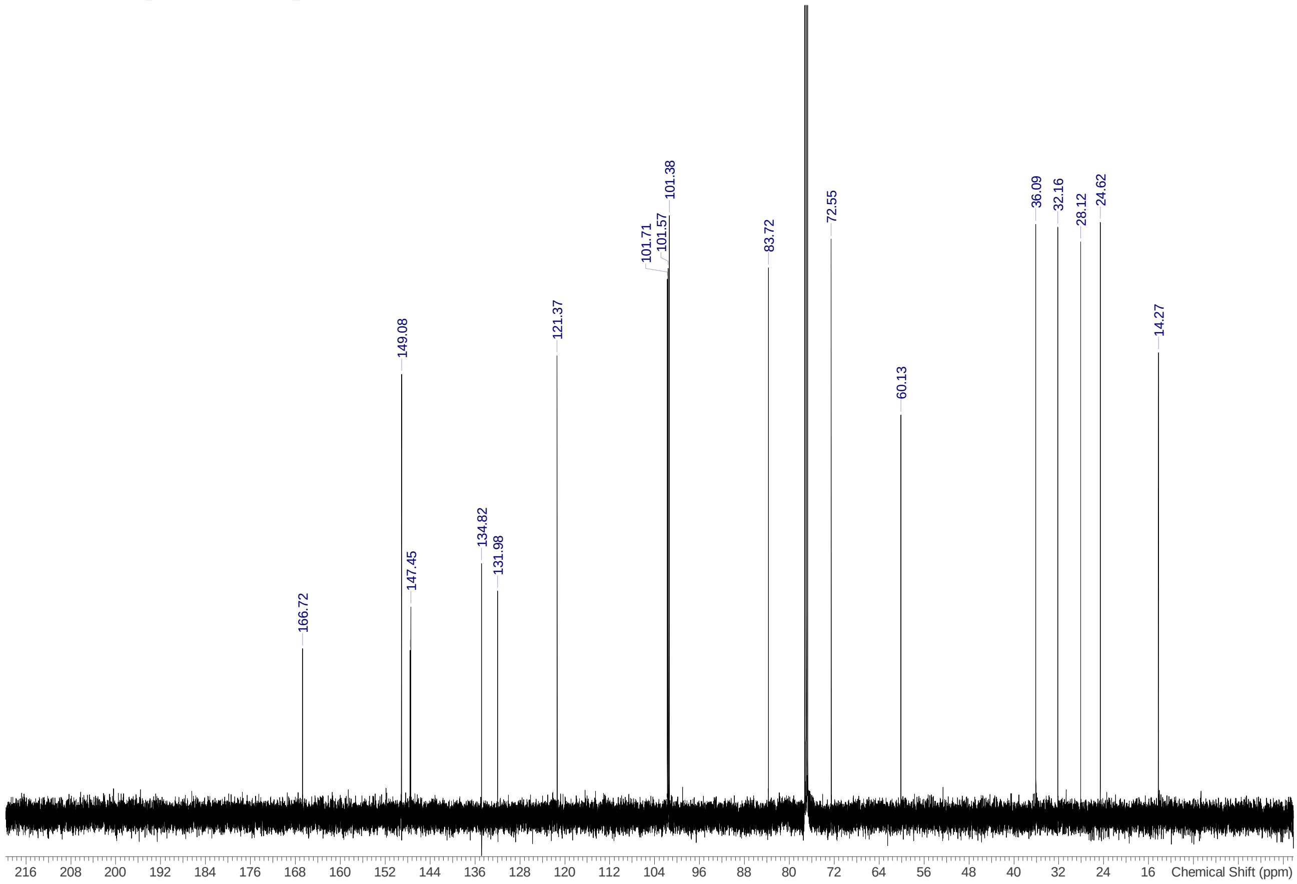

^{1}H NMR spectrum of alkyne **S36** (500 MHz, $CDCl_3$)

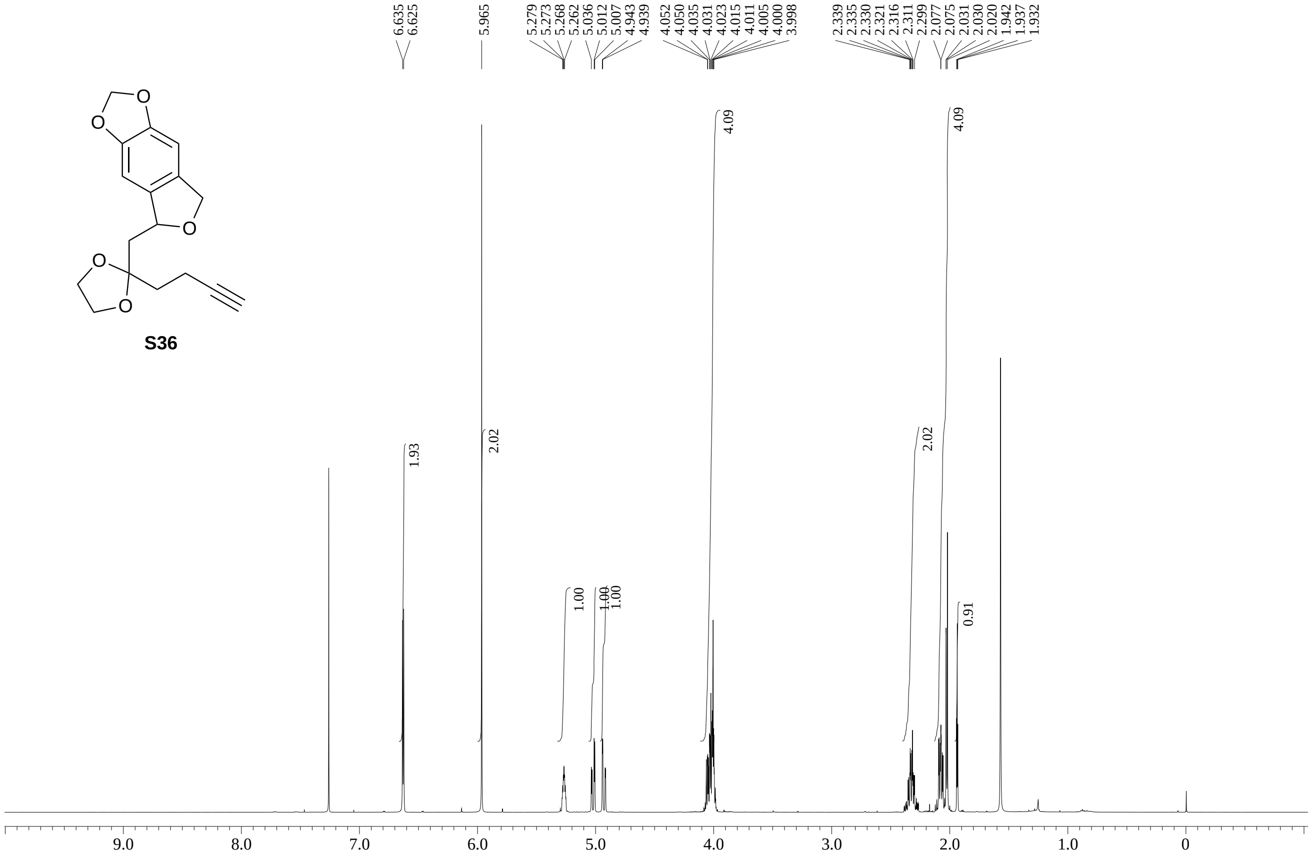

S36

^{13}C NMR spectrum of alkyne **S36** (126 MHz, $CDCl_3$)

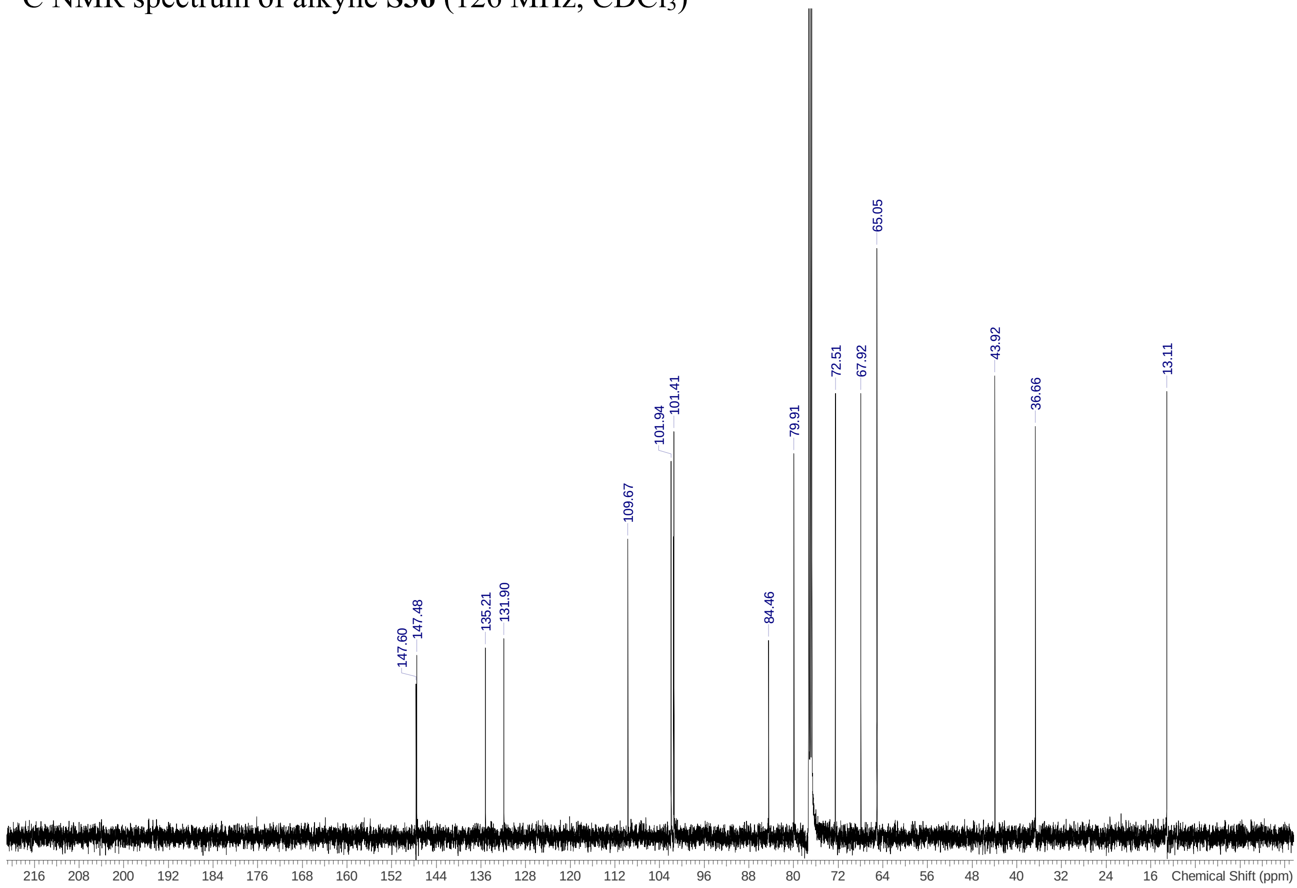

^{1}H NMR spectrum of phthalan **18** (500 MHz, $CDCl_3$)

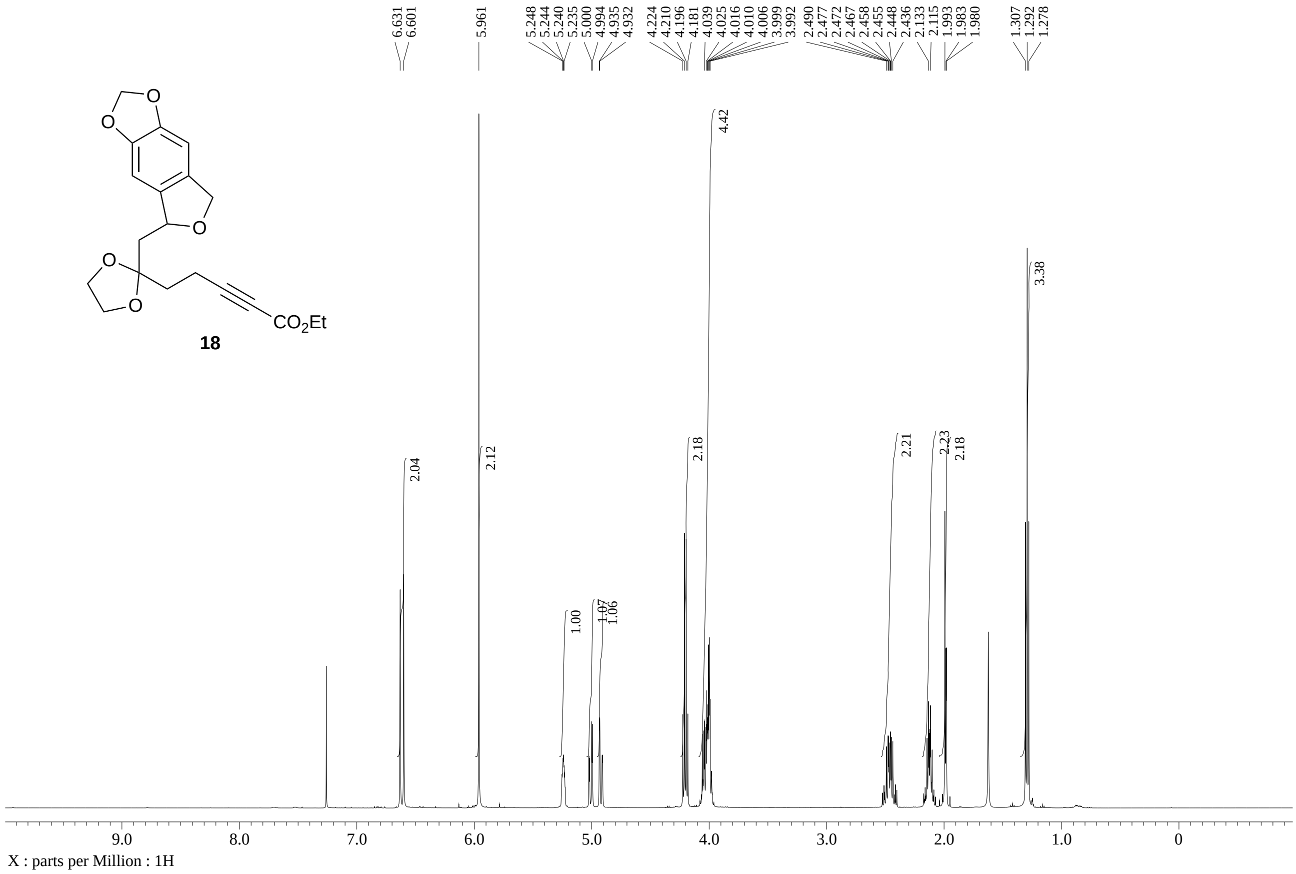

^{13}C NMR spectrum of phthalan **18** (126 MHz, $CDCl_3$)

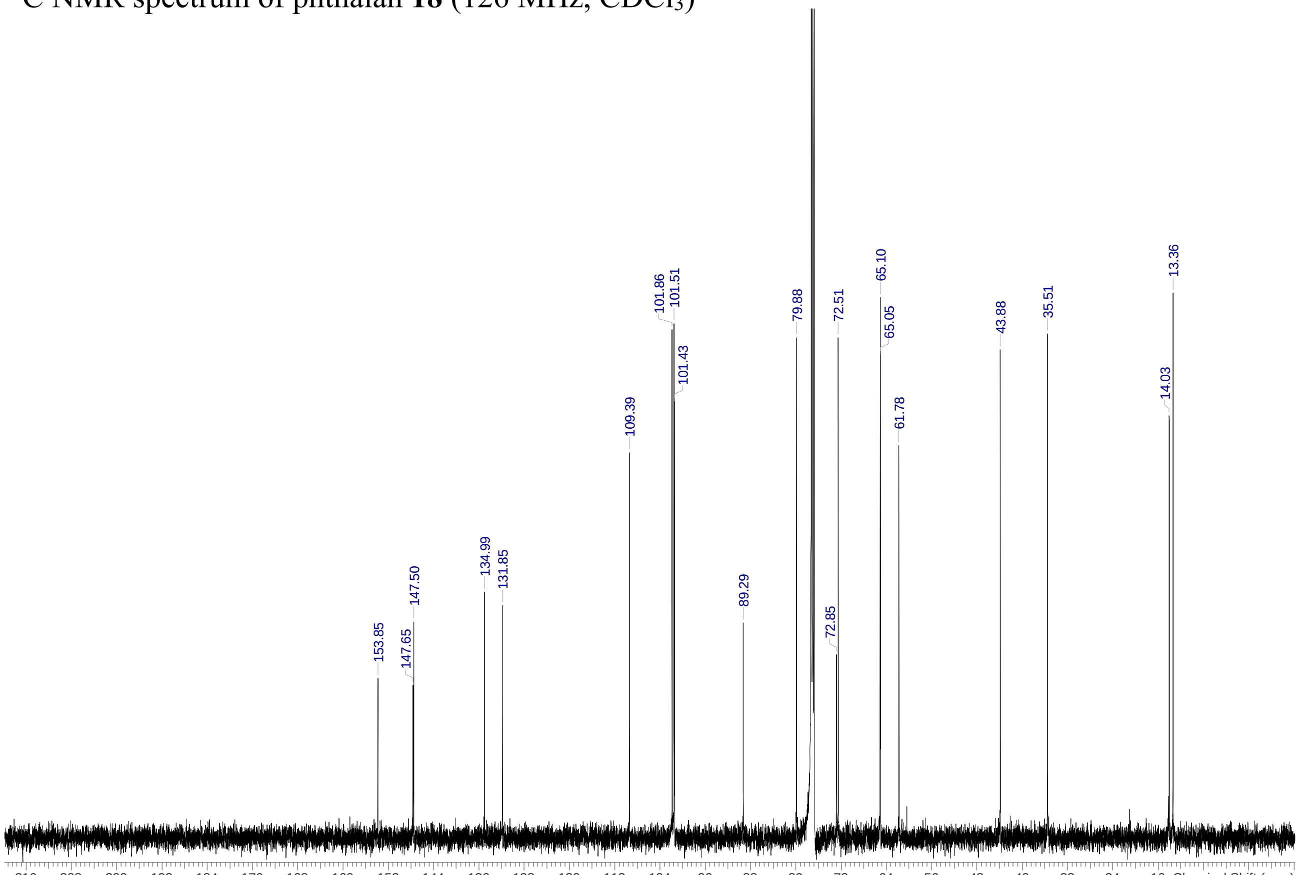

^{1}H NMR spectrum of cycloadduct **3** (500 MHz, C_6D_6)

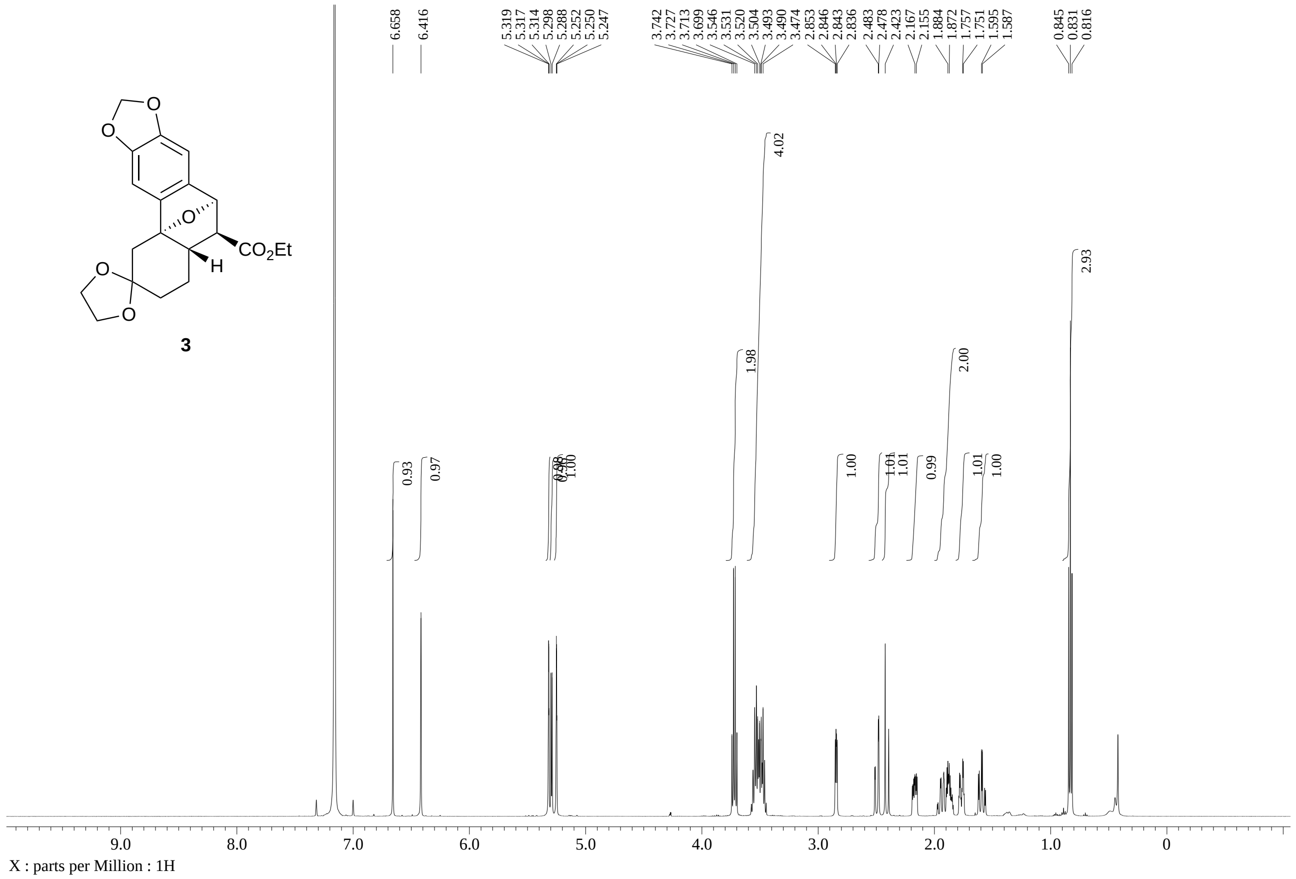

^{13}C NMR spectrum of cycloadduct **3** (126 MHz, C_6D_6)

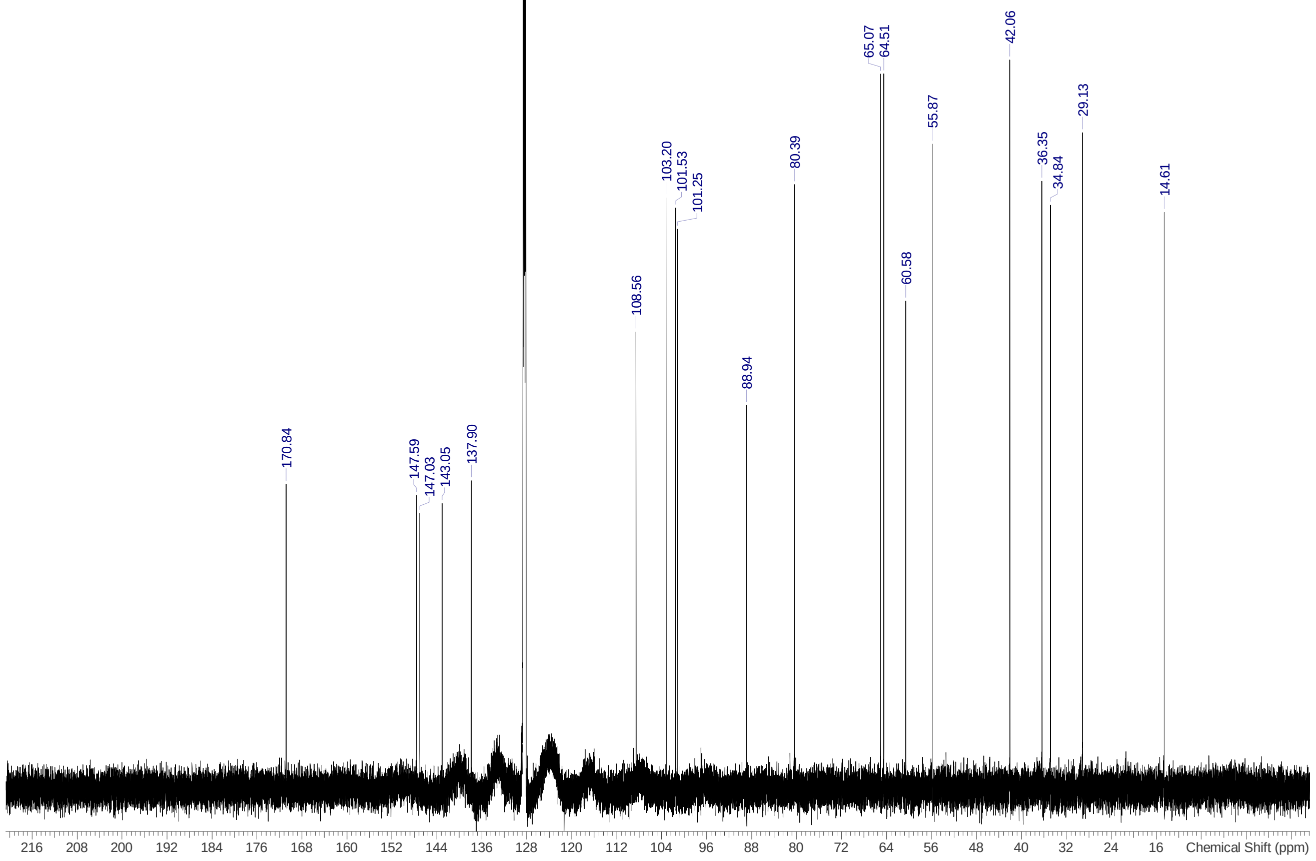

^{1}H NMR spectrum of overoxidation product **4** (500 MHz, $CDCl_3$)

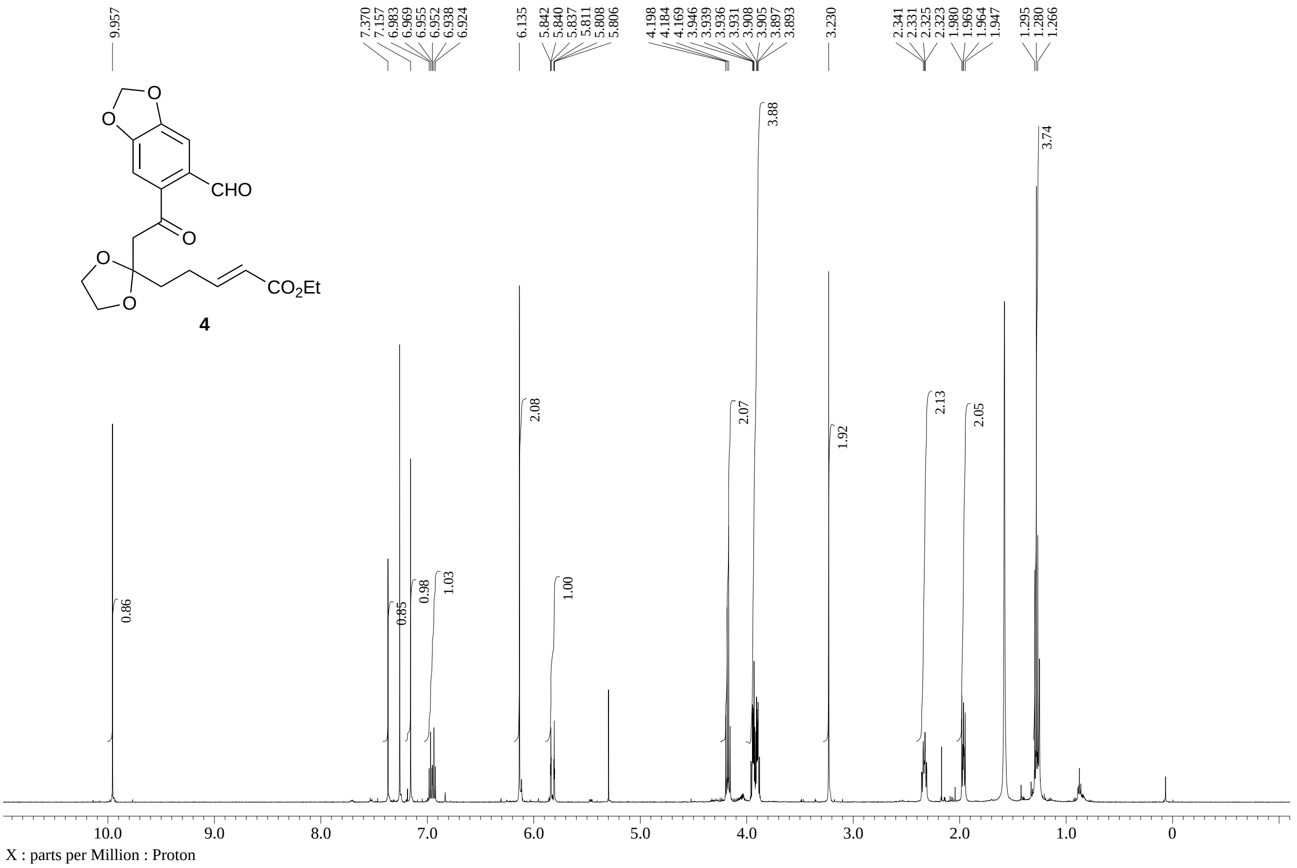

^{13}C NMR spectrum of overoxidation product **4** (126 MHz, $CDCl_3$)

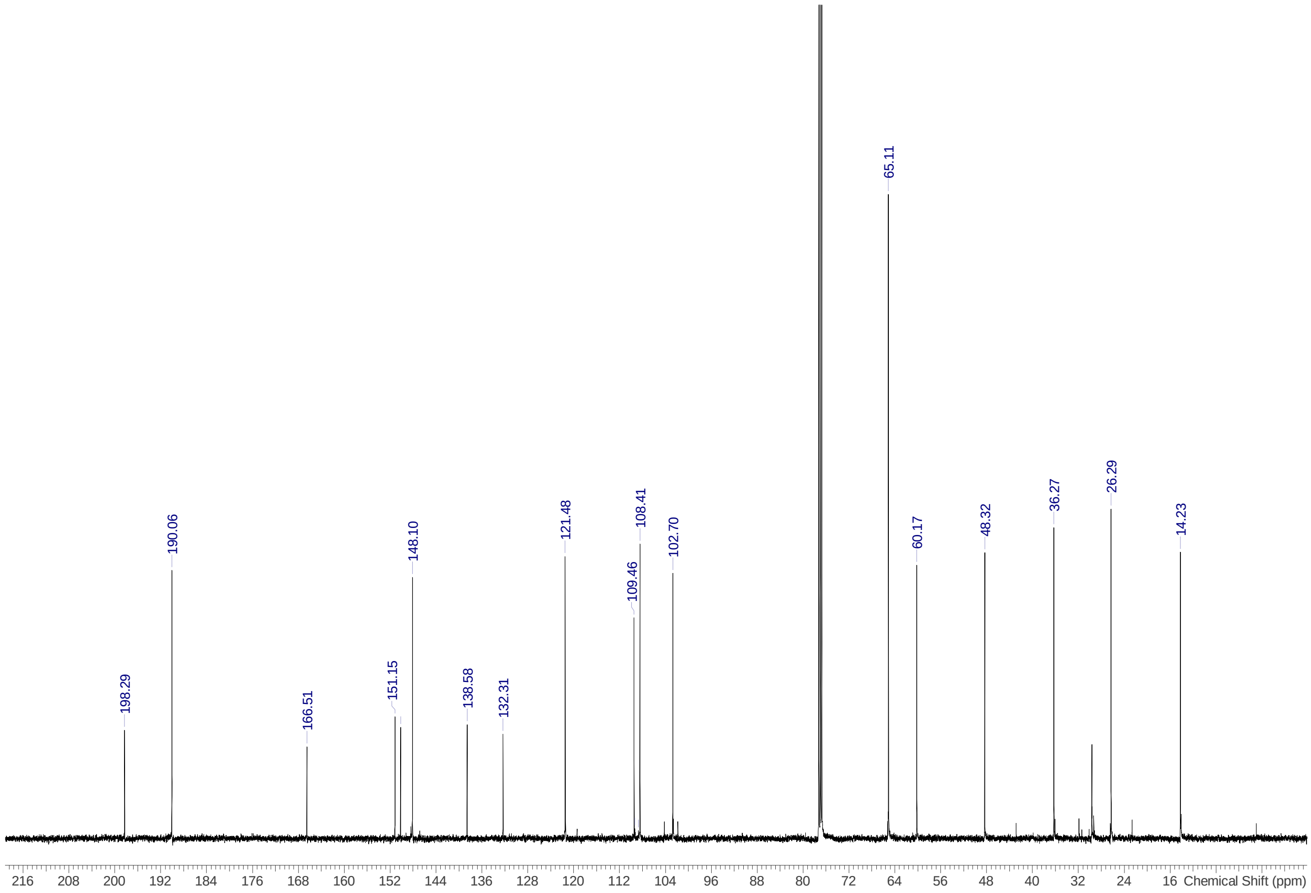

^{1}H NMR spectrum of cycloadduct **5** (500 MHz, $CDCl_3$)

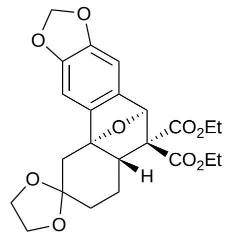

5

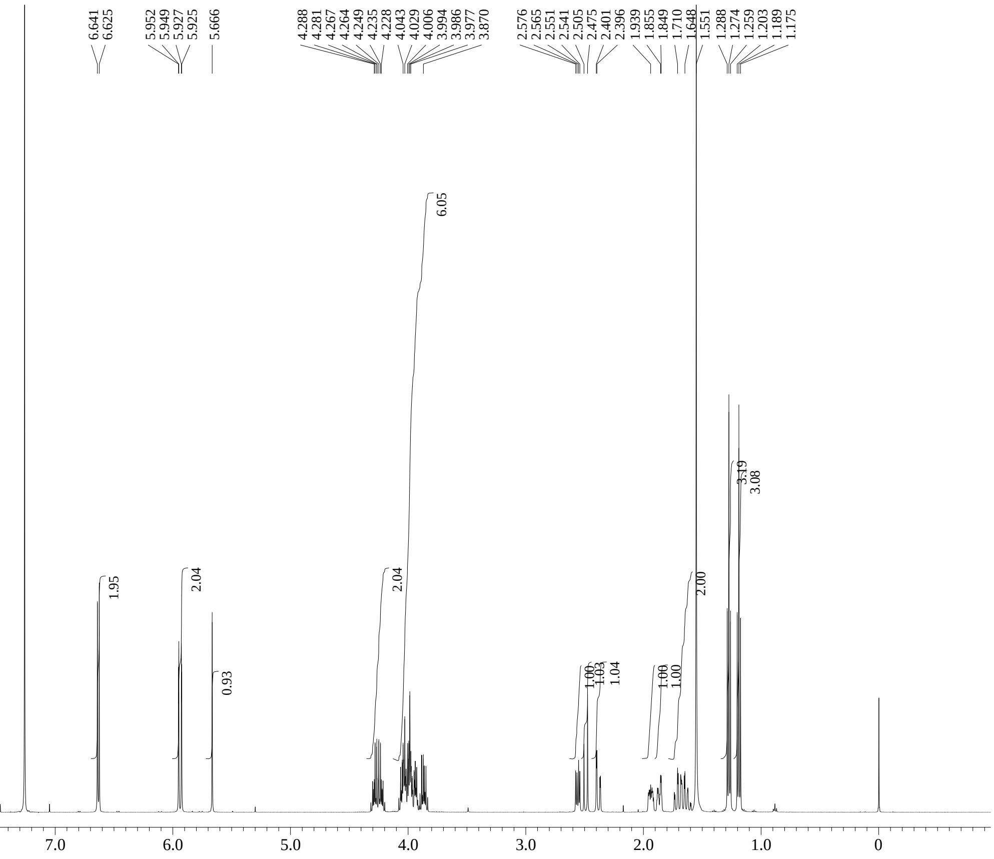

^{13}C NMR spectrum of cycloadduct **5** (126 MHz, $CDCl_3$)

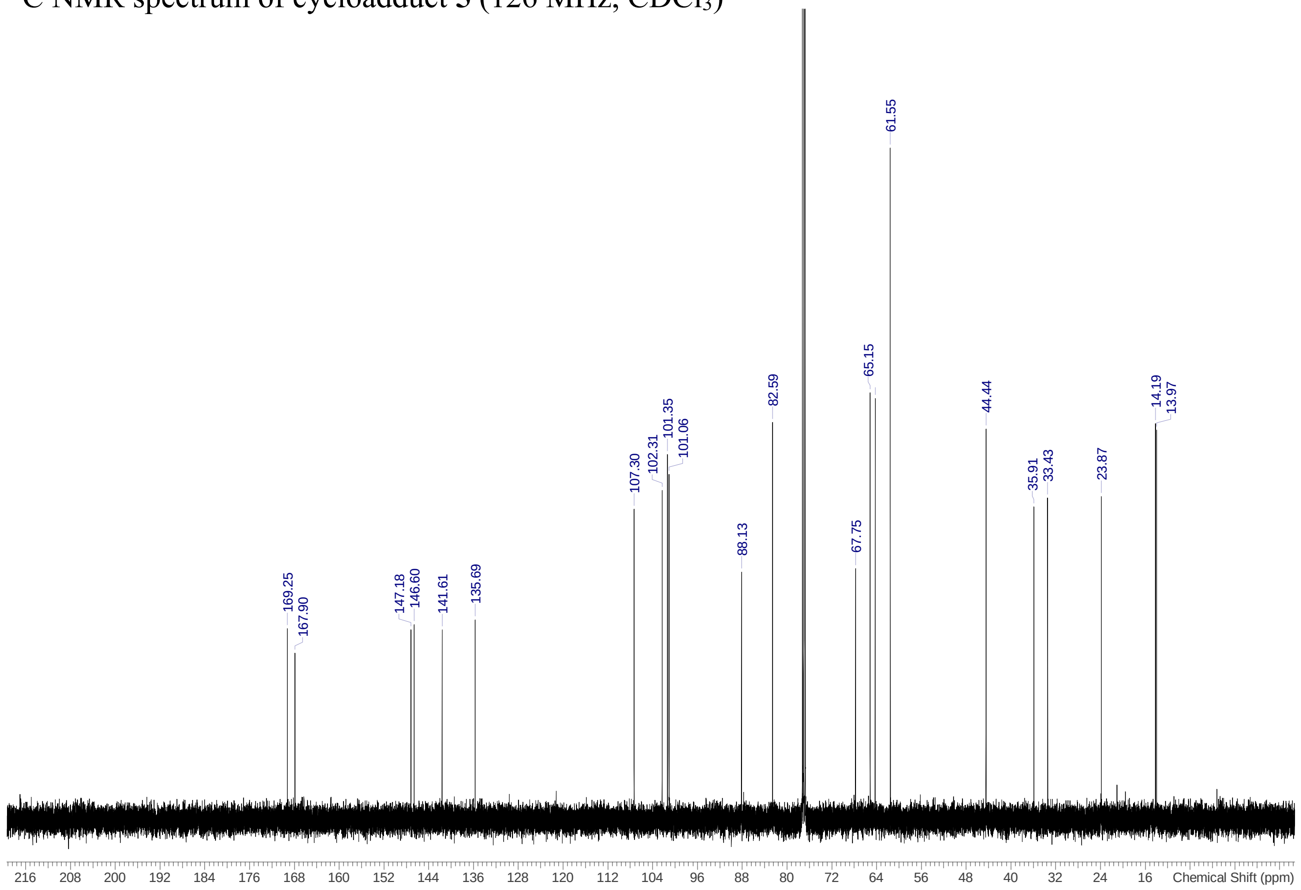

^{1}H NMR spectrum of cycloadduct **6** (500 MHz, $CDCl_3$)

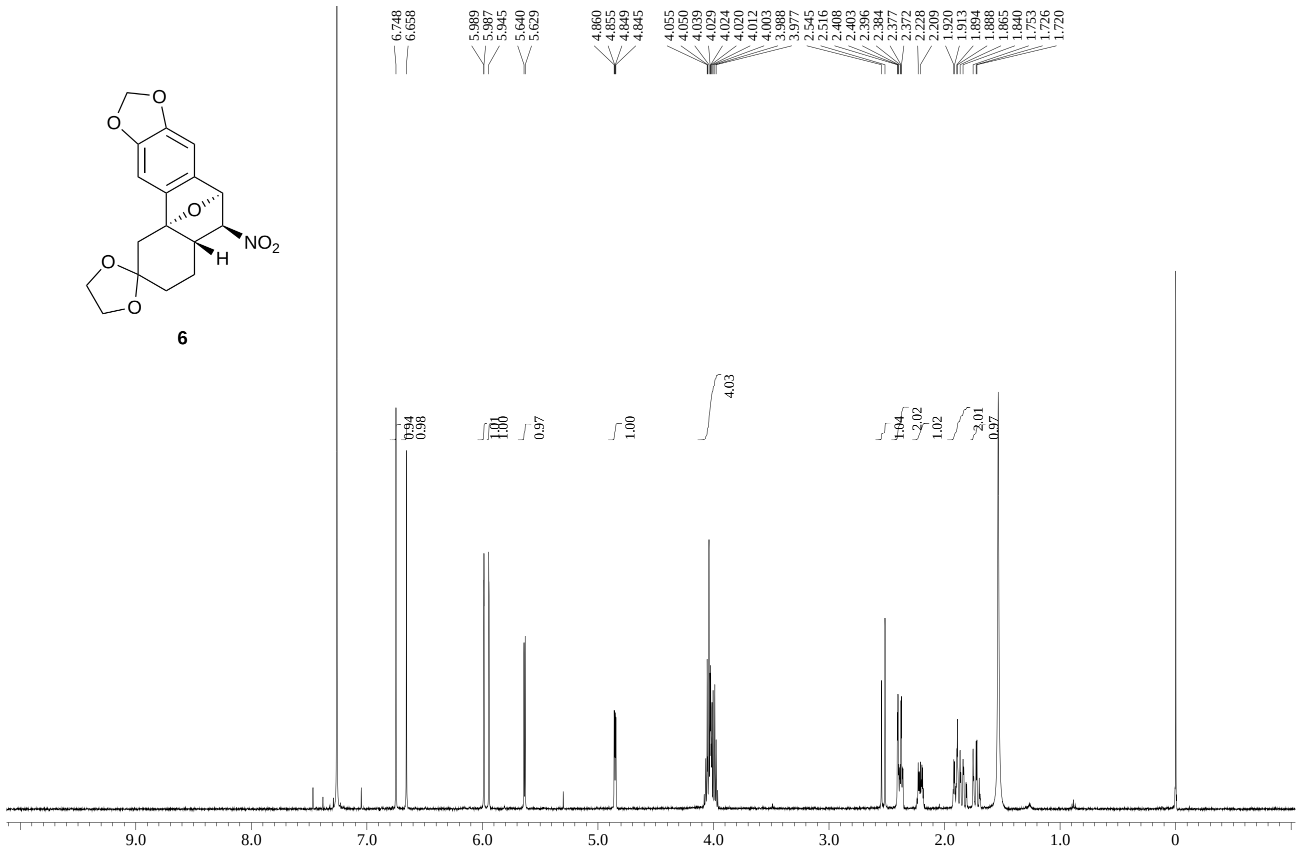

^{13}C NMR spectrum of cycloadduct **6** (126 MHz, $CDCl_3$)

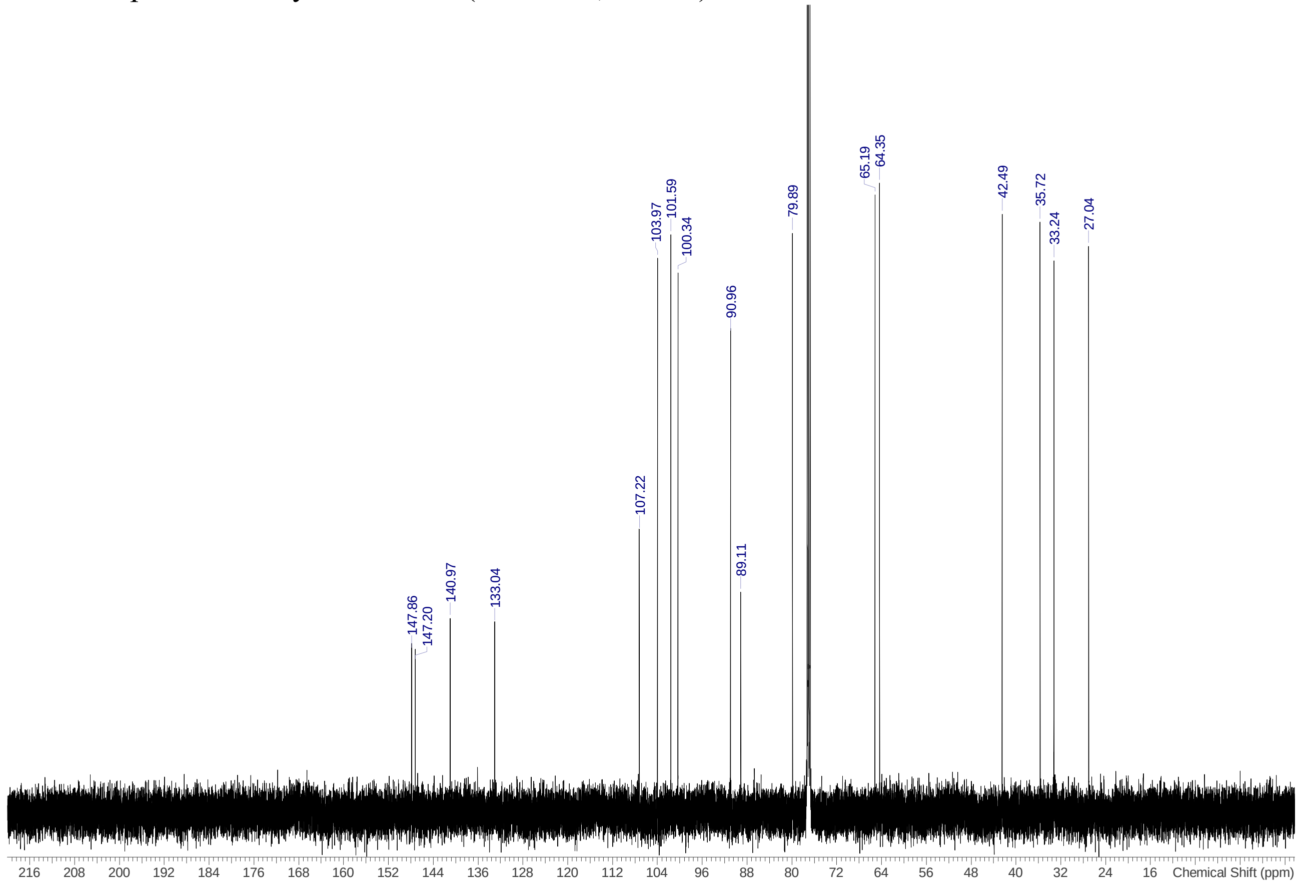

^{1}H NMR spectrum of cycloadduct **7** (500 MHz, $CDCl_3$)

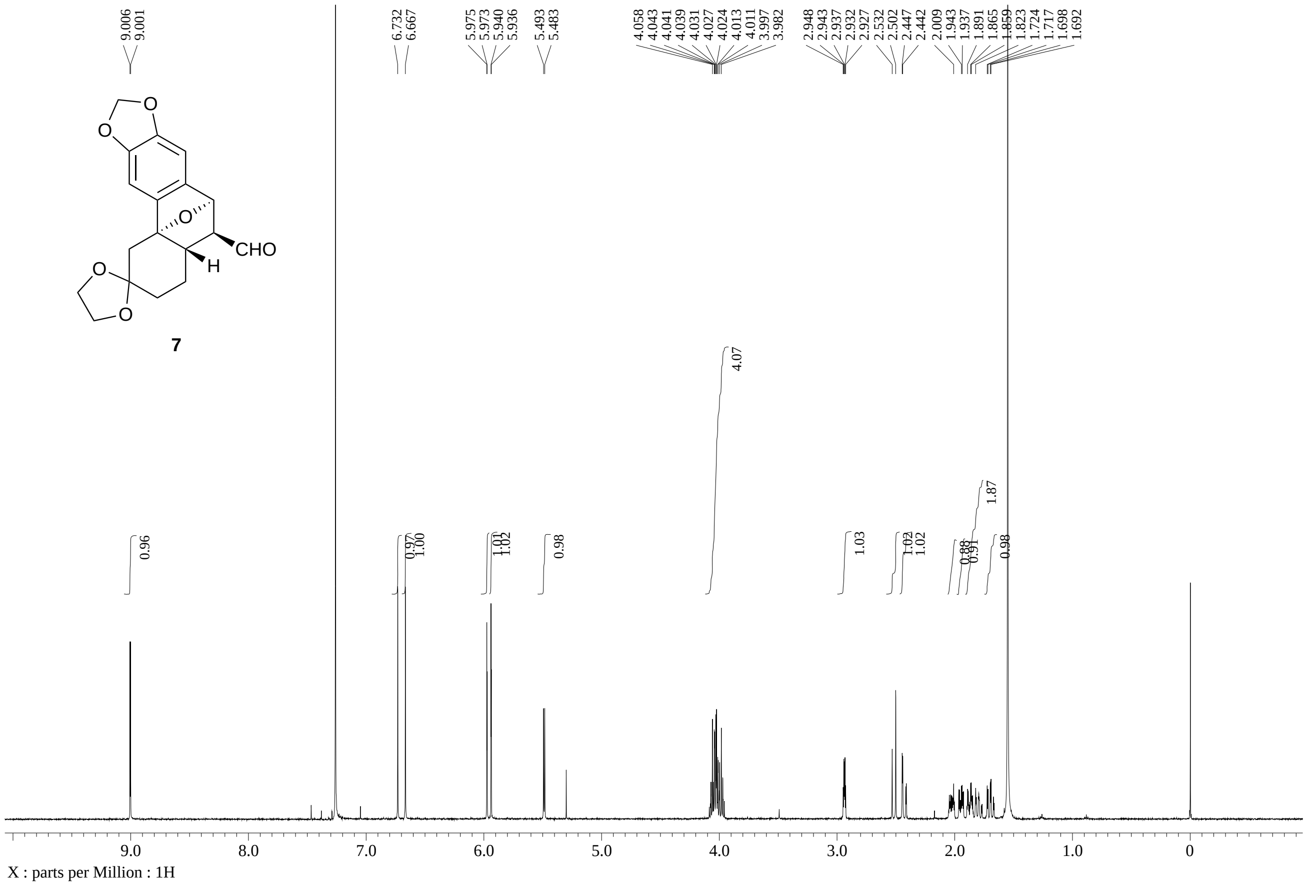

^{13}C NMR spectrum of cycloadduct **7** (126 MHz, $CDCl_3$)

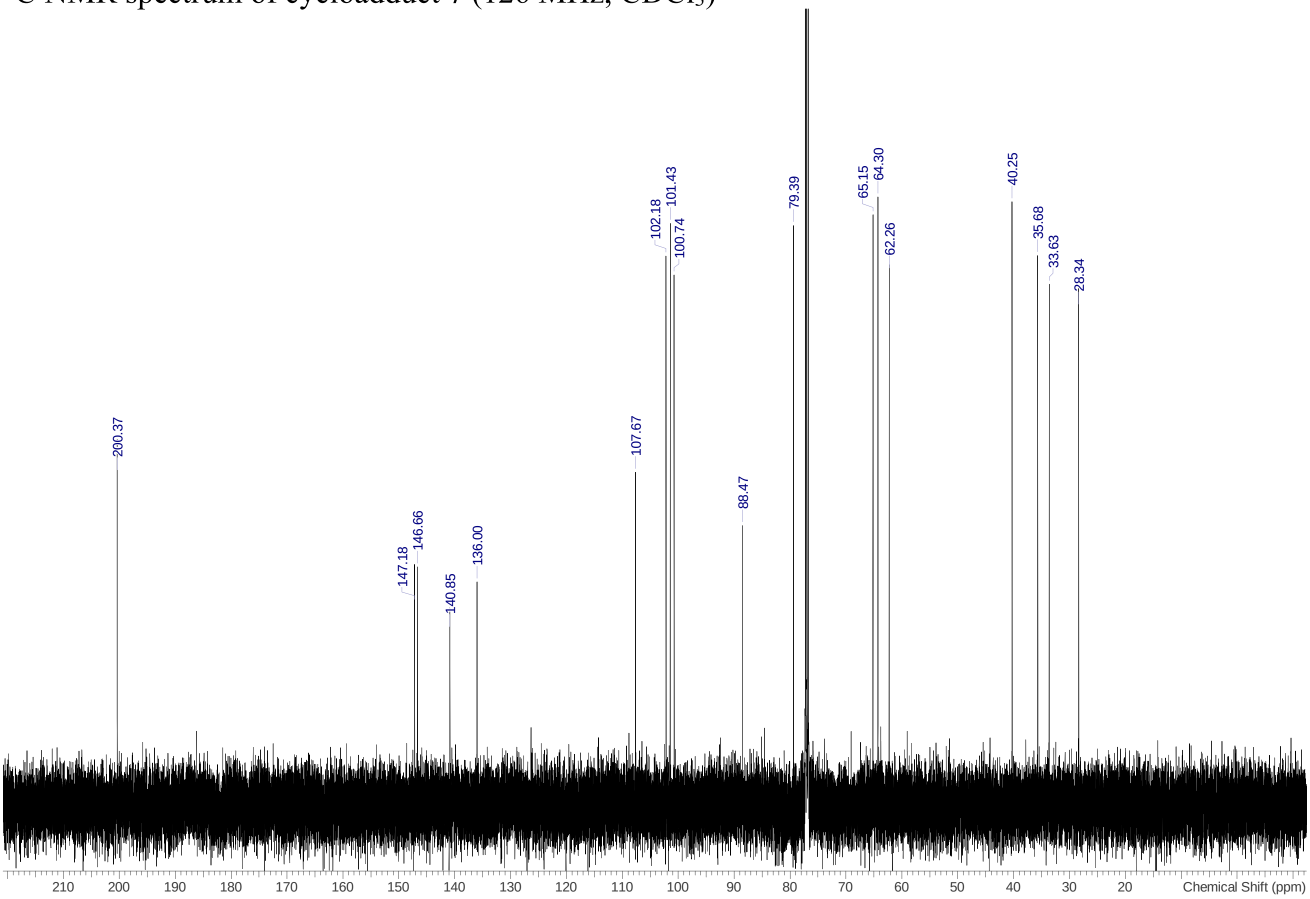

^{1}H NMR spectrum of cycloadduct **8** (500 MHz, C_6D_6)

8

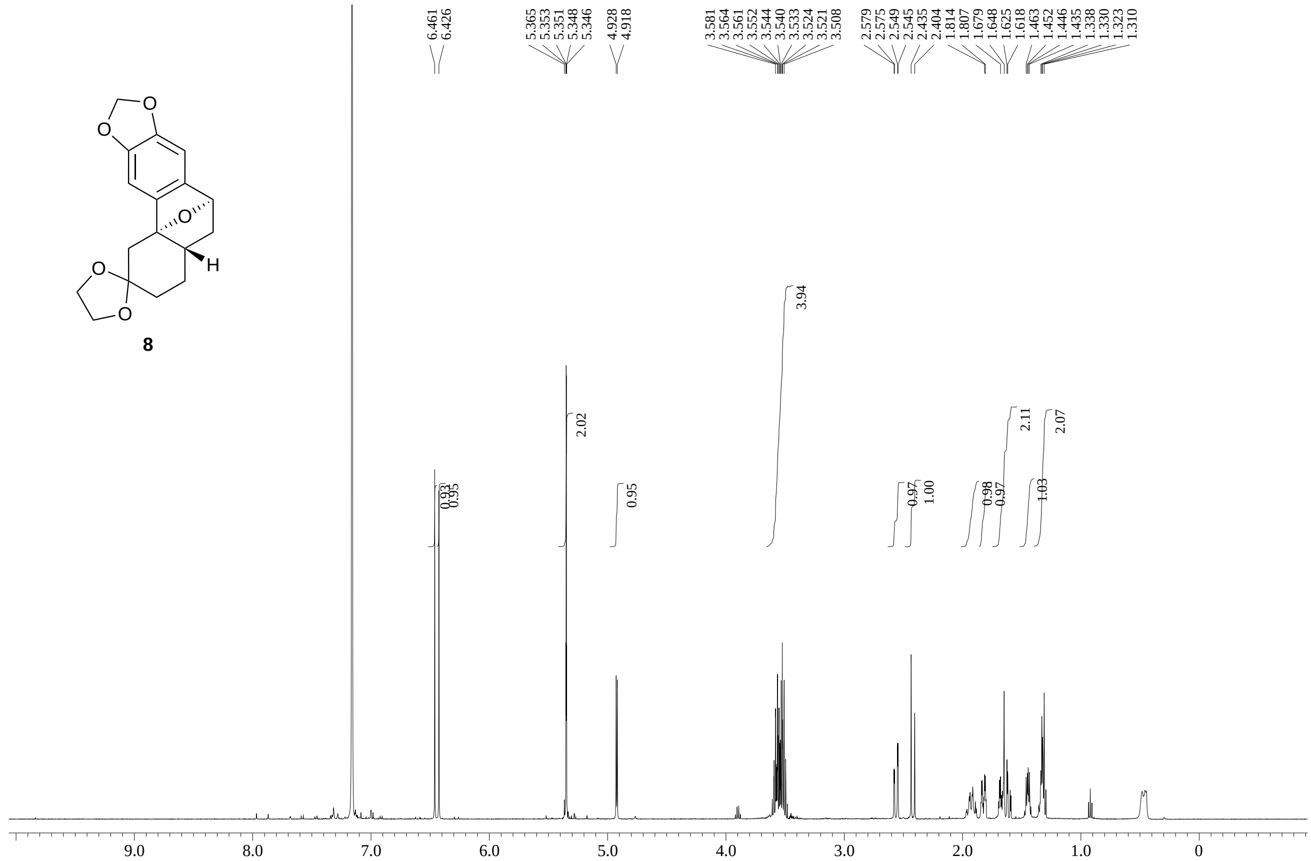

^{13}C NMR spectrum of cycloadduct **8** (126 MHz, C_6D_6)

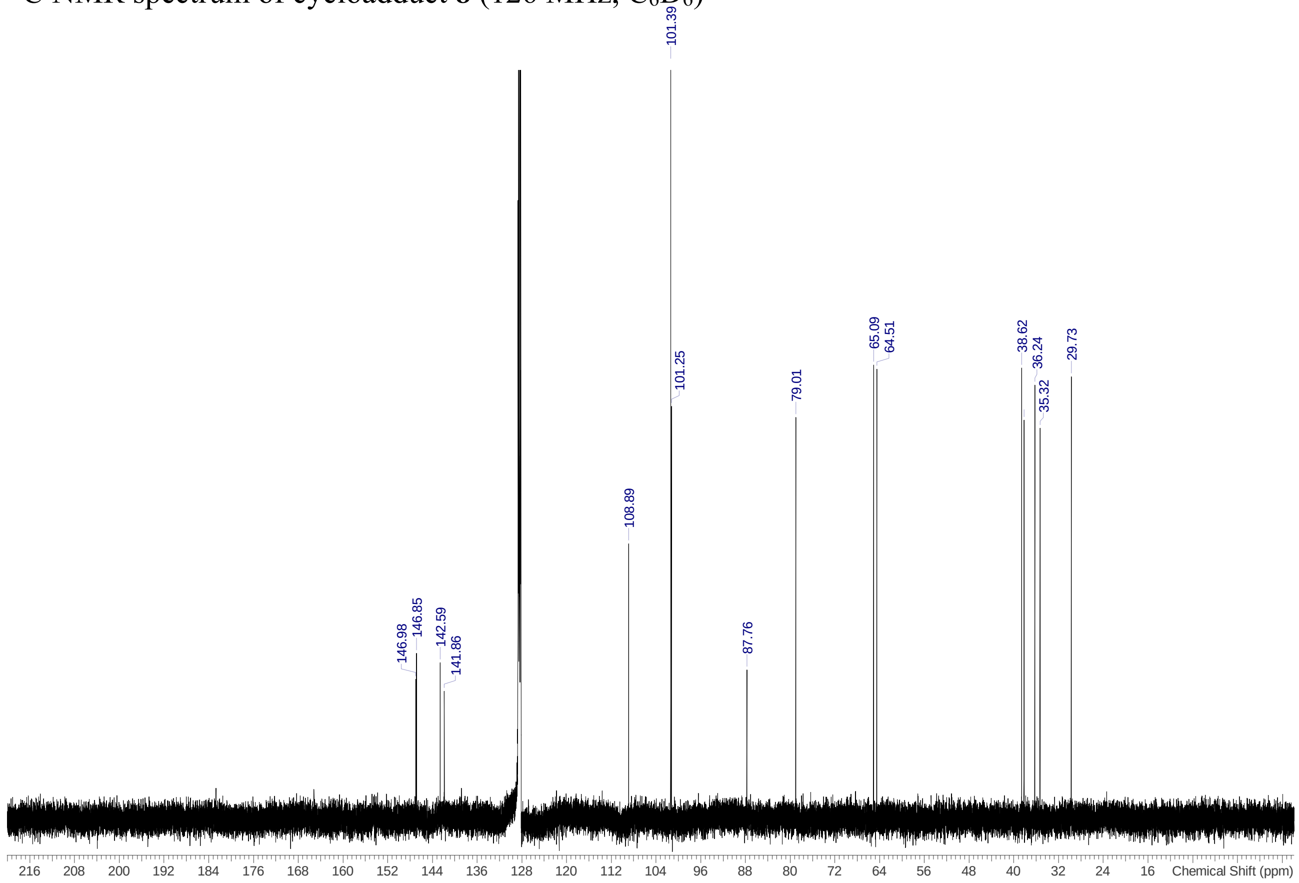

^{1}H NMR spectrum of aromatized product **16** (500 MHz, $CDCl_3$)

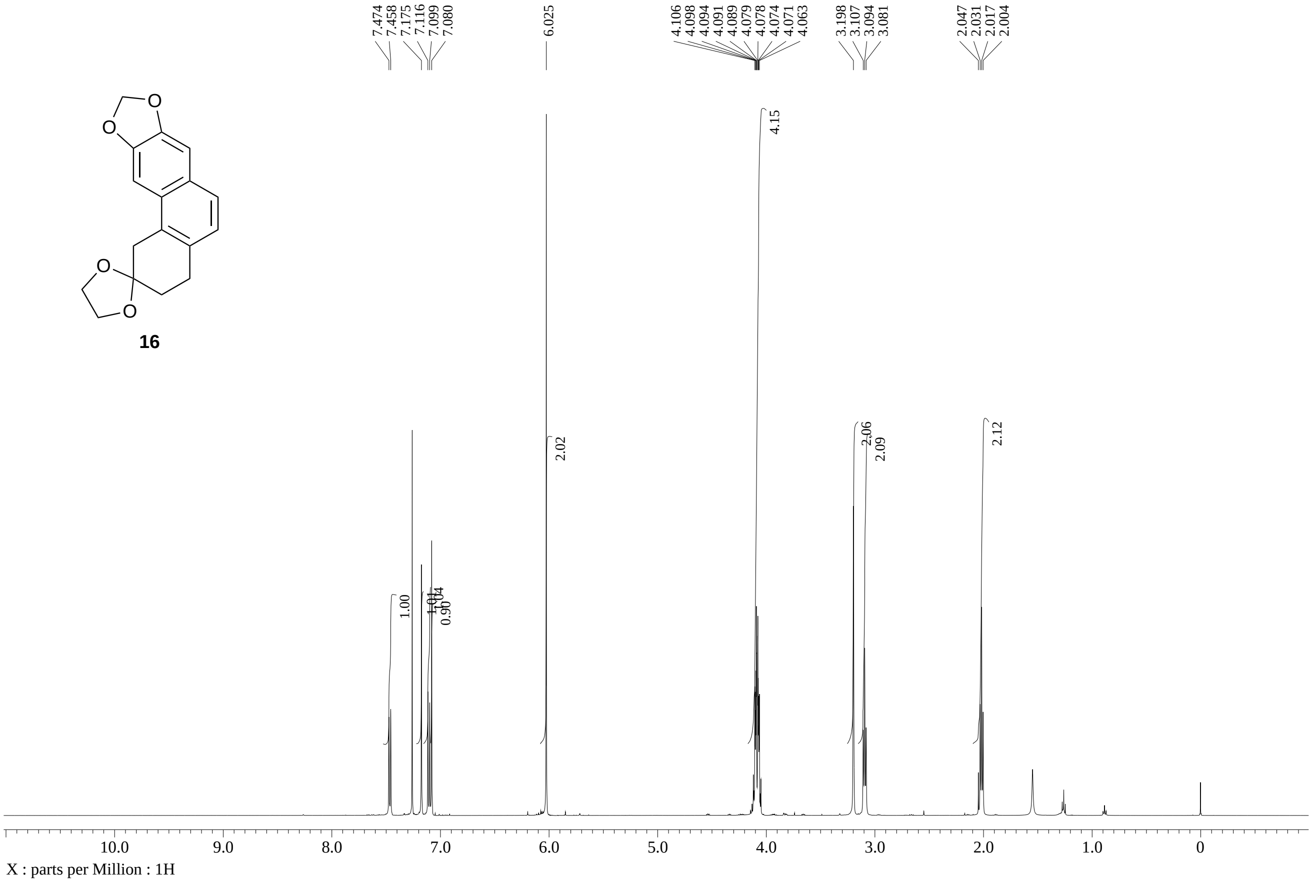

^{13}C NMR spectrum of aromatized product **16** (126 MHz, $CDCl_3$)

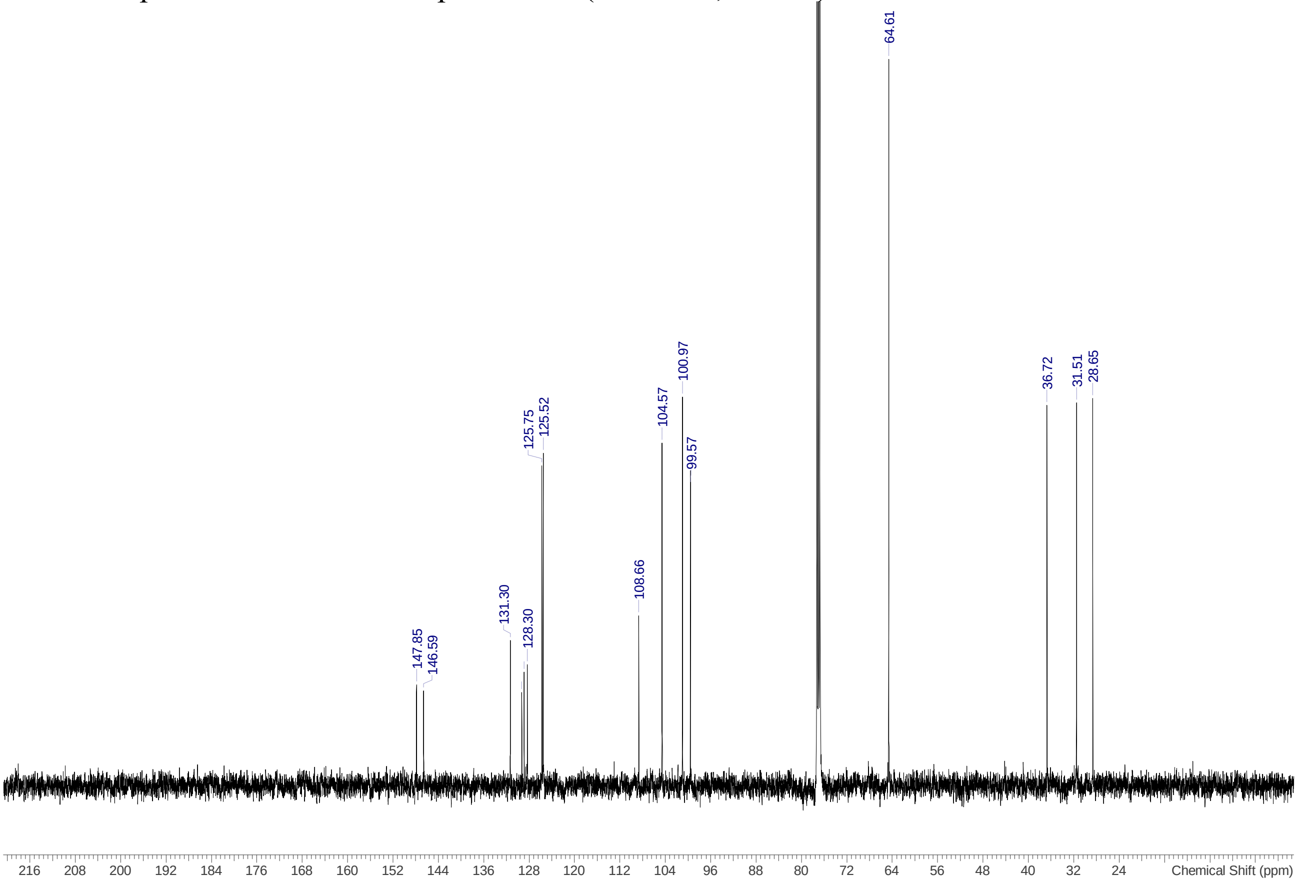

^{1}H NMR spectrum of aromatized product **17** (500 MHz, $CDCl_3$)

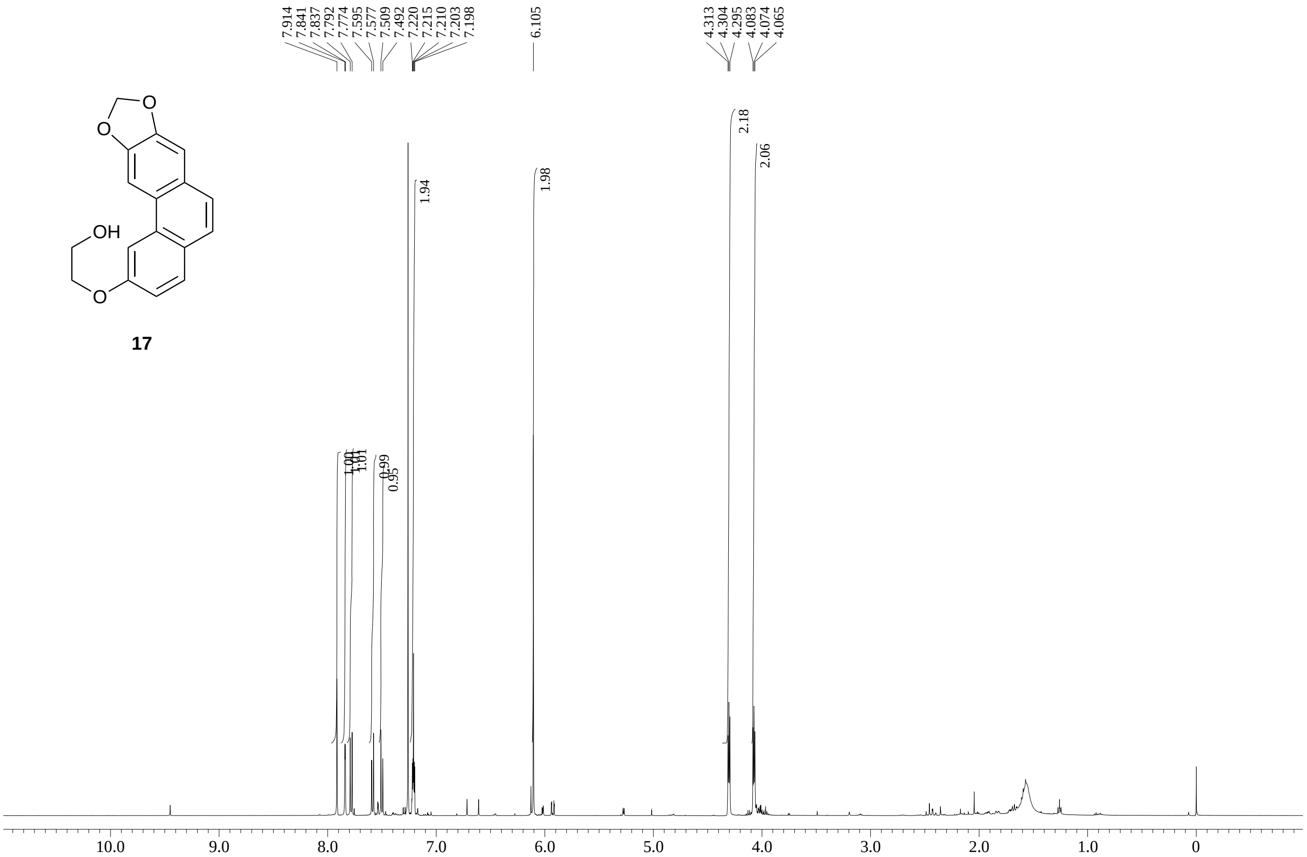

^{13}C NMR spectrum of aromatized product **17** (126 MHz, $CDCl_3$)

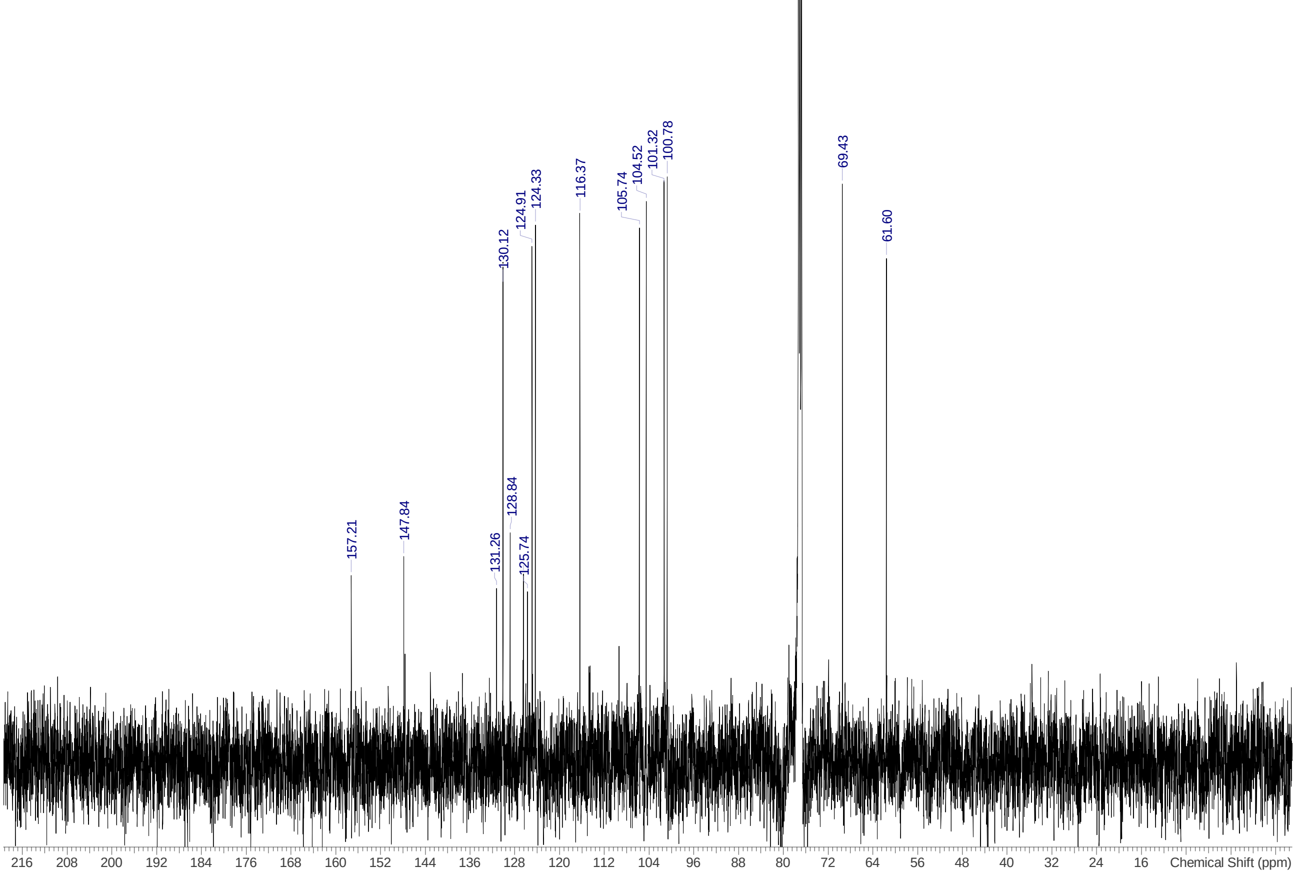

^{1}H NMR spectrum of cycloadduct **9** (500 MHz, C_6D_6)

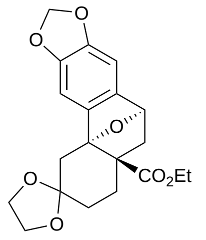

9

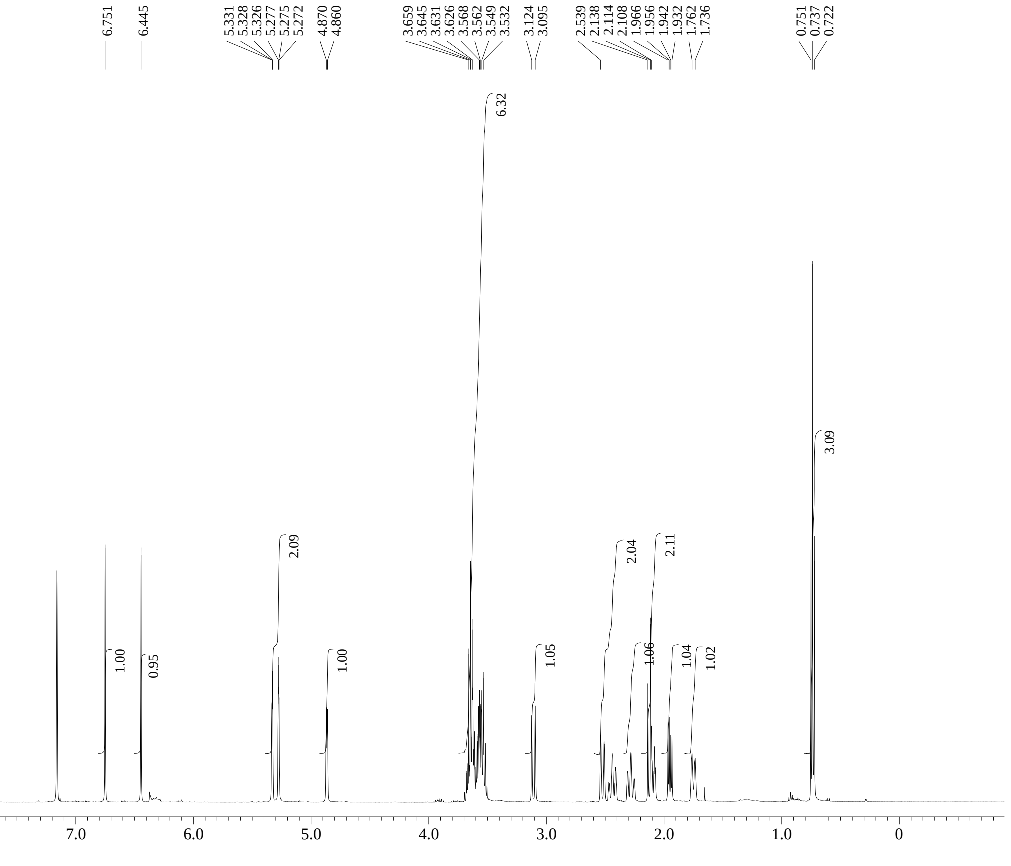

^{13}C NMR spectrum of cycloadduct **9** (126 MHz, C_6D_6)

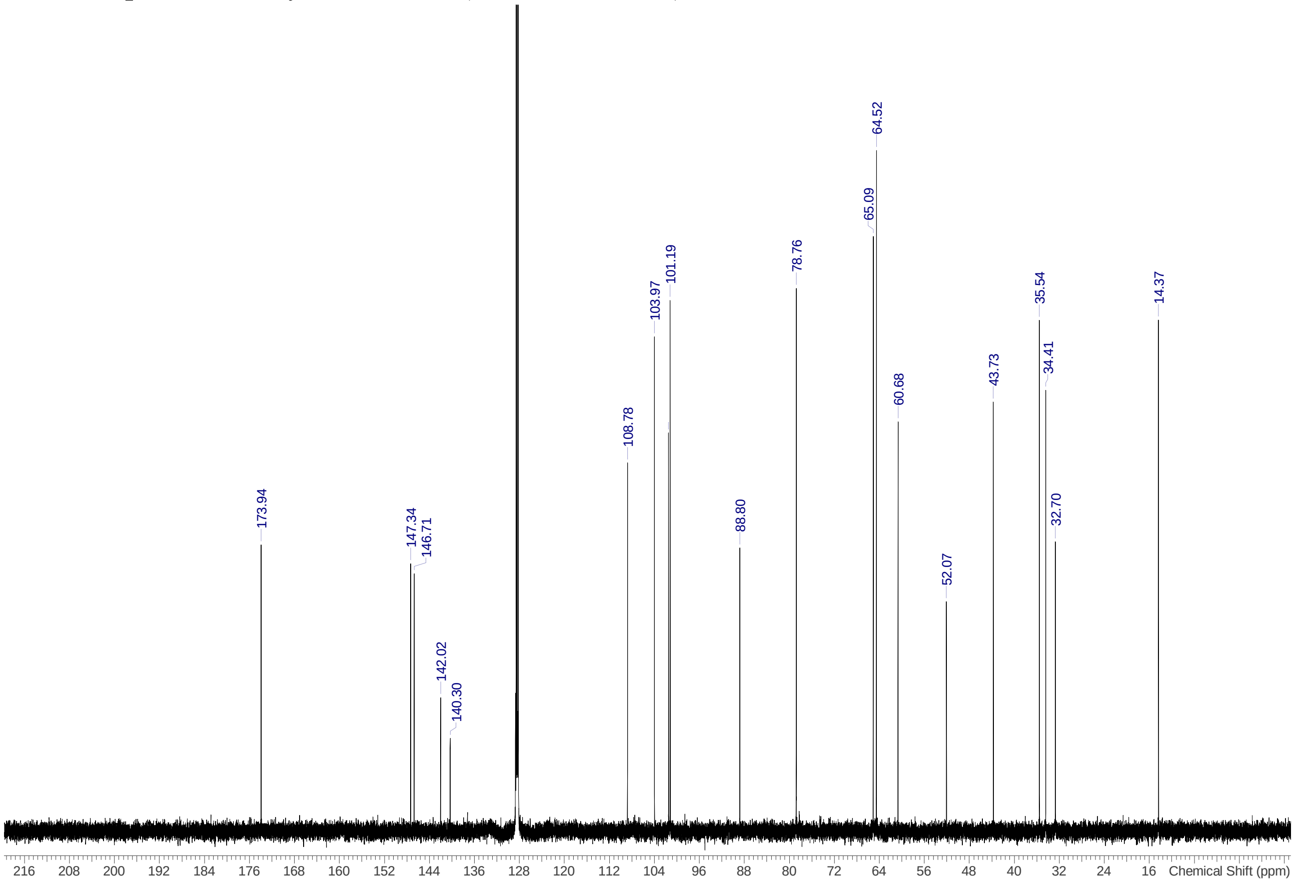

^{1}H NMR spectrum of cycloadduct **10** (500 MHz, C_6D_6)

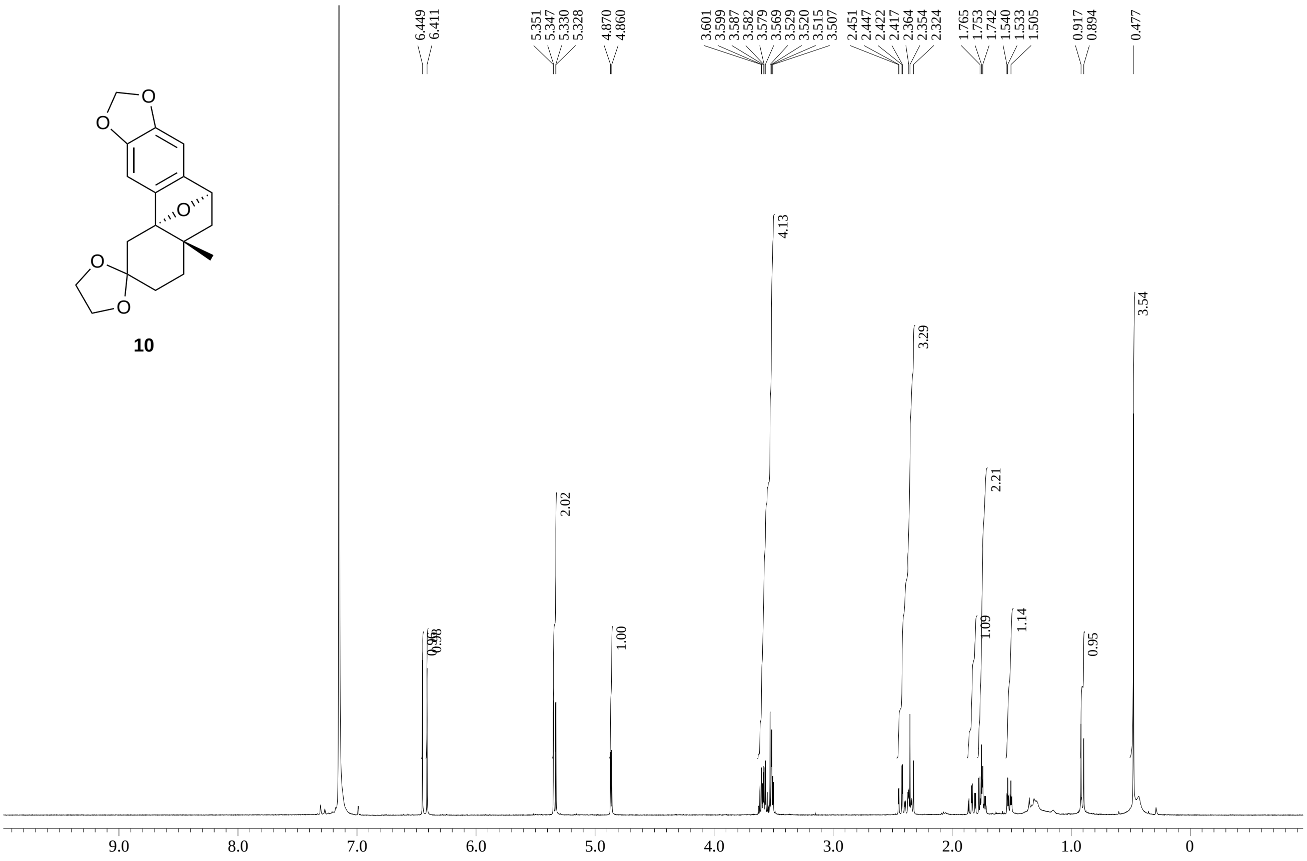

^{13}C NMR spectrum of cycloadduct **10** (126 MHz, C_6D_6)

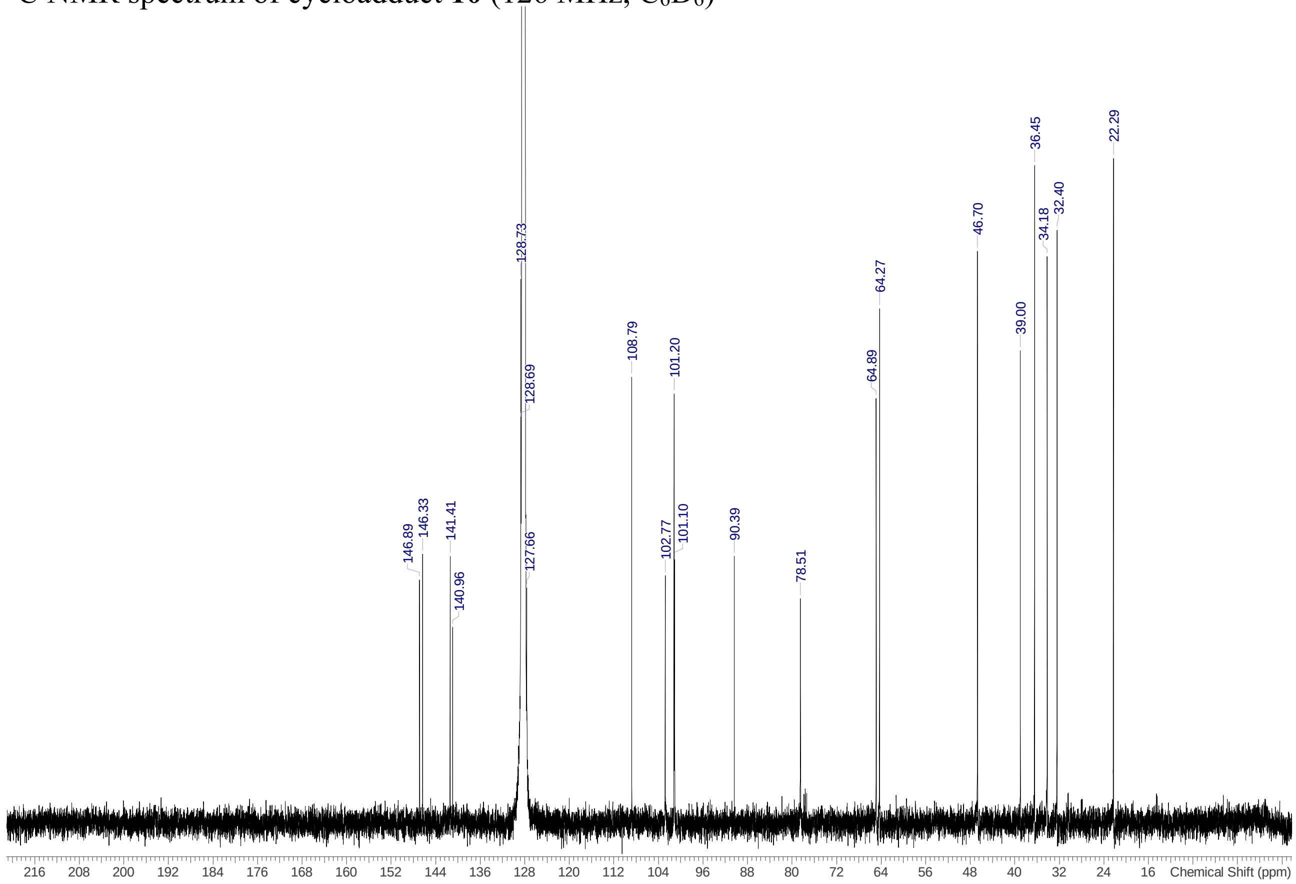

^{1}H NMR spectrum of cycloadduct **12** (500 MHz, $CDCl_3$)

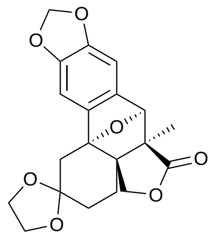

12

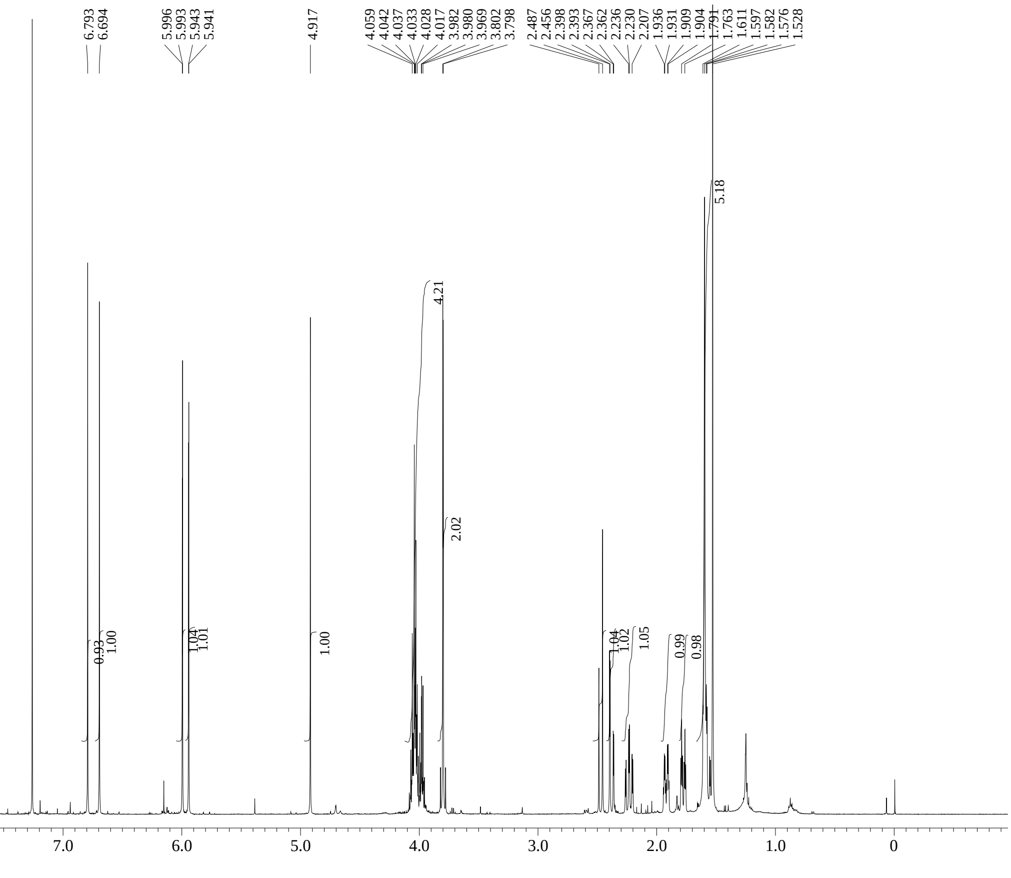

^{13}C NMR spectrum of cycloadduct **12** (126 MHz, $CDCl_3$)

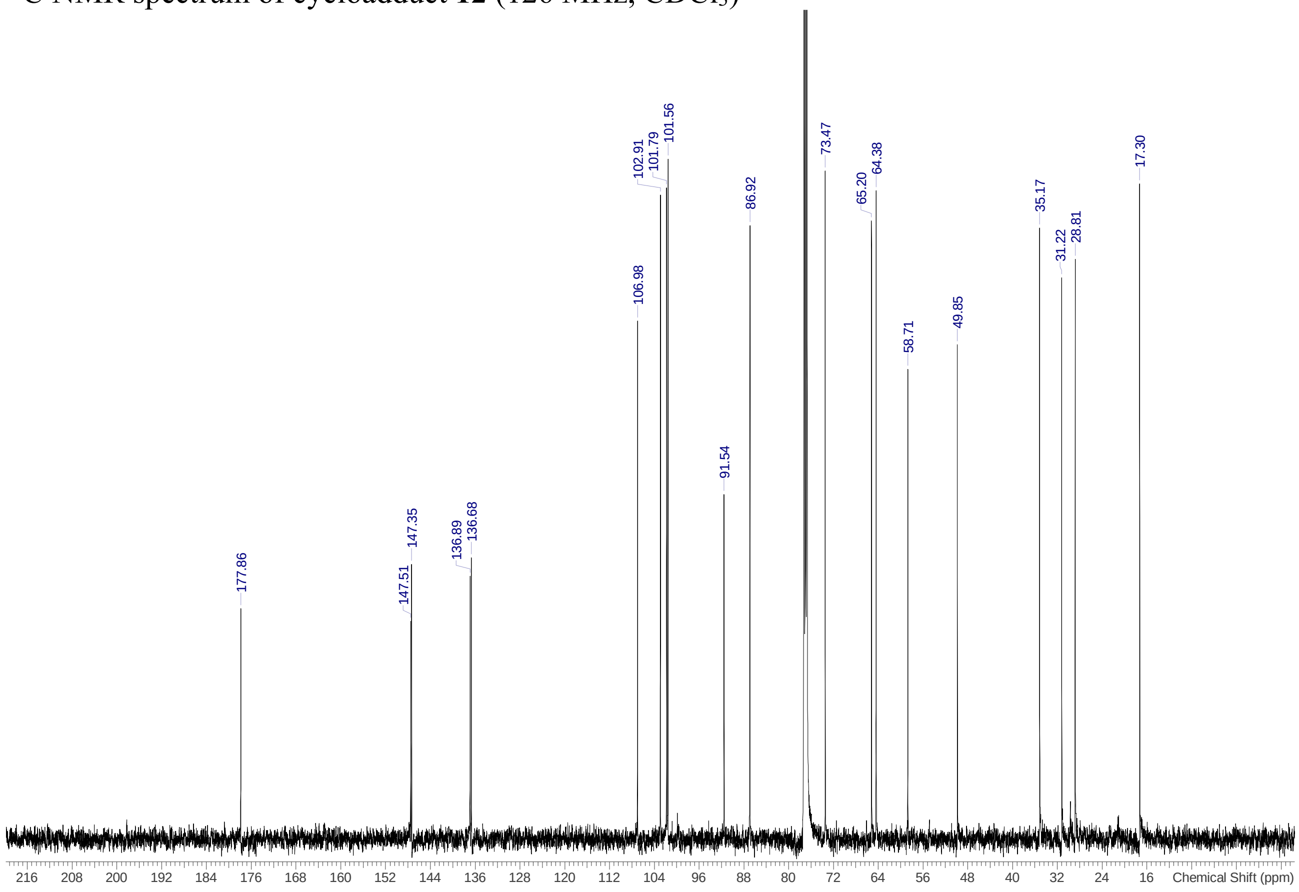

^{1}H NMR spectrum of cycloadduct **13** (500 MHz, $CDCl_3$)

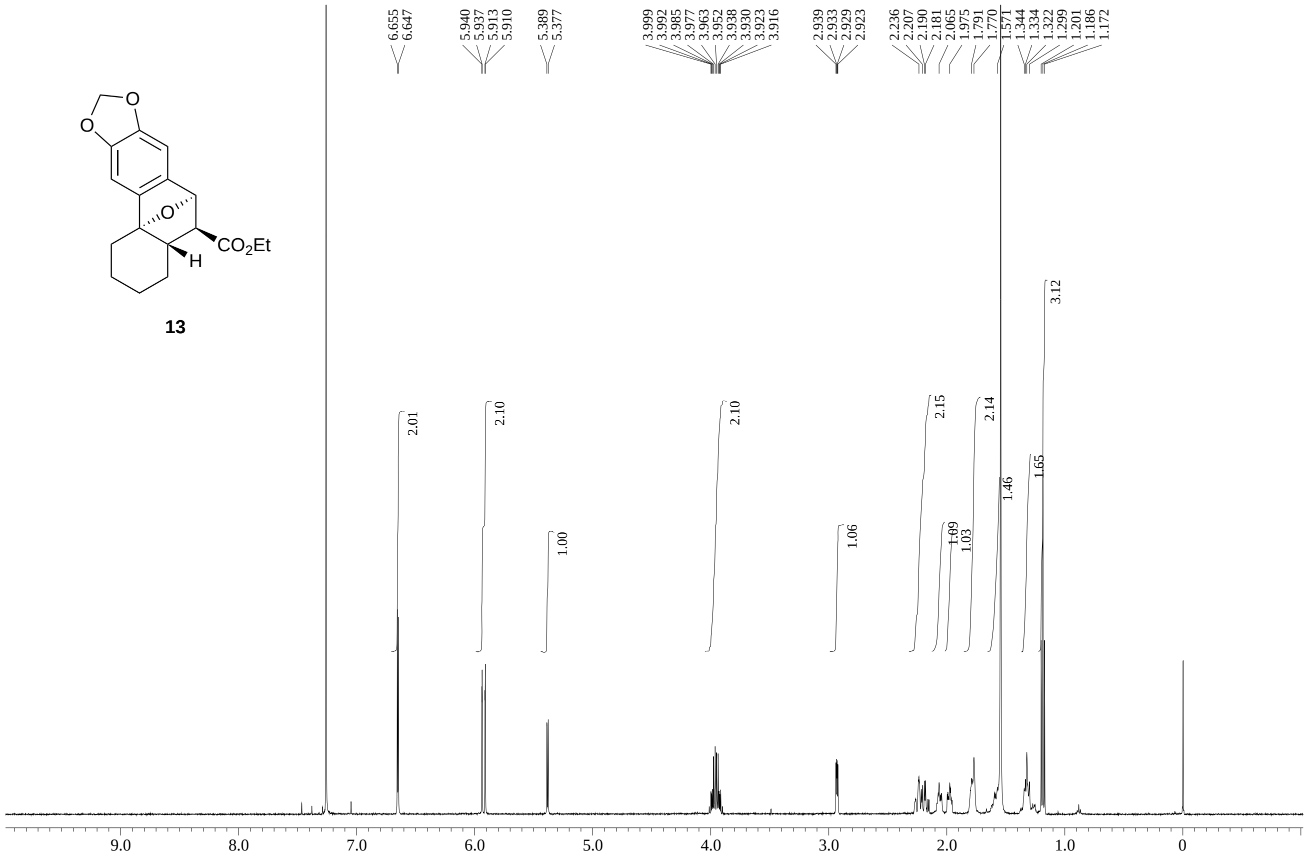

^{13}C NMR spectrum of cycloadduct **13** (126 MHz, $CDCl_3$)

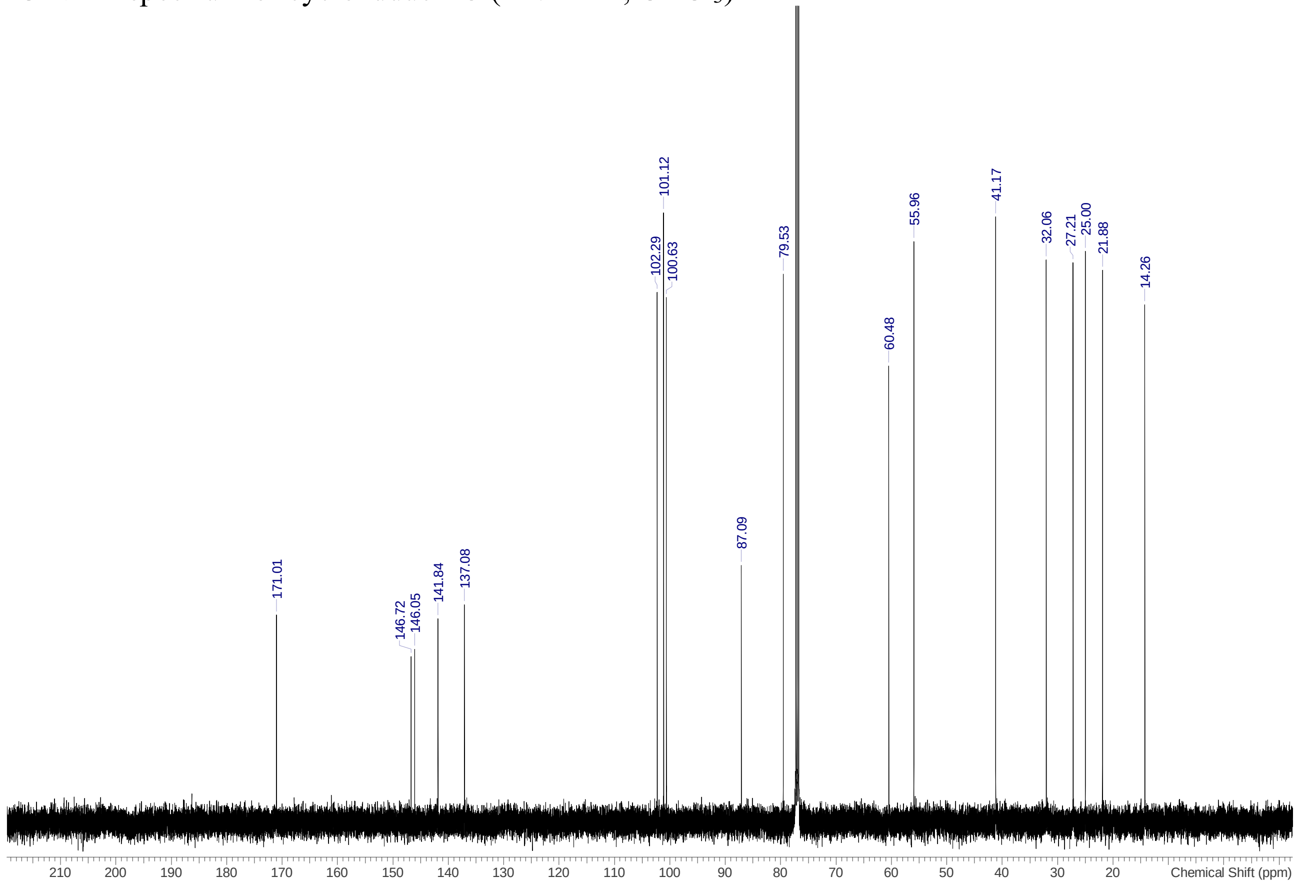

^{1}H NMR spectrum of cycloadduct **14** (500 MHz, $CDCl_3$)

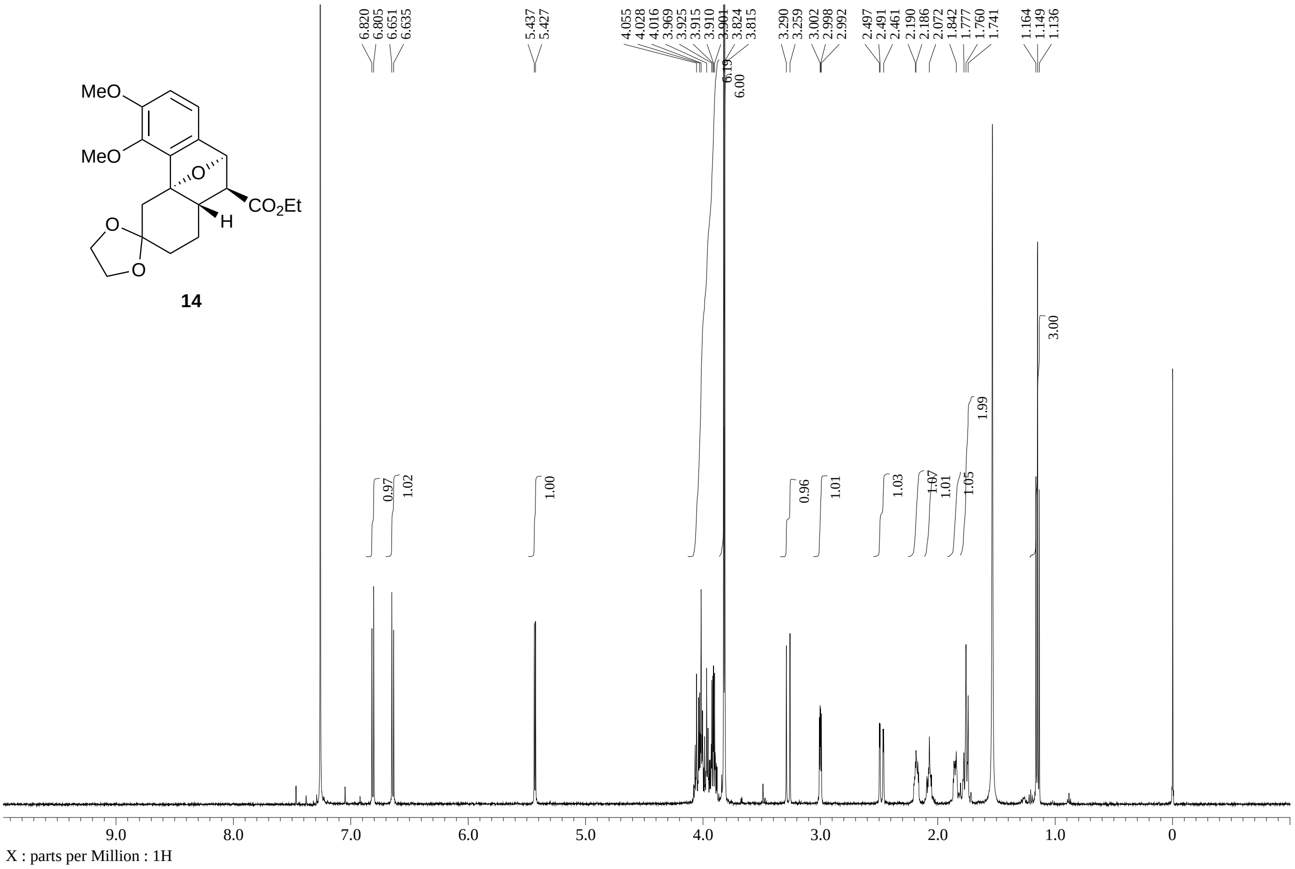

^{13}C NMR spectrum of cycloadduct **14** (126 MHz, $CDCl_3$)

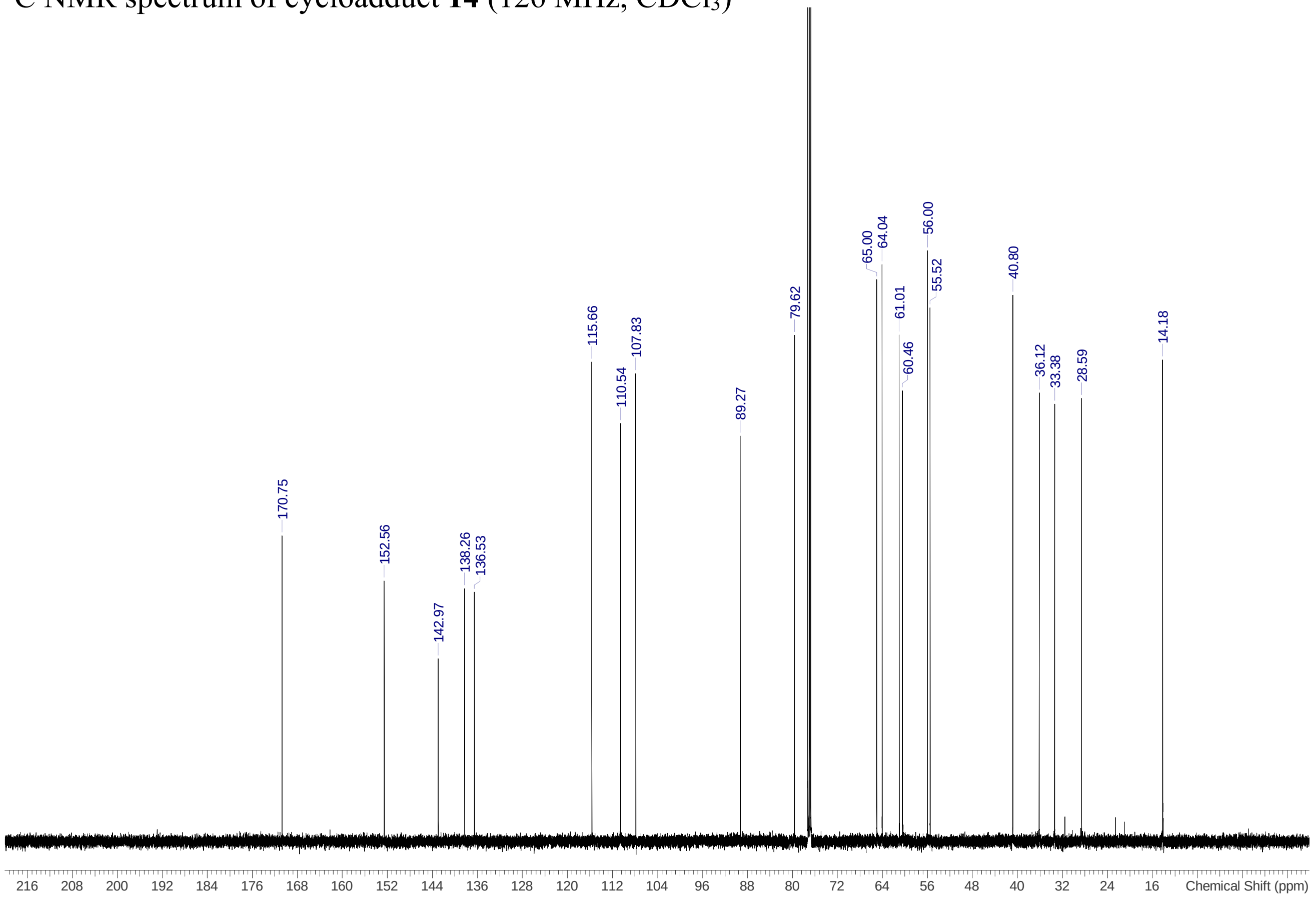

^{1}H NMR spectrum of cycloadduct **15** (500 MHz, $CDCl_3$)

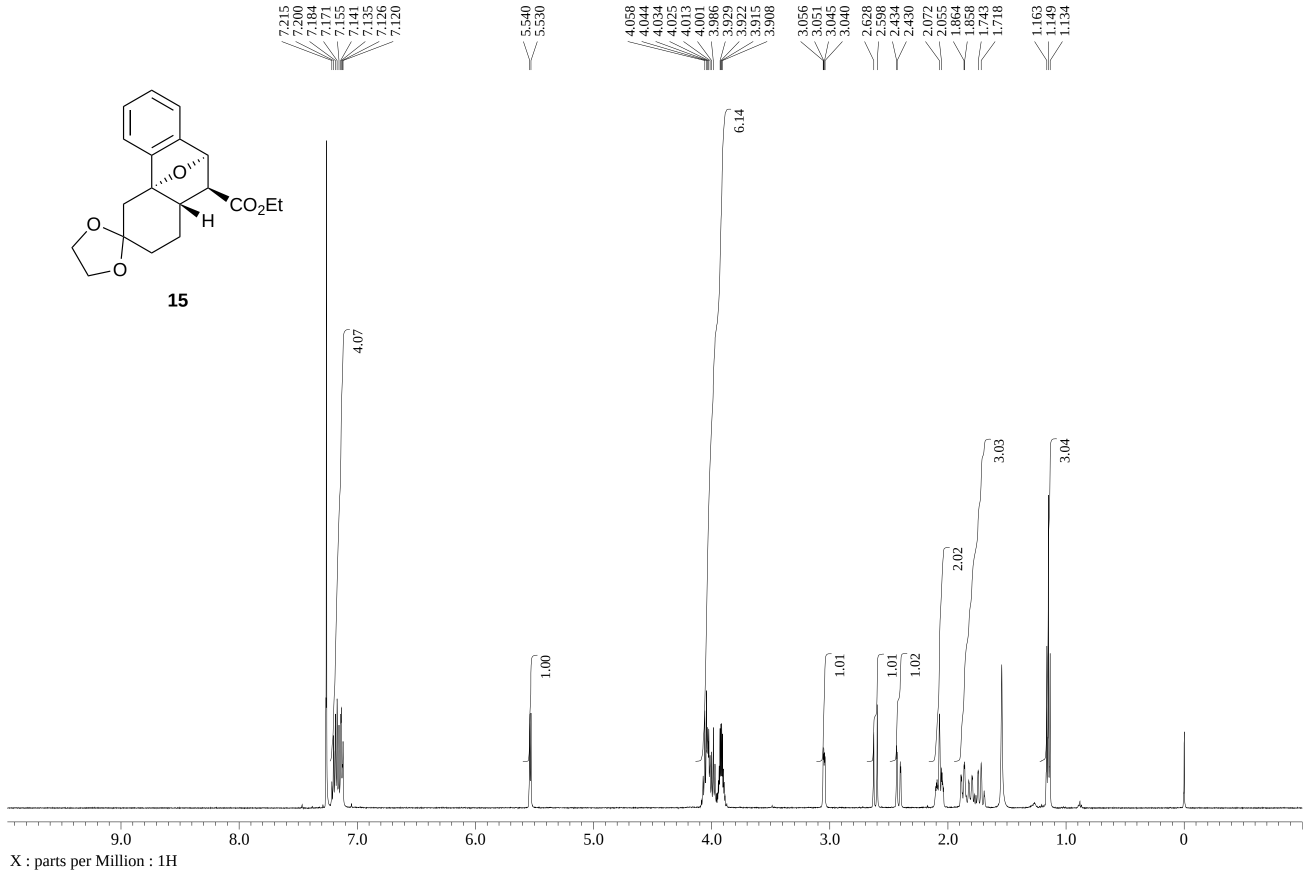

^{13}C NMR spectrum of cycloadduct **15** (126 MHz, $CDCl_3$)

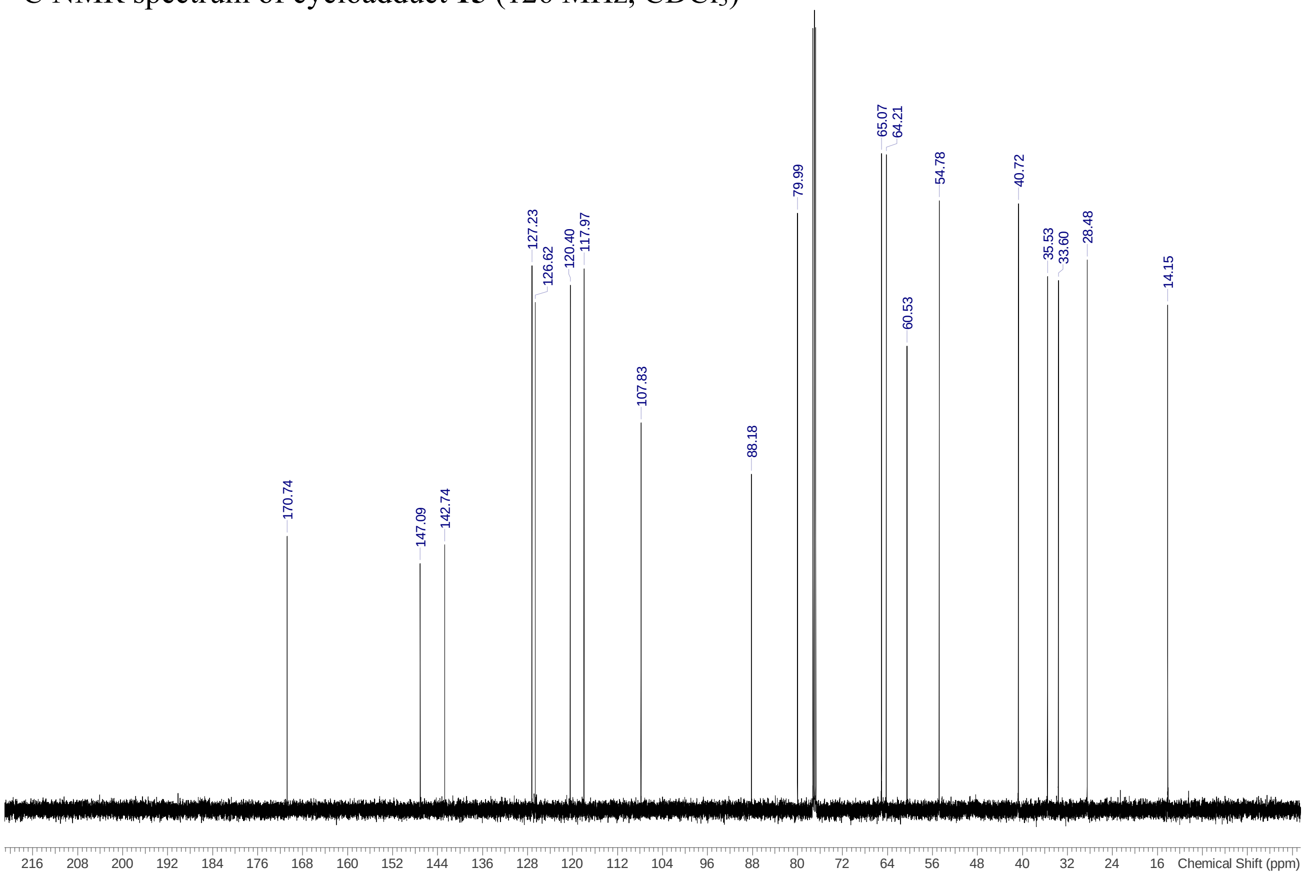

^{1}H NMR spectrum of cycloadduct **19** (500 MHz, $CDCl_3$)

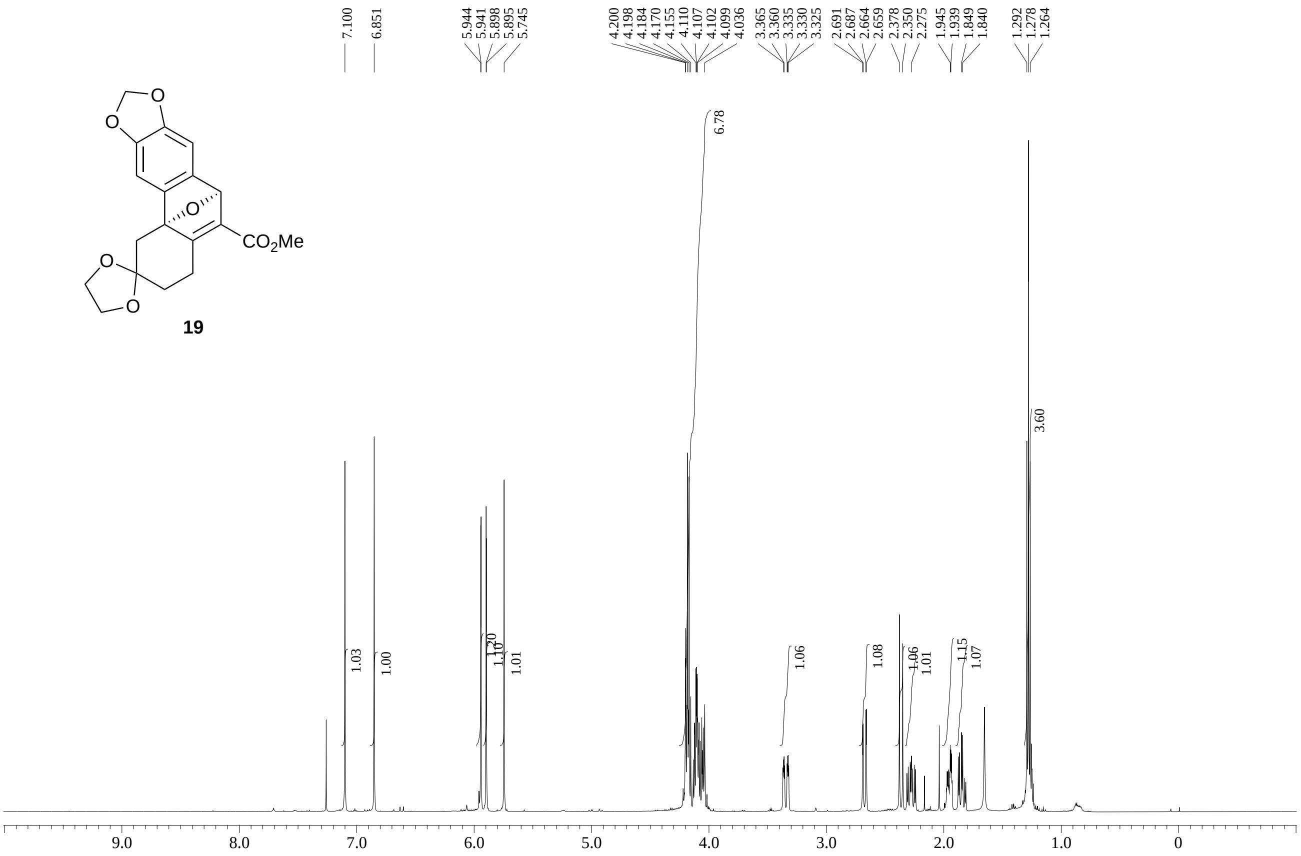

^{13}C NMR spectrum of cycloadduct **19** (126 MHz, $CDCl_3$)

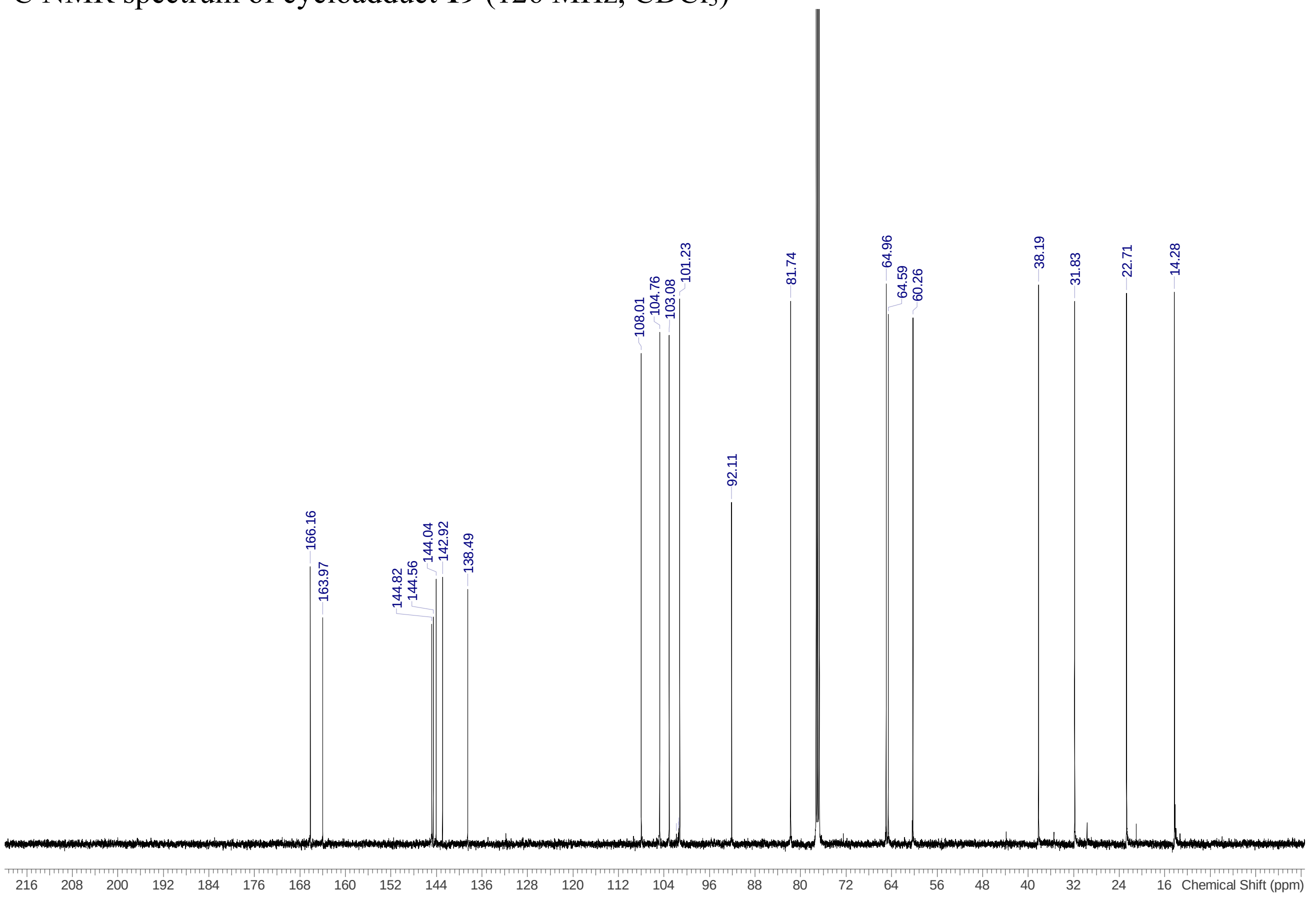

^{1}H NMR spectrum of enone **21** (500 MHz, $CDCl_3$)

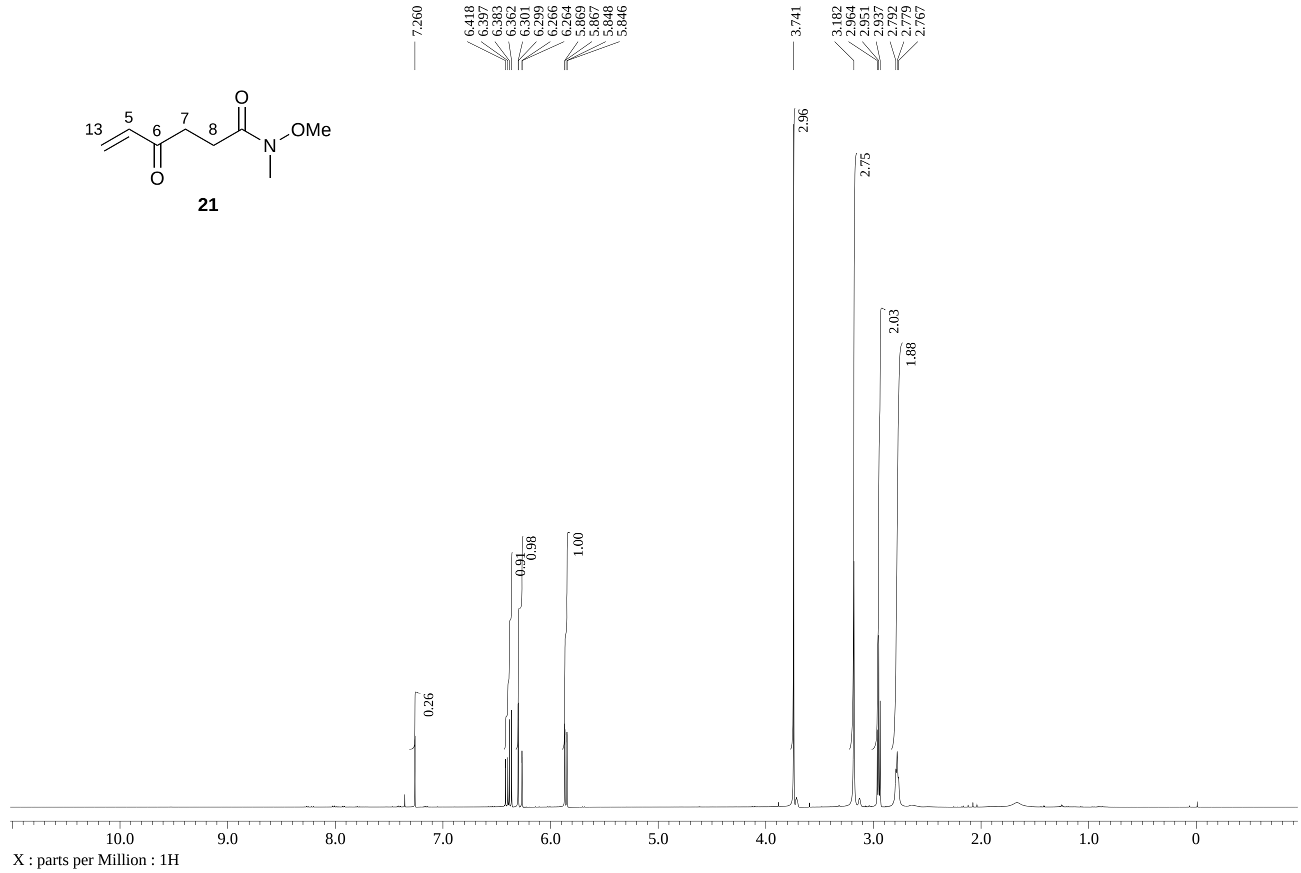

^{13}C NMR spectrum of enone **21** (126 MHz, $CDCl_3$)

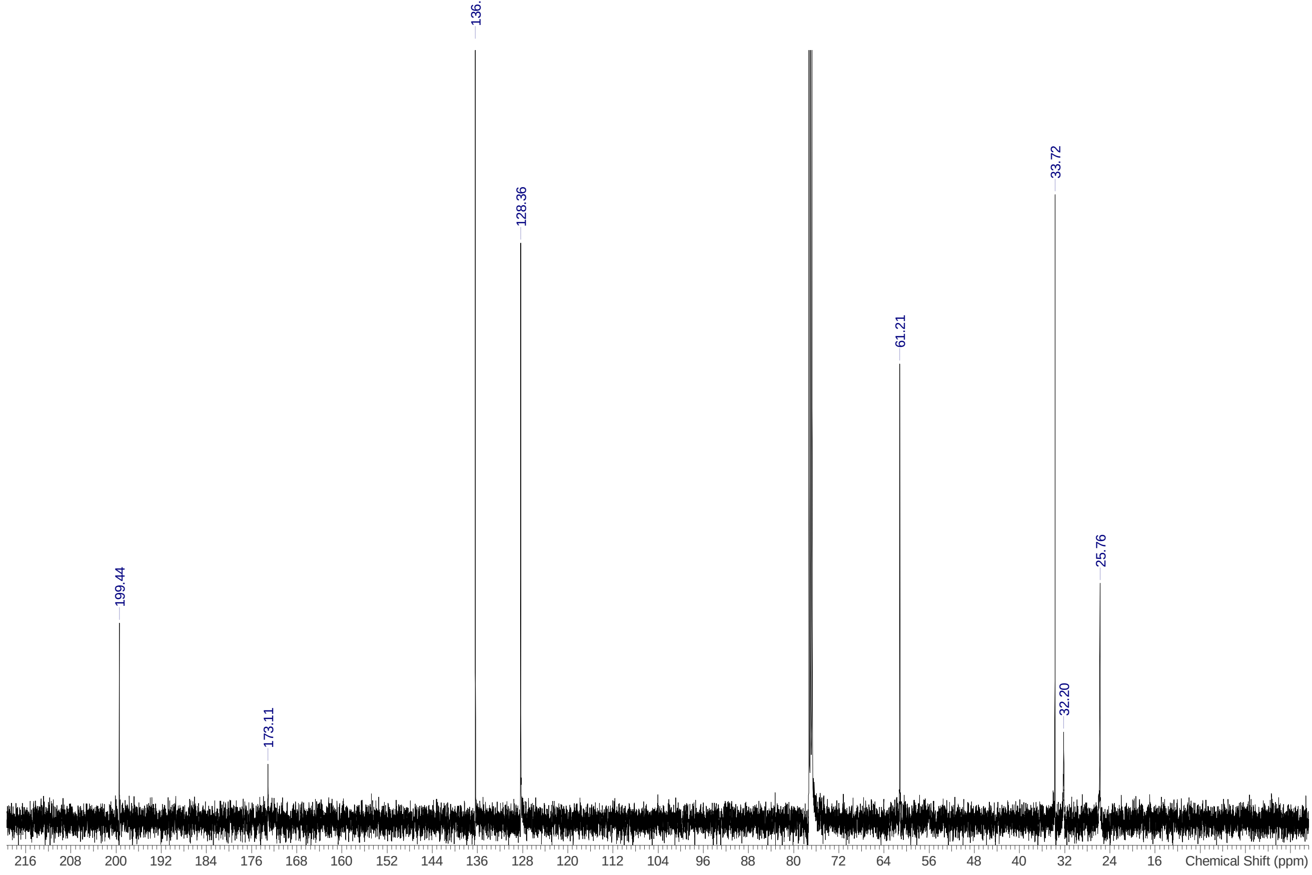

^{1}H NMR spectrum of phthalan **22** (500 MHz, $CDCl_3$)

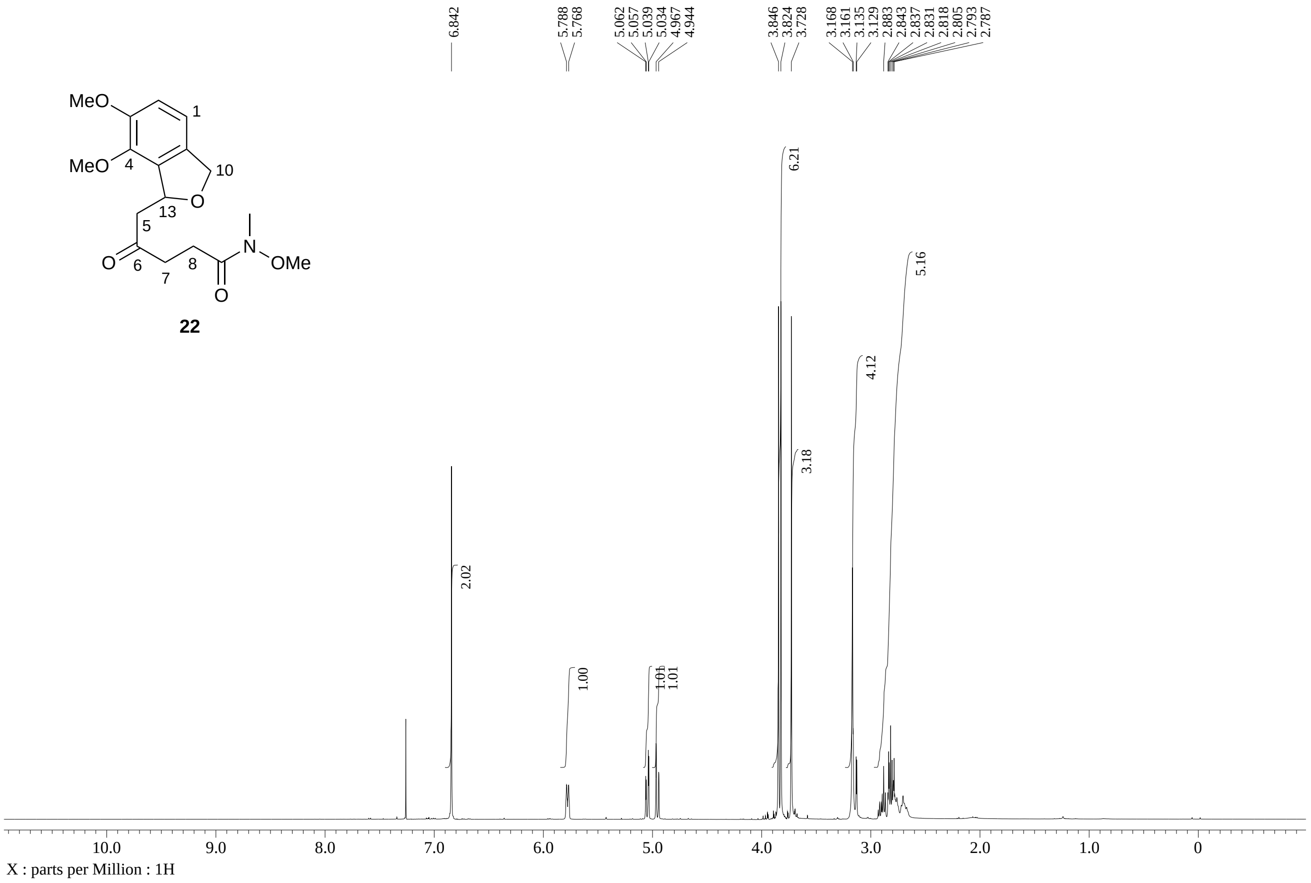

^{13}C NMR spectrum of phthalan **22** (126 MHz, $CDCl_3$)

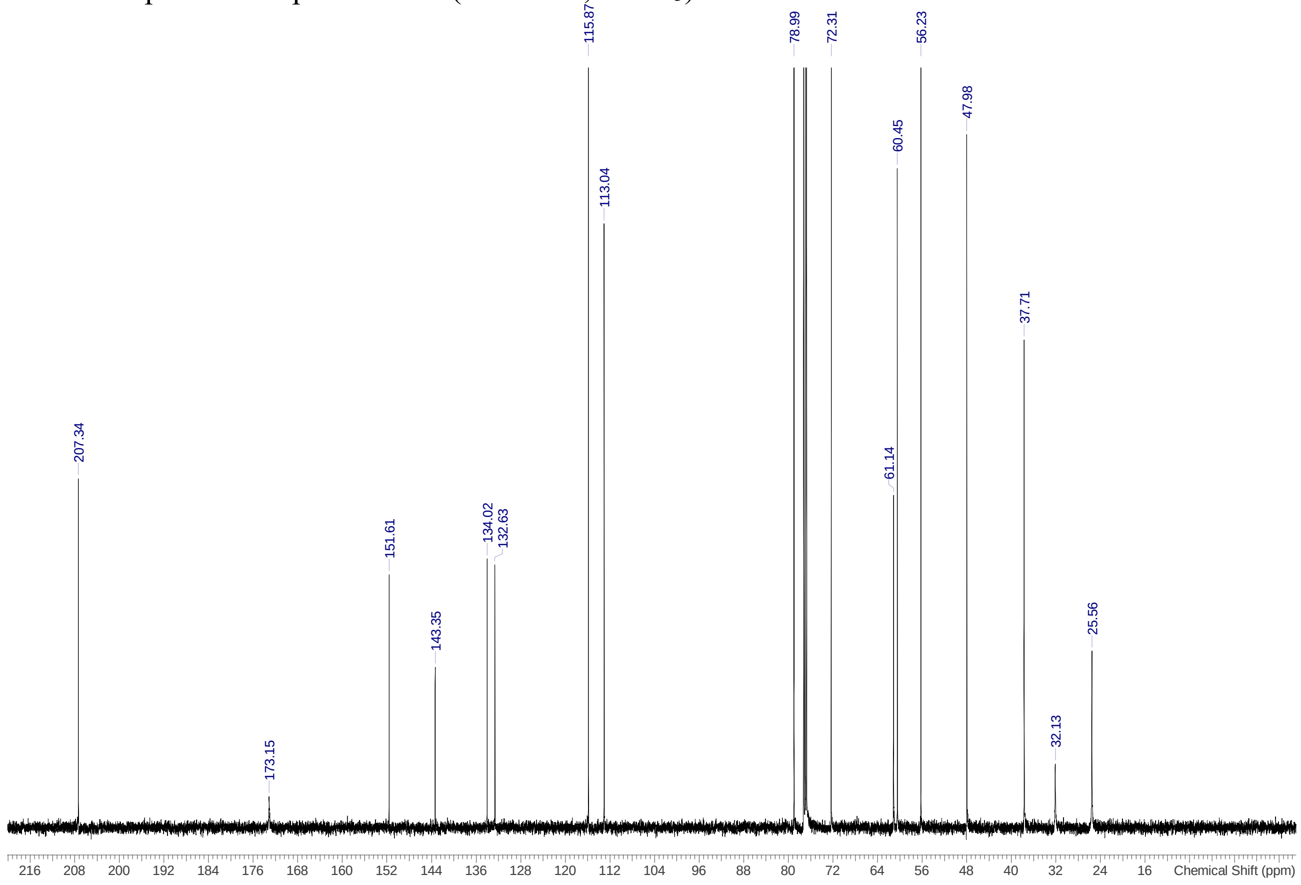

^{1}H NMR spectrum of TBS ether **23** (600 MHz, $CDCl_3$, 50 °C)

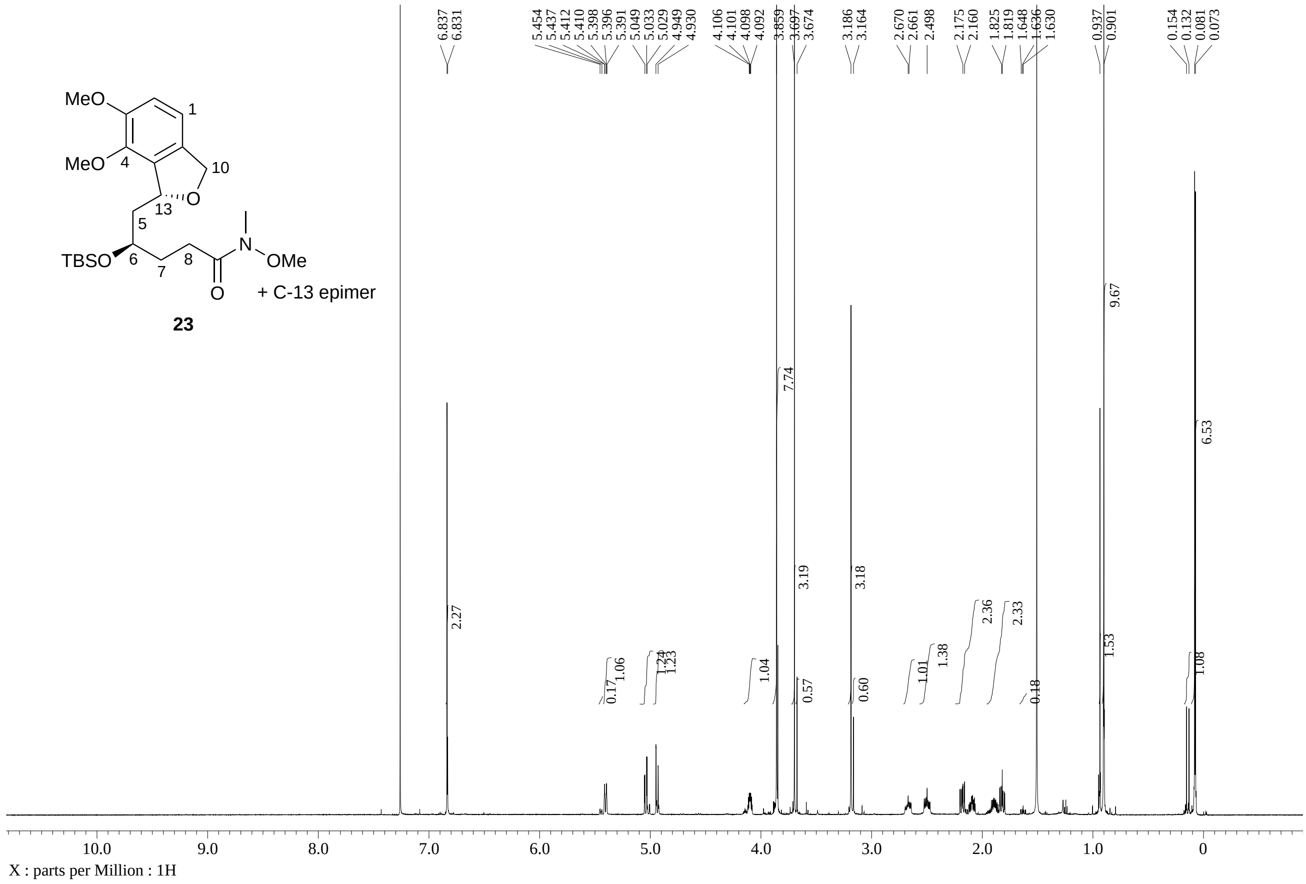

^{13}C NMR spectrum of TBS ether **23** (150 MHz, $CDCl_3$, 50 °C)

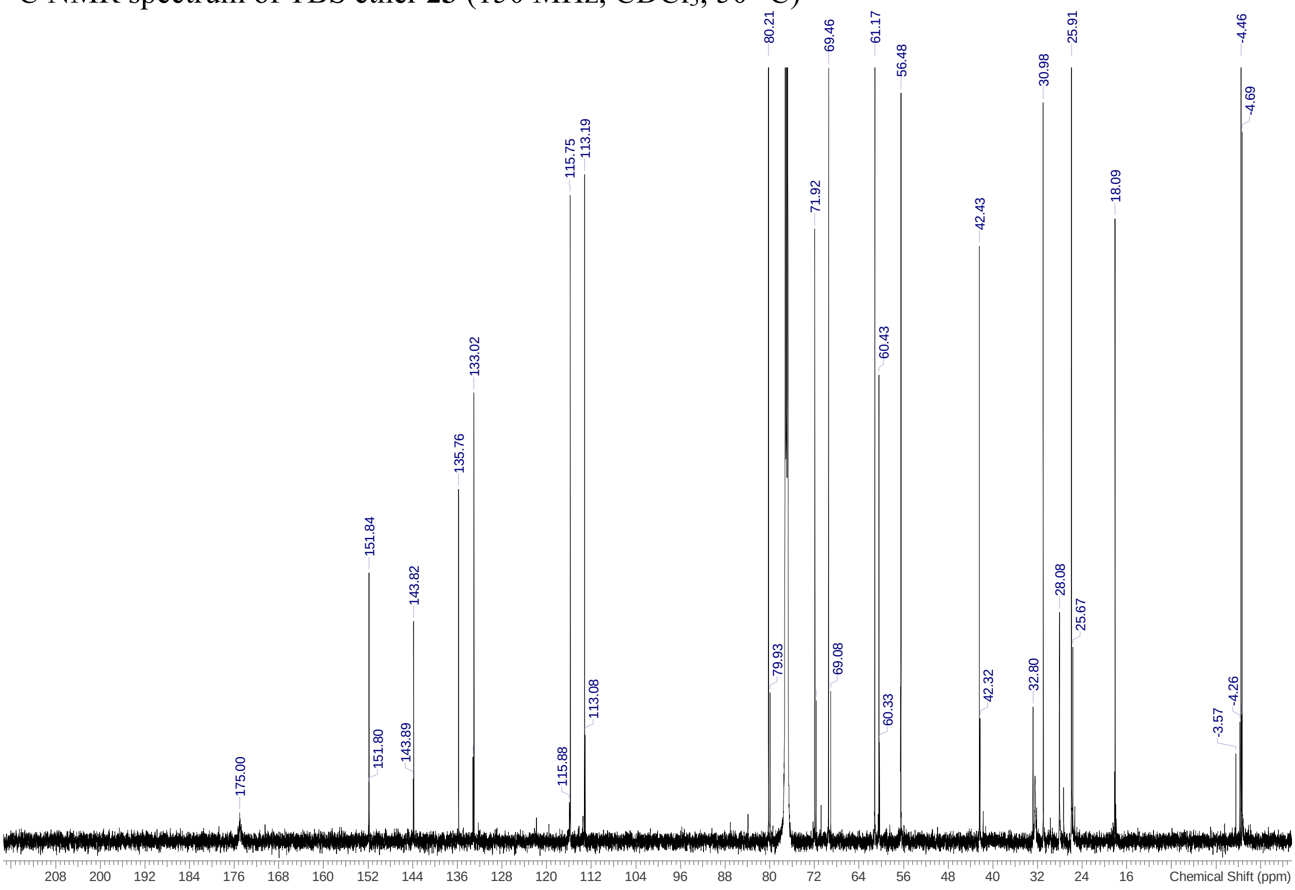

^{1}H NMR spectrum of aldehyde **24** (500 MHz, $CDCl_3$)

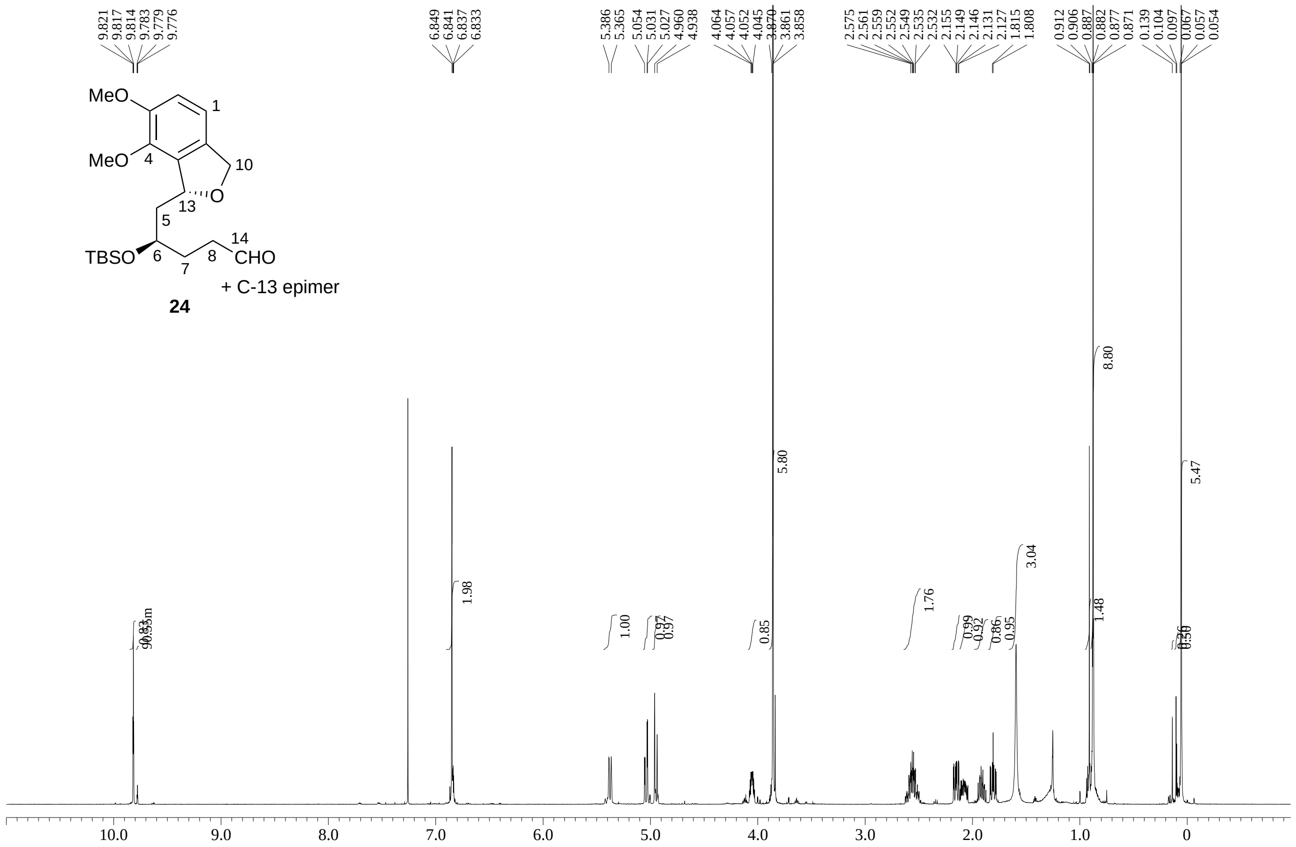

^{13}C NMR spectrum of aldehyde **24** (126 MHz, $CDCl_3$)

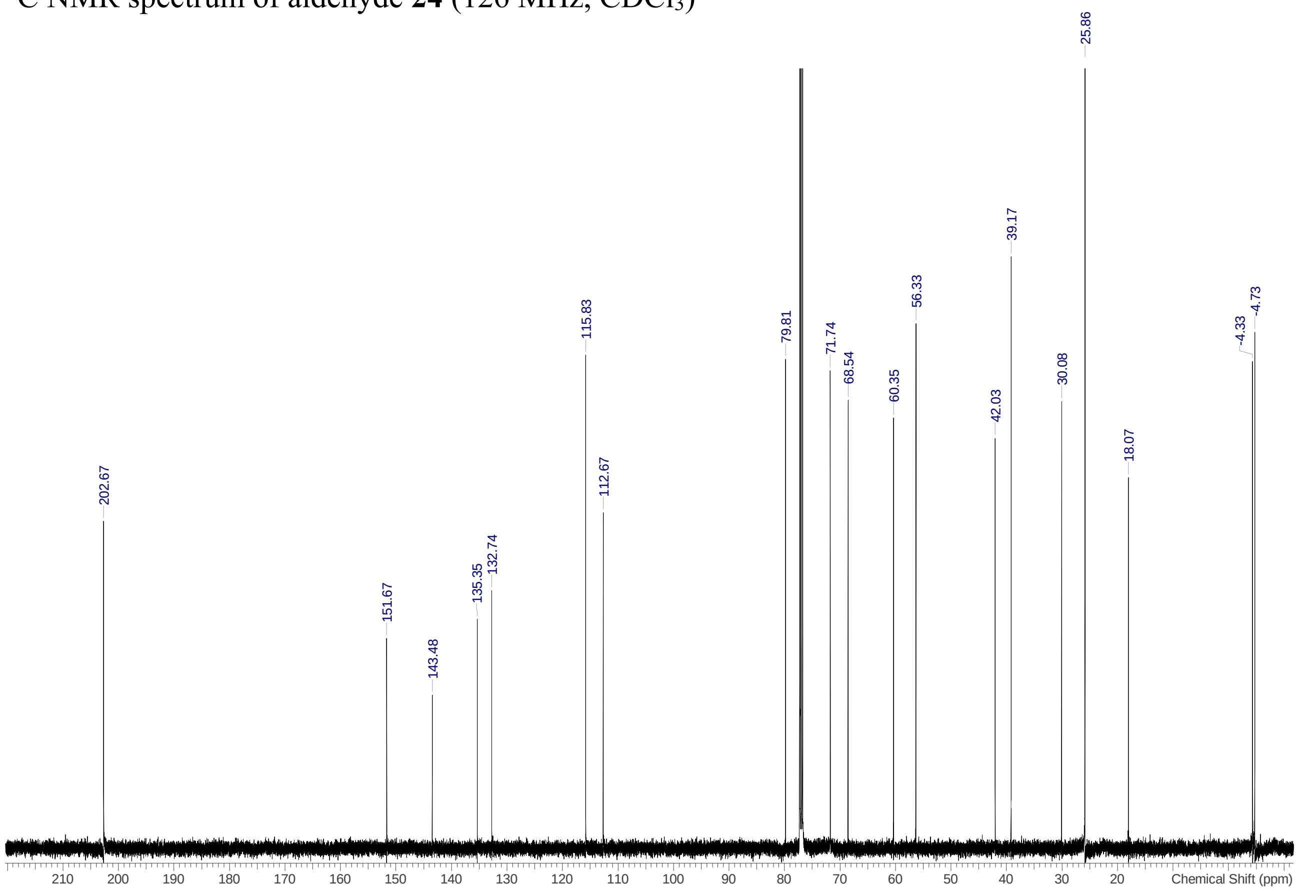

^{1}H NMR spectrum of nitroalkene **25** (500 MHz, $CDCl_3$)

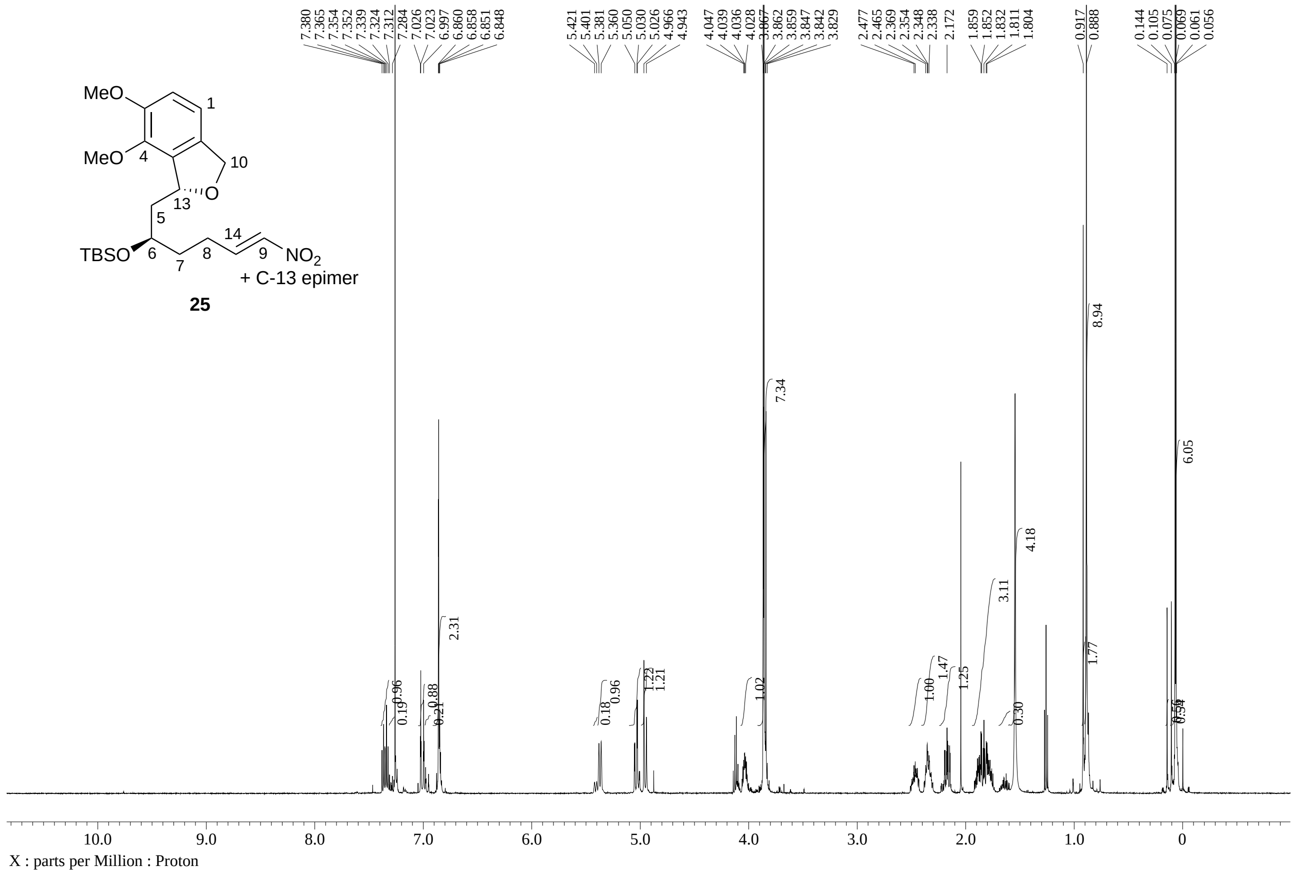

^{13}C NMR spectrum of nitroalkene **25** (126 MHz, $CDCl_3$)

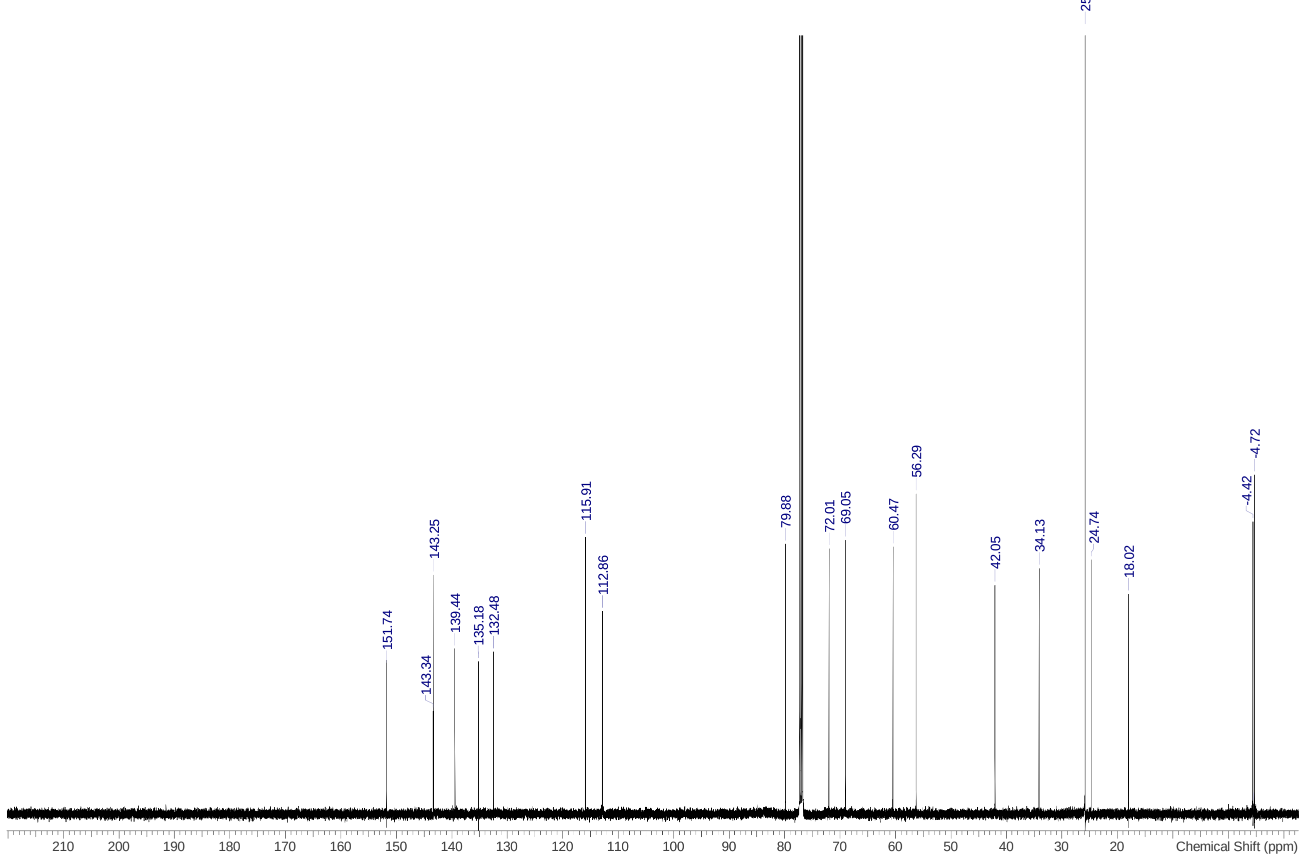

^{1}H NMR spectrum of cycloadduct **26** (500 MHz, $CDCl_3$)

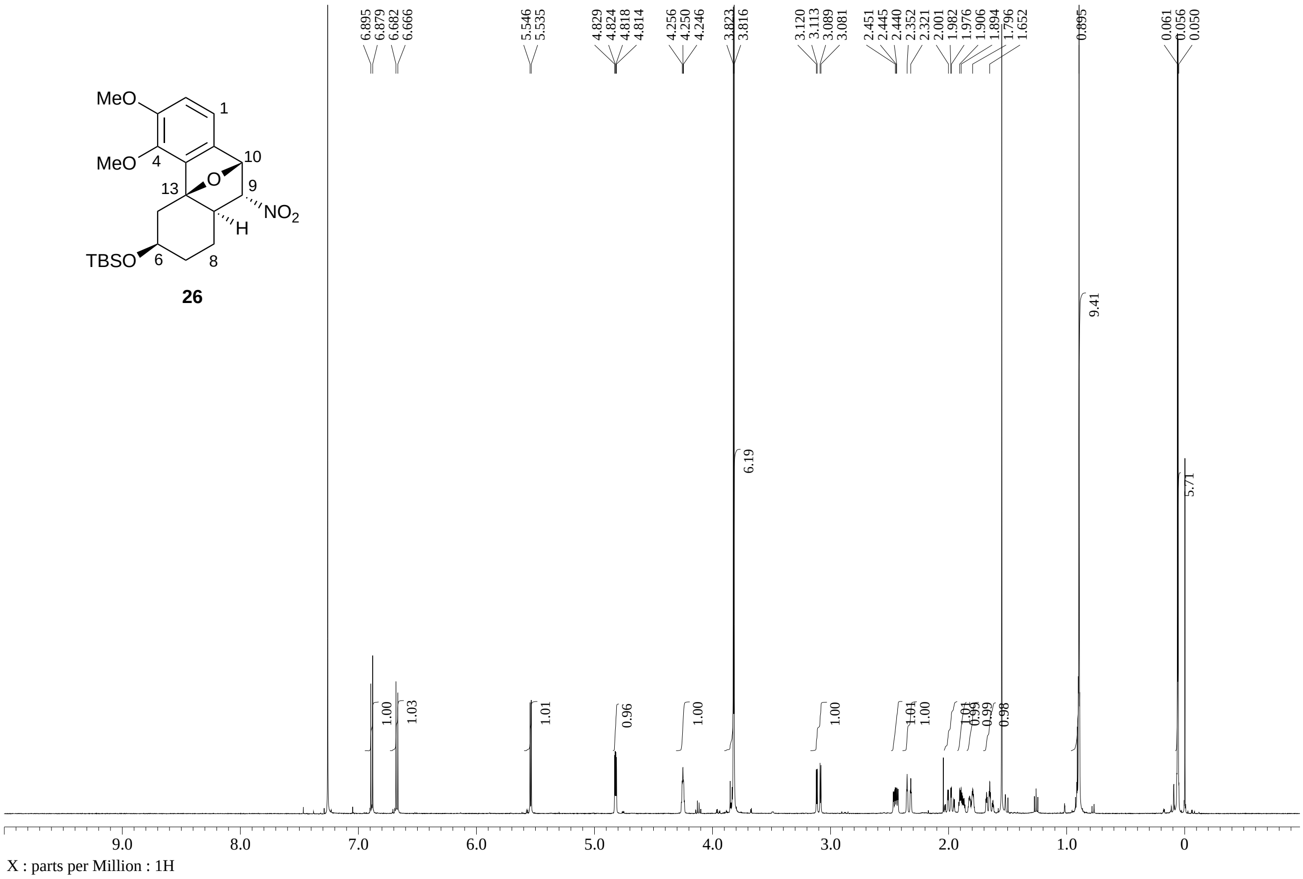

^{13}C NMR spectrum of cycloadduct **26** (126 MHz, $CDCl_3$)

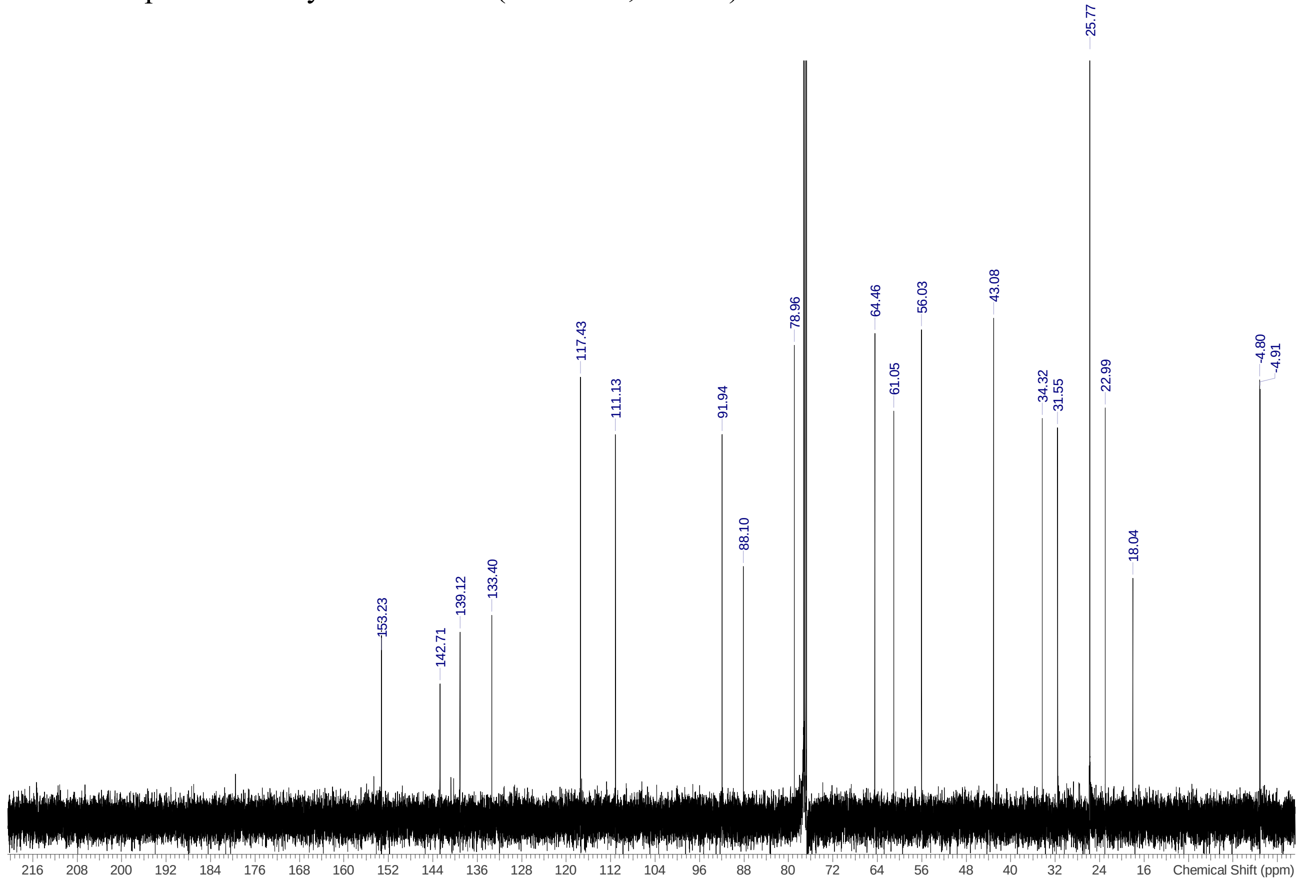

^{1}H NMR spectrum of cycloadduct **27** (500 MHz, $CDCl_3$)

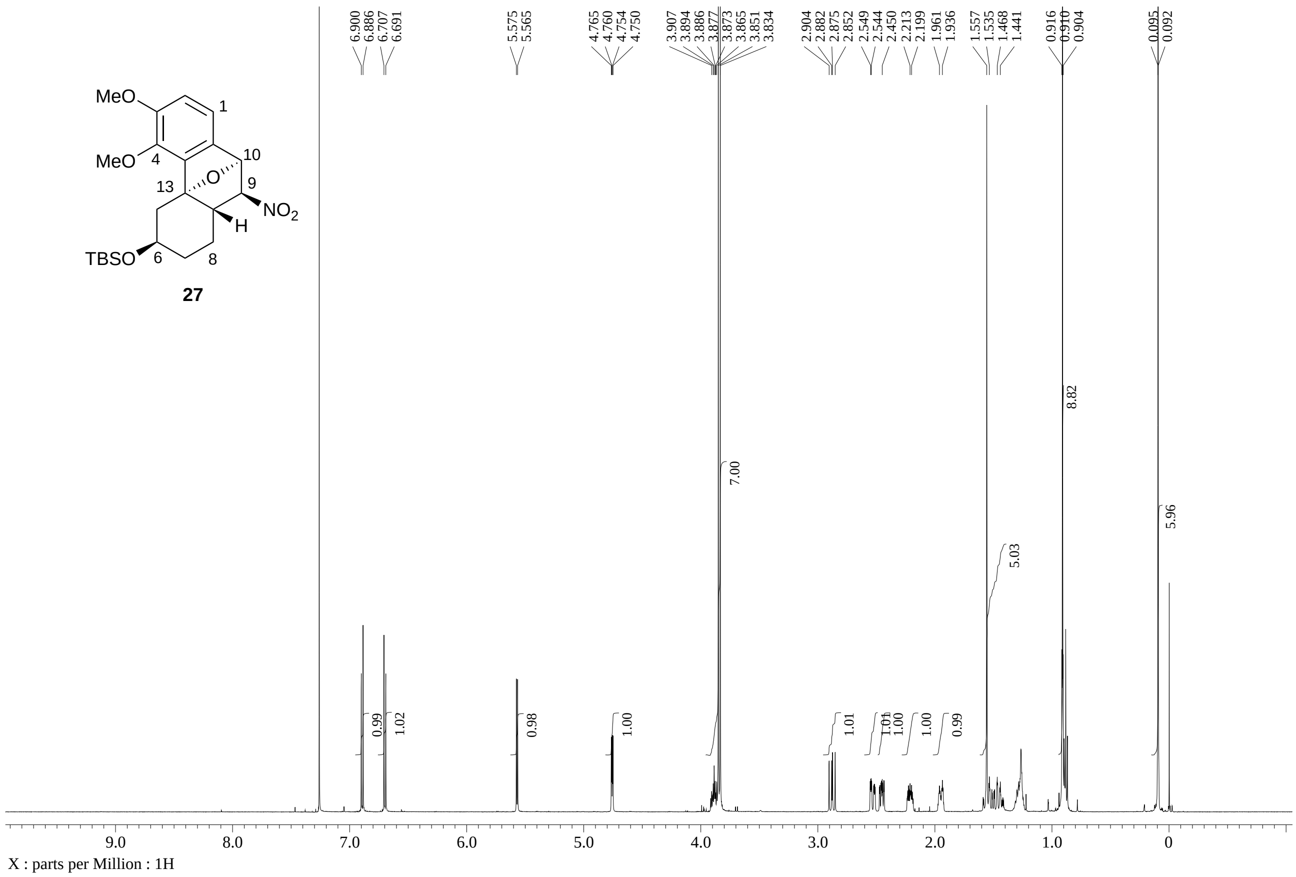

^{13}C NMR spectrum of cycloadduct **27** (126 MHz, $CDCl_3$)

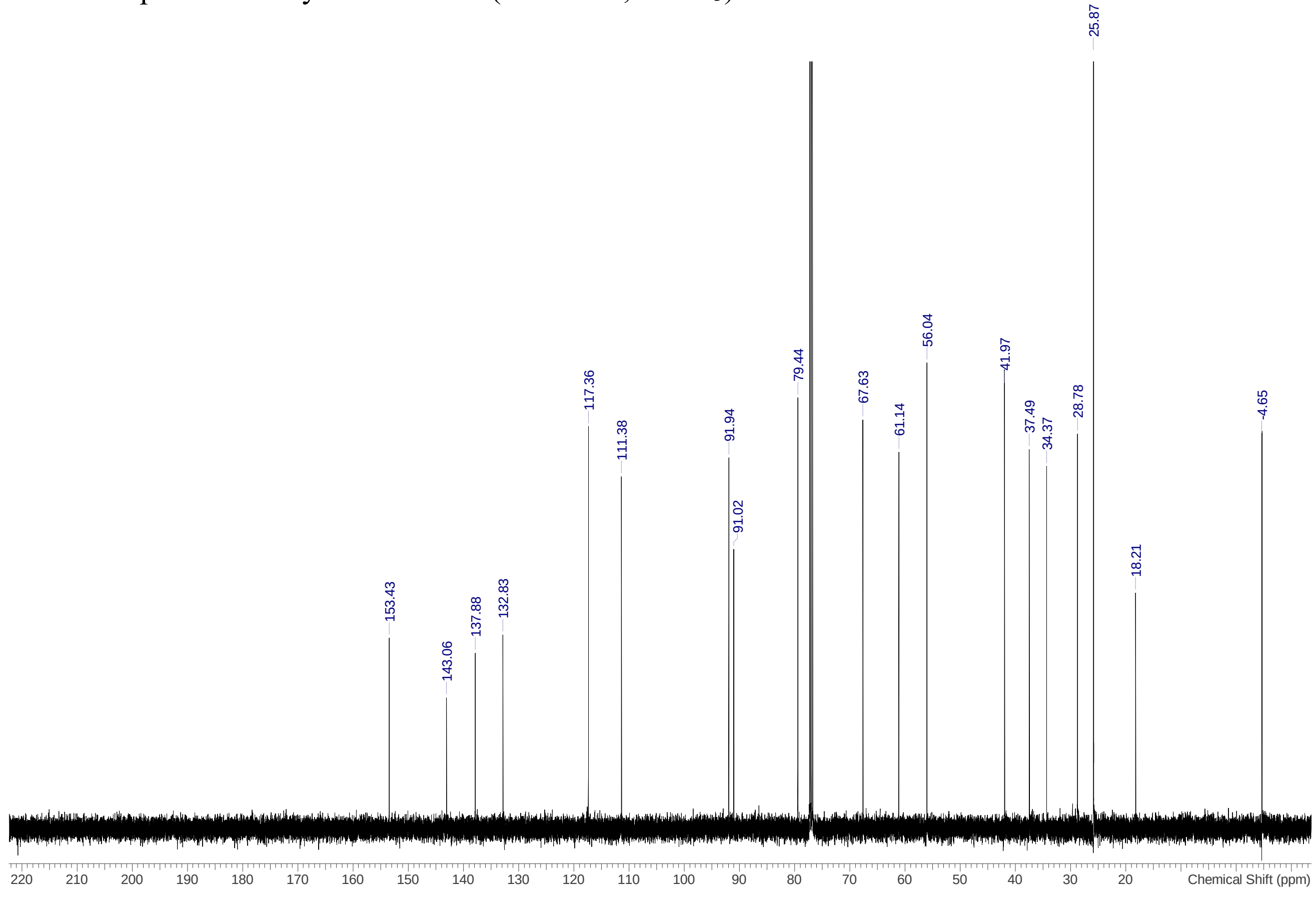

^{1}H NMR spectrum of amine **30** (500 MHz, $CDCl_3$)

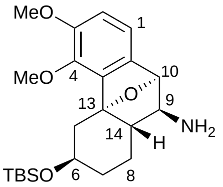

30

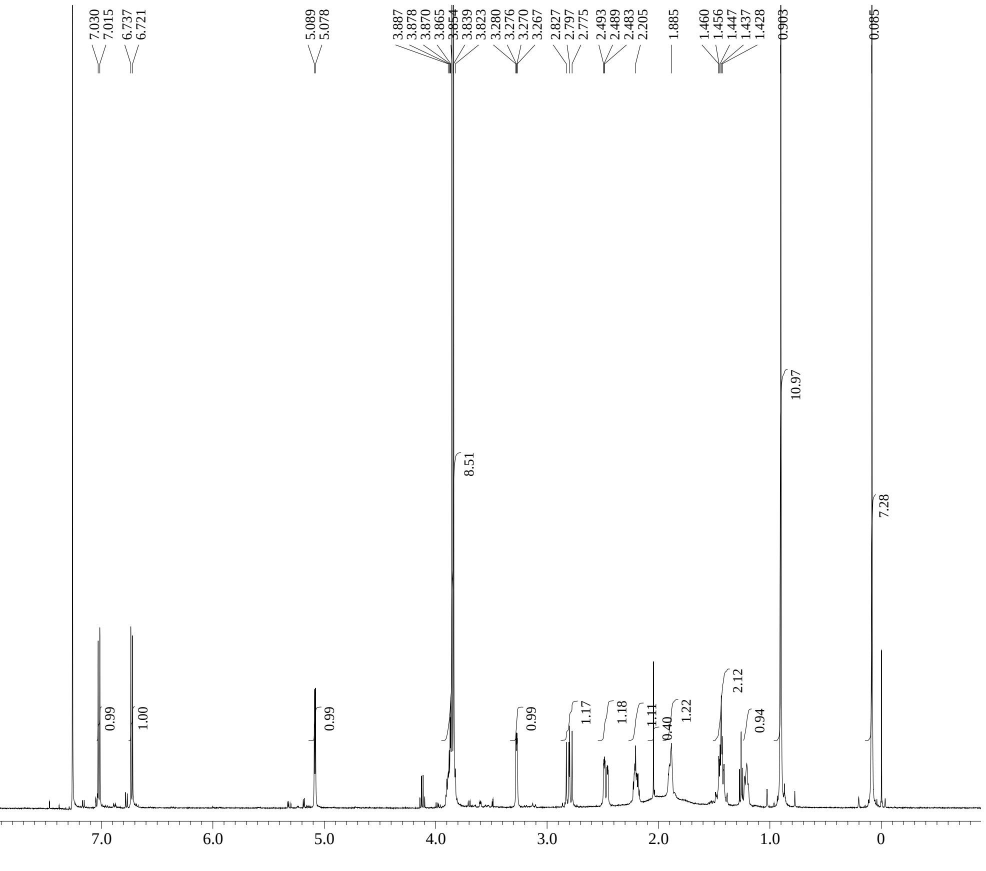

^{13}C NMR spectrum of amine **30** (126 MHz, $CDCl_3$)

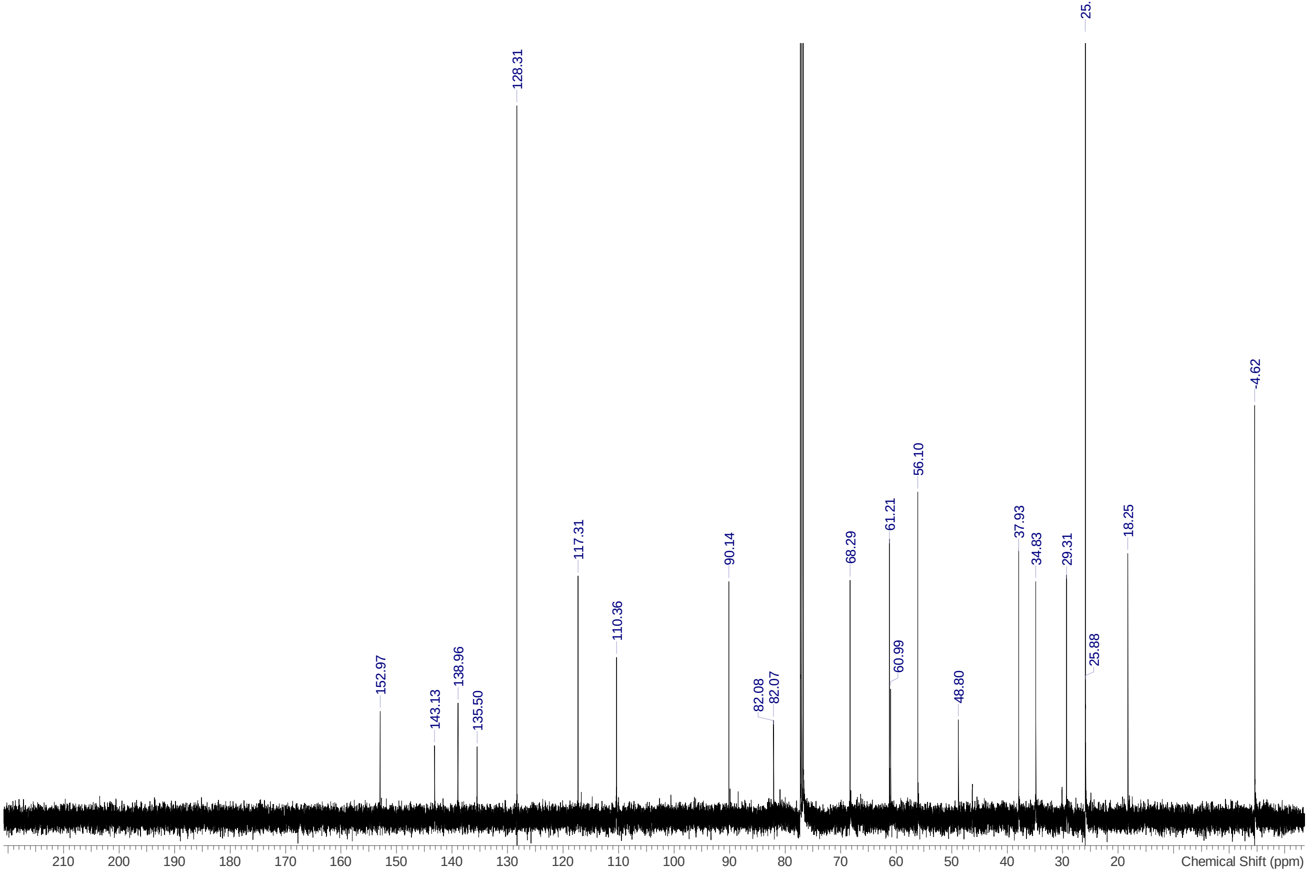

^{1}H NMR spectrum of ring-opening product **31** (500 MHz, $CDCl_3$)

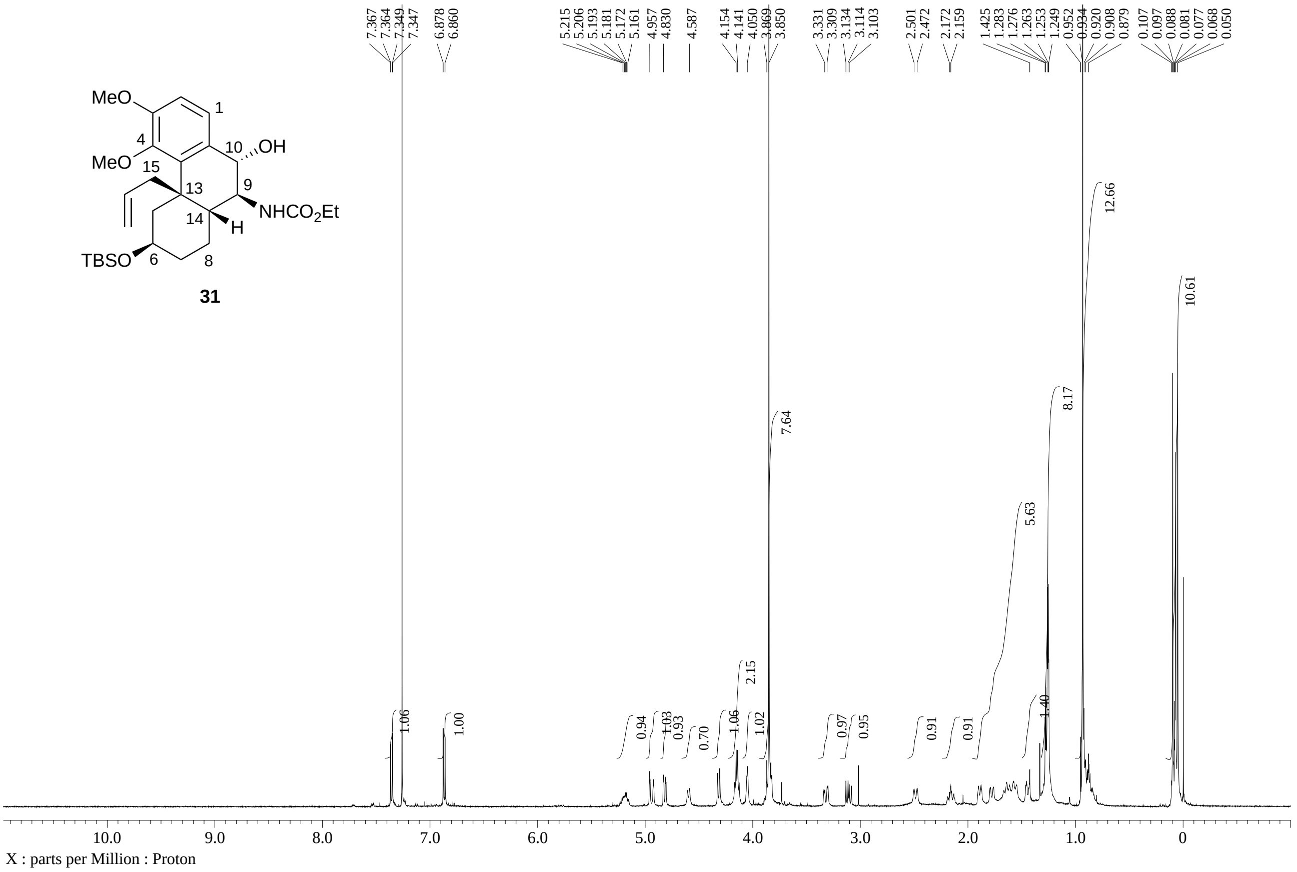

^{13}C NMR spectrum of ring-opening product **31** (126 MHz, $CDCl_3$)

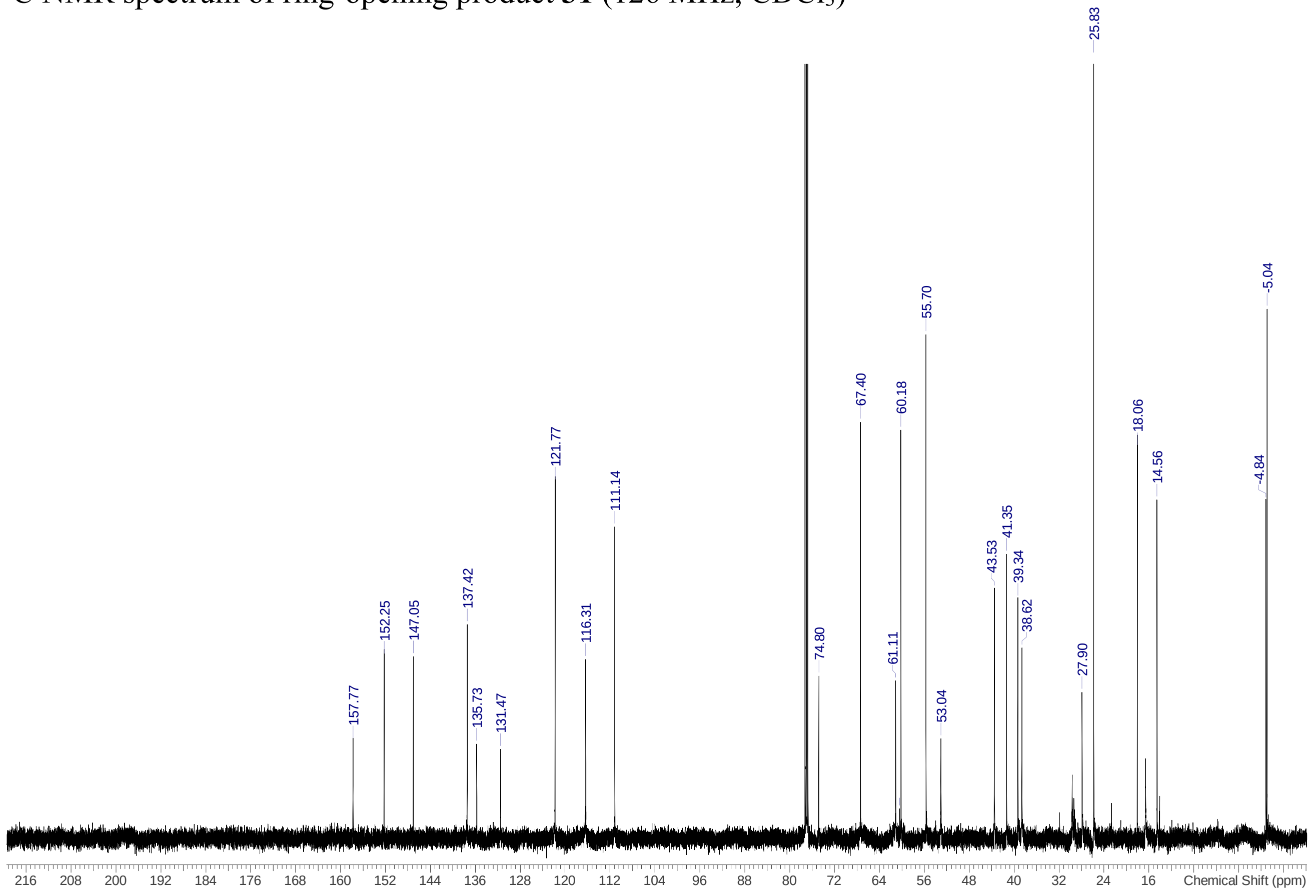

^{1}H NMR spectrum of ring-opening product 13-*epi*-**31** (500 MHz, $CDCl_3$)

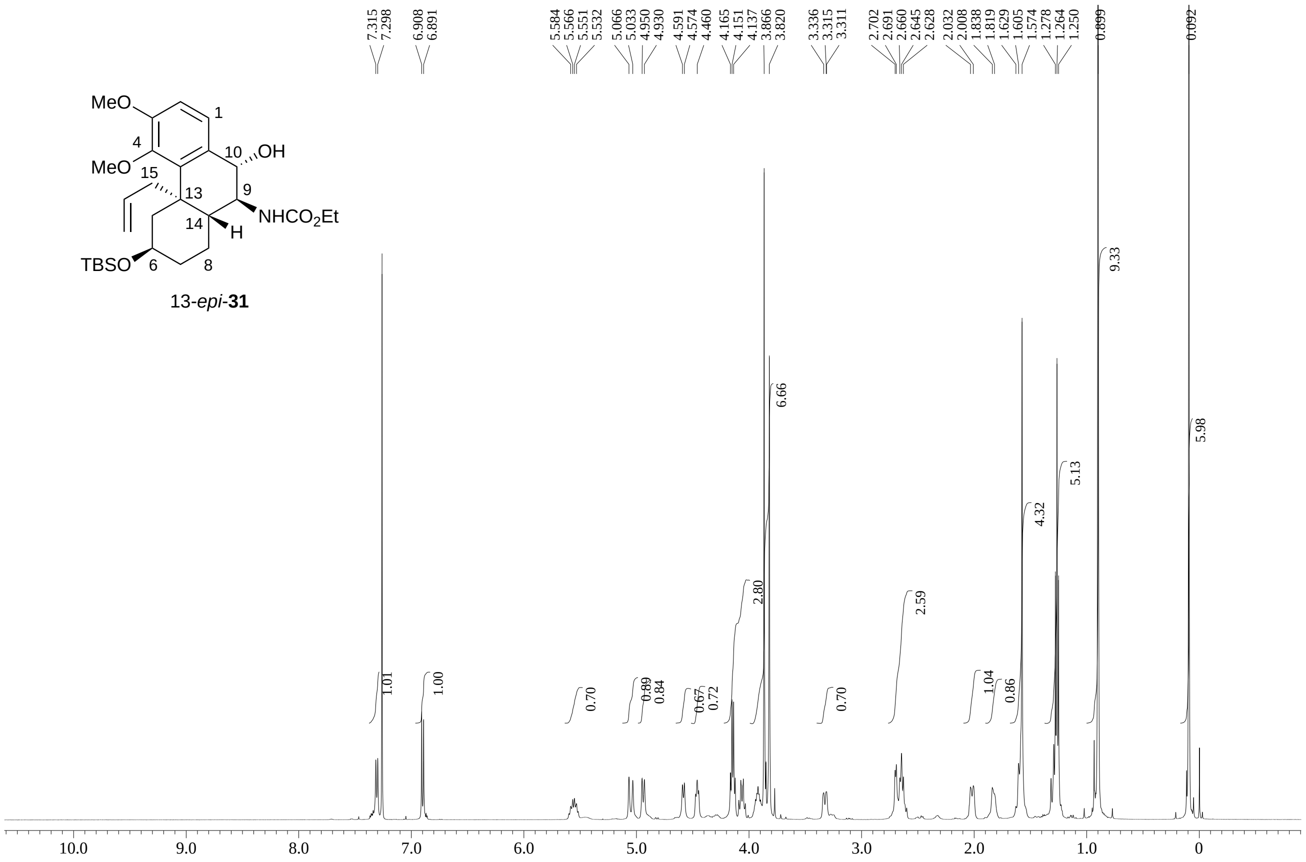

^{13}C NMR spectrum of ring-opening product 13-*epi*-**31** (126 MHz, $CDCl_3$)

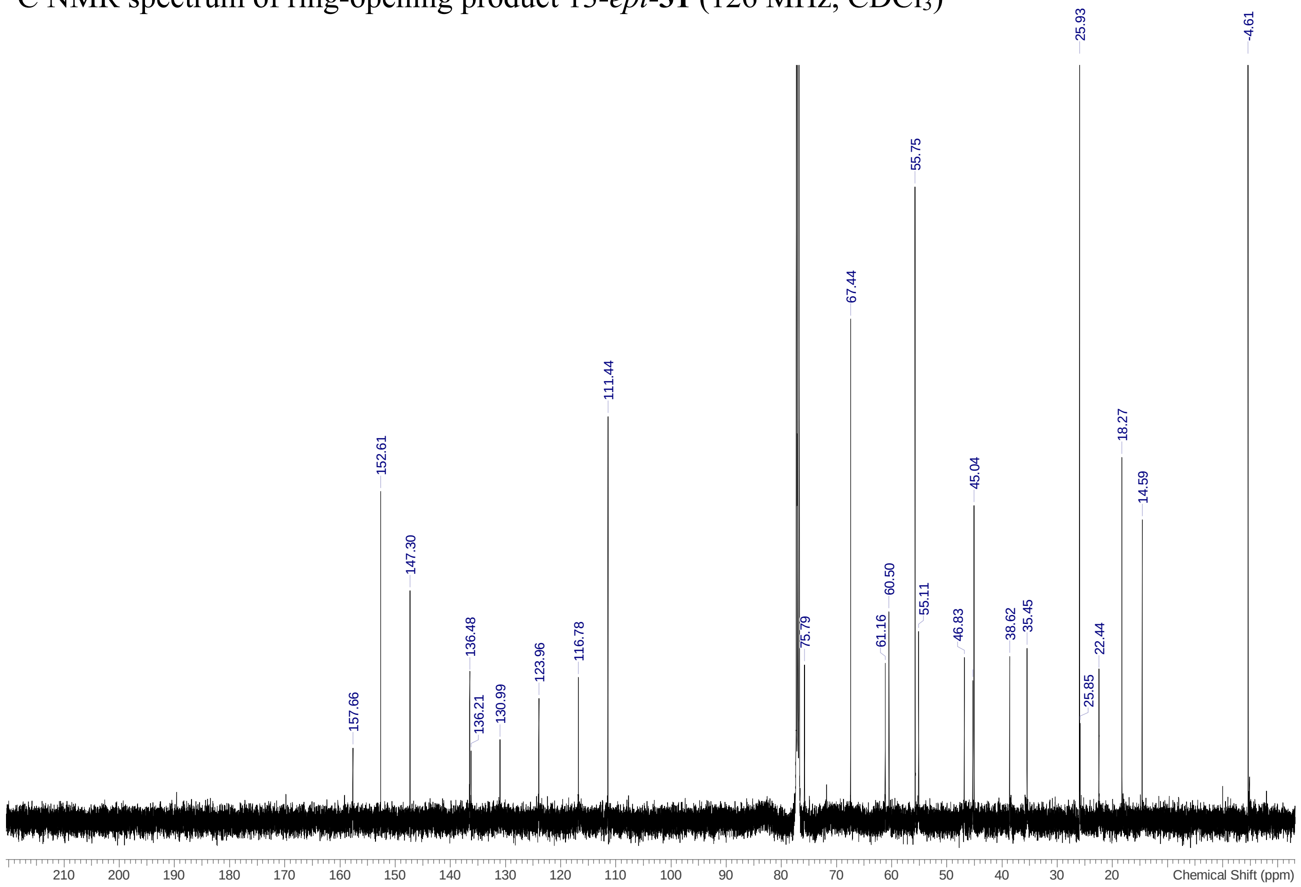

^{1}H NMR spectrum of enecarbamate **32** (500 MHz, $CDCl_3$)

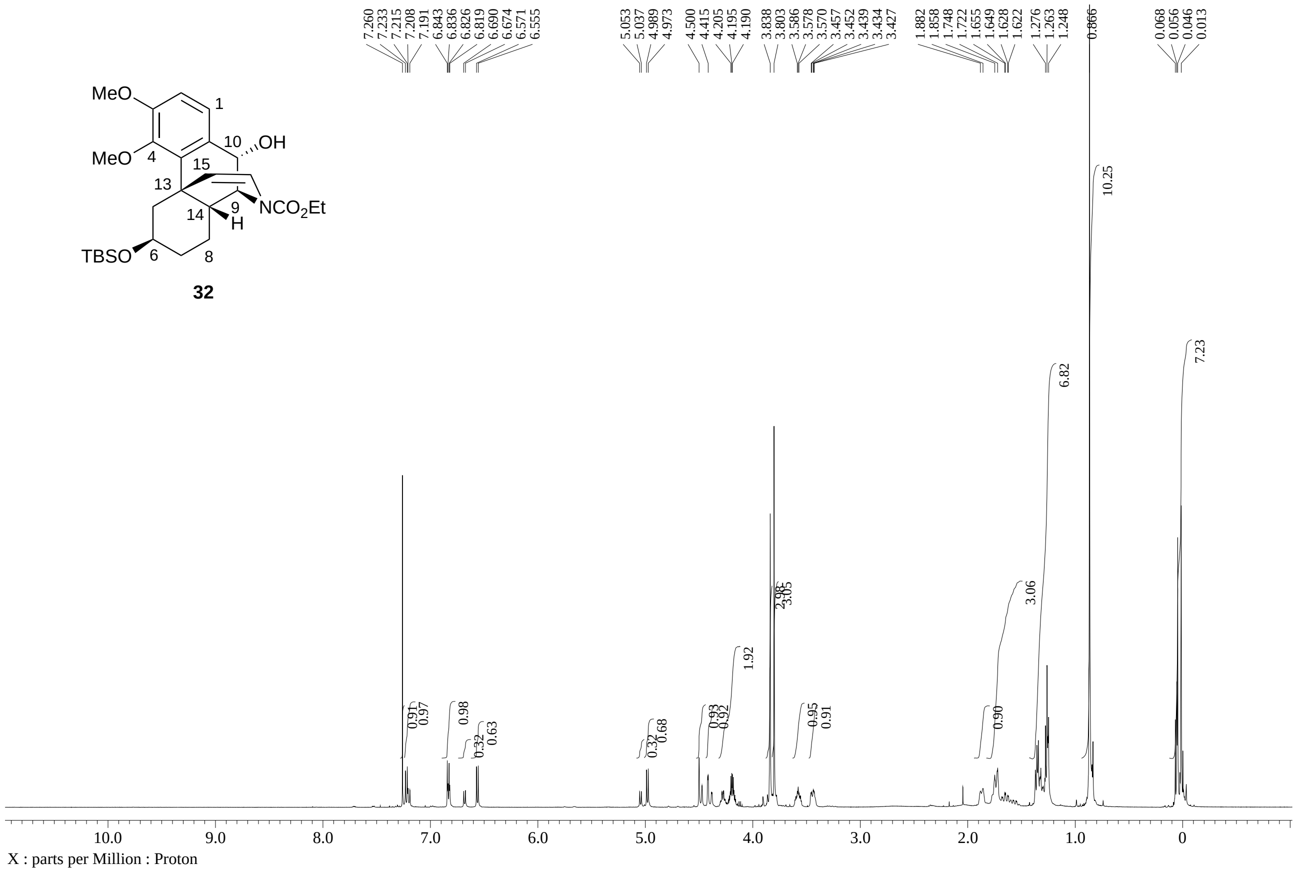

^{13}C NMR spectrum of enecarbamate **32** (126 MHz, $CDCl_3$)

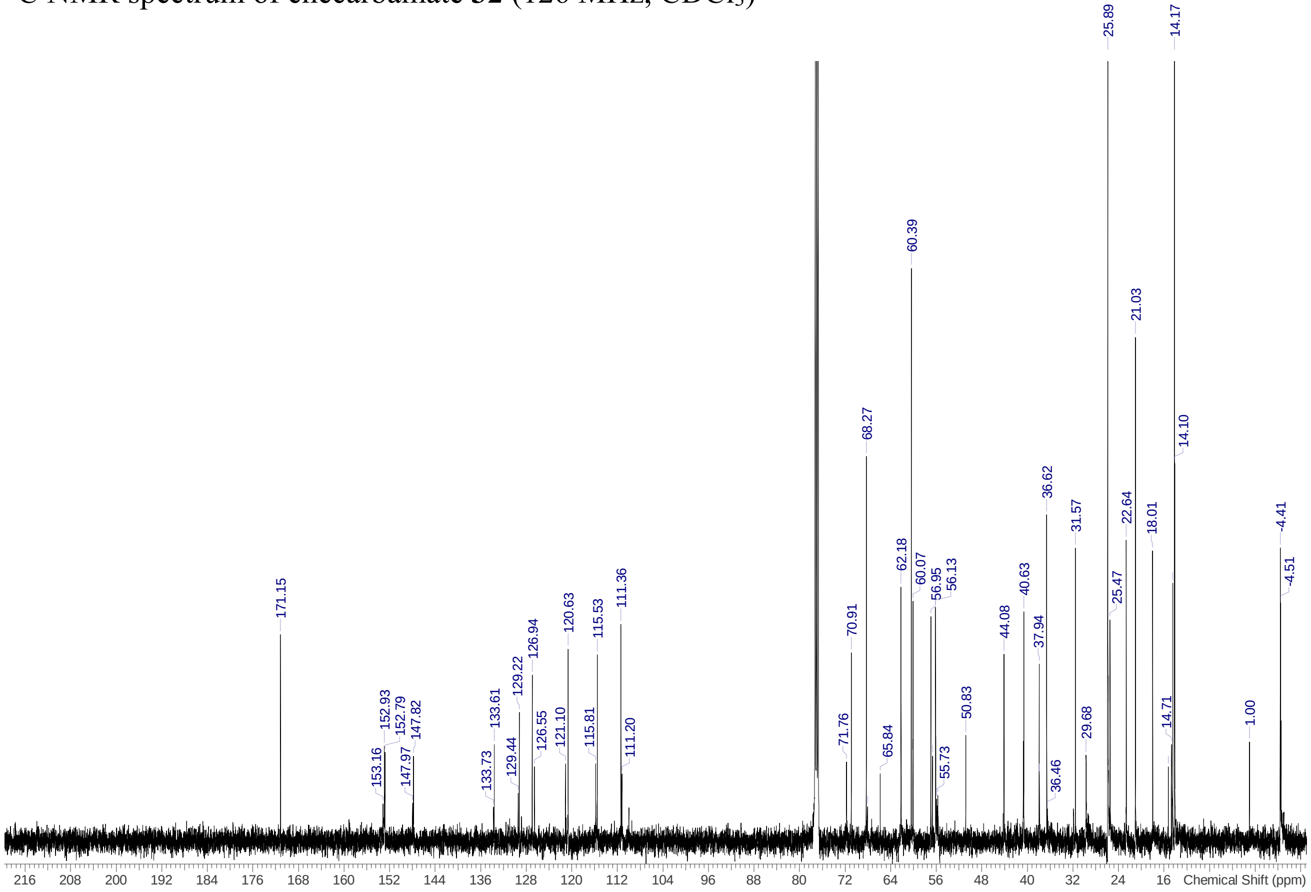

^{1}H NMR spectrum of alcohol **33** (500 MHz, $CDCl_3$, 50 °C)

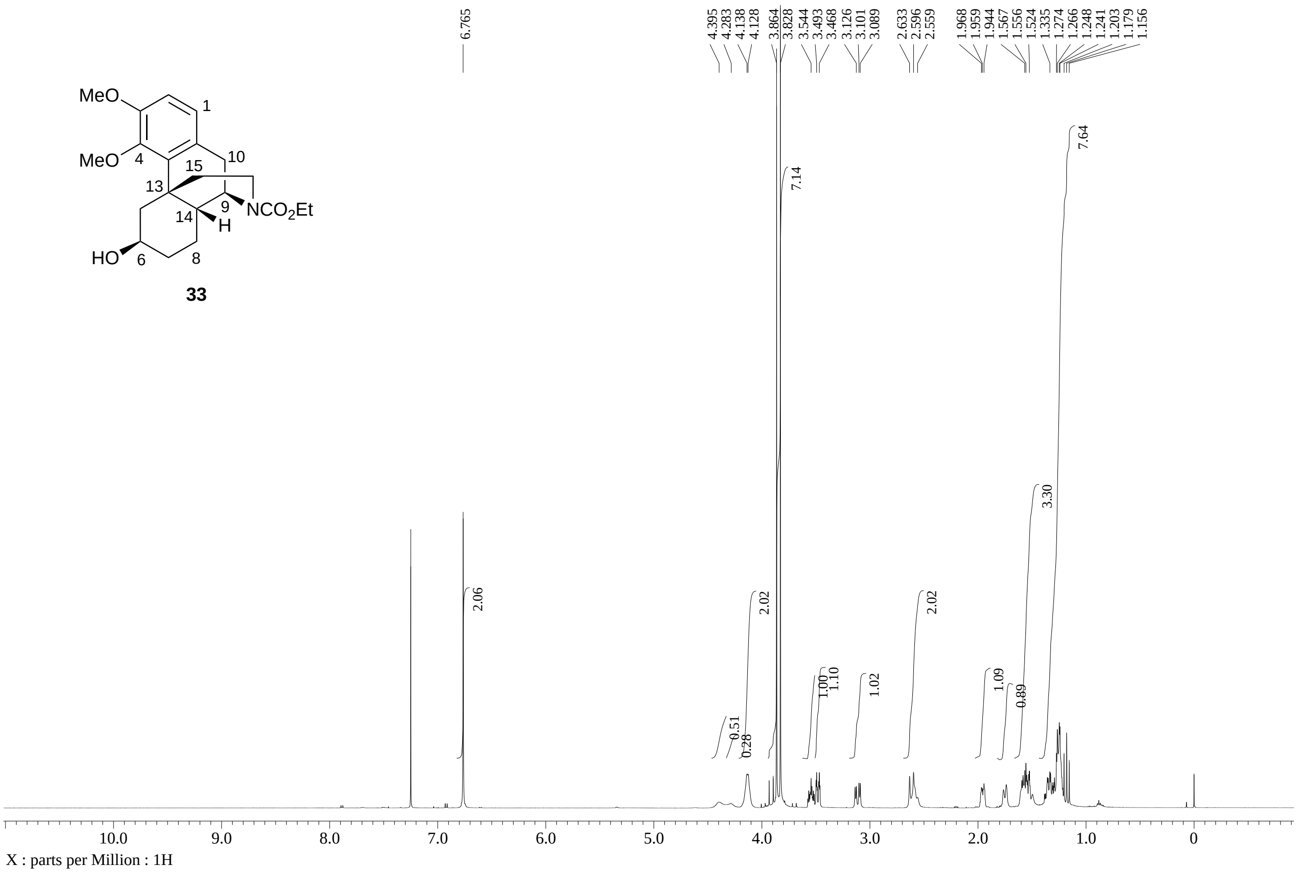

^{13}C NMR spectrum of alcohol **33** (126 MHz, $CDCl_3$)

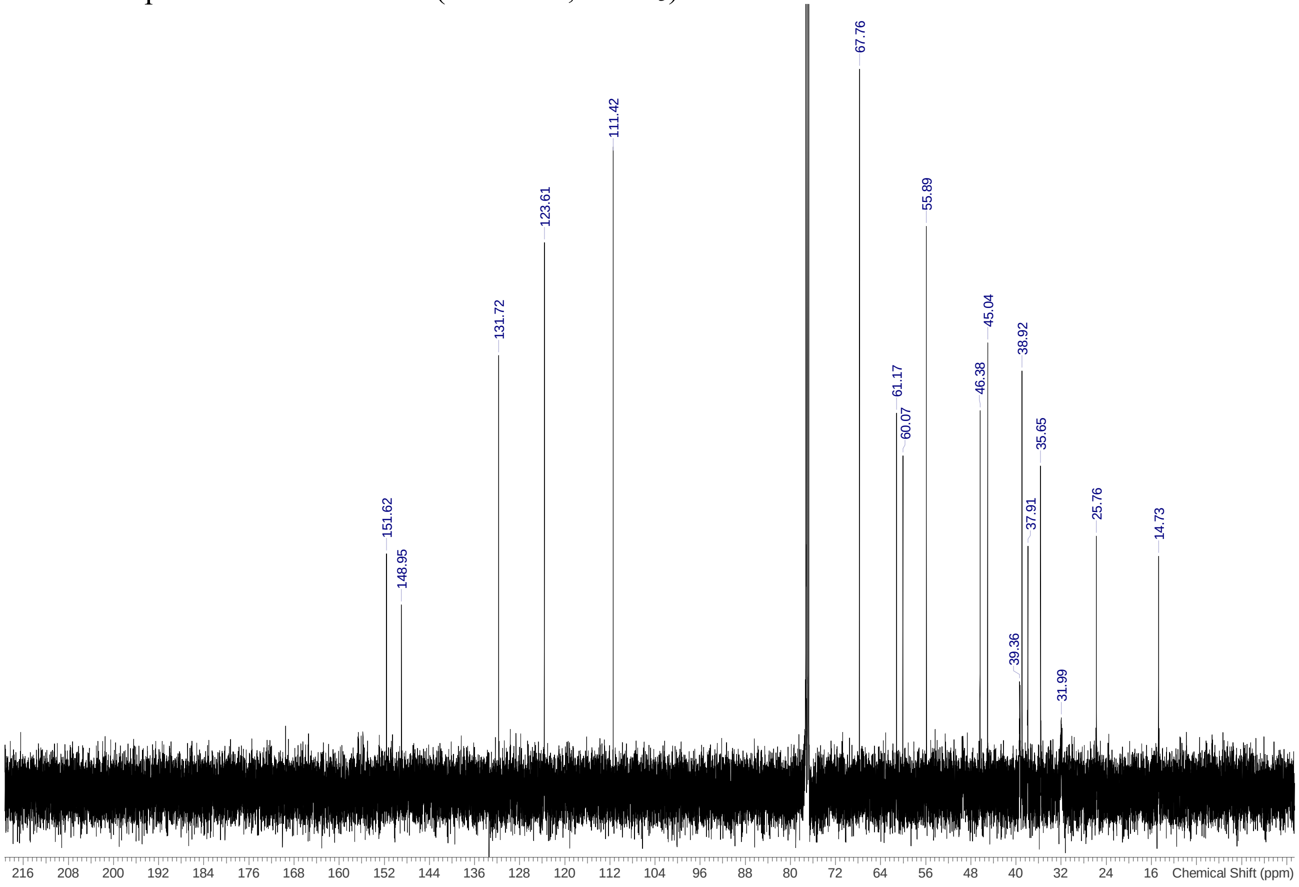

^{1}H NMR spectrum of ketone **34** (500 MHz, $CDCl_3$, 50 °C)

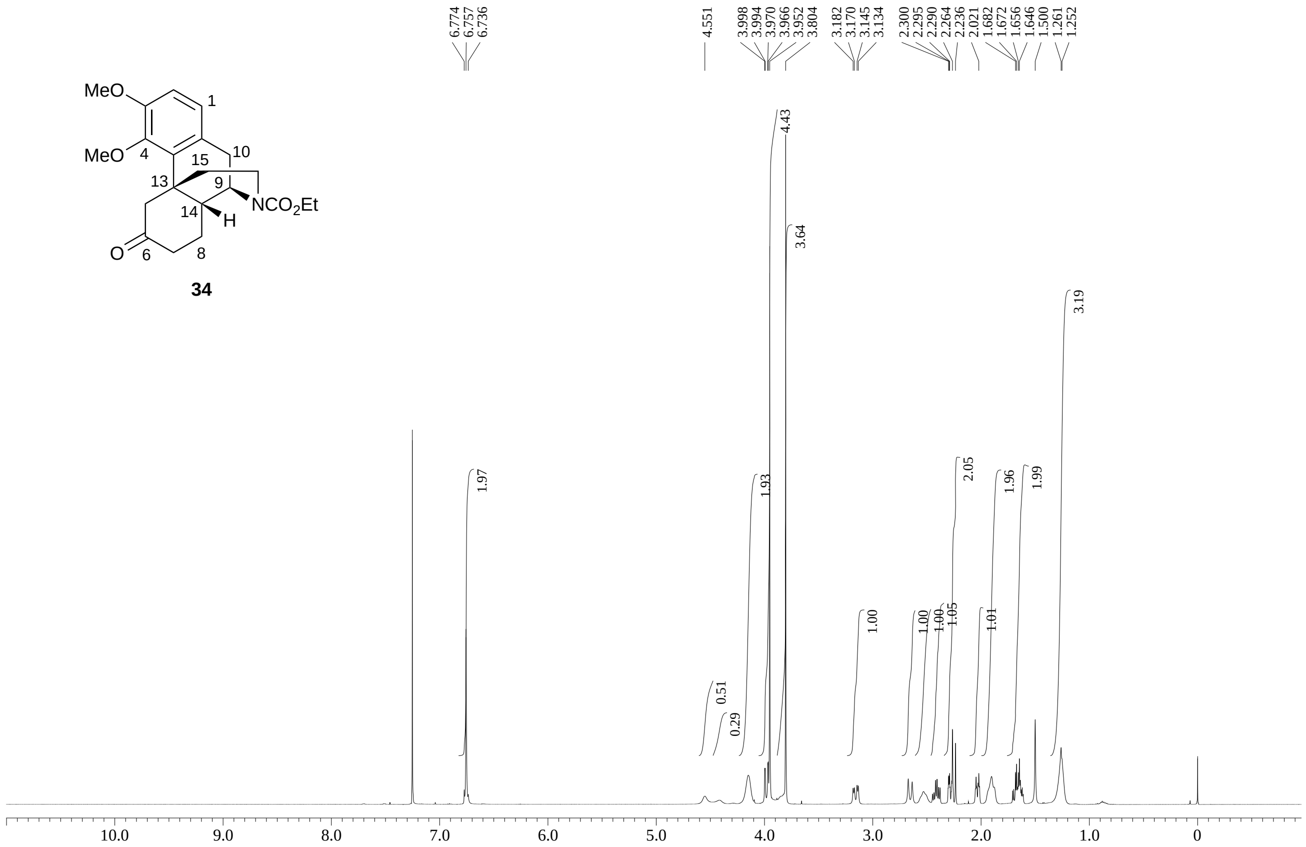

^{13}C NMR spectrum of ketone **34** (150 MHz, $CDCl_3$, 50 °C)

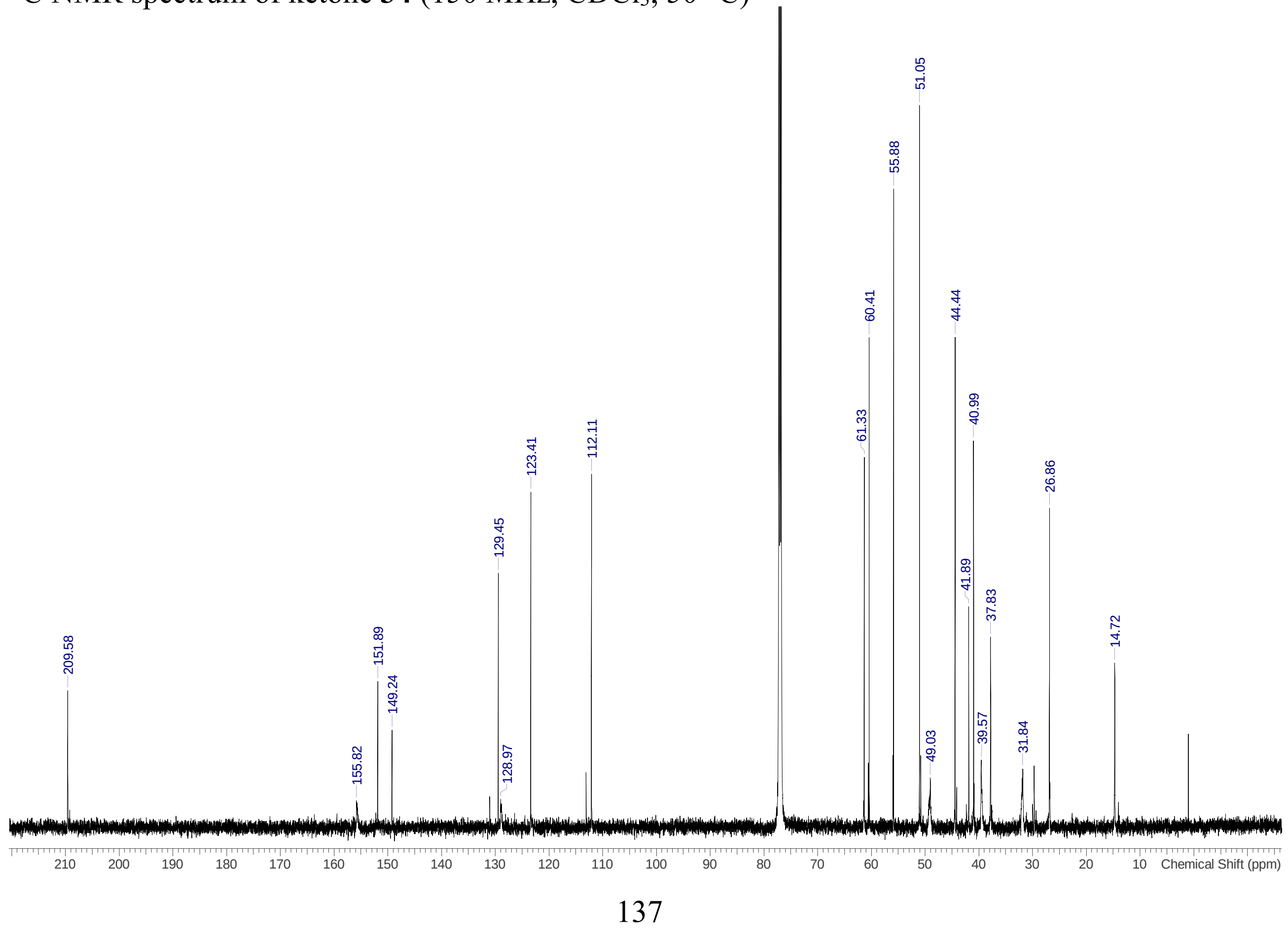

^{1}H NMR spectrum of cyclic ether **35** (500 MHz, $CDCl_3$)

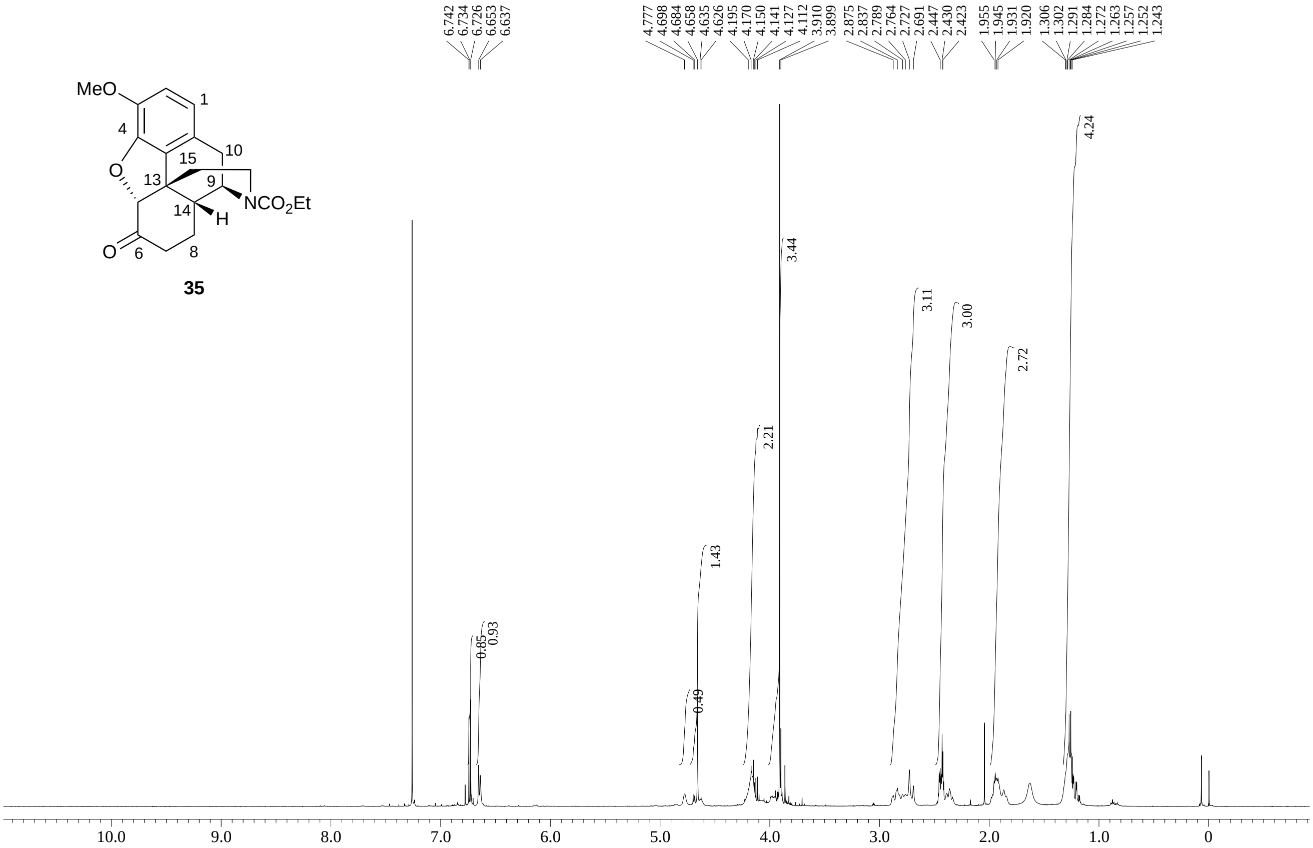

^{13}C NMR spectrum of cyclic ether **35** (150 MHz, $CDCl_3$, 50 °C)

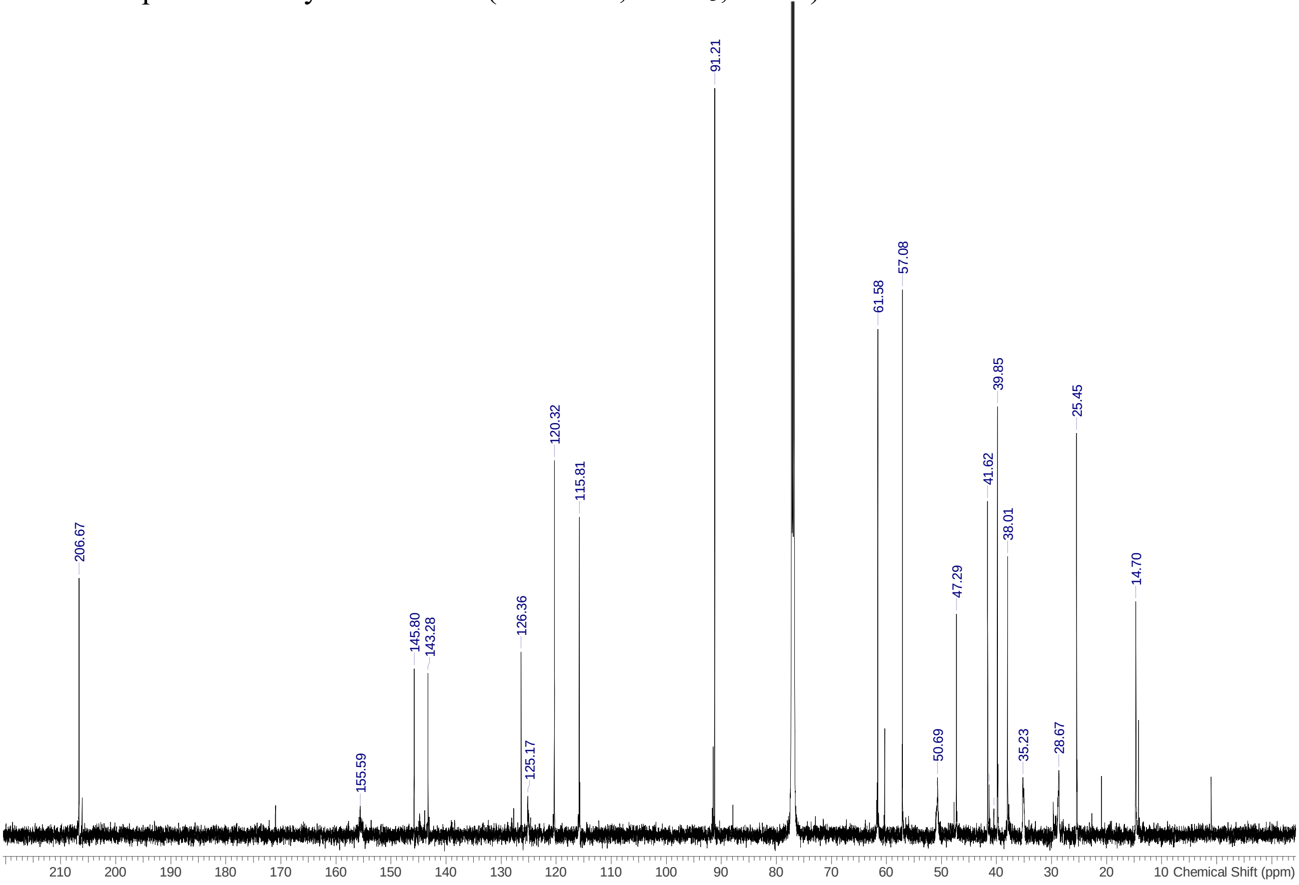

^{1}H NMR spectrum of cyclic ether **35** (500 MHz, pyridine-d_5, 80 °C)

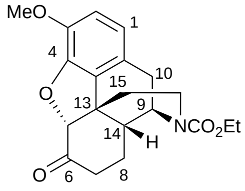

35

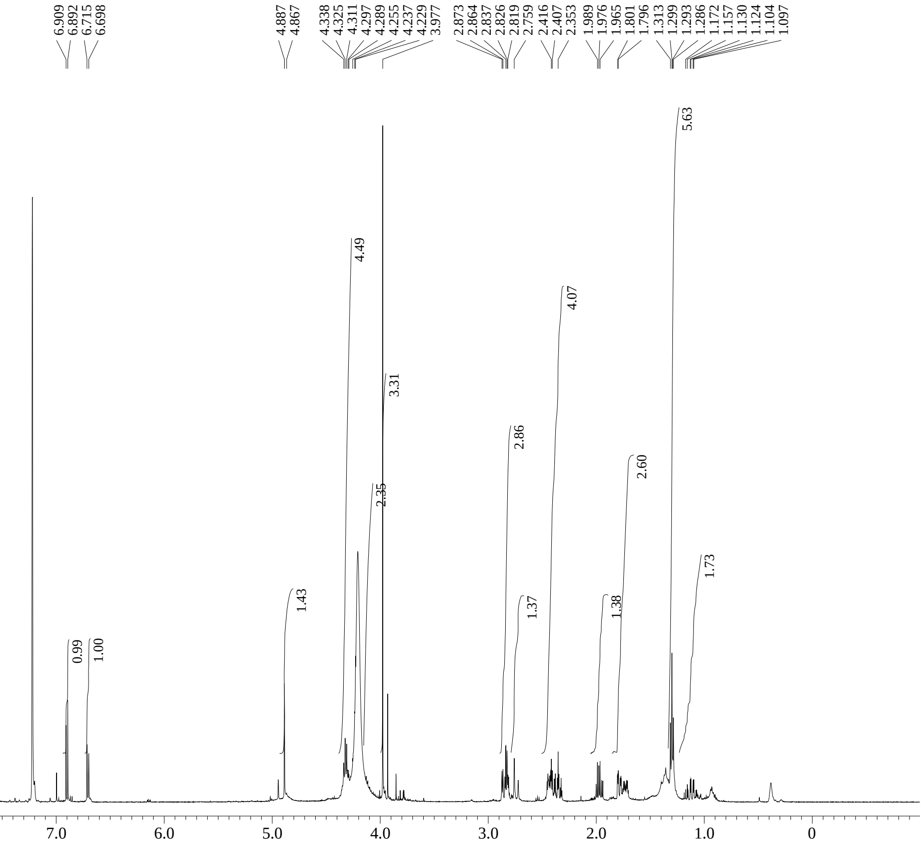

7. NOESY Correlation Diagram for Cycloadduct 3, and Copy of NOESY Spectrum

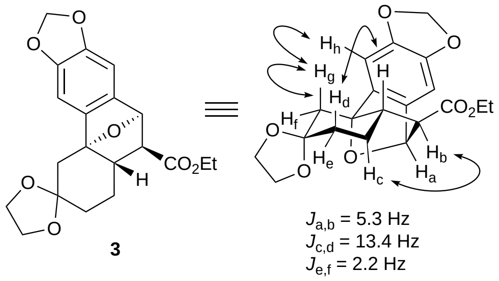

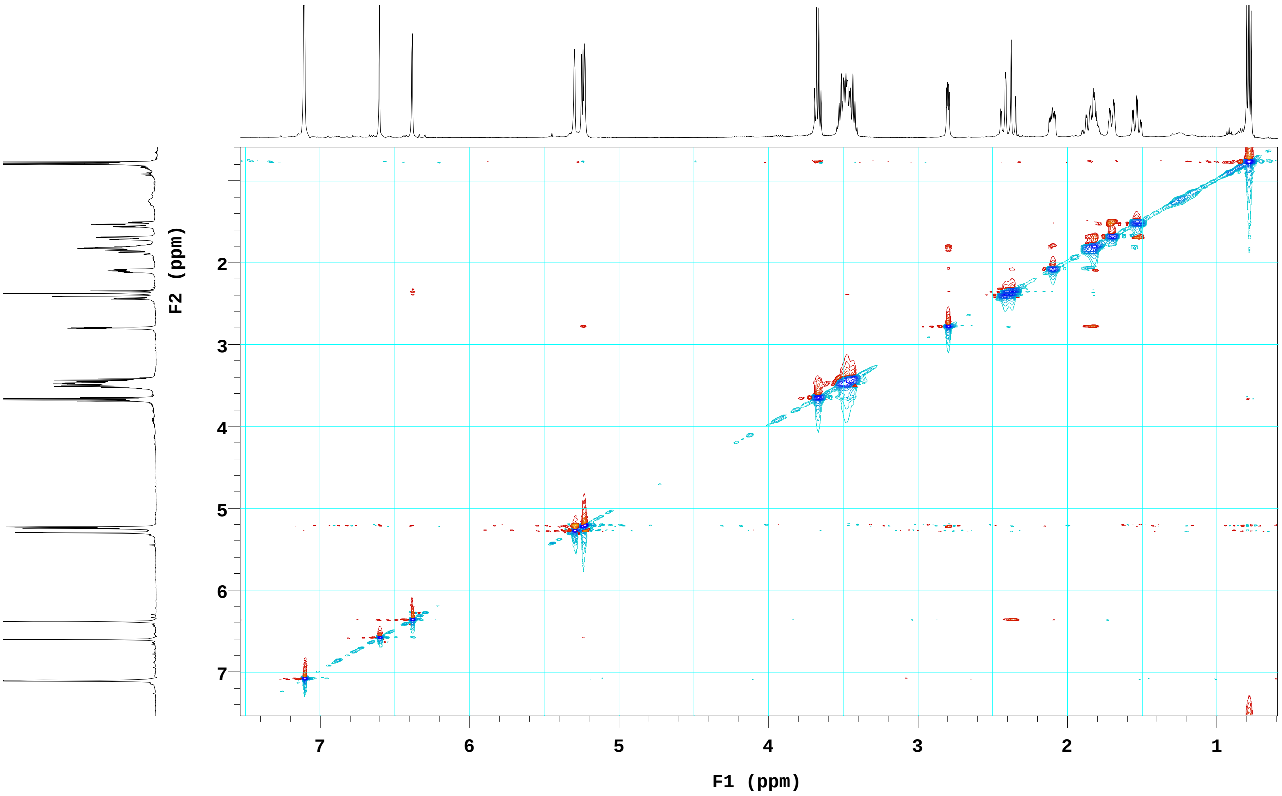

expanded spectrum

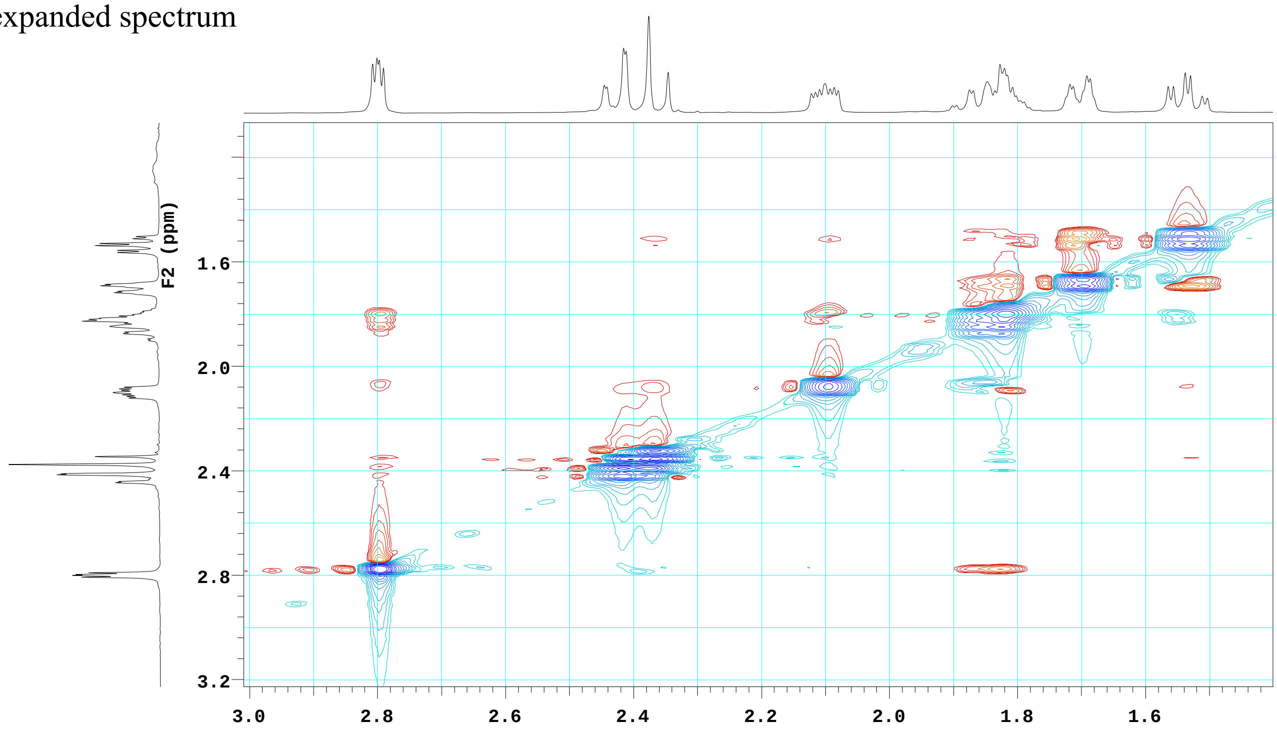

Supplement: SC-015-D4SC05890A-s001 [file SC-015-D4SC05890A-s001.pdf]
